# Supplementary material for: Ehbp1 orchestrates orderly sorting of Wnt/Wingless to the basolateral and apical cell membranes
Source: EMBO Rep. 2024 Oct 14;25(11):5053–79. doi: 10.1038/s44319-024-00289-1 (PMC11549480; doi:10.1038/s44319-024-00289-1)
Supplement: Supplementary file 1 — Appendix [file 44319_2024_289_MOESM1_ESM.pdf]

Gao et al., Appendix

Appendix Figure S1……………………………………………………………………………1

Appendix Figure S2……………………………………………………………………………2

Appendix Figure S3……………………………………………..…….………………………4

Appendix Table S1…………………………….………………………………………………6

Appendix Table S2……………………………………………………..………………………8

Appendix Figures

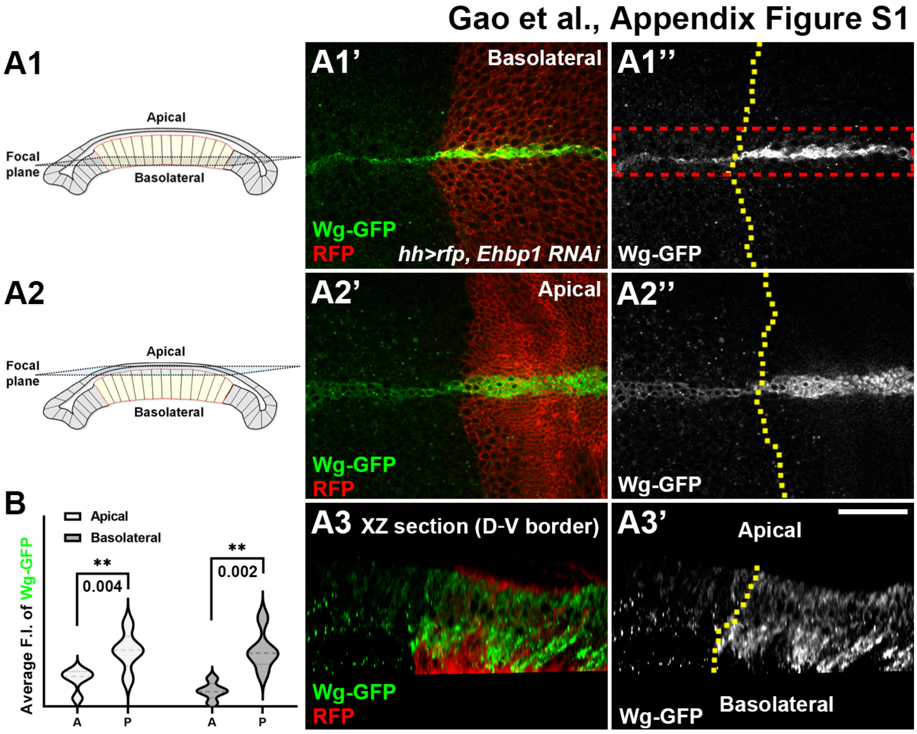

Appendix Figure S1. Ehbp1 maintains the proper activity of Wg secretion.

(A1-A3') The basolateral (A1-A1'') and apical (A2-A2'') membrane domains of immunofluorescence staining of ExWg for the indicated genotypes are shown. When RNAi against *Ehbp1* was expressed using the *hh-Gal4* driver, Wg-GFP accumulated at both the D-V boundary of basolateral domains and the apical domains in the posterior compartments of the wing discs. A 3D reconstruction of the D-V border cells (as viewed in an XZ section) of the wing disc expressing RNAi against *Ehbp1* shows the accumulation of Wg-GFP at both the basolateral and apical compartment (A3-A3'). Dotted yellow lines indicate the A-P boundaries.

(B) The statistical analysis of Wg-GFP fluorescence intensity (F.I.) at the D-V boundary in both the basolateral (A1'') and apical sections (A2'') was performed (for each genotype, $n \geq 3$). Data are presented as violin plots. Two-tailed Student's t-tests were employed to analyze the differences between anterior and posterior F.I. $^{**}p < 0.01$.

Scale bar, 25 µm.

Gao et al., Appendix Figure S2

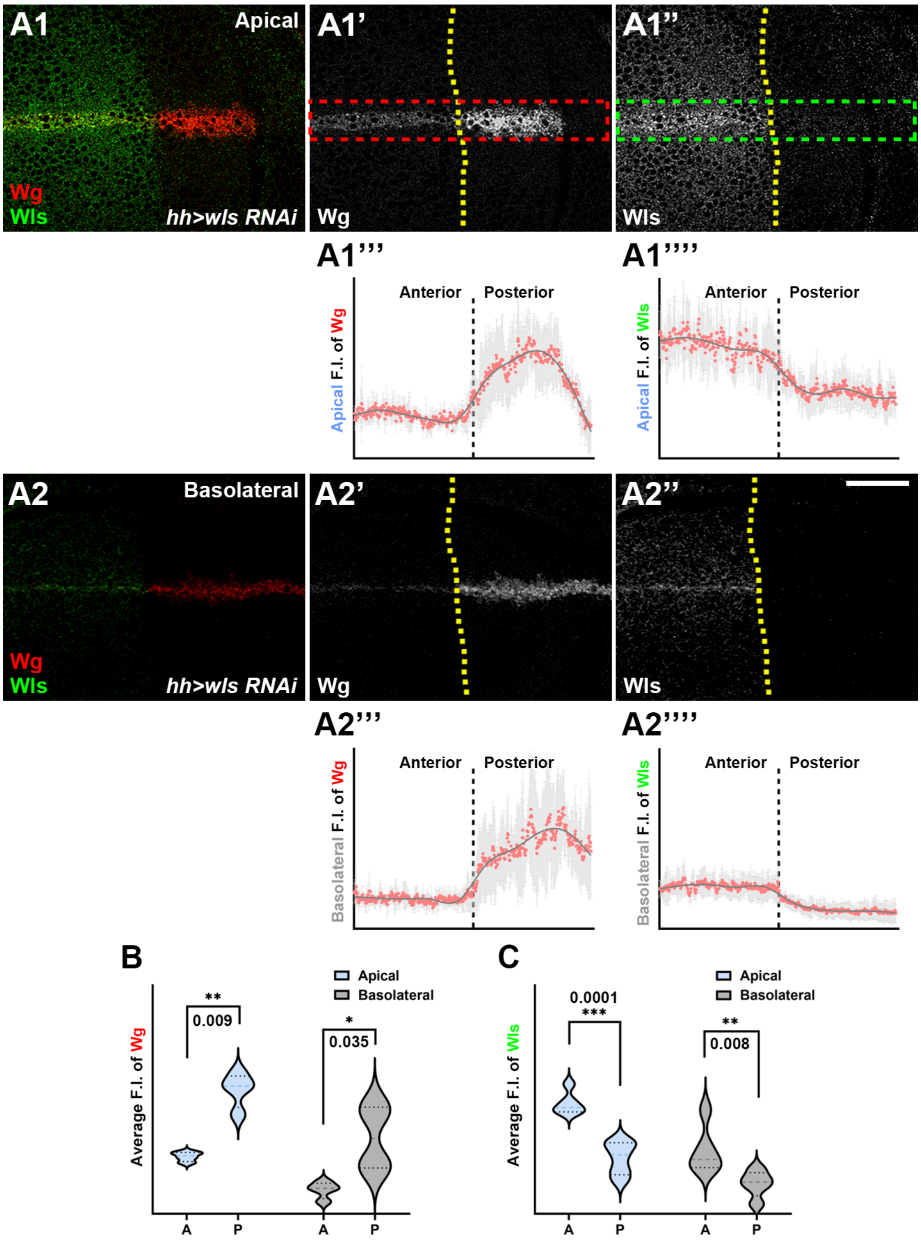

Appendix Figure S2. Validation of the specificity of the Wls antibody in wing discs.

(A1-A2'''') The apical (A1-A1'') and basolateral (A2-A2'') sections of Wls immunofluorescence staining are shown. Wls expression was detected in both basolateral and apical regions of the wing disc, with higher staining intensity along at the D-V boundary. However, this pattern was not observed in the posterior compartment when RNAi against *wls* was expressed using the *hh-Gal4* driver. Instead, a large amount of Wg accumulated at the D-V boundary, a known effect resulting from depleting *wls*. Plot profiles of immunofluorescence staining were generated in the rectangular areas demarcated by red (for Wg) and green (for Wls) dashed lines (for each genotype, $n \geq 3$). Dotted yellow lines indicate the A-P boundaries.

(B-C) The statistical analysis of Wg and Wls immunofluorescence intensity (F.I.) at the D-V boundary in both the basolateral and apical membrane domains was performed. The analyzed regions were demarcated by rectangles indicated by red and green dashed lines. An example of this analysis can be seen in panels A1-A1''. Immunofluorescence intensity data are presented as mean ± SD (for each genotype, $n \geq 3$). Two-tailed Student's t-tests were used to analyze the differences between anterior and posterior F.I. **P* < 0.05. ***P*<0.01. ****P*<0.001.

Scale bar, 25 μm.

Gao et al., Appendix Figure S3

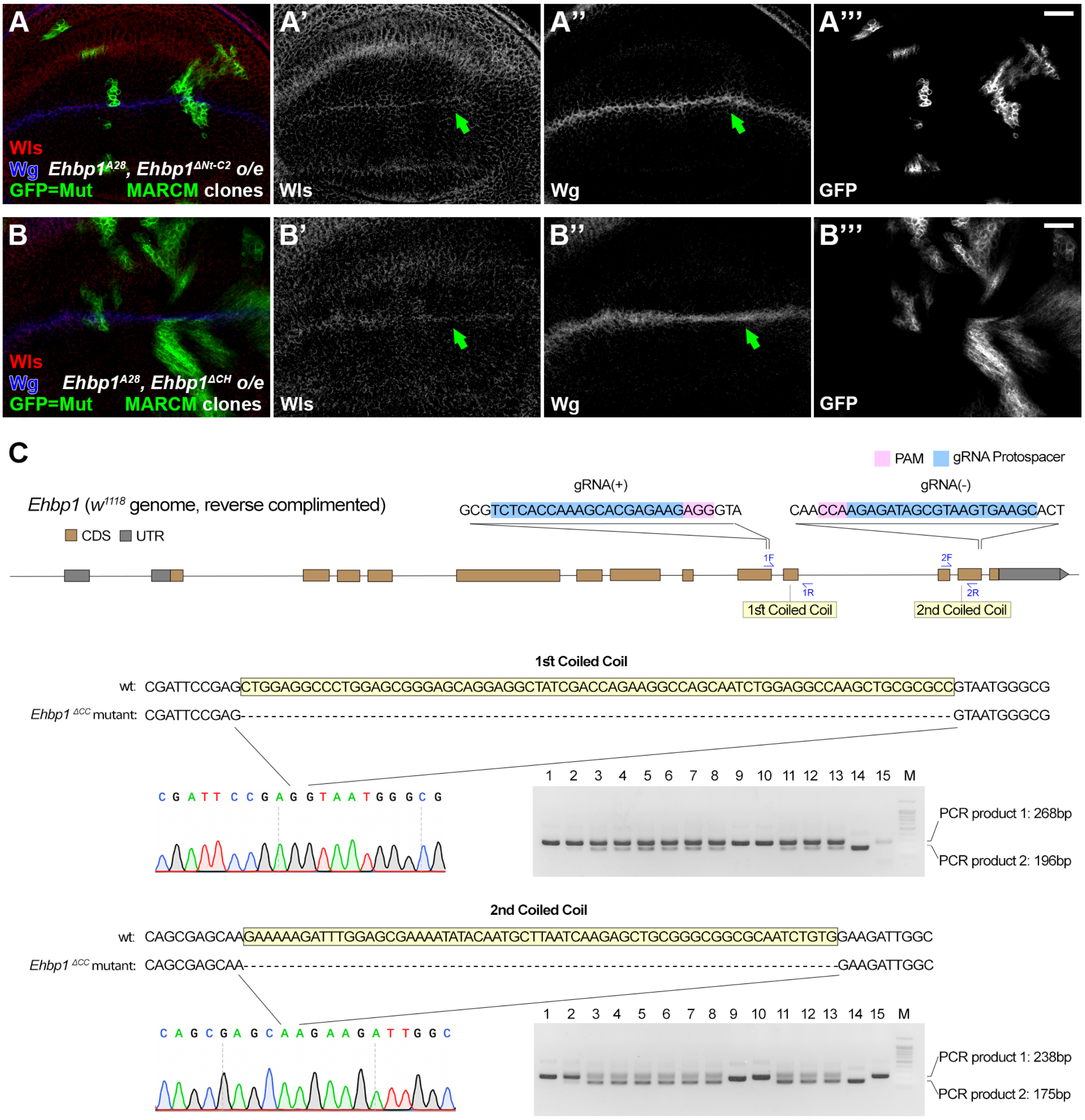

Appendix Figure S3. The Nt-C2 and CH domains are not required for Ehbp1 to antagonize the AP-1 complex.

(A-B''') In *Ehbp1*^{A28} mutant somatic clones (positively marked by GFP), the expression of *Ehbp1*$^{\Delta Nt\text{-}C2}$ or *Ehbp1*$^{\Delta CH}$ fully rescued the accumulation of Wg and Wls at the D-V boundary in the basolateral domains of the wing disc. Arrows indicate the somatic clones at the D-V boundary.

Scale bars, 25 µm.

(C) Strategy for generating *Ehbp1*$^{\Delta CC}$ mutant flies. Two guide RNAs (gRNA) were designed to target the genomic DNA, resulting in the deletion of two Coiled-Coil motifs. A template lacking the coding sequences

for Coiled-Coil motifs was used for homology-directed repair (HDR). PCR identification confirmed the deletion of Coiled-Coil motifs in the resulting knockout flies.

Appendix Tables

Appendix Table S1. Genetic crosses for figures and supplemental figures.

Figure	Genotype	Experimental condition
1C	*hh-Gal4, UAS-mCD8-gfp / UAS-w RNAi*	25ºC
1C, 1D	*wg-Gal4, UAS-gfp / + ; UAS-w RNAi / +*	25ºC
1E	*wg-Gal4, UAS-gfp / + ; UAS-mir-bft / +*	25ºC
1F	*wg-Gal4, UAS-gfp / UAS-Ehbp1 RNAi*	25ºC
1G, 1J, EV2B,	*tub-Gal80$^{[ts]}$ / + ; hh-Gal4, UAS-mCD8-gfp / UAS-mir-bft*	18ºC for 10 ~ 12 days, 29 ºC for 24 ~ 32 hrs
1H, 1I, 2C1, 2C2, 2C3, 2C4, 2D, 3B, 3E, 4K, 4L	*tub-Gal80$^{[ts]}$ / UAS-Ehbp1 RNAi ; hh-Gal4, UAS-mCD8-gfp / +*	18ºC for 10 ~ 12 days, 29 ºC for 36 ~ 44 hrs
1J	*UAS-Ehbp1 RNAi / wg-lacZ ; hh-Gal4, UAS-mCD8-gfp / +*	25ºC
2A, 2B, EV3A, EV3B, EV3C, 3A	*hs-Flp^{122} / + ; FRT42D, M2(58)f, ubi-gfp / FRT42D, Ehbp1^{A28}*	25ºC except for heat shocking at 37ºC for 0.5 hour, recovering for 3 days before dissection
EV3D	*ap-Gal4, UAS-myr-mRFP / Ehbp1 RNAi ; dpp-gfp / +*	25ºC
EV3E	*ap-Gal4, UAS-myr-mRFP, hh-gfp / Ehbp1 RNAi*	25ºC
3C, 3E, S2A, S2B, S2C,	*tub-Gal80$^{[ts]}$ / + ; hh-Gal4, UAS-mCD8-gfp / UAS-wls RNAi*	18ºC for 10 ~ 12 days, 29 ºC for 36 hrs
3D, 3E	*tub-Gal80$^{[ts]}$ / UAS-Ehbp1 RNAi ; hh-Gal4, UAS-mCD8-gfp / UAS-wls RNAi*	18ºC for 10 ~ 12 days, 29 ºC for 3 days before dissection
4A, 4C, 4D	*FRT19A AP-1γ$^{[D]}$ / whi-mRFP(nls),hs-Flp, FRT19A*	25ºC except for heat shocking at 37ºC for 0.5 hour, recovering for 3 days before dissection
4B, 4E	*hs-Flp^{122} / + ;; FRT82B, ubi-gfp / FRT82B AP-1μ$^{[SHE11]}$*	25ºC except for heat shocking at 37ºC for 0.5 hour, recovering for 3 days before dissection
4F, 4H, 4I	*tub-Gal80$^{[ts]}$ / + ; hh-Gal4, UAS-mCD8-gfp / UAS-AP-1γRNAi*	18ºC for 10 ~ 12 days, 29 ºC for 34 hrs before dissection
4G, 4H, 4I	*tub-Gal80$^{[ts]}$ / UAS-Ehbp1 RNAi ; hh-Gal4, UAS-mCD8-gfp / UAS-AP-1γ RNAi*	18ºC for 10 ~ 12 days, 29 ºC for 34 hrs before dissection
4H, 4I, 5A	*hh-Gal4, UAS-mCD8-gfp / UAS-Ehbp1^{wt}*	25ºC
5E	*hs-Flp, tub-Gal4, UAS-gfp / + ; FRT42D,tub-Gal80 / FRT42D, Ehbp1^{A28} ; UAS-Ehbp1^{wt} / +*	25ºC except for heat shocking at 37ºC for 0.5 hour, recovering for 3 days before dissection
5F	*hs-Flp, tub-Gal4, UAS-gfp / + ; FRT42D,tub-Gal80 / FRT42D, Ehbp1^{A28} ; UAS-Ehbp1$^{\triangle bMERB}$ / +*	25ºC except for heat shocking at 37ºC for 0.5 hour, recovering for 3 days before dissection
5G	*hs-Flp, tub-Gal4, UAS-gfp / + ; FRT42D,tub-Gal80 / FRT42D, Ehbp1^{A28} ; UAS-Ehbp1$^{\triangle CC}$ / +*	25ºC except for heat shocking at 37ºC for 0.5 hour, recovering for 3 days before dissection

5H	*hs-Flp*^{122} */ + ; FRT42D, M2(58)f, ubi-gfp / FRT42D, Ehbp1*$^{\triangle CC}$	25ºC except for heat shocking at 37ºC for 0.5 hour, recovering for 3 days before dissection
EV1B	*wg-Gal4, UAS-gfp / +*	29ºC
EV1C	*wg-Gal4 / UAS-gzl.LD (II)*	29ºC
EV1D	*wg-Gal4 / UAS-gzl RNAi (BDSC64034)*	29ºC
EV1E	*wg-Gal4 / UAS-gzl RNAi (NIG10277R-3)*	29ºC
EV1F	*hh-Gal4, UAS-gfp / +*	23ºC
EV1G	*UAS-gzl.LD (II) /+, hh-Gal4 / +*	23ºC
EV1H	*UAS-gzl RNAi (BDSC64034) , hh-Gal4 / +*	23ºC
EV1I	*UAS-gzl RNAi (NIG10277R-3) , hh-Gal4 / +*	23ºC
EV2C	*tub:gfp:3'UTR*^{Ehbp1} */ + ; hh-Gal4, UAS-myr-mrfp / UAS-mir-bft*	25ºC
EV2D	*tub:gfp:3'UTRmut*^{Ehbp1} *-gfp / + ; hh-Gal4, UAS-myr-mrfp / UAS-mir-bft*	25ºC
EV4A, EV4C, EV4D, EV4E, EV4F	*hh-Gal4, UAS-mCD8-gfp / UAS-sec6 RNAi*	25ºC
EV4B, EV4C, EV4D, EV4E, EV4F	*tub-Gal80*$^{[ts]}$ */ UAS-gzl.LD ; hh-Gal4, UAS-mCD8-gfp / +*	18ºC for 10 ~ 12 days, 29 ºC for 24 hrs
EV4C, EV4D, EV4E, EV4F	*hh-Gal4, UAS-mCD8-gfp / UAS-sec5 RNAi*	25ºC
S3A	*hs-Flp, tub-Gal4, UAS-gfp / + ; FRT42D,tub-Gal80 / FRT42D, Ehbp1*^{A28} *; UAS-Ehbp1*$^{\triangle Nt-C2}$ */ +*	25ºC except for heat shocking at 37ºC for 0.5 hour, recovering for 3 days before dissection
S3B	*hs-Flp, tub-Gal4, UAS-gfp / + ; FRT42D,tub-Gal80 / FRT42D, Ehbp1*^{A28} *; UAS-Ehbp1*$^{\triangle CH}$ */ +*	25ºC except for heat shocking at 37ºC for 0.5 hour, recovering for 3 days before dissection
EV5A	*UAS-yfp-Rab8CA. [Q67L] / + ; hh-Gal4 / +*	25ºC
EV5B	*UAS-yfp-Rab8CA. [Q67L] / UAS-Ehbp1 RNAi ; hh-Gal4 / +*	25ºC
EV5C	*UAS-yfp-Rab8DN.[T22N] / hh-Gal4*	25ºC
EV5D	*UAS-Ehbp1 RNAi / + ; UAS-yfp-Rab8DN.[T22N] / hh-Gal4*	25ºC
EV5E	*UAS-yfp-Rab10CA.[Q68L] / hh-Gal4*	25ºC
EV5F	*UAS-Ehbp1 RNAi / + ; UAS-yfp-Rab10CA.[Q68L] / hh-Gal4*	25ºC
EV5G	*UAS-yfp-Rab10DN.[T23N] / hh-Gal4*	25ºC
EV5H	*UAS-Ehbp1 RNAi / + ; UAS-yfp-Rab10DN.[T23N] / hh-Gal4*	25ºC
S1A, S1B	*Wg:GFP/UAS-Ehbp1 RNAi ; hh-Gal4, UAS-myr-rfp/+*	18ºC

Appendix Table S2. Primers used in this study.

**Primers for generating *pUAST-dEhbp1***	**Sequence**
Ehbp1-NotI-F	5'- AAATATGCGGCCGCATGGCCAGCGTATGGAAGCG -3'
Ehbp1-NotI-R	5'- AAATATGCGGCCGCGCTTGAAGAACACATTTGTCTTTTTCAGC -3'
Ehbp1-NtC2-Del-5R	5'- CGTCTGTAGCGAAATTGCGCTGCAATCGCTTC -3'
Ehbp1-NtC2-Del-3F	5'- GCGCAATTTCGCTACAGACGAGGACATGCAGA -3'
Ehbp1-CH-Del-5R	5'- CGGTAAAGTGCTCCTTCAGCACGATCTTCTCCA -3'
Ehbp1-CH-Del-3F	5'- GCTGAAGGAGCACTTTACCGGCAAGCAGCTAAA -3'
Ehbp1-CC-Del-5R	5'- CGCCCATTACCTCGGAATCGATGTAGGACAGCT -3'
Ehbp1-CC-Del-3F	5'- CGATTCCGAGGTAATGGGCGGCAATCCAAAGA -3'
Ehbp1-BM-Del-5R	5'- CTATTGAAGAACACATTTGTCTTTTTCAGCACTGATGTCC ACGTGCTCCAGGACGCGCTCCACGATTGC -3'
Ehbp1-BM-Del-3F	5'- ATCGTGGAGCGCGTCCTGGAGCACGTGGACATC -3'
**siRNA sequence**	**Sequence**
siEHBP1-F	5'- GCAUCAACAUGAAACAGUAUG -3'
siEHBP1-R	5'- UACUGUUUCAUGUUGAUGCUG -3'
siWLS-F	5'- GAAGCGAUUCCAAGAGAAAUC -3'
siWLS-R	5'- UUUCUCUUGGAAUCGCUUCCU -3'
siAP1μ1A-F	5'- GUGCUCAUCUGCCGGAAUUAC -3'
siAP1μ1A-R	5'- AAUUCCGGCAGAUGAGCACCU -3'
NC-F	5'- UUCUCCGAACGUGUCACGUTT -3'
NC-R	5'- ACGUGACACGUUCGGAGAATT -3'
**Primers for generating *Ehbp1 sensor - wt / mutation***	**Sequence**
Ehbp1SensorF	5'- GCTCTAGAGGATCGAAGACGACCAGGAGAT -3'
Ehbp1SensorR	5'- CGCCTCGAGTTGGGTTTATTCGATGCTGATGACA -3'
Ehbp1-Mut-F	5'- GTTTCGTTGTTGTGGCTTCTGAACCACTCCTAACTTATG -3'
Ehbp1-Mut-R	5'- AGTGGTTCAGAAGCCACAACAACGAAACATGAAACAAAGT -3'
Ehbp1$^{\Delta CC}$ gRNA targets	**Sequence**
gRNA1	5'- TCTCACCAAAGCACGAGAAG -3'
gRNA2	5'- GCTTCACTTACGCTATCTCT -3'
**Primers for *pCFD4-Ehbp1$^{\Delta CC}$ -gRNAs***	**Sequence**
gCC1-F	5'- TATATAGGAAAGATATCCGGGTGAACTTCGTCTCACCAAAGCAC GAGAAGGTTTTAGAGCTAGAAATAGCAAG -3'
gCC2-R	5'- ATTTTAACTTGCTATTTCTAGCTCTAAAACAGAGATAGCGTAAGTG AAGCCGACGTTAAATTGAAAATAGGTC -3'
**Primers for *pGEMT-Ehbp1$^{\Delta CC}$ template***	**Sequence**
Up-CC1-F	5'- CCCATATGGTCGACCTGCAGCCGGCAAGCAGCTAAAAATTGAGC -3'
Up-CC1-R	5'- CGATTATTACTTGGATTGCCGCCCATTACCT CGGAATCGATGTAGGACAGCTTGT -3'

CC1-CC2-F	5'- ACAAGCTGTCCTACATCGATTCCGAGGTAAT GGGCGGCAATCCAAGTAATAATCG -3'
CC1-CC2-R	5'- GAACTTCCGTTTTGCGCCAATCTTCTTGCTCG CTGAAGGGTGTAGATAATAATAAGAAAC -3'
Down-CC2-F	5'- GTTTCTTATTATTATCTACACCCTTCAG CGAGCAAGAAGATTGGCGCAAAACGGAAGTTC -3'
Down-CC2-R	5'- AATTGGGCCCGACGTCGCATACTAGAGCAATGGTAGCGCCGTC -3'
**Primers for Genotype identification**	**Sequence**
CC1-F	5'- TTATCTATCACTCCACTCACTGGGG -3'
CC1-R	5'- CCACCGACAGACAATGTTGCTATG -3'
CC2-F	5'- CACCCAAGGCTCTGCTCCCACAAT -3'
CC2-R	5'- CAATCATATCGCTGTCTCACTC -3'
**Primers for generating *pCaspeR-hs-wls-HA***	**Sequence**
wls-HA-F	5'- ATTCGTTAACAGATCT GCGGCCGC ATGTCGGGCACCATACTGGAGAA -3'
wls-HA-R	5'- CAGCTGGAATTAGGCCT TCTAGA TTA CTTGTCATCGTCGTCCTTGTAGTC ATCGAAGGCCACCTTGCGT -3'
**Primers for generating *pActin5.1-AP-1γ-flag***	**Sequence**
AP1-G-F	5'- ACGATGACAAGCTTGGCGGCCGCATGAACTCCGAGCATGGATTTAATCC -3'
AP1-G-R	5'- GAAGGGCCCTCTAGACTCGAGCCTTAGTGTTGCTGCTGTTCTTGT -3'
**Primers for generating *pSIN-WNT1-gfp***	**Sequence**
WNT1-Sin-F	5'- AGGAATTGATCCTTCGAAACTAGTCAGGCCATGGGGCTCTGGGCGCTGTTGC -3'
WNT1-Sin-R	5'- CTTGCTCACCATGCTAGCCATATGCAGACACTCGTGCAGTACGCGC -3'
**Primers for MDCK Wnts RT-PCR**	**Sequence**
Wnt1-F	5'- ctcttcggcaagatcgtcaacc -3'
Wnt1-R	5'- gaagtcgatgttgtcgctgcag -3'
Wnt2-F	5'- gggatcacagcctctttggc -3'
Wnt2-R	5'- atgttatcgctgcagccacc -3'
Wnt3-F	5'- gaacaagcacaacaacgaggc -3'
Wnt3-R	5'- tgaacagcgcatacttggcg -3'
Wnt3a-F	5'- ggaattcgctgacgcacgg -3'
Wnt3a-R	5'- aggcgctgtcgtacttgtcc -3'
Wnt5a-F	5'- ccaagggctcgtacgagagc -3'
Wnt5a-R	5'- ctgcgctgtcgtacttctcc -3'
Wnt5b-F	5'- ggctaccgctttgctaaggag -3'
Wnt5b-R	5'- ggagccagcaggtcttgagg -3'
Wnt7a-F	5'- gaagcaaggccagtaccacc -3'
Wnt7a-R	5'- cggcctcgttgtacttgtcc -3'
Wnt7b-F	5'- gcagggctactacaaccagg -3'

Wnt7b-R	5'- ctccatgggcttctggtagc -3'
Wnt8a-F	5'- cgcccagagtggcatagagg -3'
Wnt8a-R	5'- ccccagatccagccatgacc -3'
Wnt8b-F	5'- acctgtatcctccgacttggc -3'
Wnt8b-R	5'- ccaaggctgcagttcctagtc -3'
Wnt9a-F	5'- ccgaaggtcgagcaaggacc -3'
Wnt9a-R	5'- tggtgctgcccaccttgagc -3'
Wnt9b-F	5'- gcacctgtgatgactctccg -3'
Wnt9b-R	5'- gagctgcttccagcaggtg -3'
Wnt11-F	5'- cccaagccaataaactgatgcg -3'
Wnt11-R	5'- acaggtagcgggtcttgaggtc -3'
