# Supplementary figures and images for: Ehbp1 orchestrates orderly sorting of Wnt/Wingless to the basolateral and apical cell membranes (part 1 of 3)
Source: EMBO Rep. 2024 Oct 14;25(11):5053–79. doi: 10.1038/s44319-024-00289-1 (PMC11549480; doi:10.1038/s44319-024-00289-1)

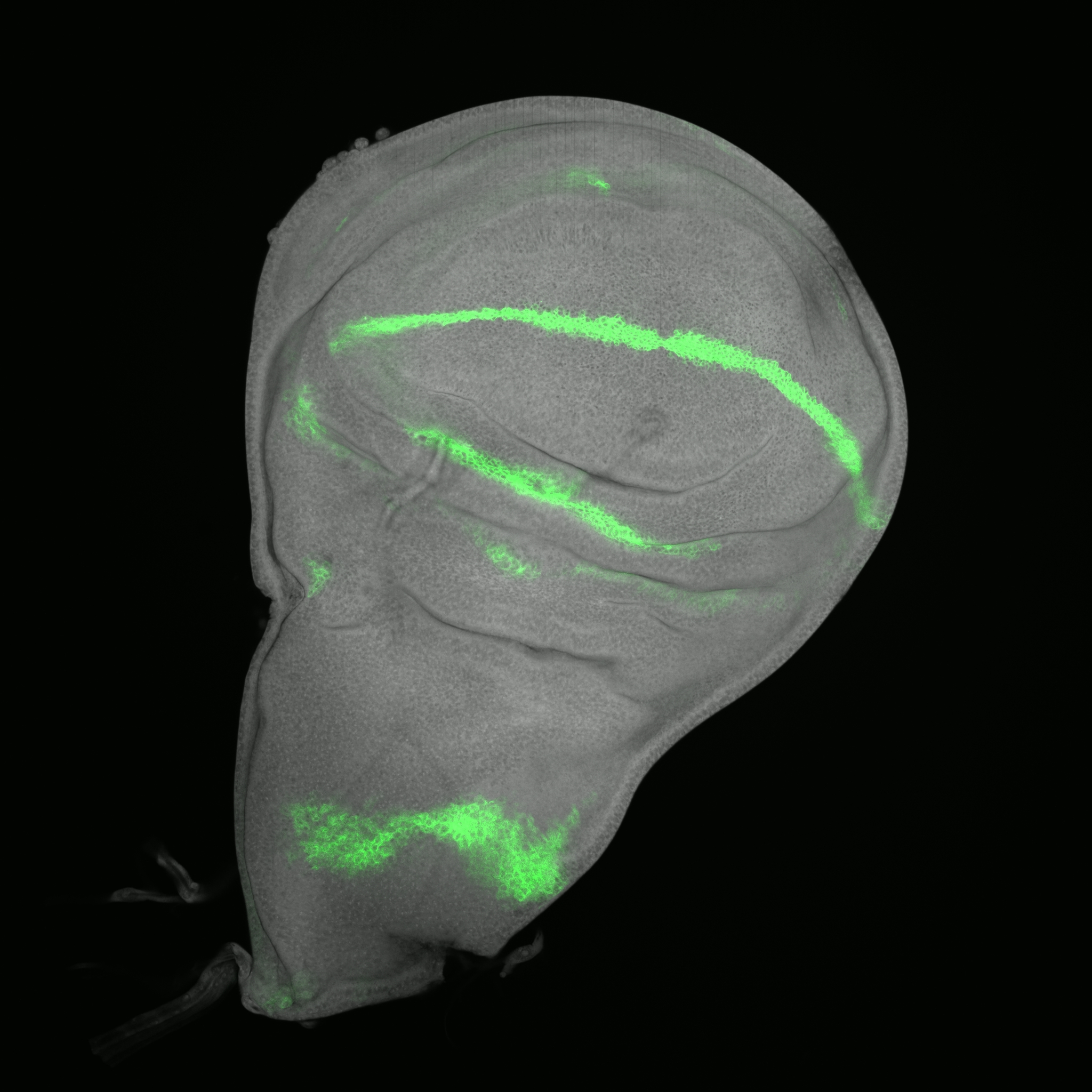

Supplement: Supplementary file 3 — Source data Fig. 1 [file 44319_2024_289_MOESM3_ESM.zip › Figure 1/F1C/F1C 20210928 Wg-gal4 mcd8-GFP.tif]

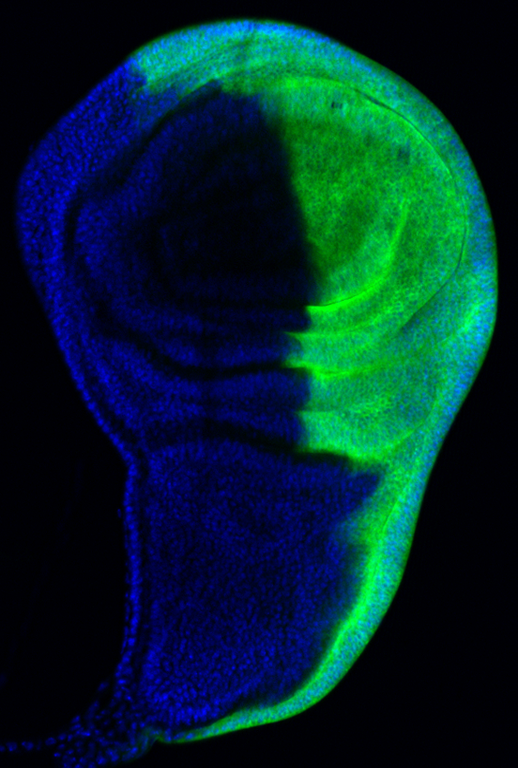

Supplement: Supplementary file 3 — Source data Fig. 1 [file 44319_2024_289_MOESM3_ESM.zip › Figure 1/F1C/F1C hh-G4 GFP.tif]

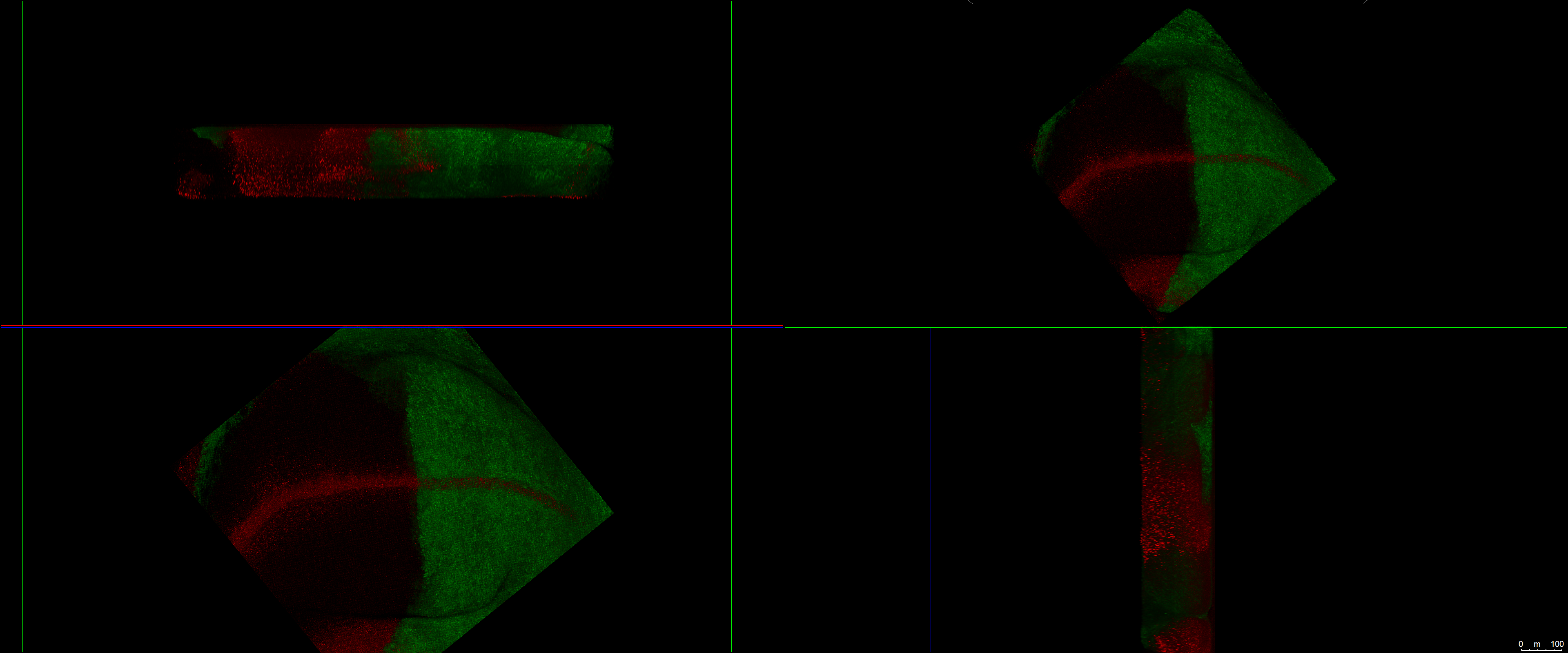

Supplement: Supplementary file 3 — Source data Fig. 1 [file 44319_2024_289_MOESM3_ESM.zip › Figure 1/F1C/F1C hh-G4-GFP GFP wg wls_3D 1.tif]

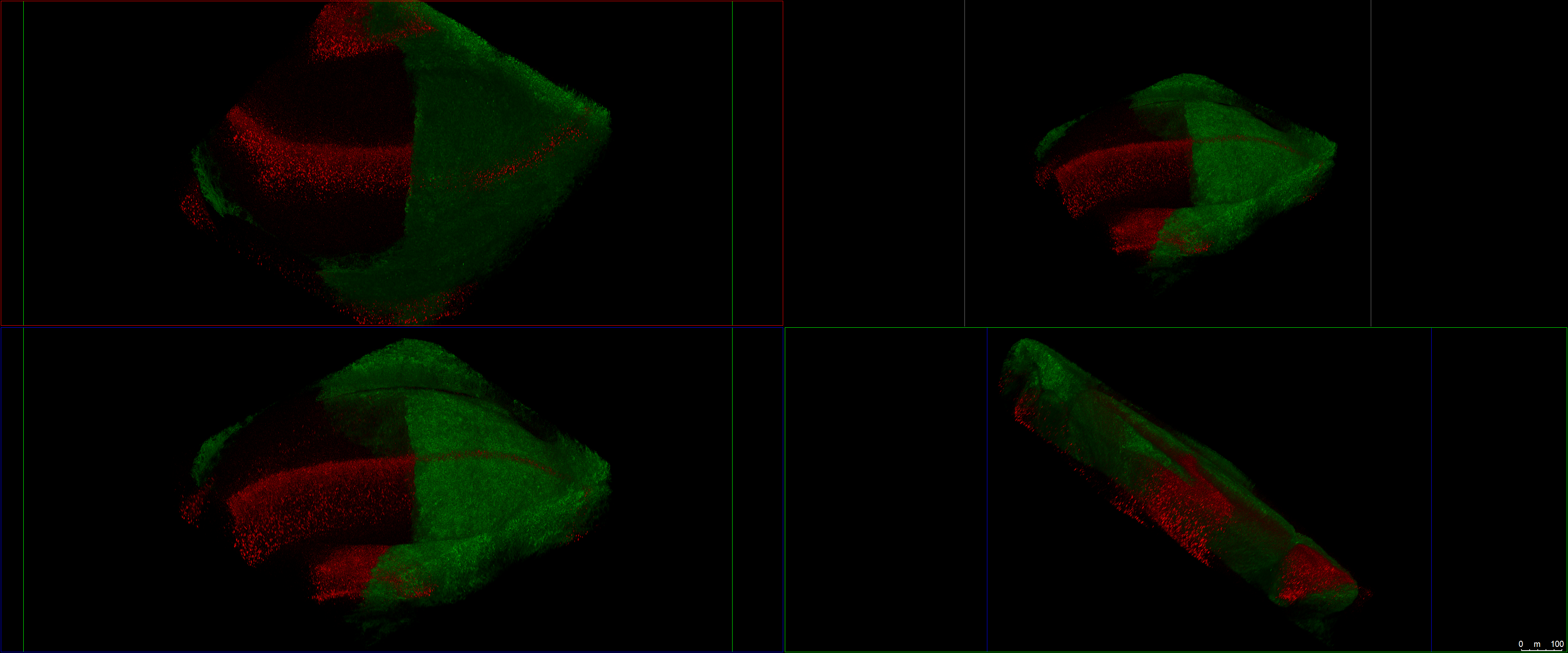

Supplement: Supplementary file 3 — Source data Fig. 1 [file 44319_2024_289_MOESM3_ESM.zip › Figure 1/F1C/F1C hh-G4-GFP GFP wg wls_3D 2.tif]

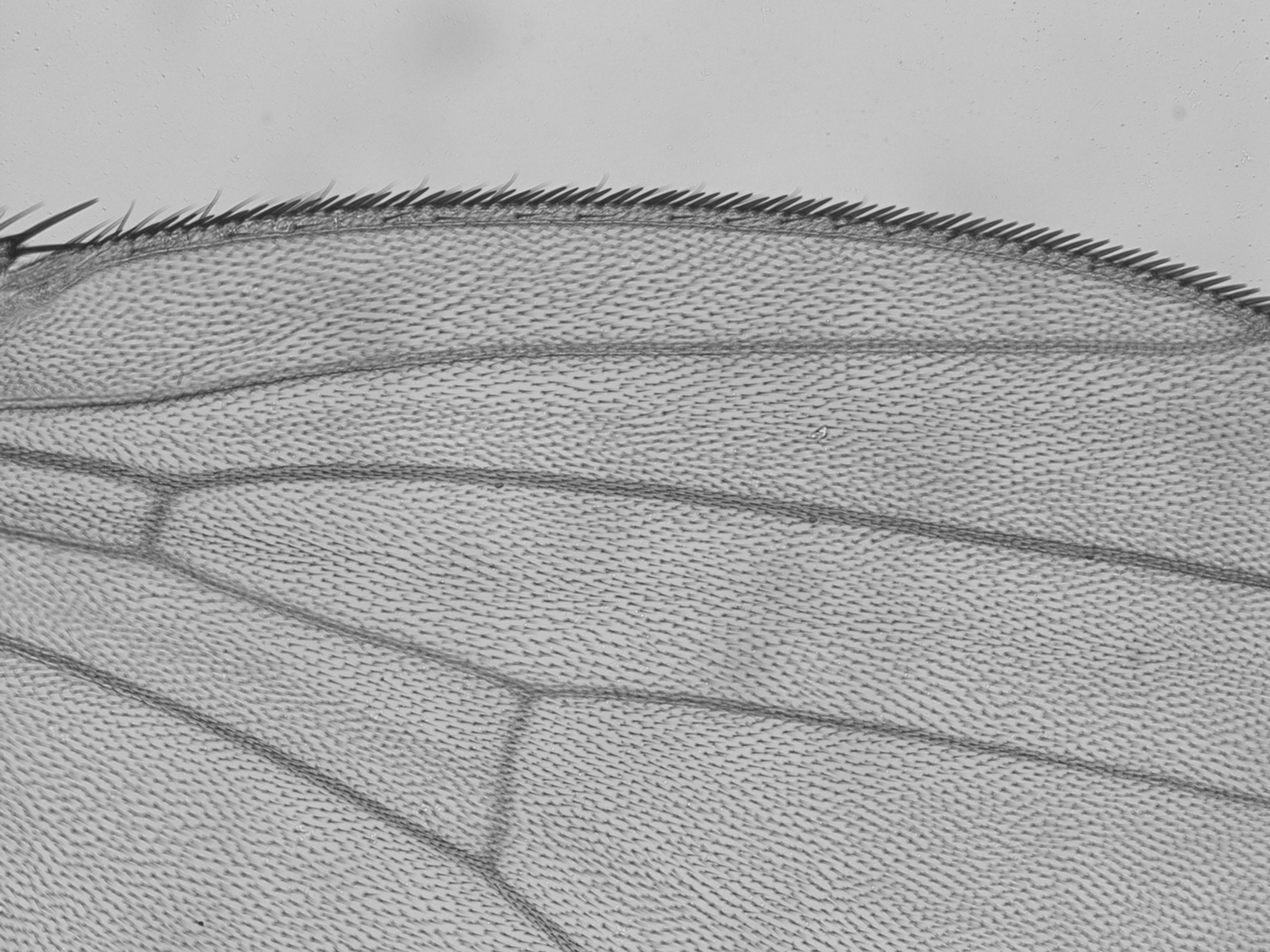

Supplement: Supplementary file 3 — Source data Fig. 1 [file 44319_2024_289_MOESM3_ESM.zip › Figure 1/F1D/F1D 160129 Wg-G4 GFP -1 -2.tif]

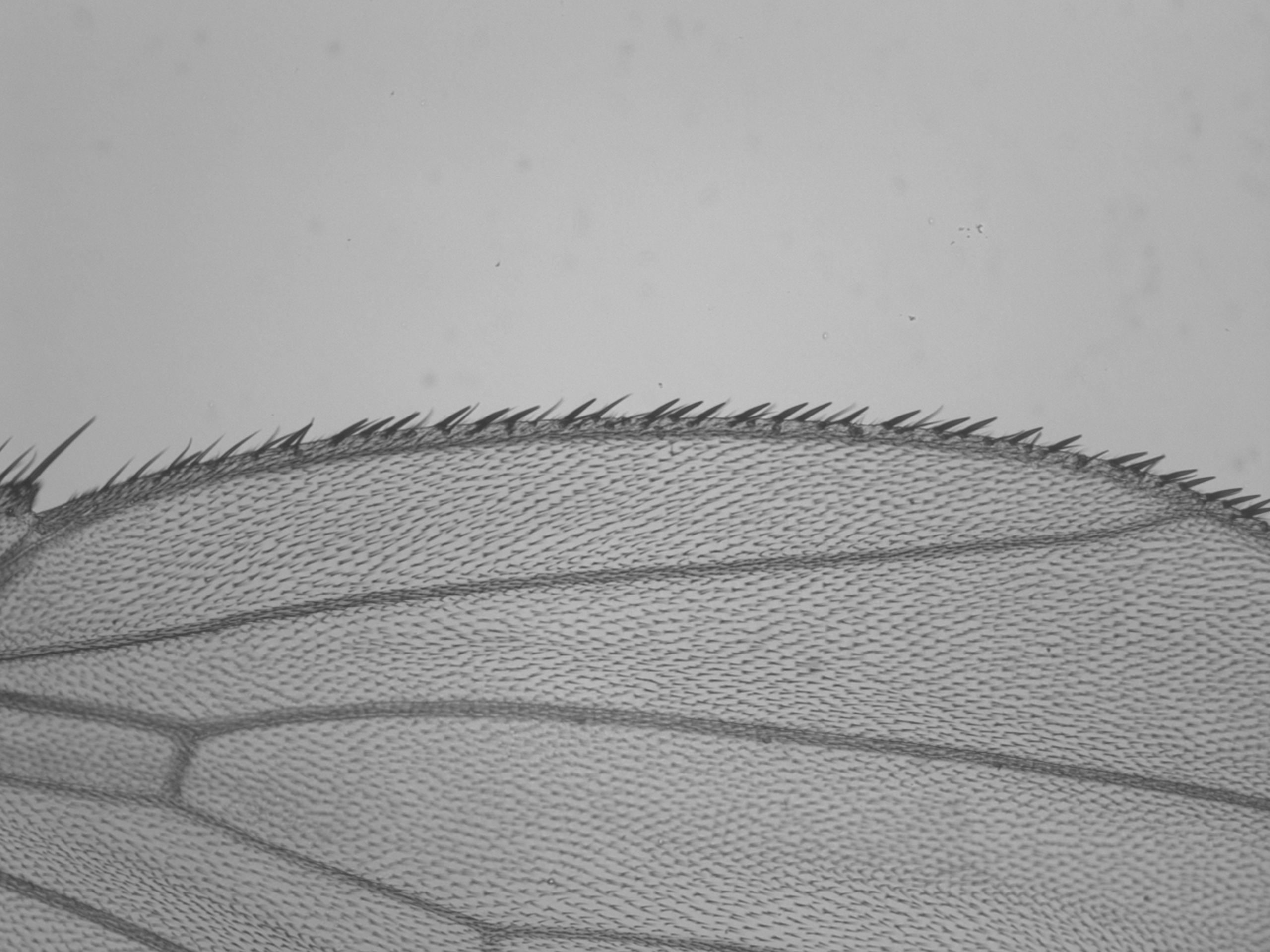

Supplement: Supplementary file 3 — Source data Fig. 1 [file 44319_2024_289_MOESM3_ESM.zip › Figure 1/F1F/F1F 220316 18C wg-G4 TH02340(Ehbp1 RNAi) -2.tif]

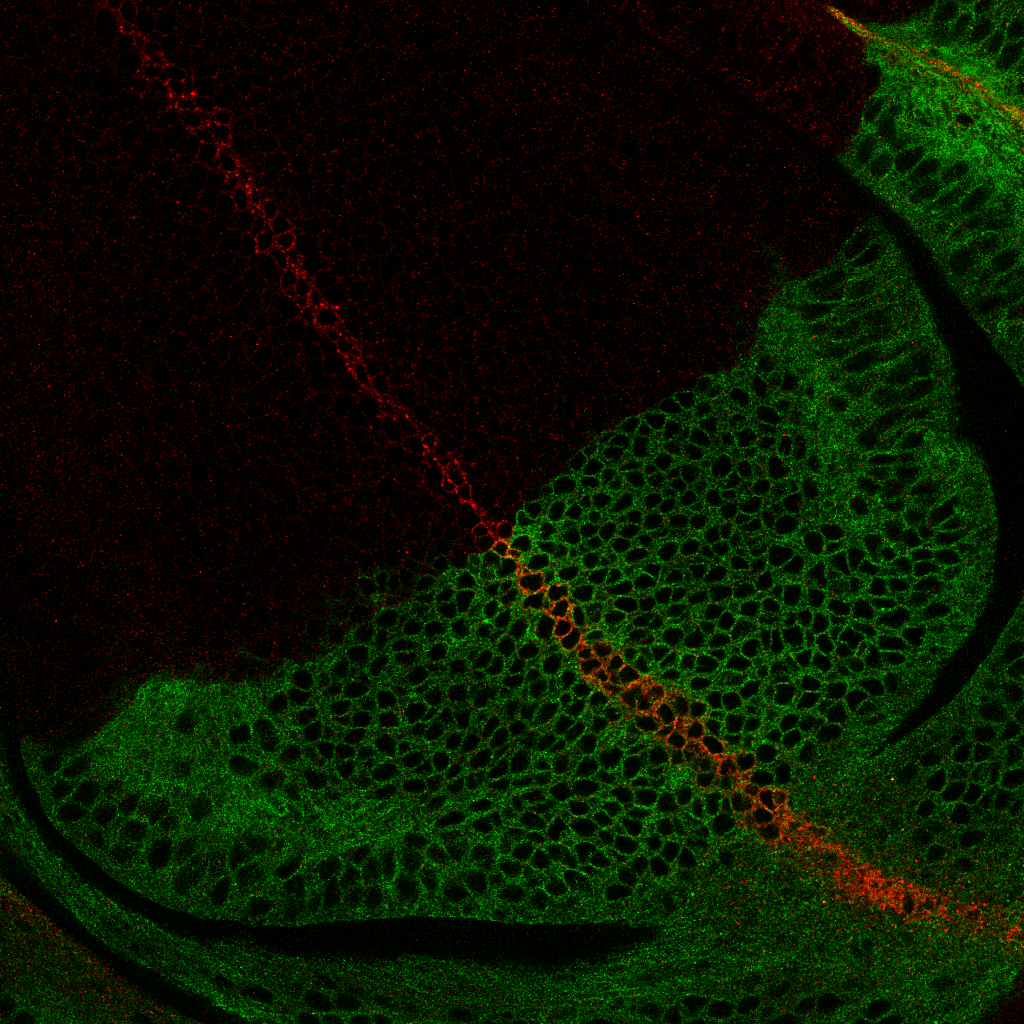

Supplement: Supplementary file 3 — Source data Fig. 1 [file 44319_2024_289_MOESM3_ESM.zip › Figure 1/F1G/F1G and F1G' ts-Gal80 hh-G4-GFP BL41133 36h wg_Series001_Lng_global_z055.tif]

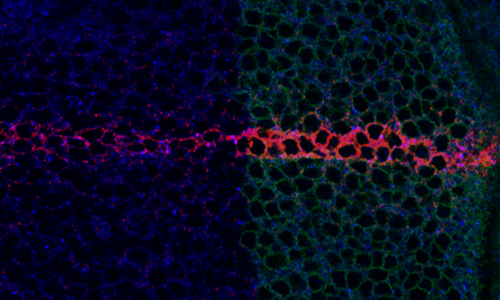

Supplement: Supplementary file 3 — Source data Fig. 1 [file 44319_2024_289_MOESM3_ESM.zip › Figure 1/F1G/F1G'' Images for statictical analysis/1 ts-Gal80 hh-G4-GFP BL41133 34h wg wls S1-z49.tif]

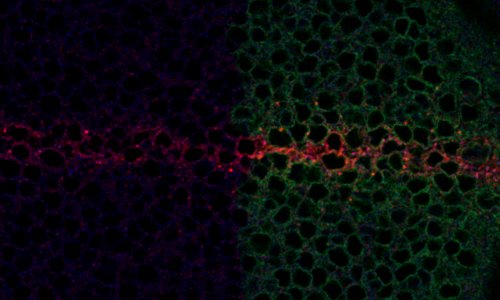

Supplement: Supplementary file 3 — Source data Fig. 1 [file 44319_2024_289_MOESM3_ESM.zip › Figure 1/F1G/F1G'' Images for statictical analysis/2 ts-Gal80 hh-G4-GFP BL41133 wg wls S3-z43.tif]

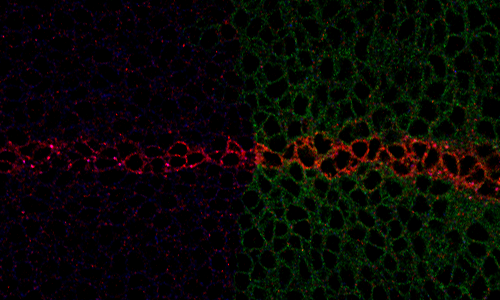

Supplement: Supplementary file 3 — Source data Fig. 1 [file 44319_2024_289_MOESM3_ESM.zip › Figure 1/F1G/F1G'' Images for statictical analysis/3 ts-Gal80 hh-G4-GFP BL41133 36h wg wls S1z50.tif]

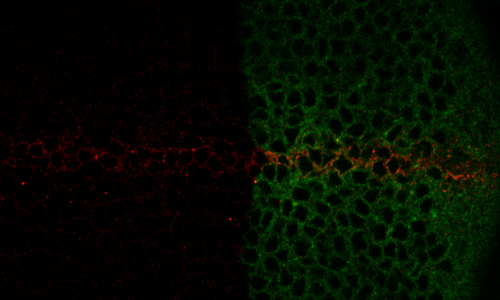

Supplement: Supplementary file 3 — Source data Fig. 1 [file 44319_2024_289_MOESM3_ESM.zip › Figure 1/F1G/F1G'' Images for statictical analysis/4 ts-Gal80 hh-G4-GFP BL41133 wg S2z69.tif]

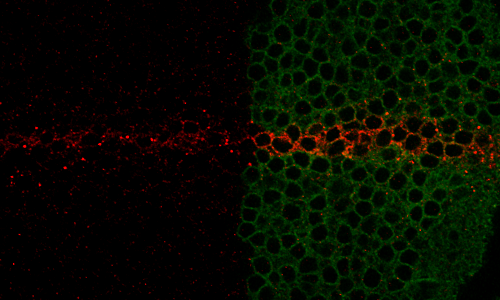

Supplement: Supplementary file 3 — Source data Fig. 1 [file 44319_2024_289_MOESM3_ESM.zip › Figure 1/F1G/F1G'' Images for statictical analysis/5 ts-Gal80 hh-G4-GFP BL41133 wg S1z65.tif]

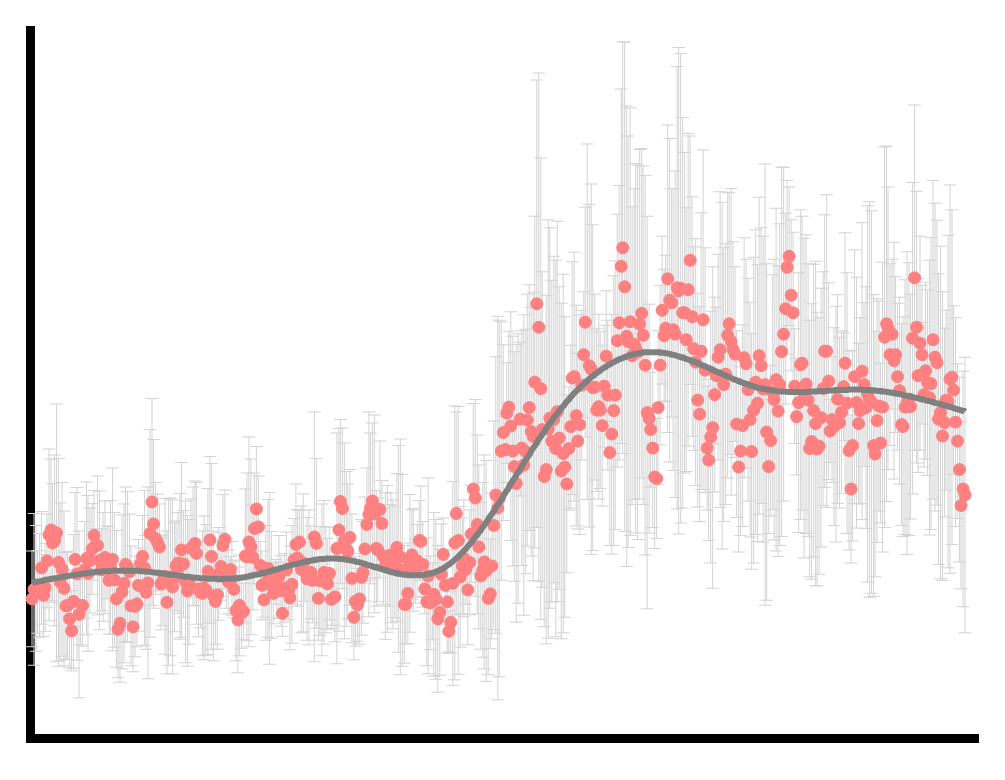

Supplement: Supplementary file 3 — Source data Fig. 1 [file 44319_2024_289_MOESM3_ESM.zip › Figure 1/F1G/F1G'' statistical analysis-mir-bft oe-Wg.tif]

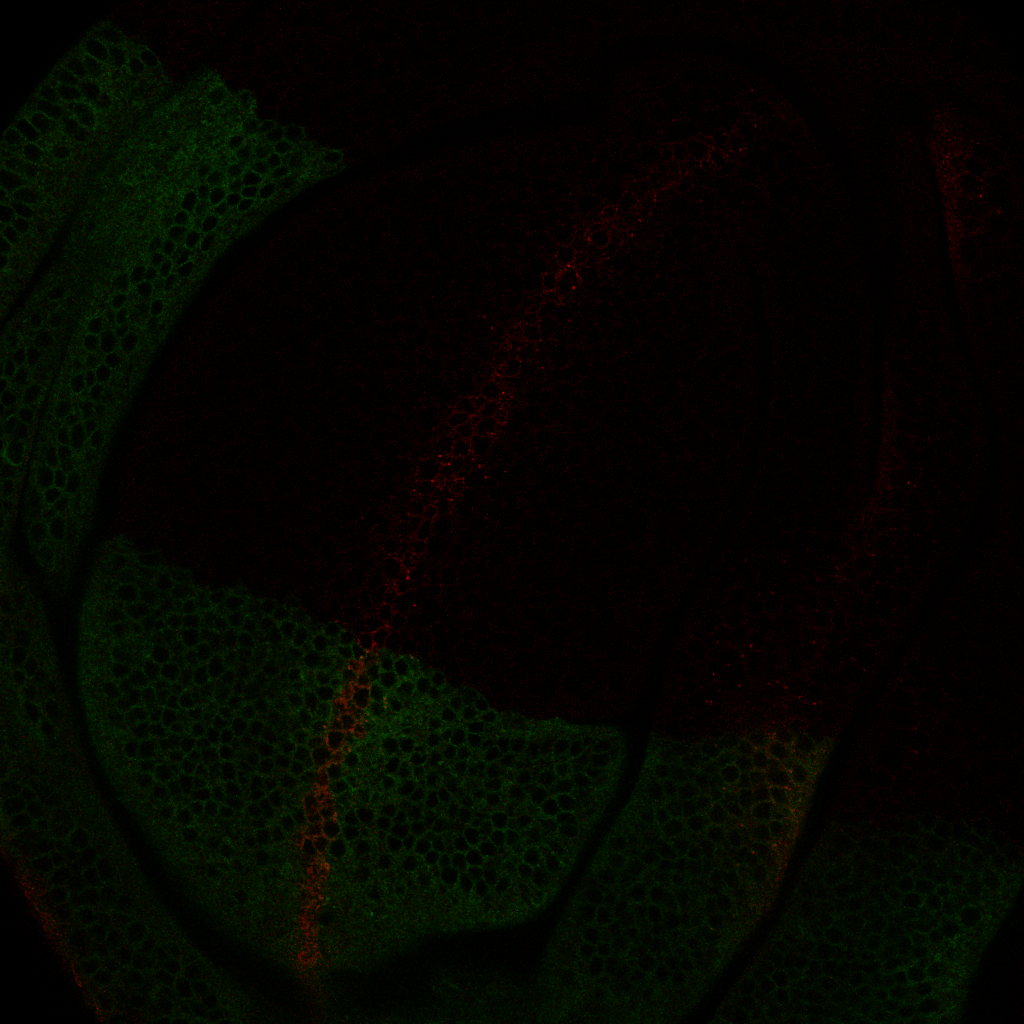

Supplement: Supplementary file 3 — Source data Fig. 1 [file 44319_2024_289_MOESM3_ESM.zip › Figure 1/F1H/F1H1 ts-Gal80 hh-G4-GFP TH02340 wg.tif]

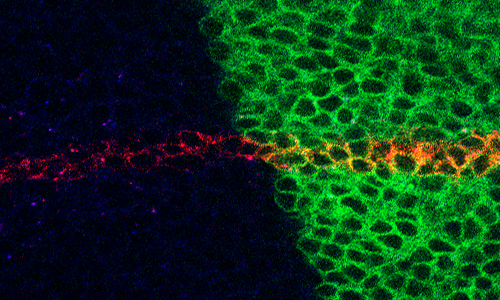

Supplement: Supplementary file 3 — Source data Fig. 1 [file 44319_2024_289_MOESM3_ESM.zip › Figure 1/F1H/F1H1'' Images for statistical analysis/181224 hh-G4-GFP TH02340 wg Dl_Series006_z40.tif]

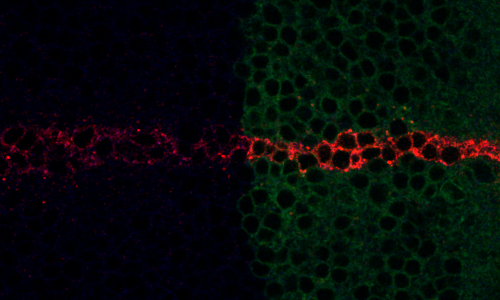

Supplement: Supplementary file 3 — Source data Fig. 1 [file 44319_2024_289_MOESM3_ESM.zip › Figure 1/F1H/F1H1'' Images for statistical analysis/191231 ts-Gal80 hh-G4-GFP TH02340 48h wg wls_Series001_Lng_000_z048.tif]

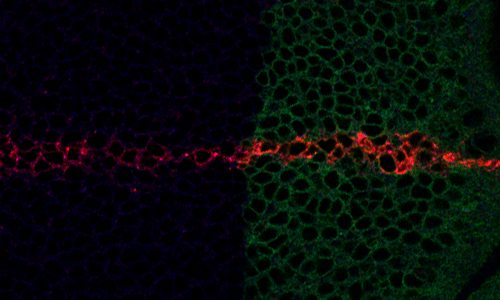

Supplement: Supplementary file 3 — Source data Fig. 1 [file 44319_2024_289_MOESM3_ESM.zip › Figure 1/F1H/F1H1'' Images for statistical analysis/191231 ts-Gal80 hh-G4-GFP TH02340 48h wg wls_Series002_Lng_z051.tif]

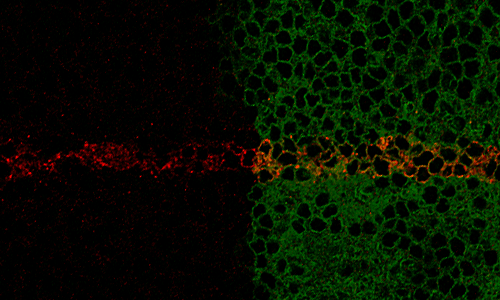

Supplement: Supplementary file 3 — Source data Fig. 1 [file 44319_2024_289_MOESM3_ESM.zip › Figure 1/F1H/F1H1'' Images for statistical analysis/200516 ts-Gal80 hh-G4-GFP TH02340 wg 2_Series002_Lng_global_z039.tif]

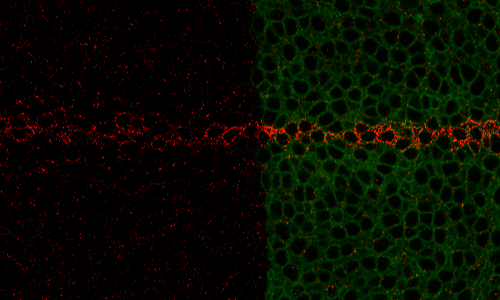

Supplement: Supplementary file 3 — Source data Fig. 1 [file 44319_2024_289_MOESM3_ESM.zip › Figure 1/F1H/F1H1'' Images for statistical analysis/200814 ts-Gal80 hh-G4-GFP TH02340 wg_Series006_Lng_adaptive_z068.tif]

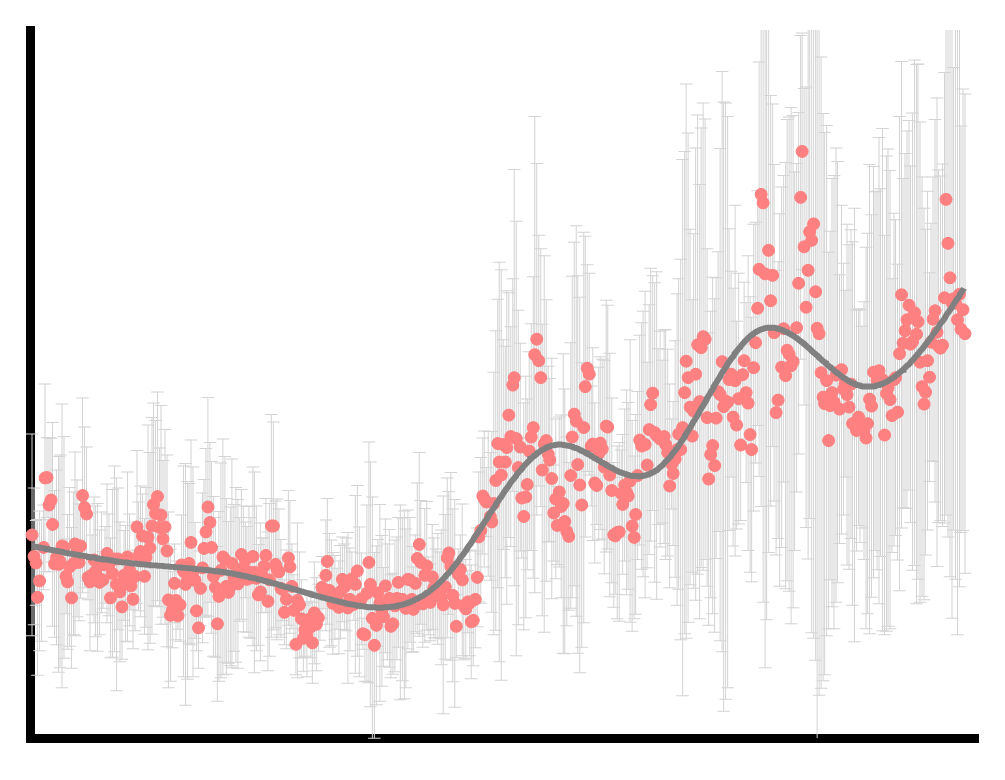

Supplement: Supplementary file 3 — Source data Fig. 1 [file 44319_2024_289_MOESM3_ESM.zip › Figure 1/F1H/F1H1'' Statistical analysis - Ehbp1 RNAi - Wg.tif]

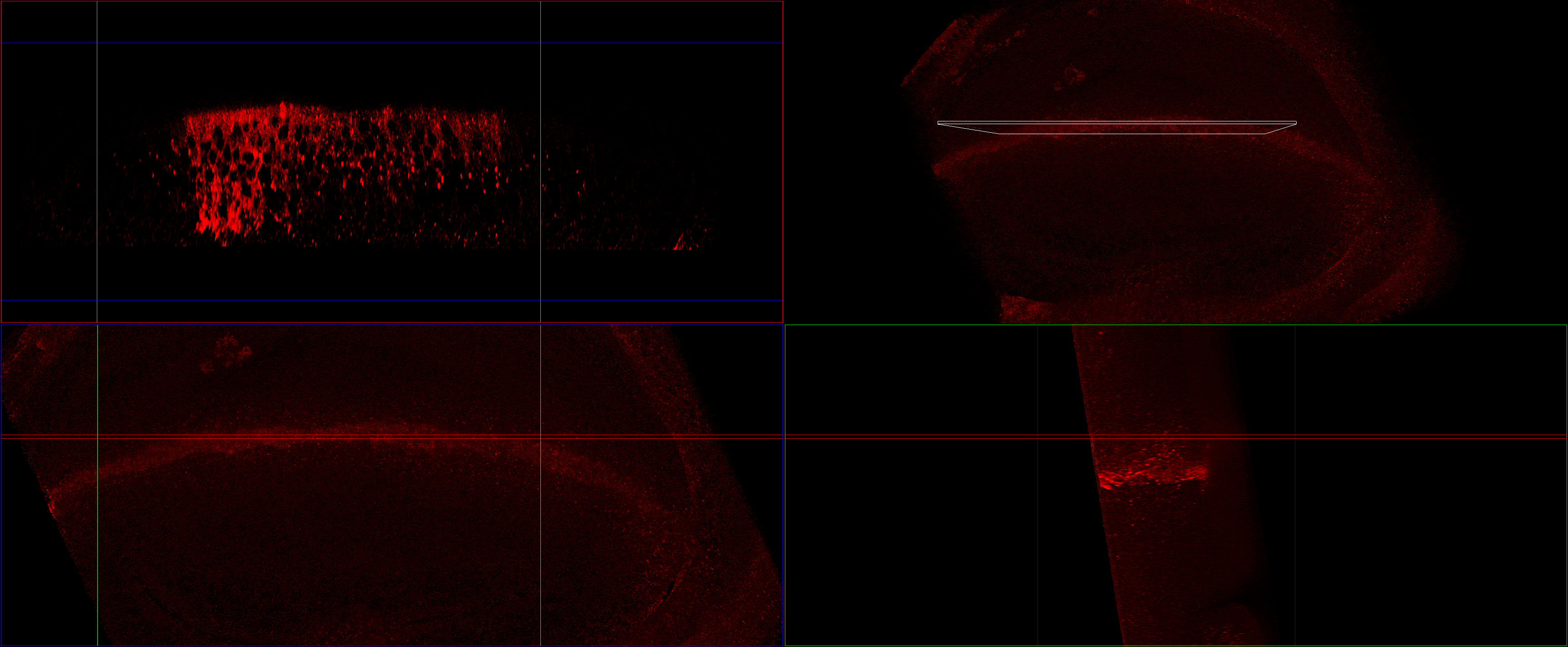

Supplement: Supplementary file 3 — Source data Fig. 1 [file 44319_2024_289_MOESM3_ESM.zip › Figure 1/F1H/F1H2 ts-Gal80 hh-G4-GFP TH02340 wg_3D 2.tif]

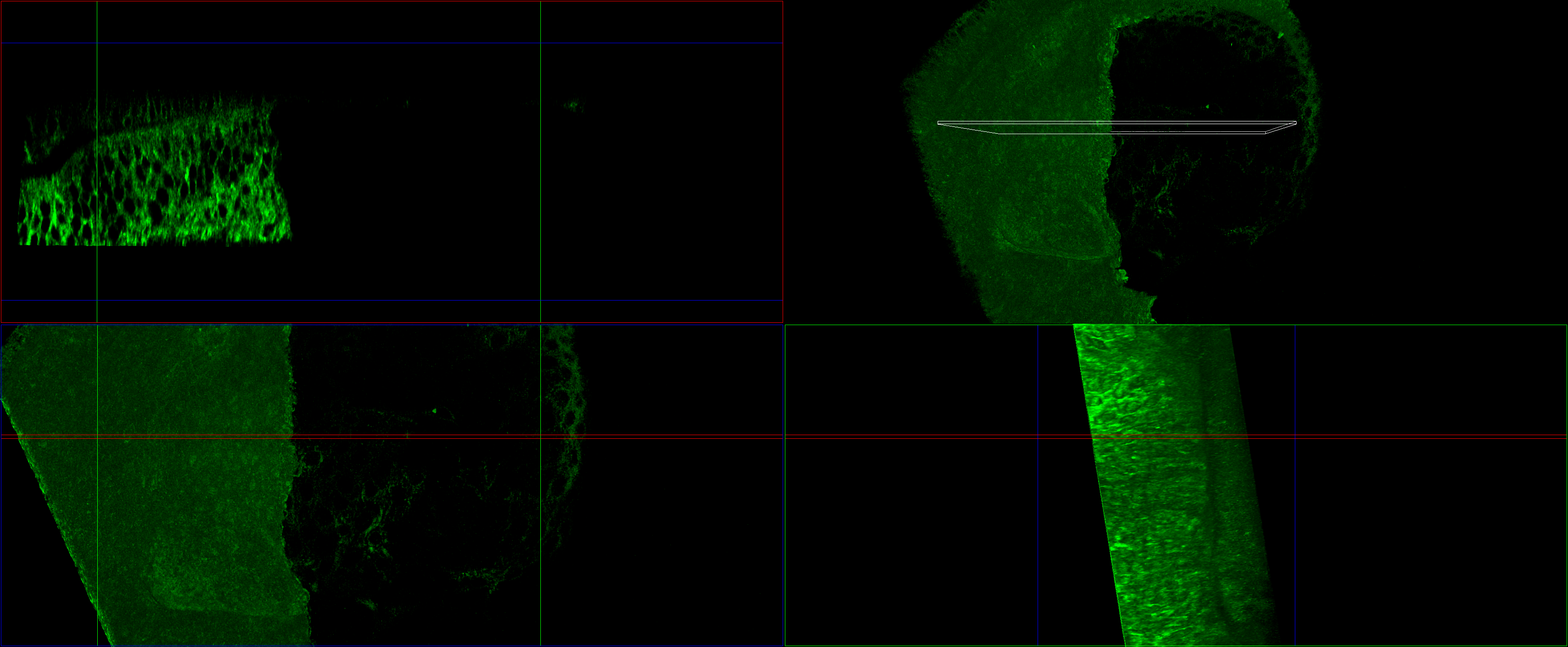

Supplement: Supplementary file 3 — Source data Fig. 1 [file 44319_2024_289_MOESM3_ESM.zip › Figure 1/F1H/F1H2 ts-Gal80 hh-G4-GFP TH02340 wg_3D.tif]

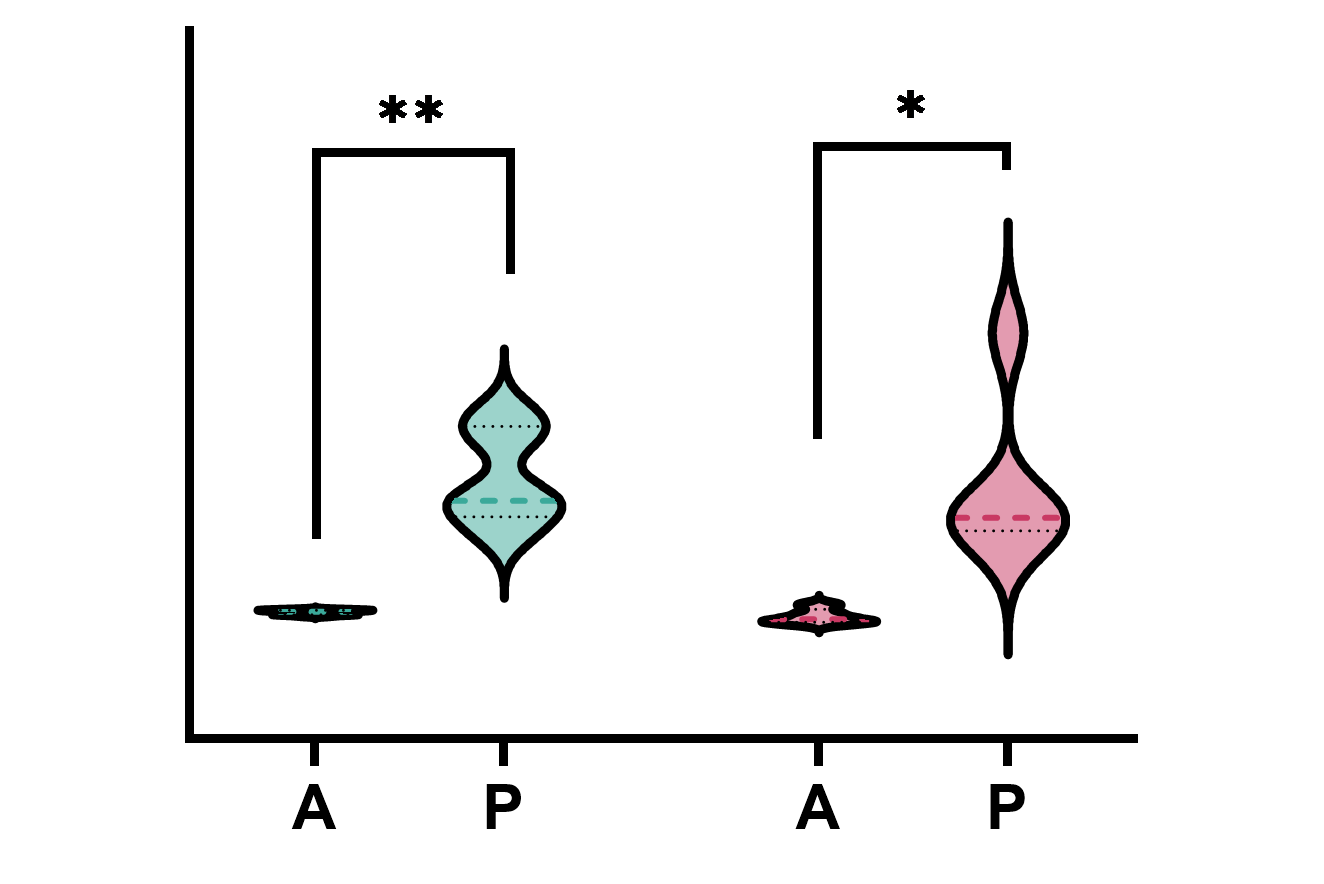

Supplement: Supplementary file 3 — Source data Fig. 1 [file 44319_2024_289_MOESM3_ESM.zip › Figure 1/F1I/F1I - mir-bft OE and Ehbp1 RNAi- Wg .tif]

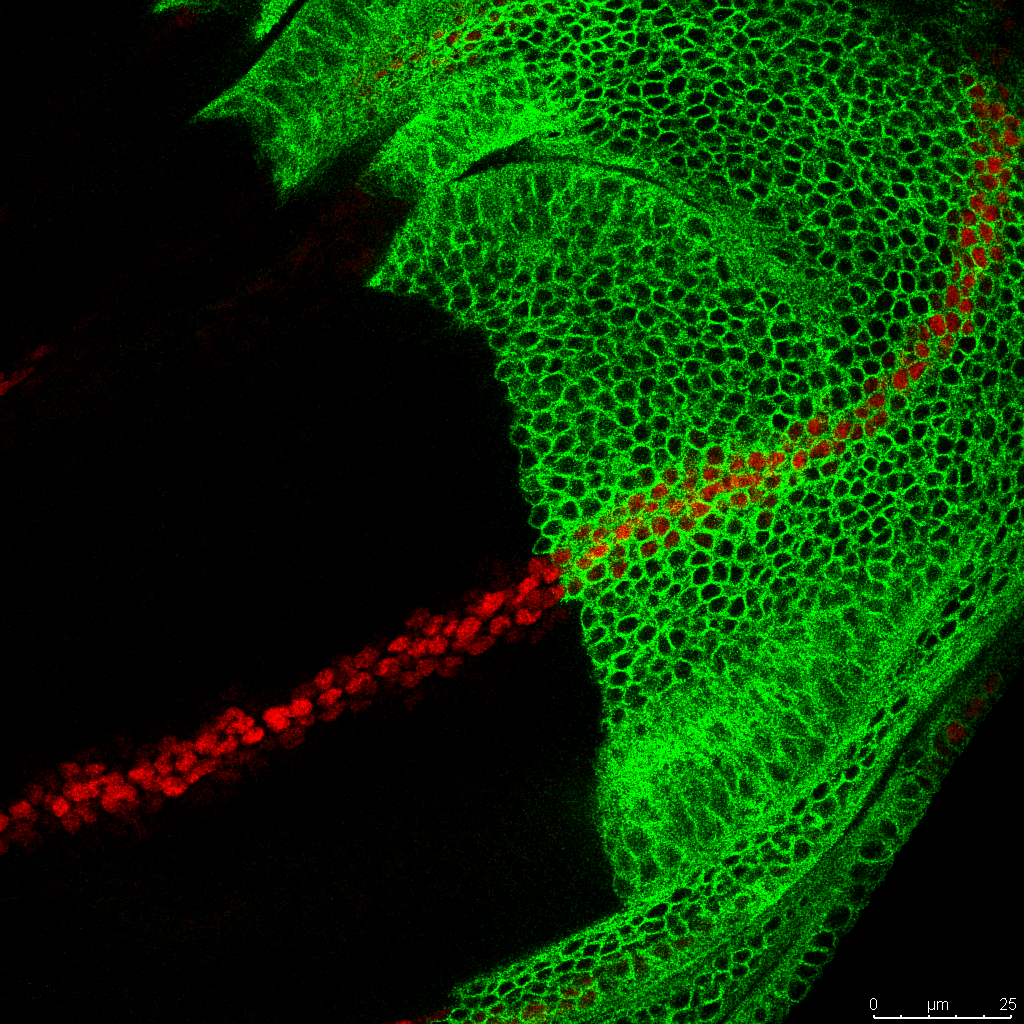

Supplement: Supplementary file 3 — Source data Fig. 1 [file 44319_2024_289_MOESM3_ESM.zip › Figure 1/F1J/F1J hh-G4-GFP TH02340 Wg-lacZ_Series001_Lng_z15.tif]

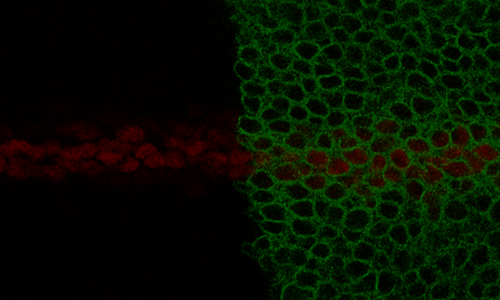

Supplement: Supplementary file 3 — Source data Fig. 1 [file 44319_2024_289_MOESM3_ESM.zip › Figure 1/F1J/F1J images for statistical analysis/1 hh-G4-GFP TH02340 Wg-lacZ_Series001_Lng_z12.tif]

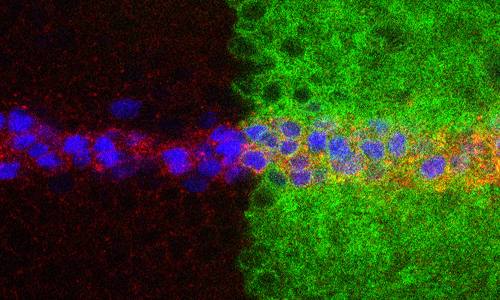

Supplement: Supplementary file 3 — Source data Fig. 1 [file 44319_2024_289_MOESM3_ESM.zip › Figure 1/F1J/F1J images for statistical analysis/2 hh-G4-GFP TH02340 Wg-lacZ2 Wg lacZ_Series004_z07.tif]

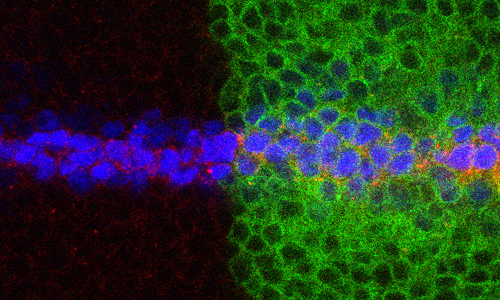

Supplement: Supplementary file 3 — Source data Fig. 1 [file 44319_2024_289_MOESM3_ESM.zip › Figure 1/F1J/F1J images for statistical analysis/3 hh-G4-GFP TH02340 Wg-lacZ2 Wg lacZ_Series007_z10.tif]

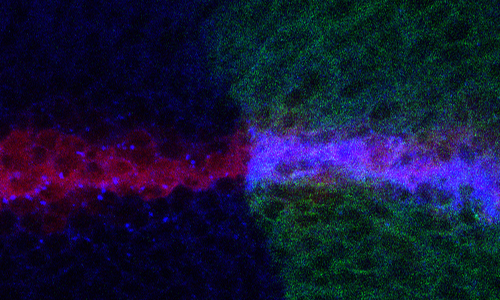

Supplement: Supplementary file 3 — Source data Fig. 1 [file 44319_2024_289_MOESM3_ESM.zip › Figure 1/F1J/F1J images for statistical analysis/4 hh-G4-GFP TH02340 Wg lacZ Wg_Series010_z22.tif]

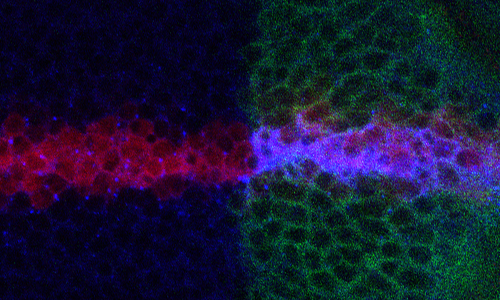

Supplement: Supplementary file 3 — Source data Fig. 1 [file 44319_2024_289_MOESM3_ESM.zip › Figure 1/F1J/F1J images for statistical analysis/5 hh-G4-GFP TH02340 Wg lacZ Wg_Series012_z26.tif]

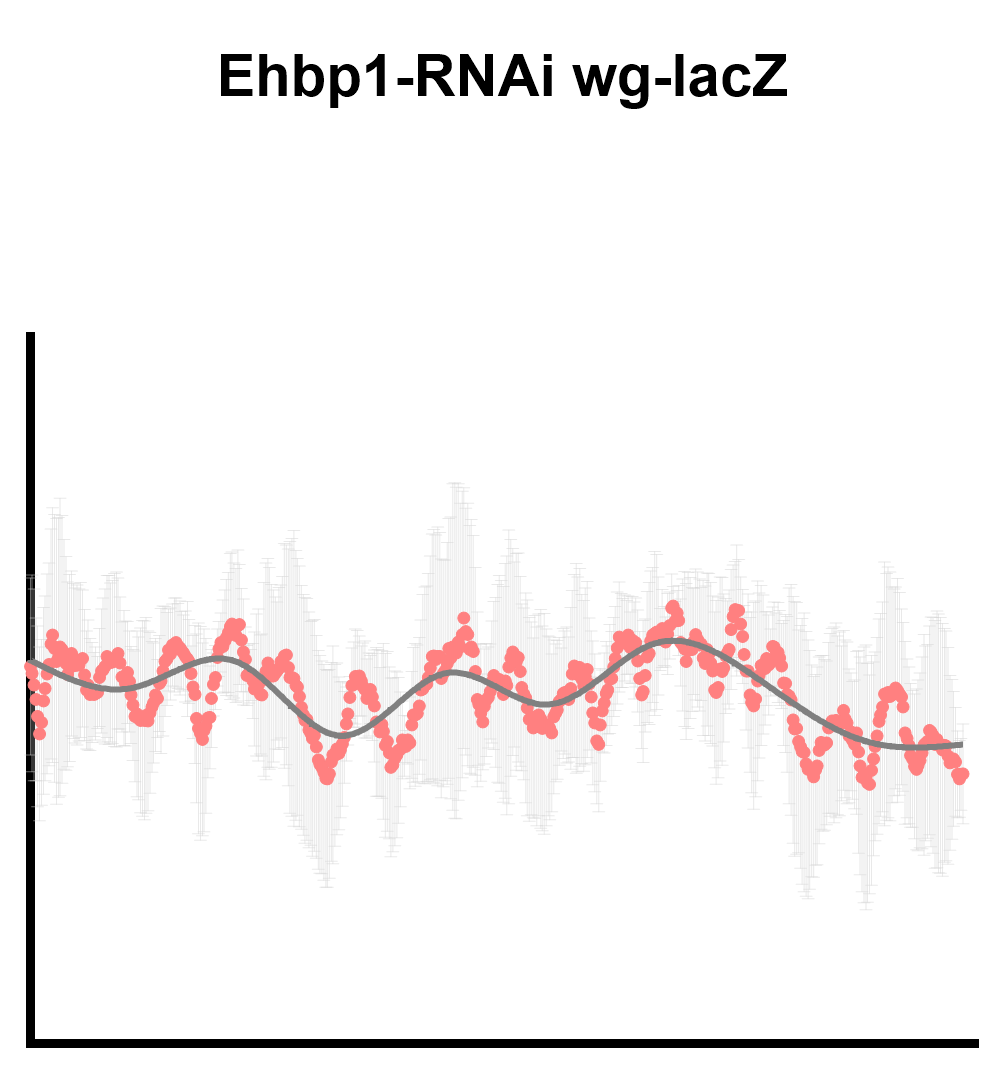

Supplement: Supplementary file 3 — Source data Fig. 1 [file 44319_2024_289_MOESM3_ESM.zip › Figure 1/F1J/F1J statistical analysis- Ehbp1 RNAi-wg-LacZ.tif]

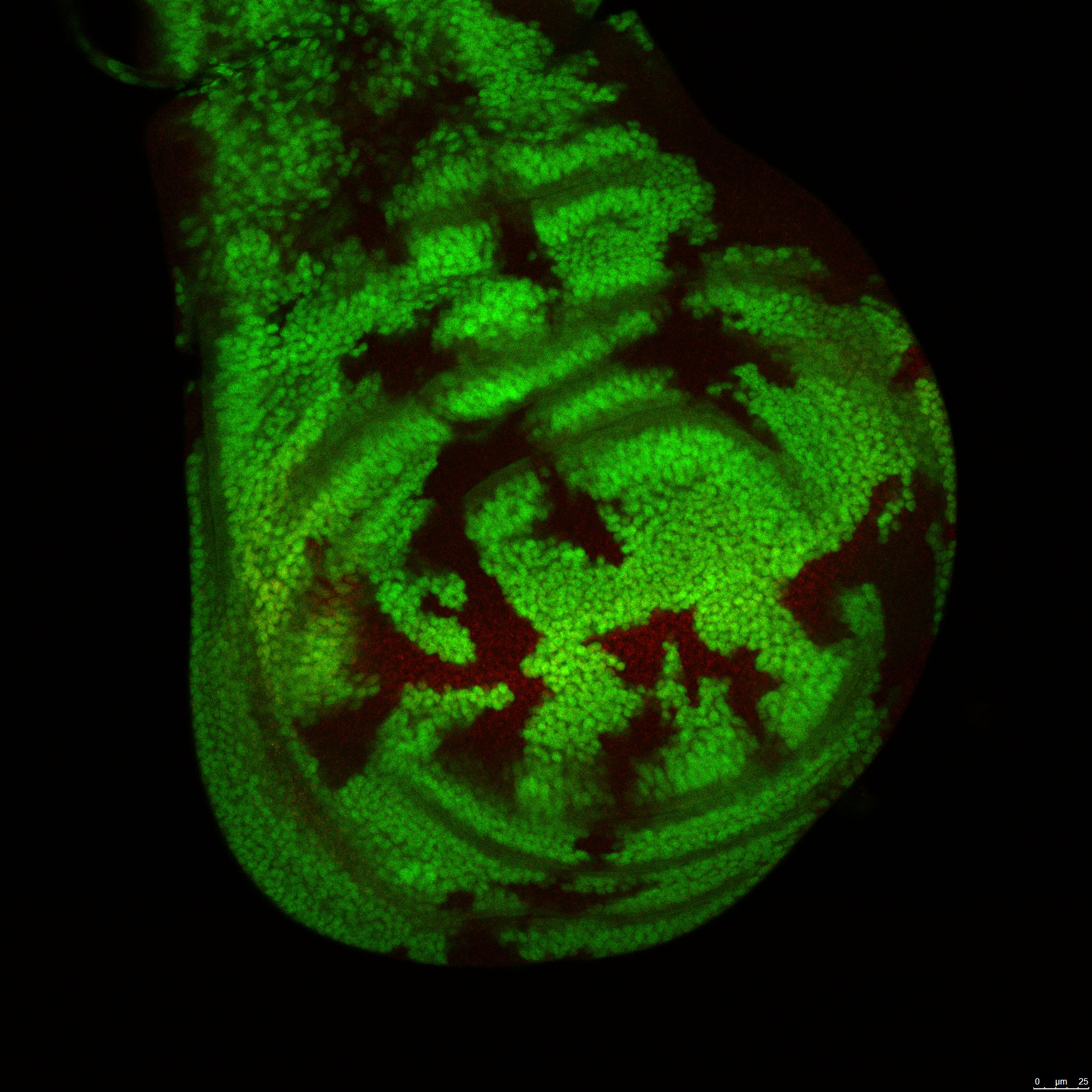

Supplement: Supplementary file 4 — Source data Fig. 2 [file 44319_2024_289_MOESM4_ESM.zip › Figure 2/F2A/F2A 20210418 Ehbp1 mutant Sens Dll Wg.lif_20210418 A28 Minute 42D Dll -1_z0.tif]

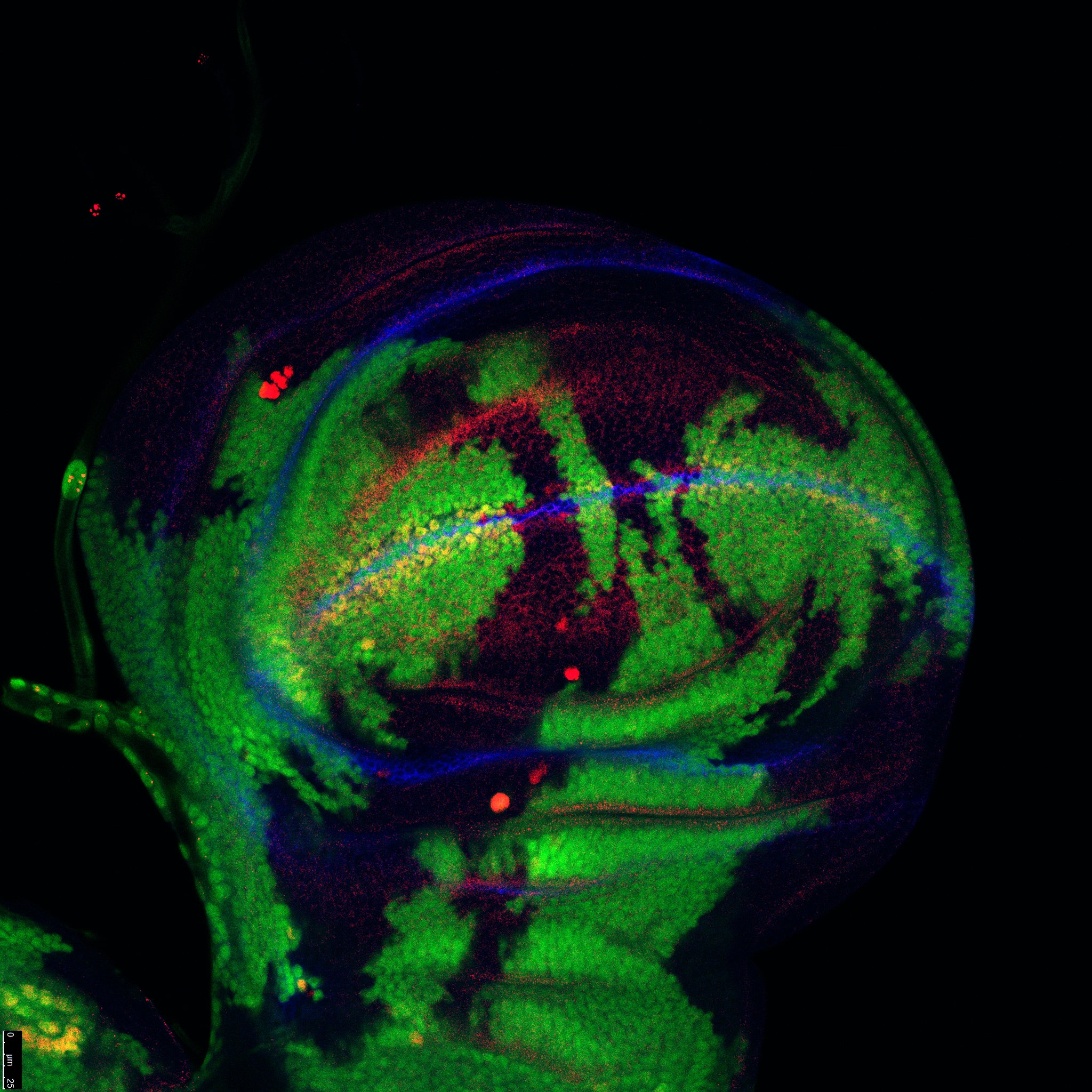

Supplement: Supplementary file 4 — Source data Fig. 2 [file 44319_2024_289_MOESM4_ESM.zip › Figure 2/F2B/F2B 20210418 Ehbp1 mutant Sens Dll Wg.lif_20210418 A28 Minute 42D Sens Wg -2_z0.tif]

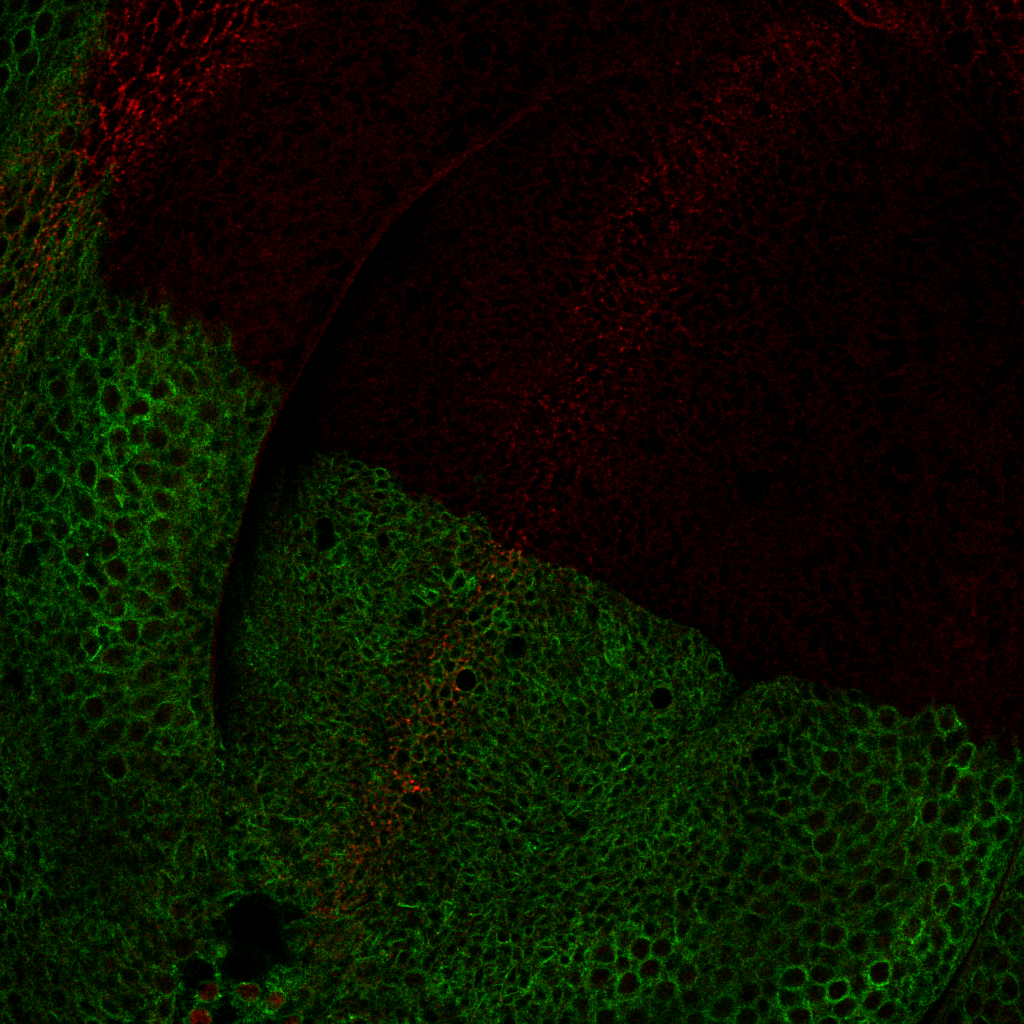

Supplement: Supplementary file 4 — Source data Fig. 2 [file 44319_2024_289_MOESM4_ESM.zip › Figure 2/F2C/F2C1 ts-Gal80 hh-G4-GFP Th02340 Exwg_Series001_Lng_001_z121.tif]

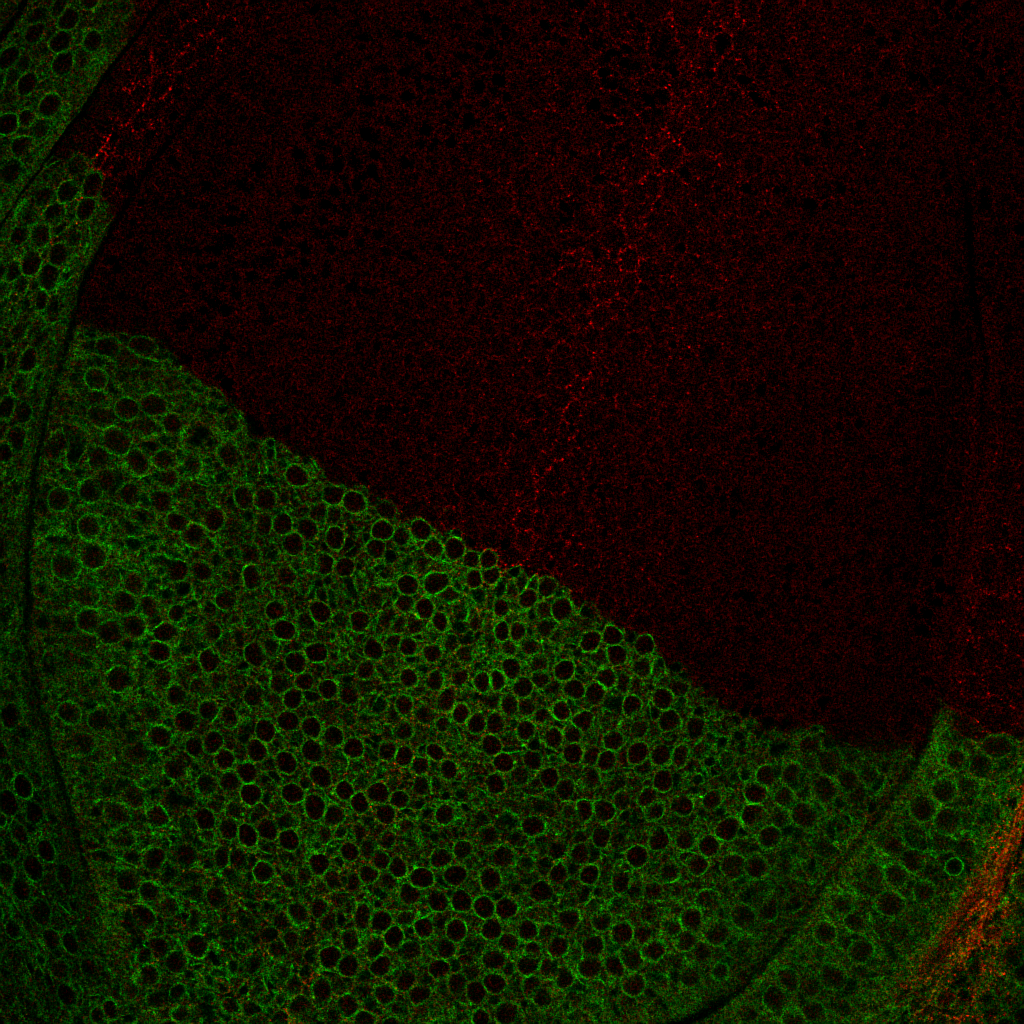

Supplement: Supplementary file 4 — Source data Fig. 2 [file 44319_2024_289_MOESM4_ESM.zip › Figure 2/F2C/F2C2 ts-Gal80 hh-G4-GFP Th02340 Exwg_Series001_Lng_001_z065.tif]

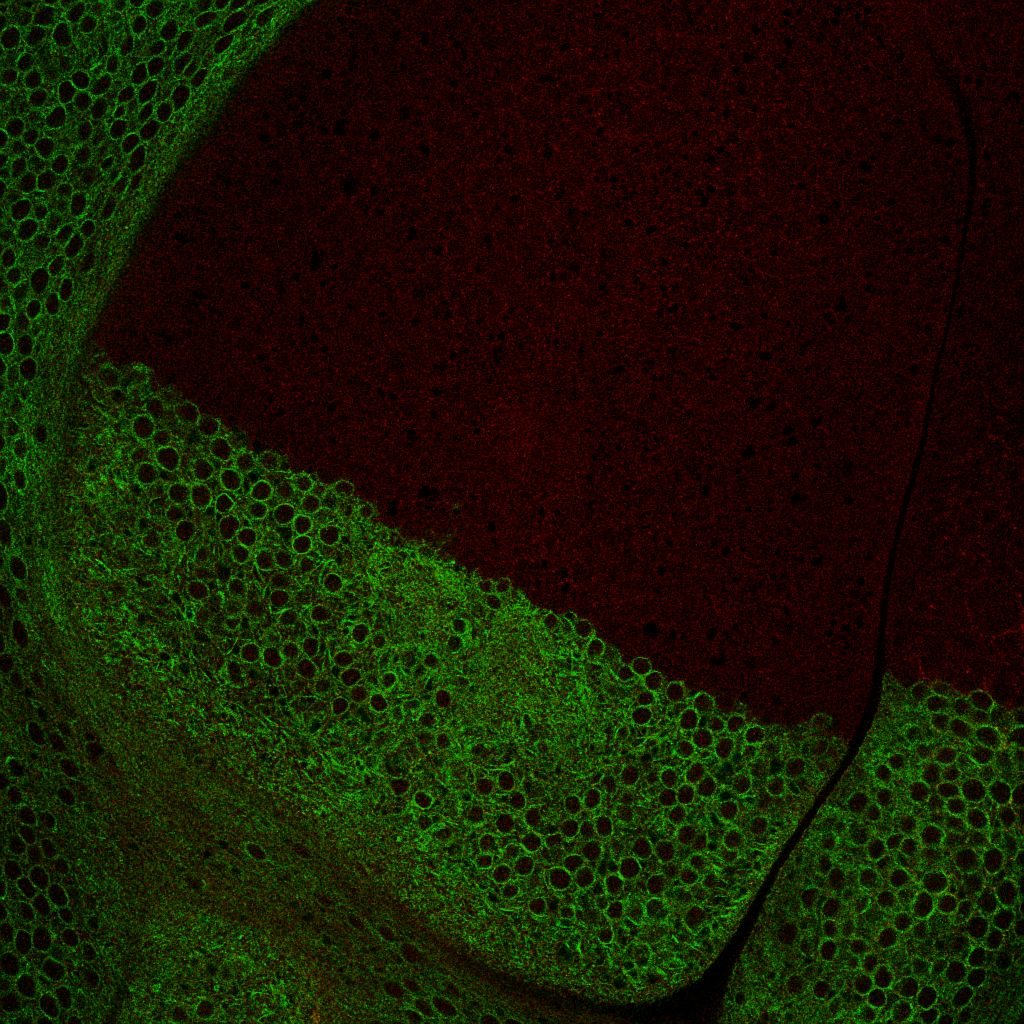

Supplement: Supplementary file 4 — Source data Fig. 2 [file 44319_2024_289_MOESM4_ESM.zip › Figure 2/F2C/F2C3 ts-Gal80 hh-G4-GFP Th02340 Exwg_Series001_Lng_001_z018.tif]

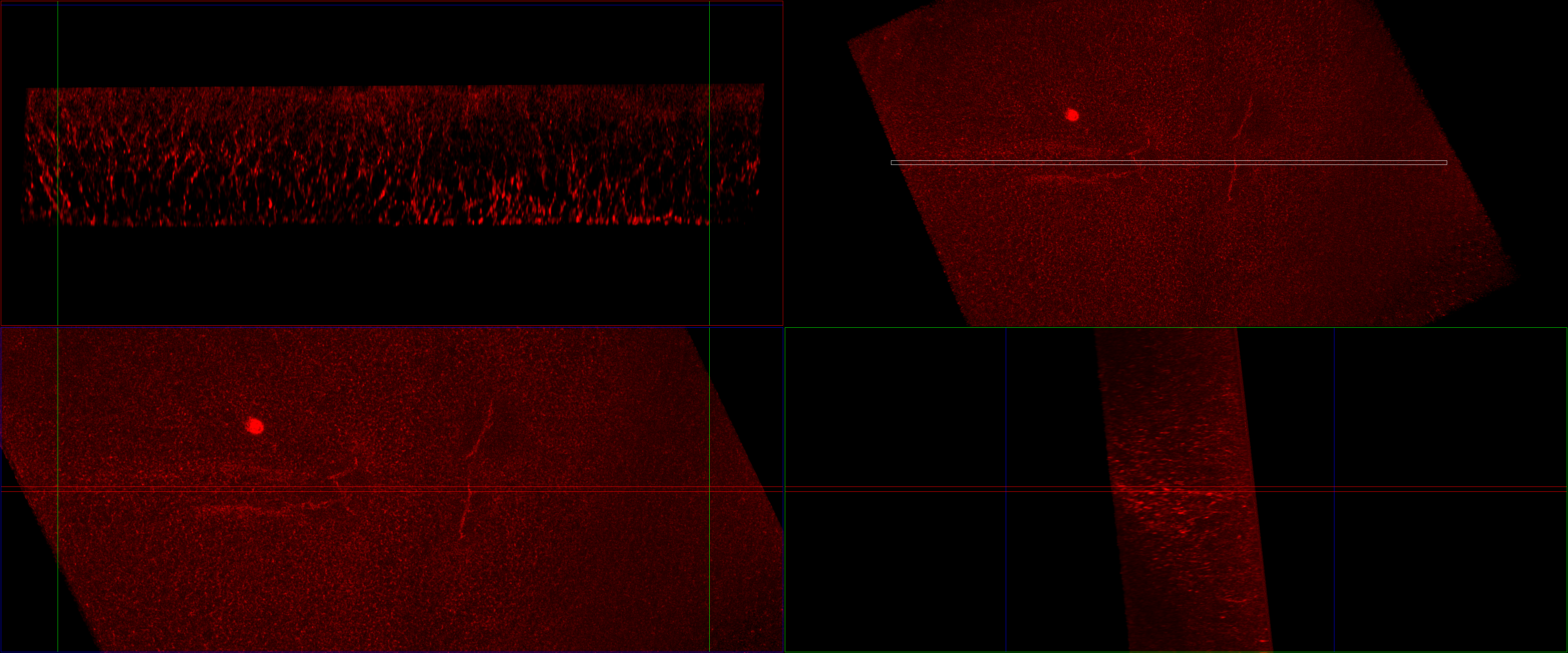

Supplement: Supplementary file 4 — Source data Fig. 2 [file 44319_2024_289_MOESM4_ESM.zip › Figure 2/F2C/F2C4 ts-Gal80 hh-G4-GFP Th02340 Exwg_Series001_Lng_001 - 3D-Projection.tif]

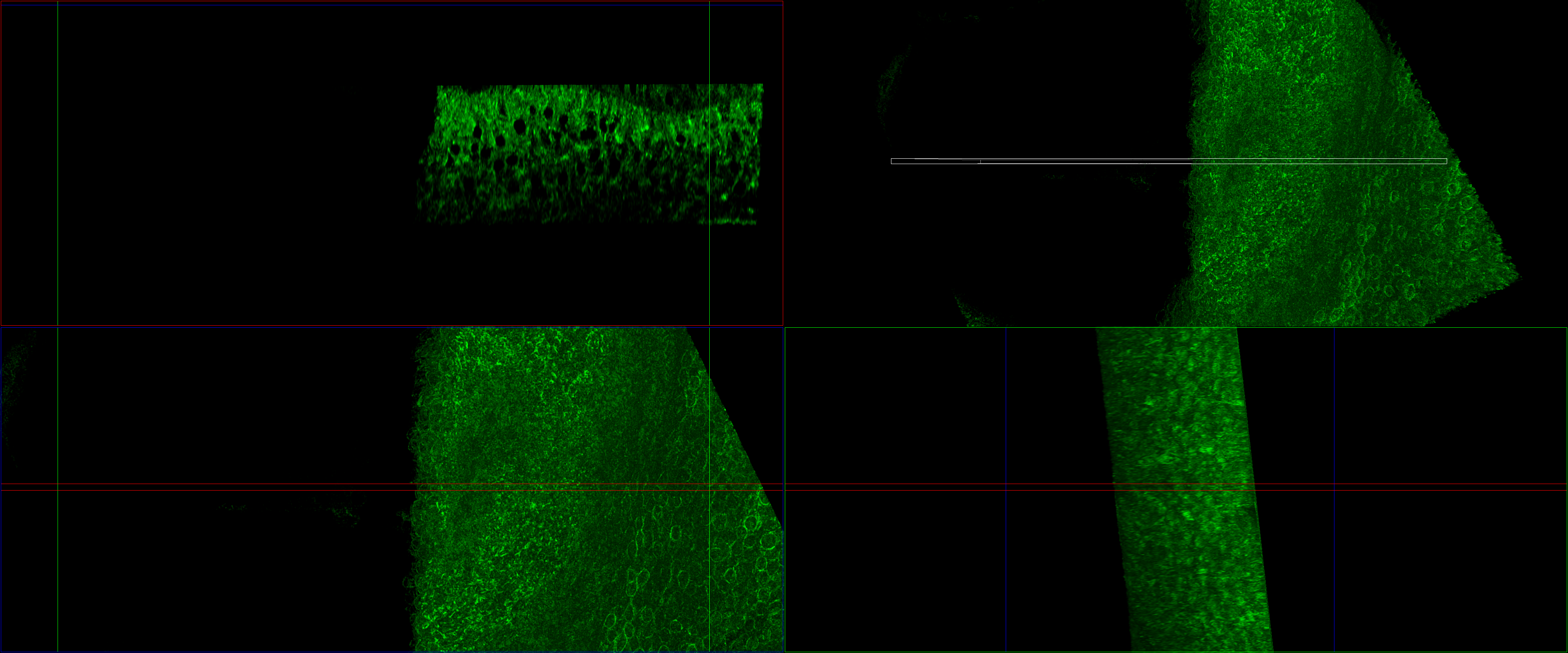

Supplement: Supplementary file 4 — Source data Fig. 2 [file 44319_2024_289_MOESM4_ESM.zip › Figure 2/F2C/F2C4 ts-Gal80 hh-G4-GFP Th02340 Exwg_Series001_Lng_001 - 3D-Projection_1.tif]

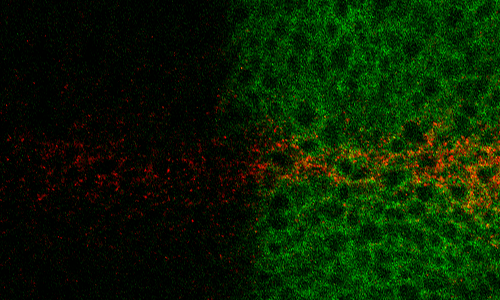

Supplement: Supplementary file 4 — Source data Fig. 2 [file 44319_2024_289_MOESM4_ESM.zip › Figure 2/F2D/F2D Images for statistical analysis/Basalateral ExWg/1 ts-Gal80 hh-G4-GFP Th02340 ExWg_Series009_z27.tif]

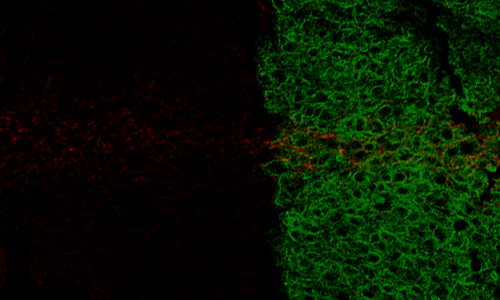

Supplement: Supplementary file 4 — Source data Fig. 2 [file 44319_2024_289_MOESM4_ESM.zip › Figure 2/F2D/F2D Images for statistical analysis/Basalateral ExWg/2 ts-Gal80 hh-G4-GFP Th02340 34h Exwg_Series001_Lng_z118.tif]

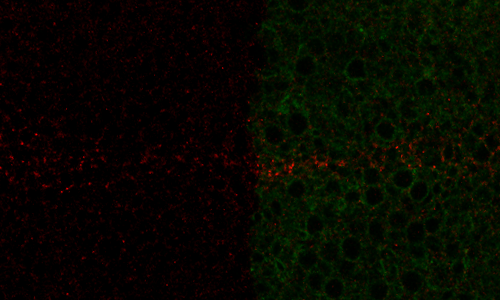

Supplement: Supplementary file 4 — Source data Fig. 2 [file 44319_2024_289_MOESM4_ESM.zip › Figure 2/F2D/F2D Images for statistical analysis/Basalateral ExWg/3 ts-Gal80 hh-G4-GFP Th02340 exwg_Series001_Lng_global_z137.tif]

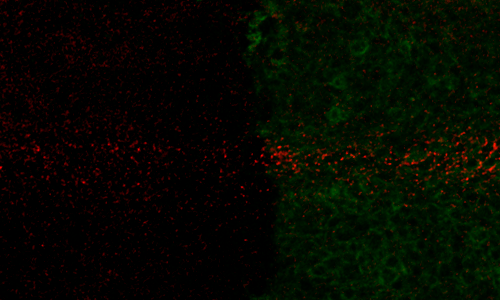

Supplement: Supplementary file 4 — Source data Fig. 2 [file 44319_2024_289_MOESM4_ESM.zip › Figure 2/F2D/F2D Images for statistical analysis/Basalateral ExWg/4 ts-Gal80 hh-G4-GFP TH02340 Exwg_Series002_Lng_global_z125.tif]

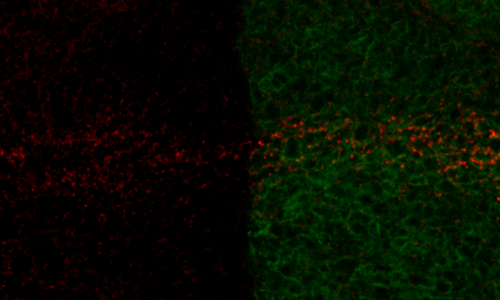

Supplement: Supplementary file 4 — Source data Fig. 2 [file 44319_2024_289_MOESM4_ESM.zip › Figure 2/F2D/F2D Images for statistical analysis/Basalateral ExWg/5 ts-Gal80 hh-G4-GFP TH02340 Exwg_Series004_Lng_adaptive_z123.tif]

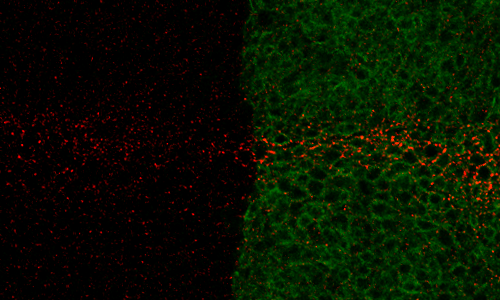

Supplement: Supplementary file 4 — Source data Fig. 2 [file 44319_2024_289_MOESM4_ESM.zip › Figure 2/F2D/F2D Images for statistical analysis/Basalateral ExWg/6 ts-Gal80 hh-G4-GFP TH02340 Exwg_Series006_Lng_global_001_z115.tif]

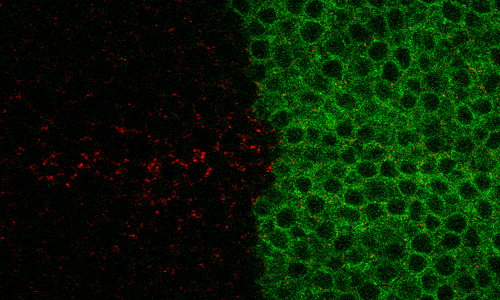

Supplement: Supplementary file 4 — Source data Fig. 2 [file 44319_2024_289_MOESM4_ESM.zip › Figure 2/F2D/F2D Images for statistical analysis/Subapical ExWg/1 ts-Gal80 hh-G4-GFP Th02340 ExWg_Series004_z09.tif]

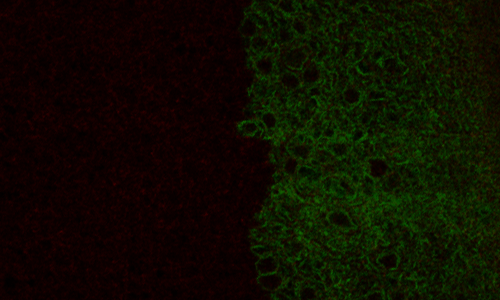

Supplement: Supplementary file 4 — Source data Fig. 2 [file 44319_2024_289_MOESM4_ESM.zip › Figure 2/F2D/F2D Images for statistical analysis/Subapical ExWg/2 ts-Gal80 hh-G4-GFP Th02340 34h Exwg_Series001_Lng_z026.tif]

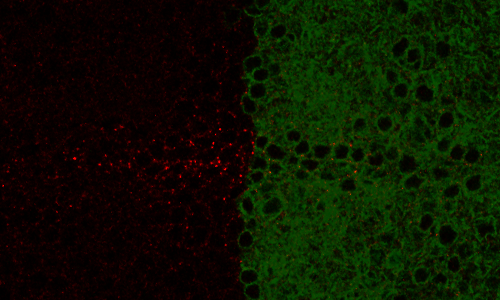

Supplement: Supplementary file 4 — Source data Fig. 2 [file 44319_2024_289_MOESM4_ESM.zip › Figure 2/F2D/F2D Images for statistical analysis/Subapical ExWg/3 ts-Gal80 hh-G4-GFP Th02340 exwg_Series001_Lng_global_z037.tif]

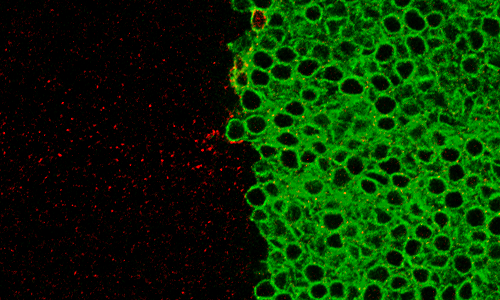

Supplement: Supplementary file 4 — Source data Fig. 2 [file 44319_2024_289_MOESM4_ESM.zip › Figure 2/F2D/F2D Images for statistical analysis/Subapical ExWg/4 ts-Gal80 hh-G4-GFP TH02340 Exwg_Series002_Lng_global_z045.tif]

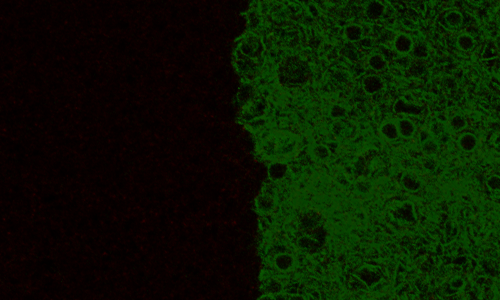

Supplement: Supplementary file 4 — Source data Fig. 2 [file 44319_2024_289_MOESM4_ESM.zip › Figure 2/F2D/F2D Images for statistical analysis/Subapical ExWg/5 ts-Gal80 hh-G4-GFP TH02340 Exwg_Series004_Lng_adaptive_z028.tif]

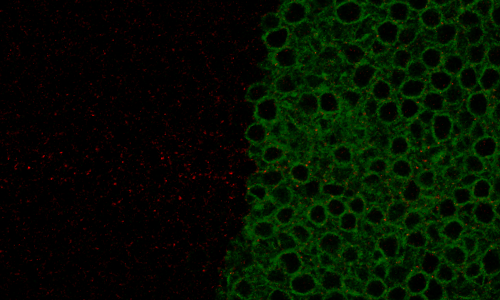

Supplement: Supplementary file 4 — Source data Fig. 2 [file 44319_2024_289_MOESM4_ESM.zip › Figure 2/F2D/F2D Images for statistical analysis/Subapical ExWg/6 ts-Gal80 hh-G4-GFP TH02340 Exwg_Series006_Lng_global_001_z054.tif]

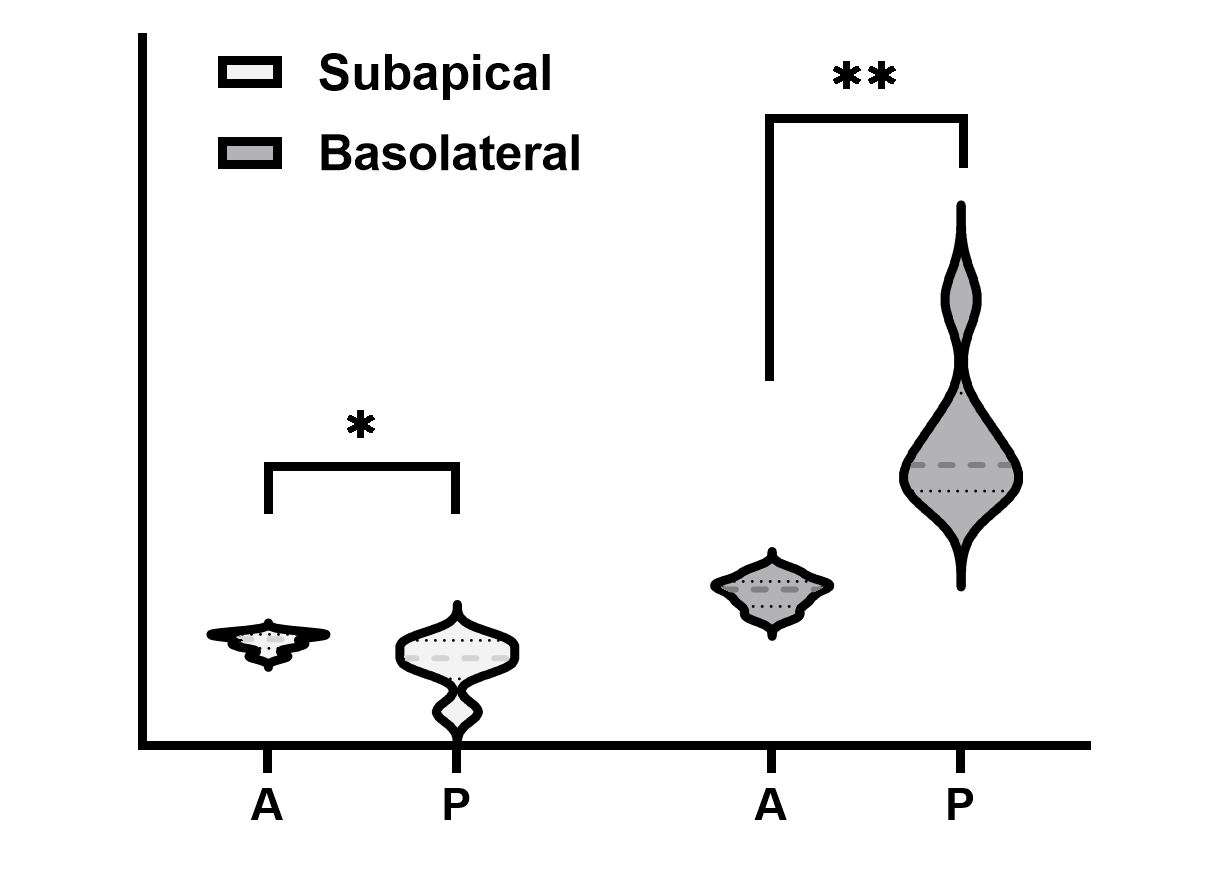

Supplement: Supplementary file 4 — Source data Fig. 2 [file 44319_2024_289_MOESM4_ESM.zip › Figure 2/F2D/F2D.tif]

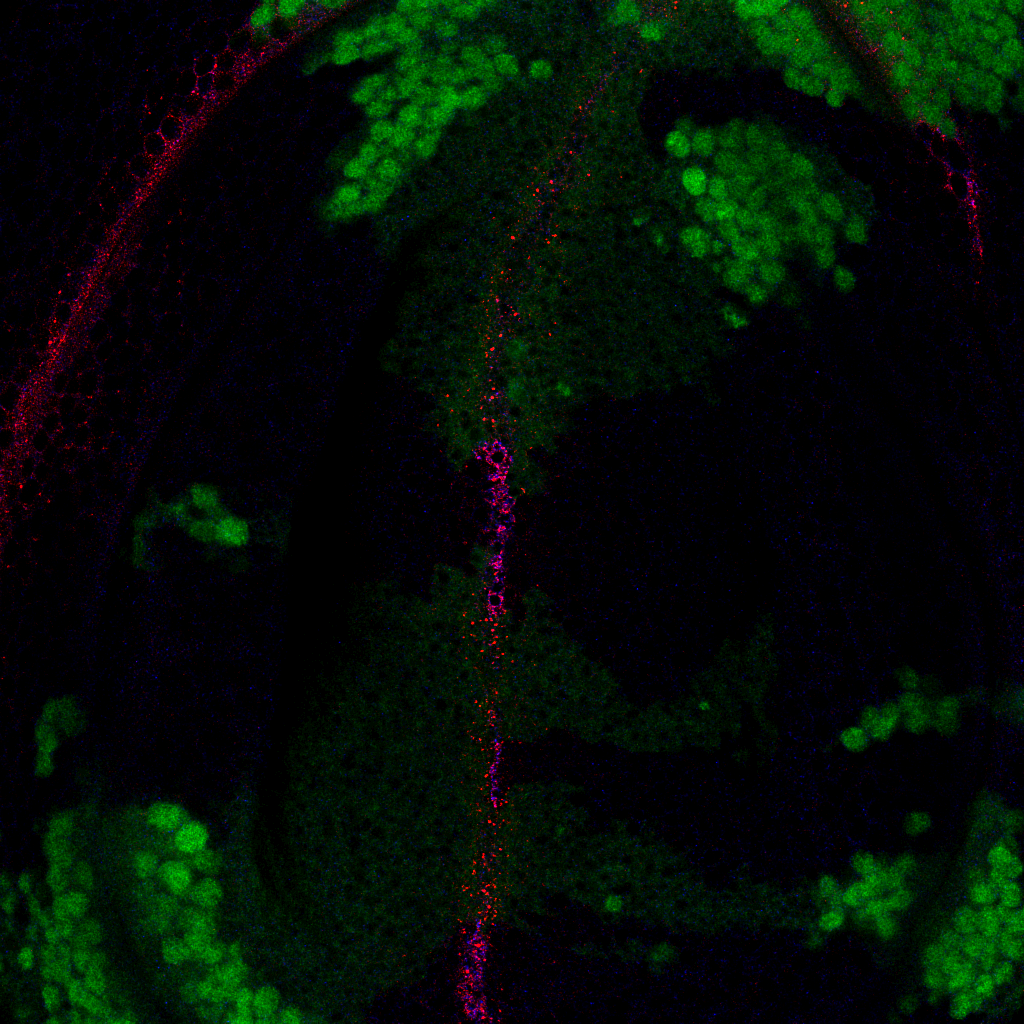

Supplement: Supplementary file 5 — Source data Fig. 3 [file 44319_2024_289_MOESM5_ESM.zip › Figure 3/F3A/F3A1-Ehbp1 mutant Basal - Wg Wls.tif]

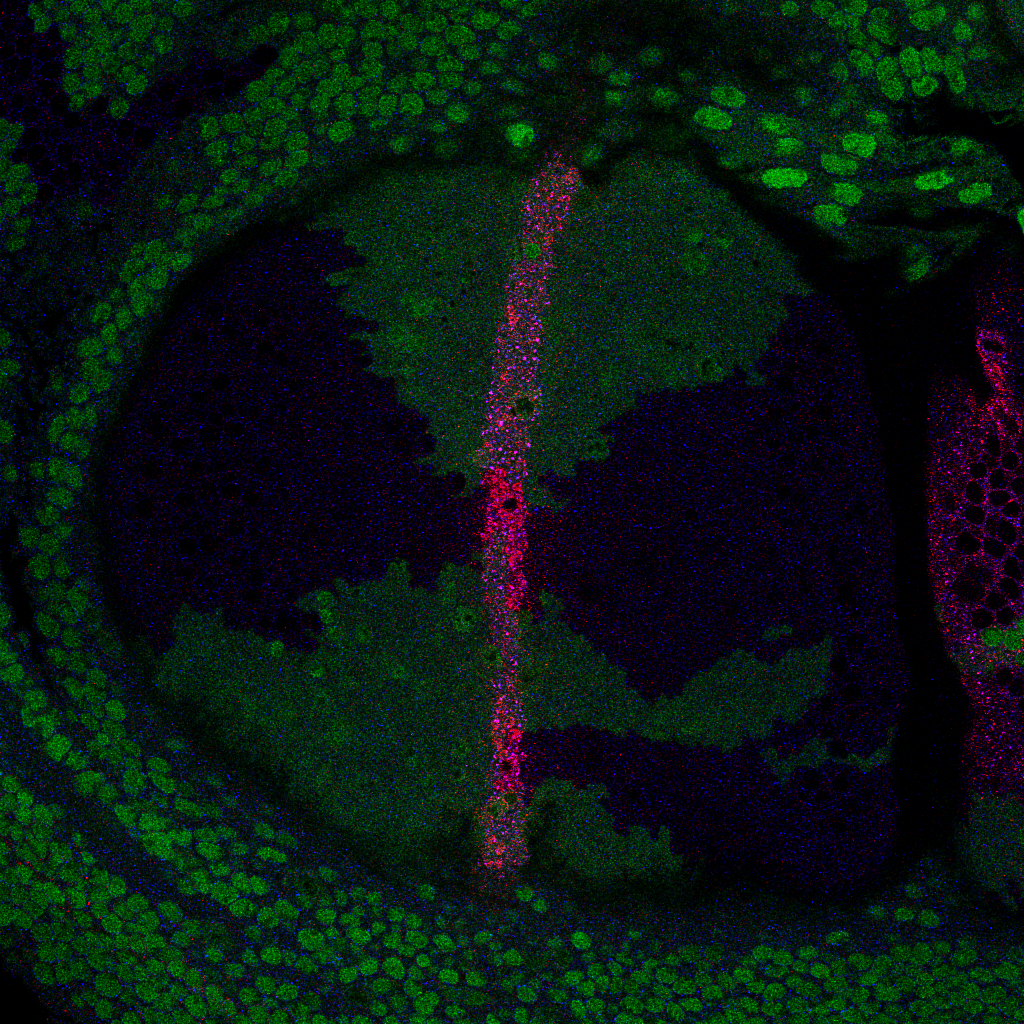

Supplement: Supplementary file 5 — Source data Fig. 3 [file 44319_2024_289_MOESM5_ESM.zip › Figure 3/F3A/F3A2-Ehbp1 mutant Apical-Wg Wls.tif]

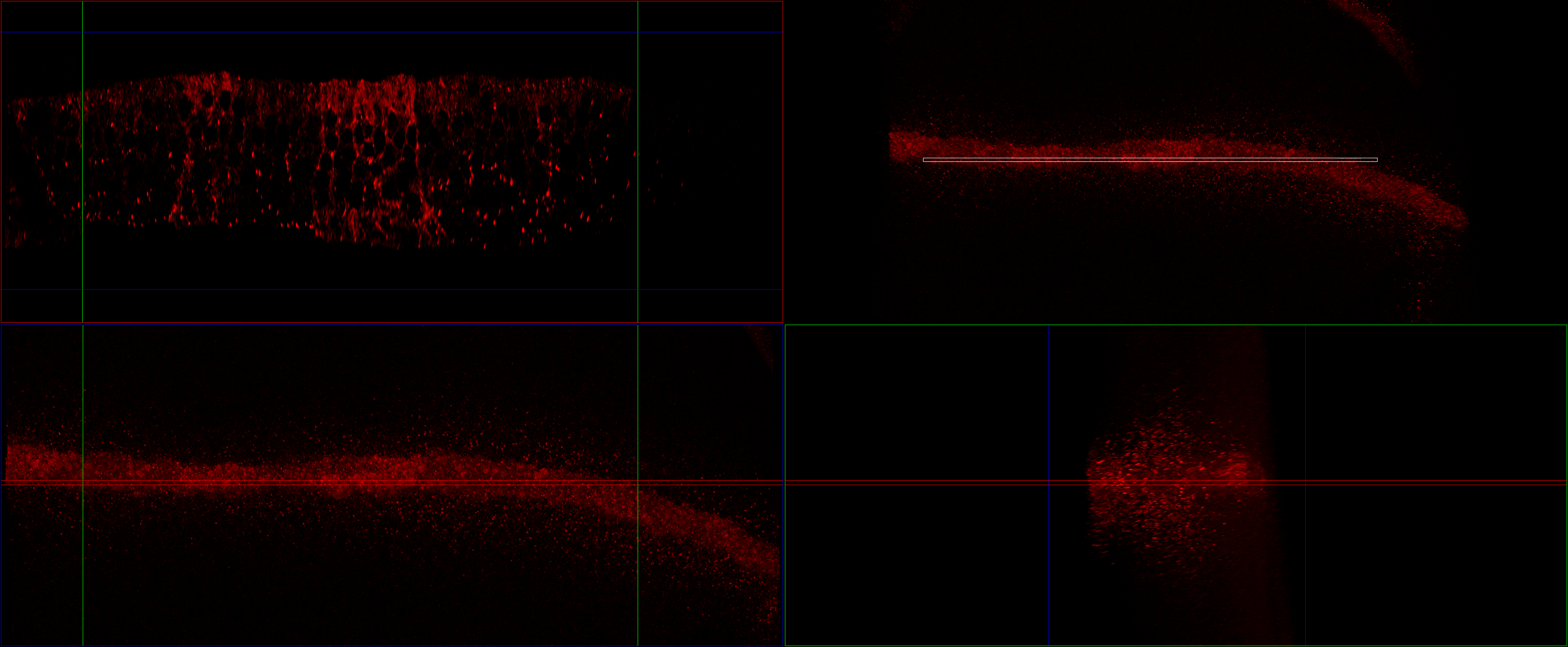

Supplement: Supplementary file 5 — Source data Fig. 3 [file 44319_2024_289_MOESM5_ESM.zip › Figure 3/F3A/F3A3-Ehbp1 mutant X-Z - Wg.tif]

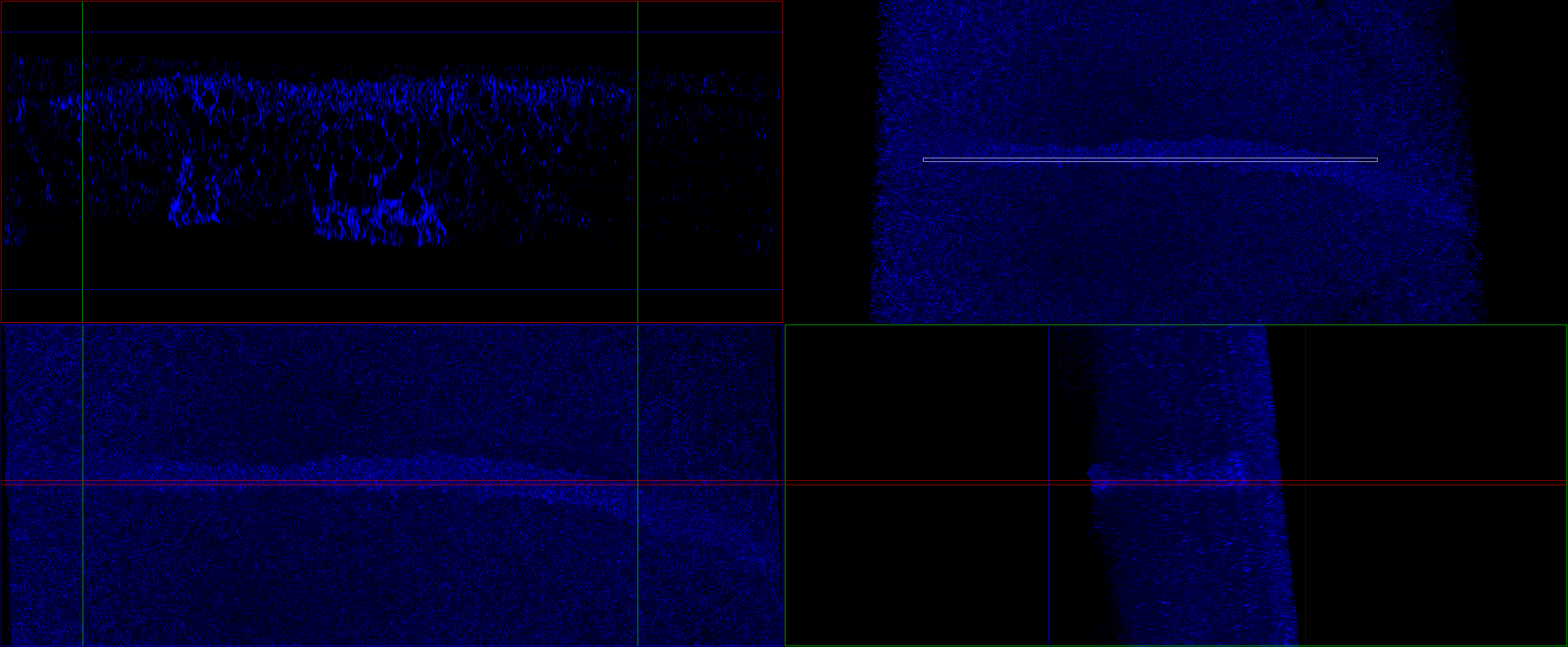

Supplement: Supplementary file 5 — Source data Fig. 3 [file 44319_2024_289_MOESM5_ESM.zip › Figure 3/F3A/F3A3-Ehbp1 mutant X-Z - Wls.tif]

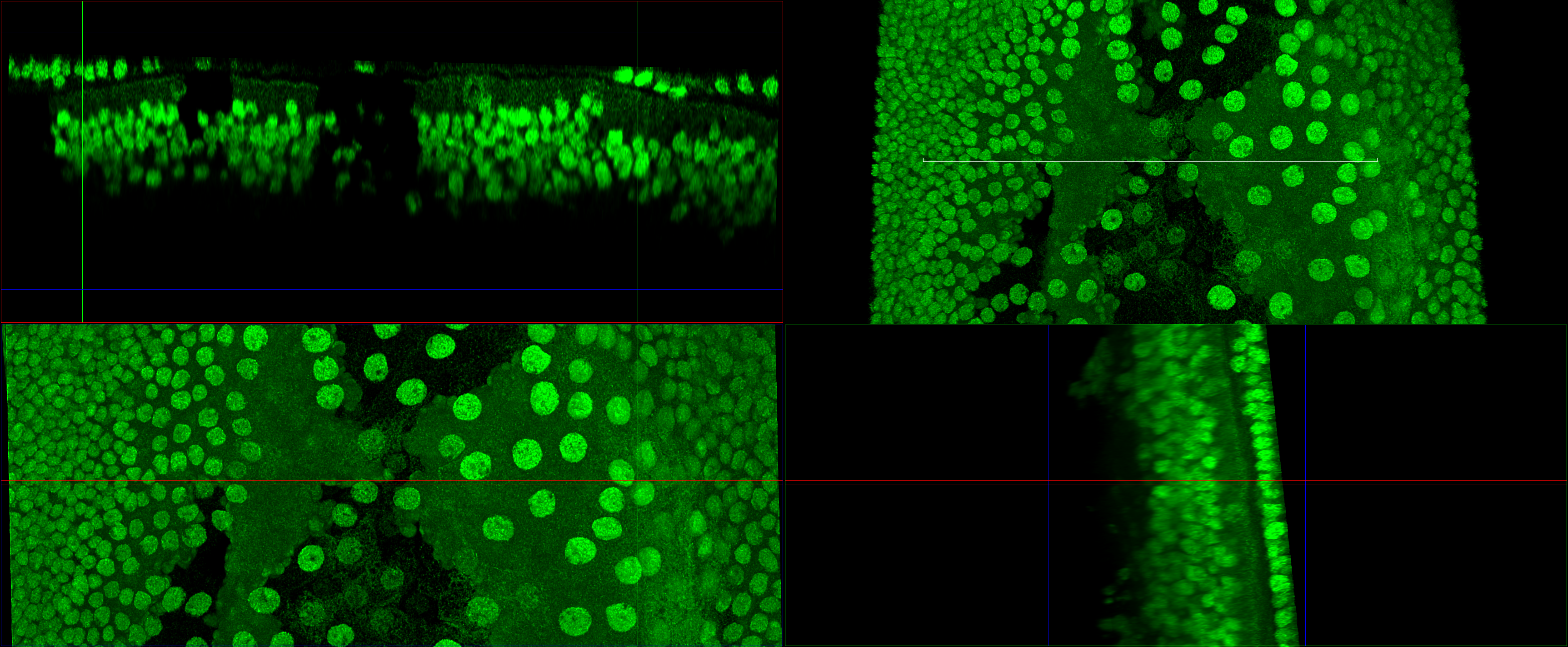

Supplement: Supplementary file 5 — Source data Fig. 3 [file 44319_2024_289_MOESM5_ESM.zip › Figure 3/F3A/F3A3-Ehbp1 mutant X-Z - mosaic clone.tif]

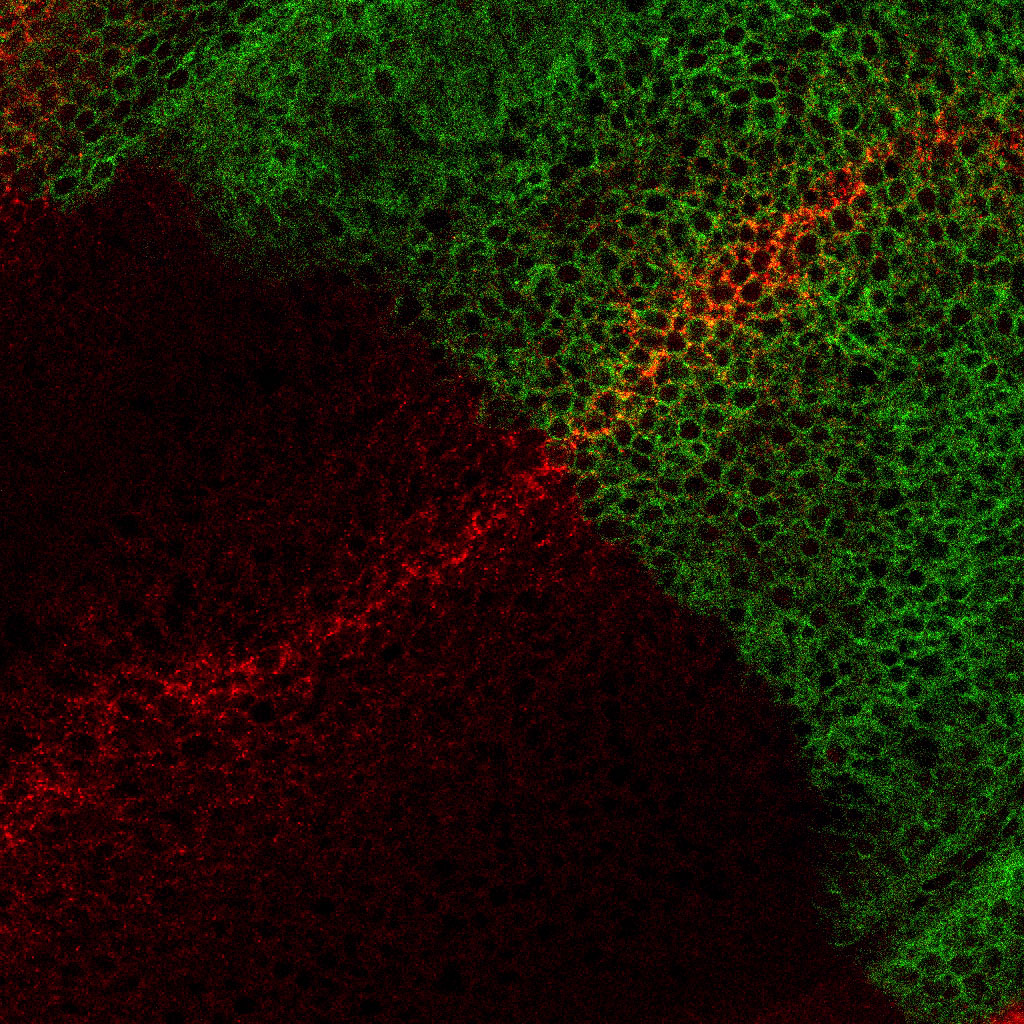

Supplement: Supplementary file 5 — Source data Fig. 3 [file 44319_2024_289_MOESM5_ESM.zip › Figure 3/F3B/F3B ts-Gal80 hh-G4-GFP Th02340 ExWg.lif_Series009_z20.jpg]

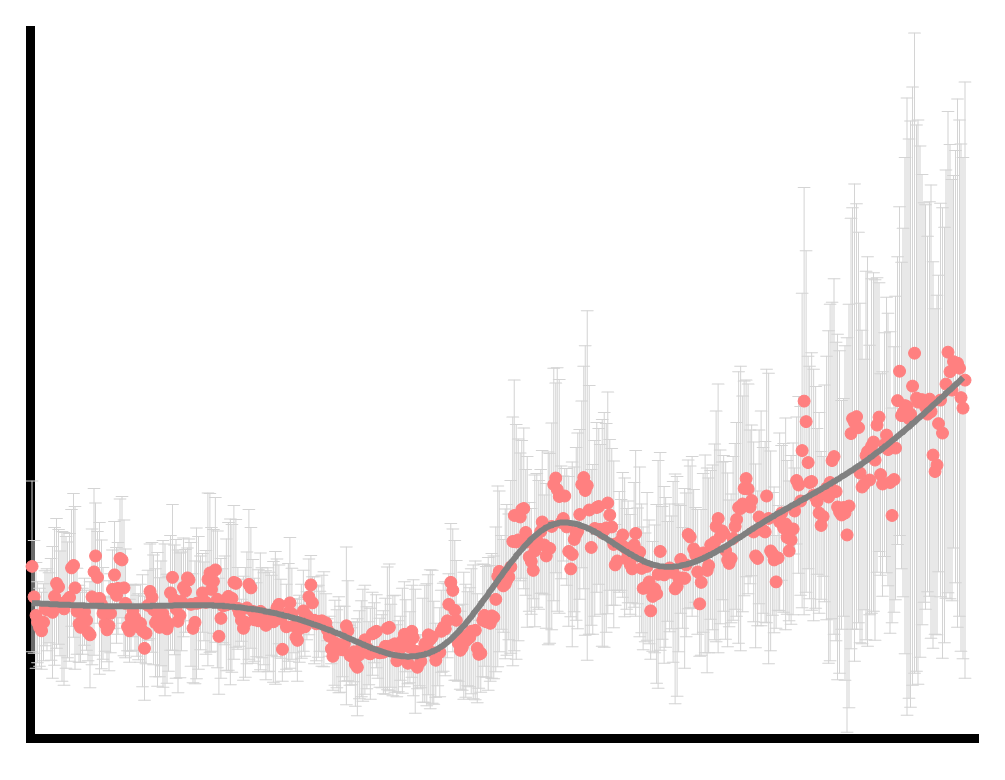

Supplement: Supplementary file 5 — Source data Fig. 3 [file 44319_2024_289_MOESM5_ESM.zip › Figure 3/F3B/F3B'' Ehbp1 RNAi - ExWg.tif]

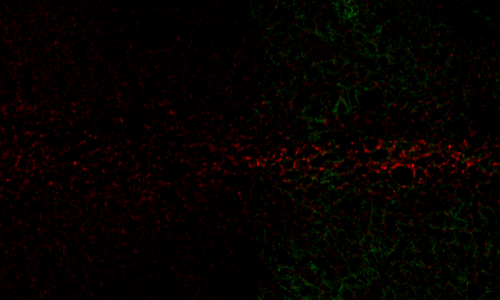

Supplement: Supplementary file 5 — Source data Fig. 3 [file 44319_2024_289_MOESM5_ESM.zip › Figure 3/F3B/F3B'' Image for statistical analysis/3 ts-Gal80 hh-G4-GFP Th02340 Exwg_Series001_Lng_001_z124.tif]

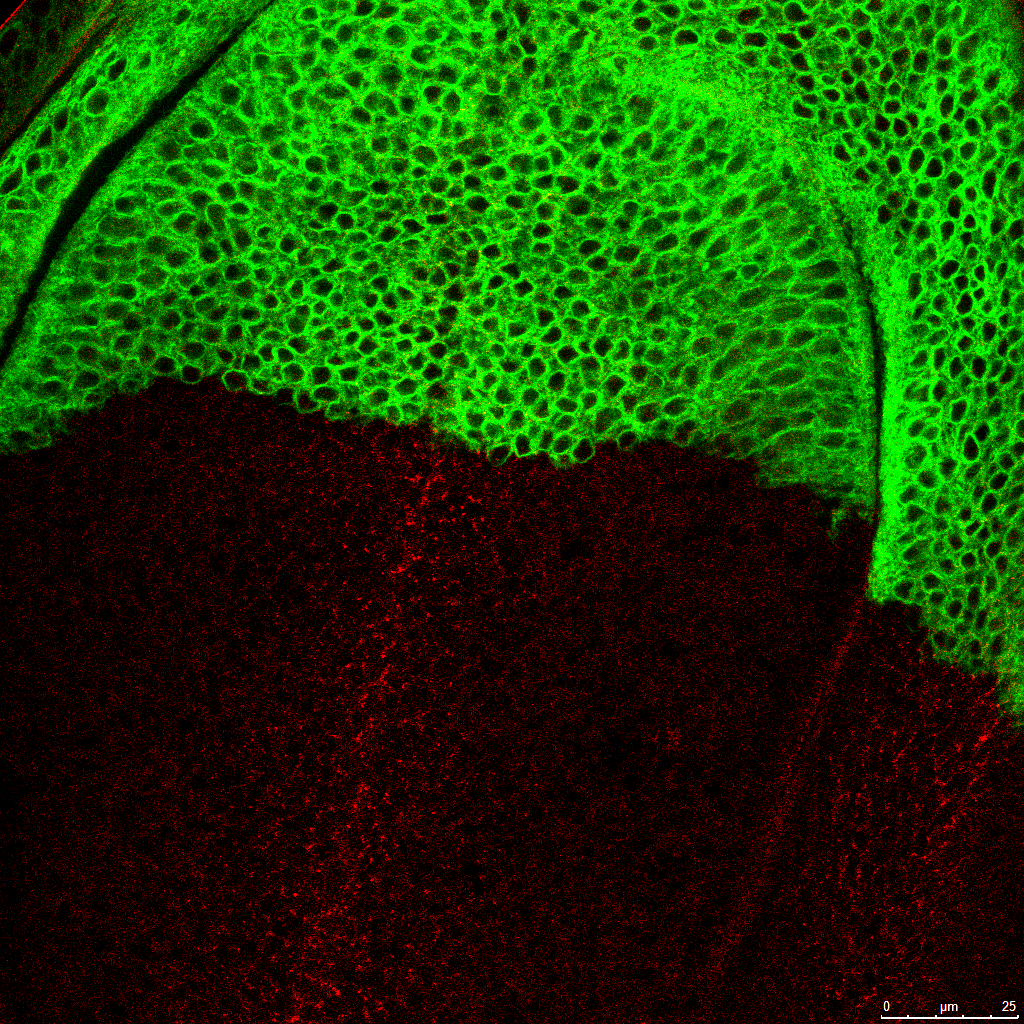

Supplement: Supplementary file 5 — Source data Fig. 3 [file 44319_2024_289_MOESM5_ESM.zip › Figure 3/F3C/F3C hh-G4-GFP wls RNAi ExWg good_Series013_z39.tif]

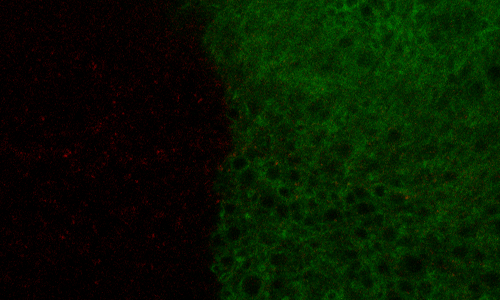

Supplement: Supplementary file 5 — Source data Fig. 3 [file 44319_2024_289_MOESM5_ESM.zip › Figure 3/F3C/F3C'' Images for statistical analysis/1 hh-G4-GFP wls RNAi ExWg bad_Series028_z063.tif]

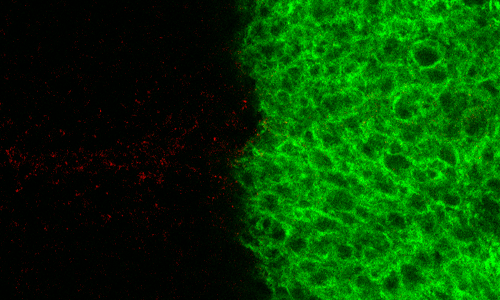

Supplement: Supplementary file 5 — Source data Fig. 3 [file 44319_2024_289_MOESM5_ESM.zip › Figure 3/F3C/F3C'' Images for statistical analysis/2 hh-G4-GFP wls RNAi ExWg good_Series013_z67.tif]

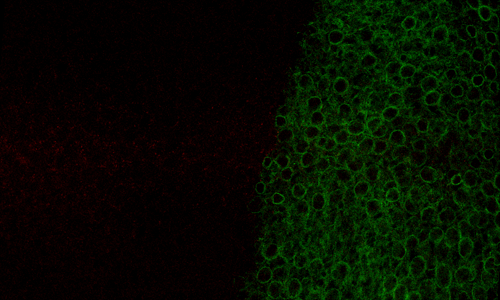

Supplement: Supplementary file 5 — Source data Fig. 3 [file 44319_2024_289_MOESM5_ESM.zip › Figure 3/F3C/F3C'' Images for statistical analysis/3 ts-Gal80 hh-G4-GFP wls RNAi_Series001_000_Lng_adaptive.tif]

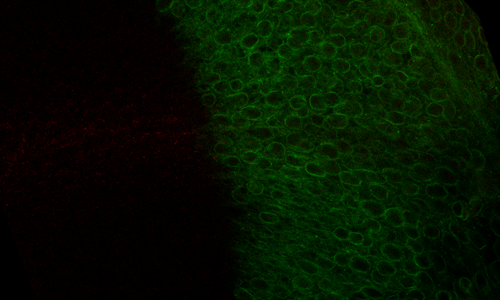

Supplement: Supplementary file 5 — Source data Fig. 3 [file 44319_2024_289_MOESM5_ESM.zip › Figure 3/F3C/F3C'' Images for statistical analysis/4 ts-Gal80 hh-G4-GFP wls RNAi_Series004_Lng_adaptive.tif]

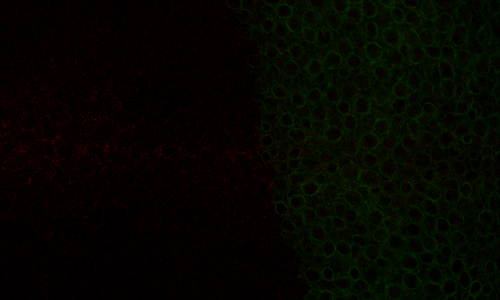

Supplement: Supplementary file 5 — Source data Fig. 3 [file 44319_2024_289_MOESM5_ESM.zip › Figure 3/F3C/F3C'' Images for statistical analysis/5 ts-Gal80 hh-G4-GFP wls RNAi_Series003_Lng_adaptive.tif]

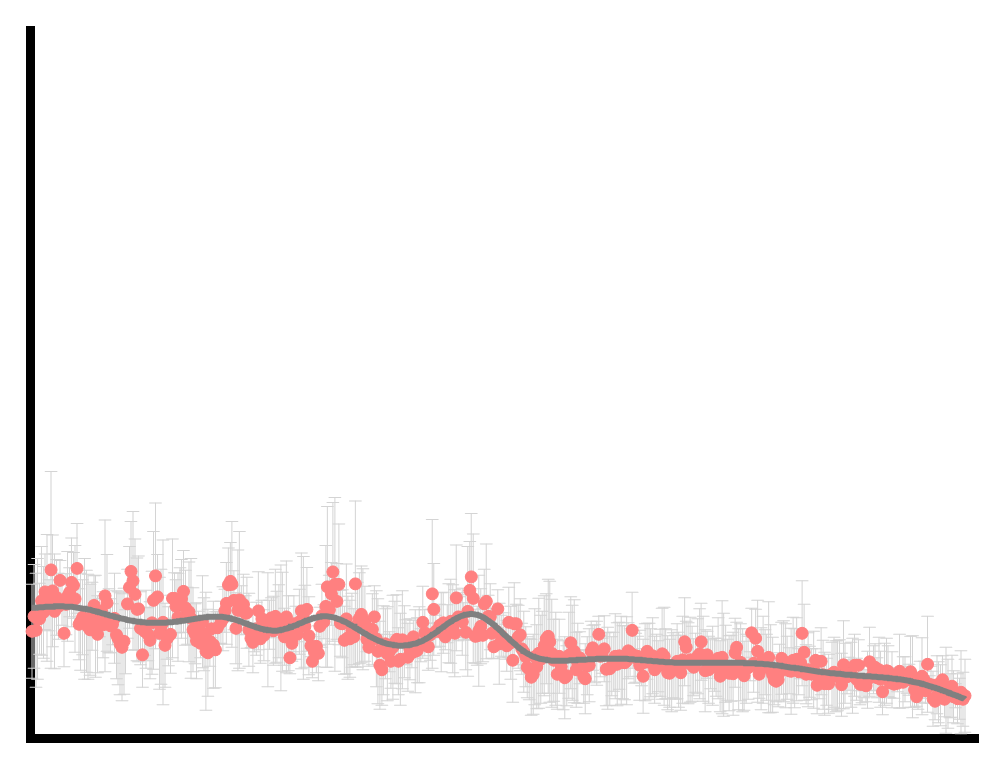

Supplement: Supplementary file 5 — Source data Fig. 3 [file 44319_2024_289_MOESM5_ESM.zip › Figure 3/F3C/F3C'' wls RNAi - ExWg.tif]

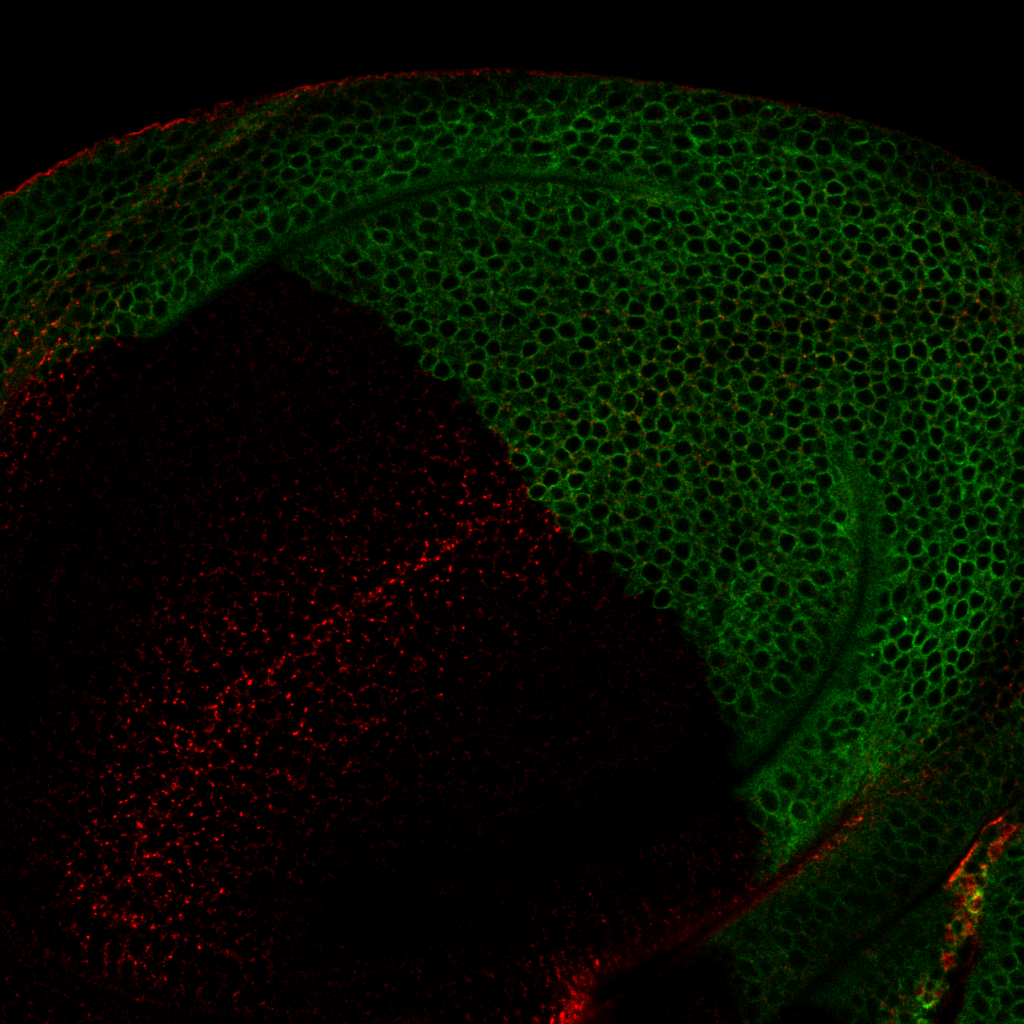

Supplement: Supplementary file 5 — Source data Fig. 3 [file 44319_2024_289_MOESM5_ESM.zip › Figure 3/F3D/F3D and F3D' - double RNAi - ExWg .tif]

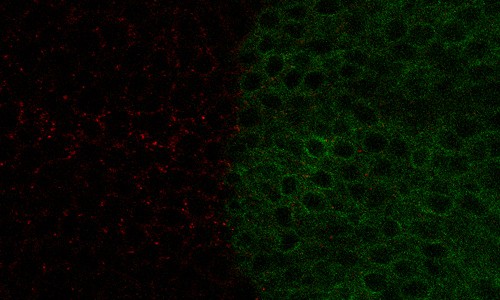

Supplement: Supplementary file 5 — Source data Fig. 3 [file 44319_2024_289_MOESM5_ESM.zip › Figure 3/F3D/F3D'' Images for statistical analysis - double RNAi - ExWg/1 hh-G4-GFP EHBP1 RNAi and Wls RNAi Wg ExWg_Series012_z09.tif]

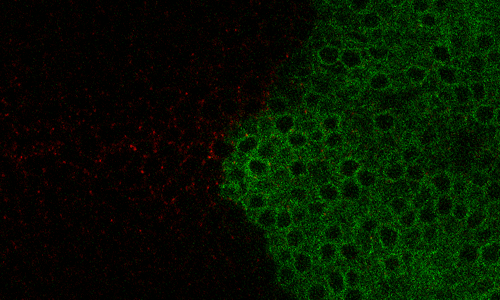

Supplement: Supplementary file 5 — Source data Fig. 3 [file 44319_2024_289_MOESM5_ESM.zip › Figure 3/F3D/F3D'' Images for statistical analysis - double RNAi - ExWg/2 hh-G4-GFP wls RNAi EHBP1 RNAi ExWg 2_Series004_z075.tif]

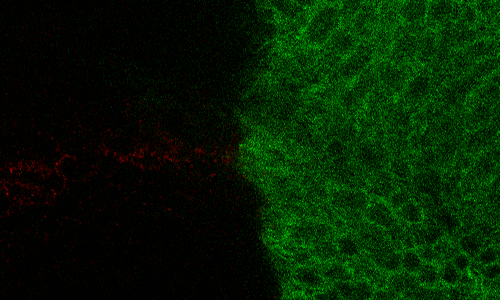

Supplement: Supplementary file 5 — Source data Fig. 3 [file 44319_2024_289_MOESM5_ESM.zip › Figure 3/F3D/F3D'' Images for statistical analysis - double RNAi - ExWg/3 hh-G4-GFP wls RNAi EHBP1 RNAi ExWg 2_Series007_z075.tif]

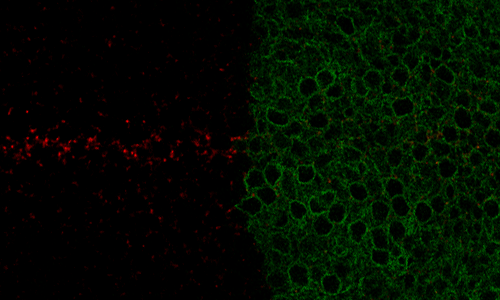

Supplement: Supplementary file 5 — Source data Fig. 3 [file 44319_2024_289_MOESM5_ESM.zip › Figure 3/F3D/F3D'' Images for statistical analysis - double RNAi - ExWg/4 hh-G4-GFP wls Ehbp1 double RNAi wg exwg_Series001_000_Lng_z073.tif]

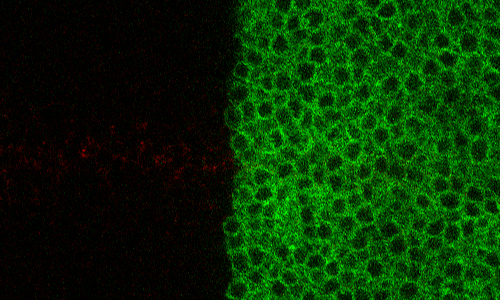

Supplement: Supplementary file 5 — Source data Fig. 3 [file 44319_2024_289_MOESM5_ESM.zip › Figure 3/F3D/F3D'' Images for statistical analysis - double RNAi - ExWg/5 hh-G4-GFP wls Ehbp1 double RNAi wg exwg_Series001_z071.tif]

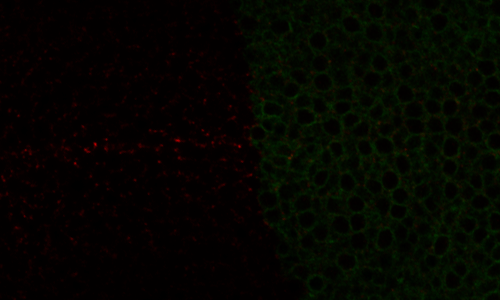

Supplement: Supplementary file 5 — Source data Fig. 3 [file 44319_2024_289_MOESM5_ESM.zip › Figure 3/F3D/F3D'' Images for statistical analysis - double RNAi - ExWg/6 hh-G4-GFP wls Ehbp1 double RNAi wg exwg_Series002_Lng_z095.tif]

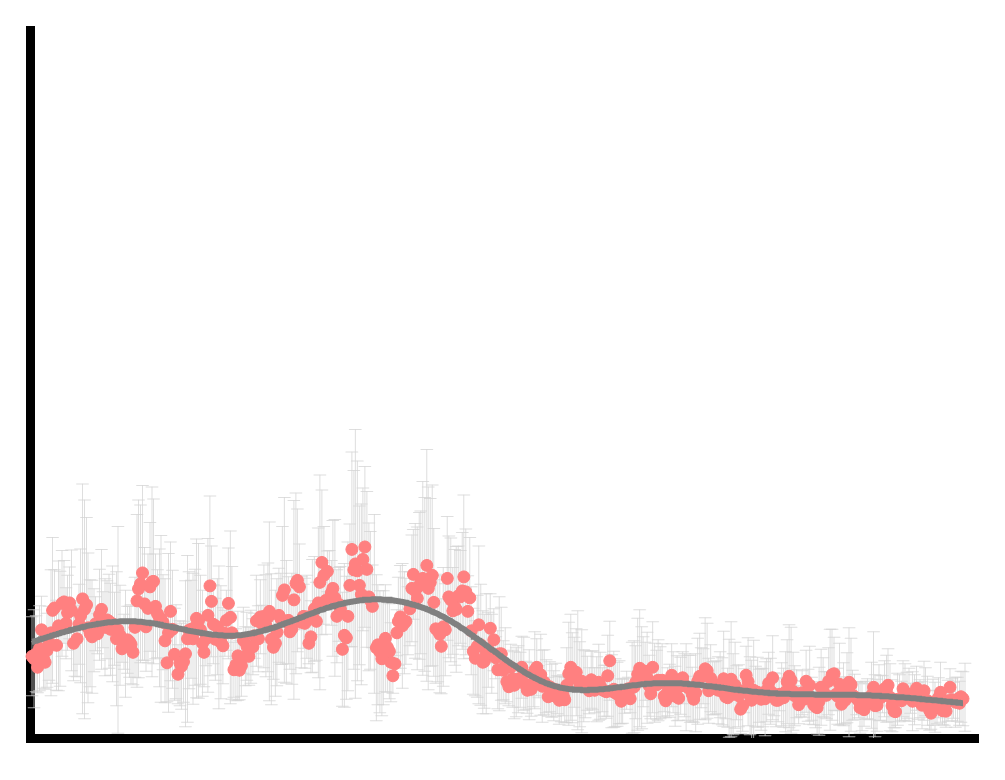

Supplement: Supplementary file 5 — Source data Fig. 3 [file 44319_2024_289_MOESM5_ESM.zip › Figure 3/F3D/F3D'' double RNAi - ExWg.tif]

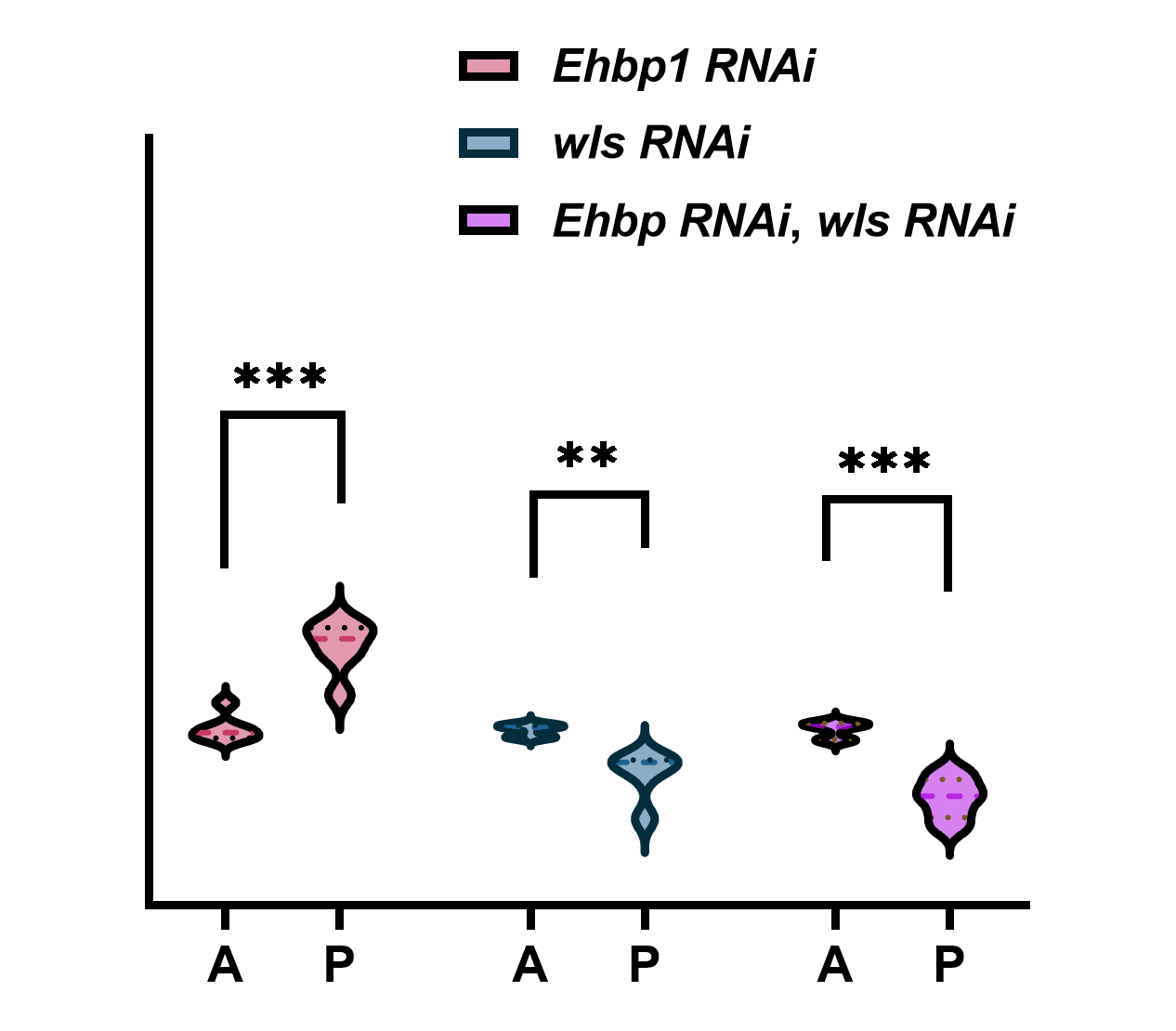

Supplement: Supplementary file 5 — Source data Fig. 3 [file 44319_2024_289_MOESM5_ESM.zip › Figure 3/F3E/F3E ExWg.tif]

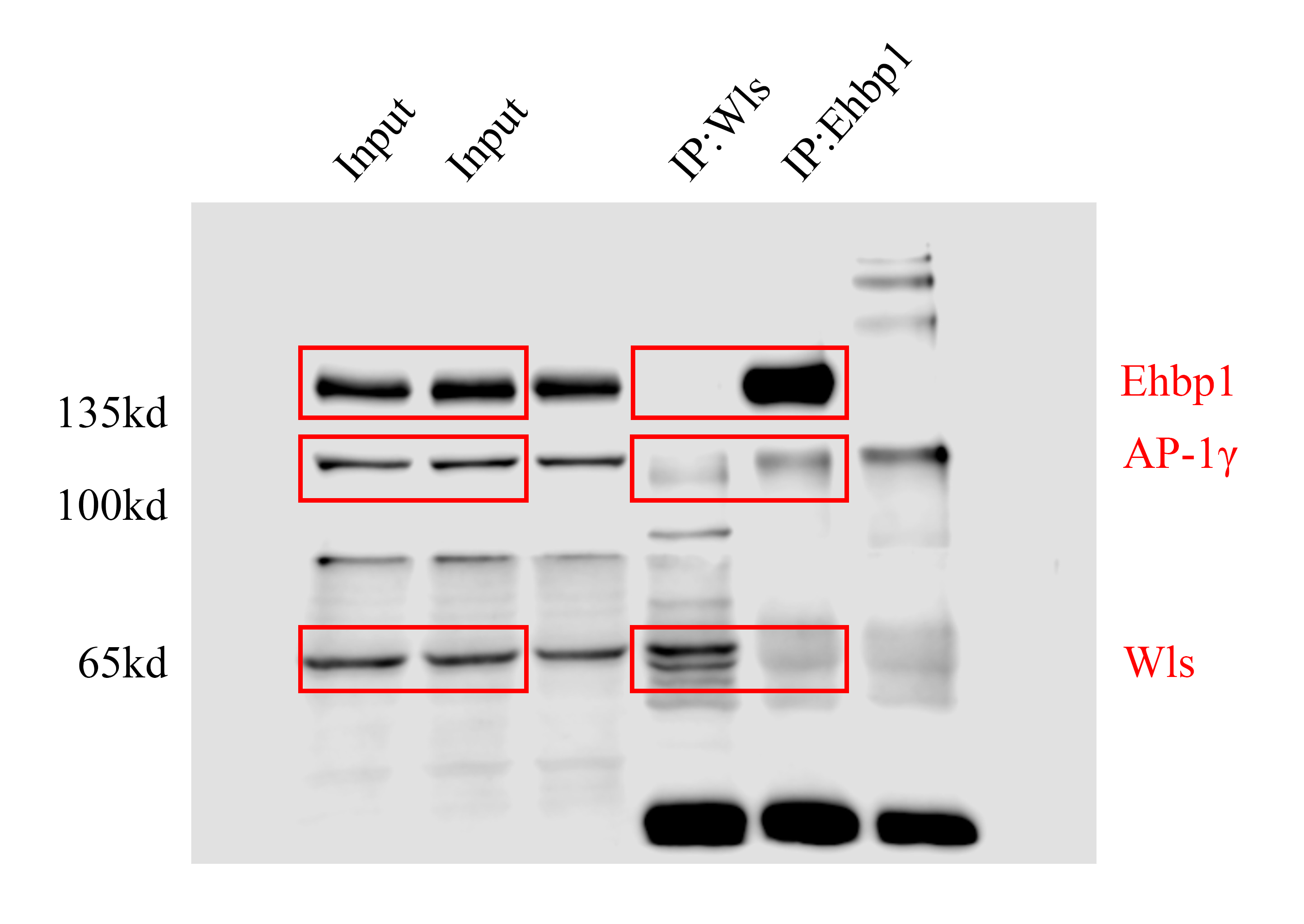

Supplement: Supplementary file 5 — Source data Fig. 3 [file 44319_2024_289_MOESM5_ESM.zip › Figure 3/F3F/F3F endogenous IP- Wls AP1gamma Ehbp1.tif]

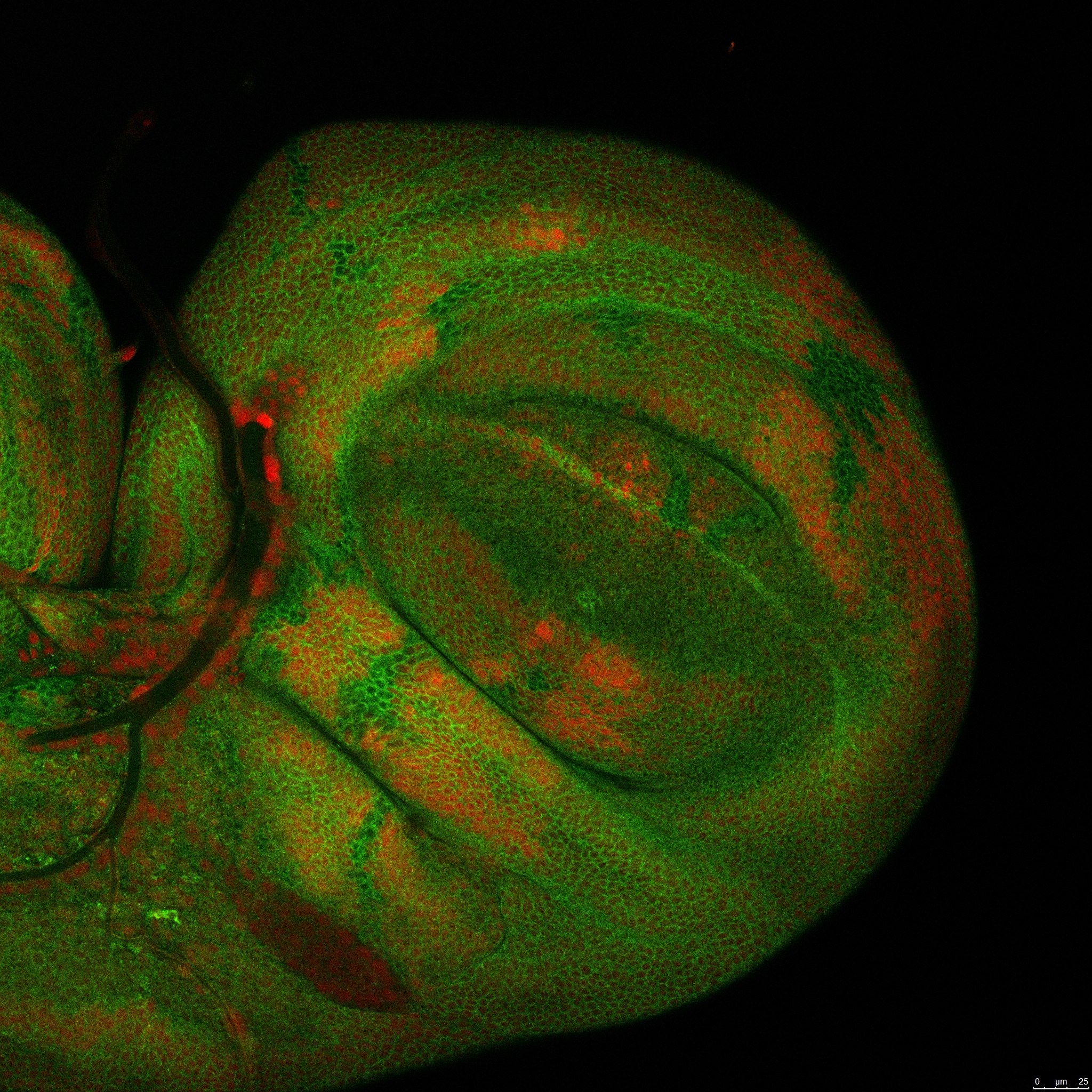

Supplement: Supplementary file 6 — Source data Fig. 4 [file 44319_2024_289_MOESM6_ESM.zip › Figure 4/F4A/F4A 20210730 AP-1 Wls.lif_20210730 hs-Flp 19A AP-1rD Wls -4 -1_z0.tif]

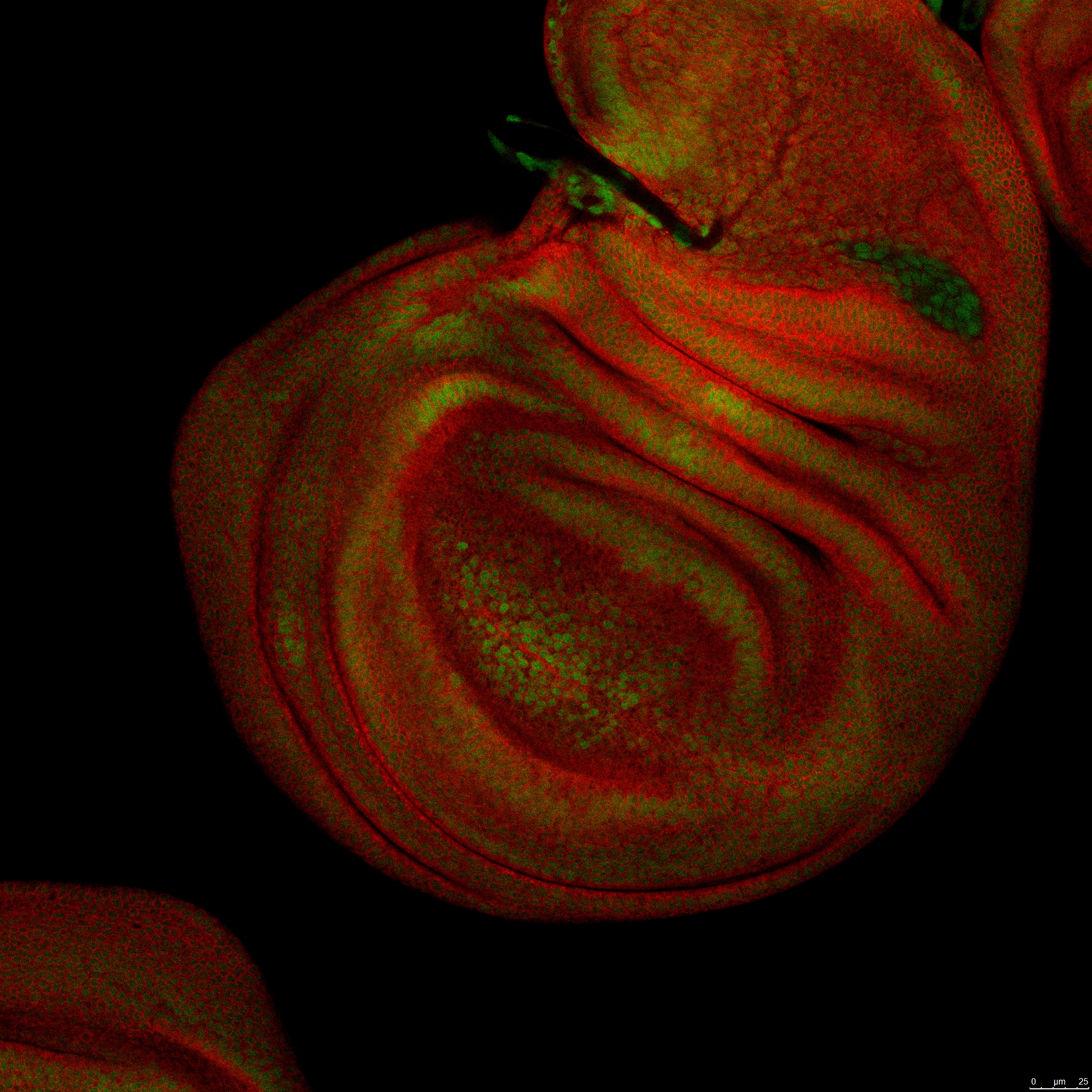

Supplement: Supplementary file 6 — Source data Fig. 4 [file 44319_2024_289_MOESM6_ESM.zip › Figure 4/F4B/F4B 20210730 AP-1 Wls.lif_20210730 Ubx-Flp 82B AP-47 Wls -3 -1_z0.tif]

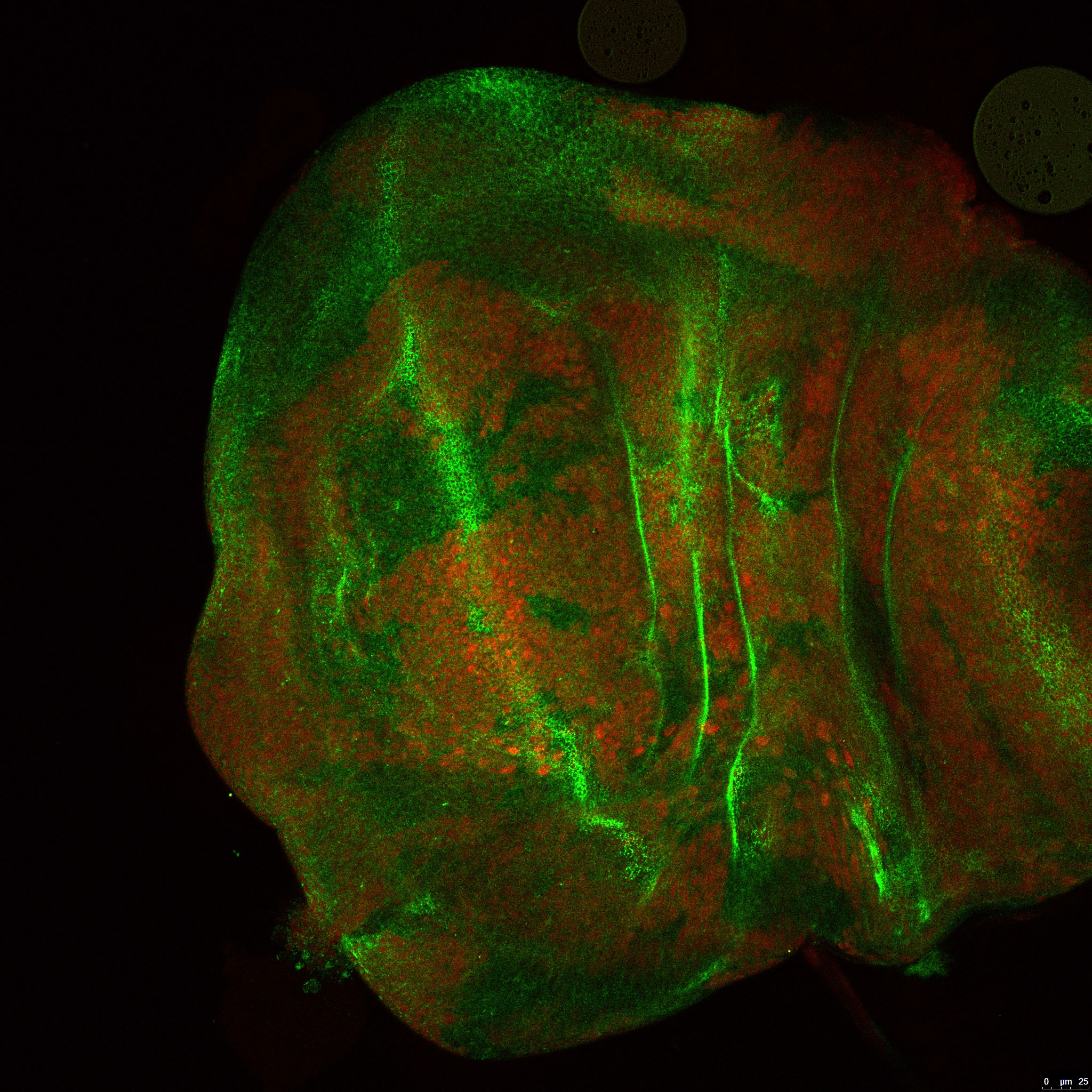

Supplement: Supplementary file 6 — Source data Fig. 4 [file 44319_2024_289_MOESM6_ESM.zip › Figure 4/F4C/F4C1 20210531 AP-1gama ExWg.lif_20210531 Ubx-Flp AP-1gama 19A ExWg -1 -1_z0.tif]

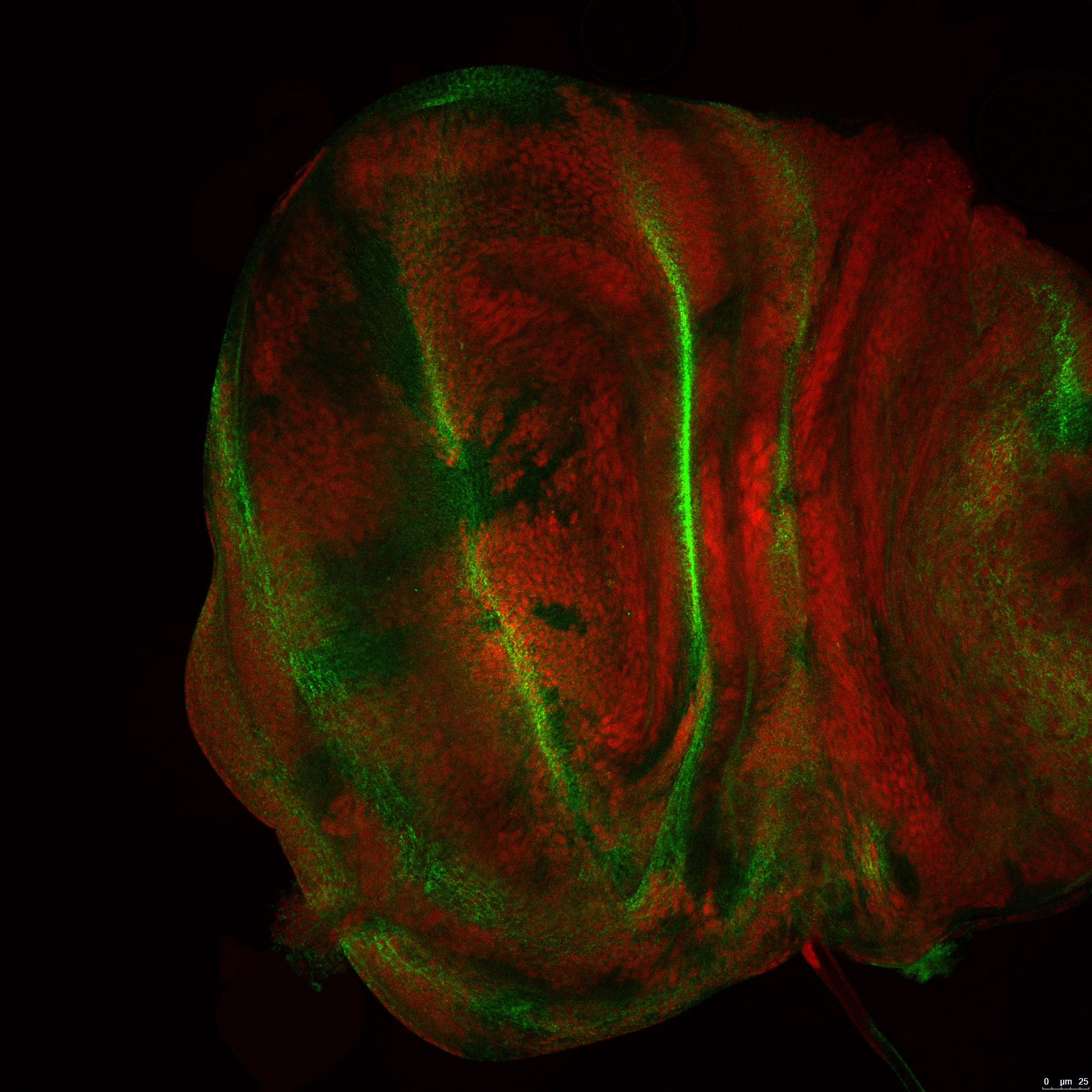

Supplement: Supplementary file 6 — Source data Fig. 4 [file 44319_2024_289_MOESM6_ESM.zip › Figure 4/F4C/F4C2 20210531 AP-1gama ExWg.lif_20210531 Ubx-Flp AP-1gama 19A ExWg -1 -2_z0.tif]

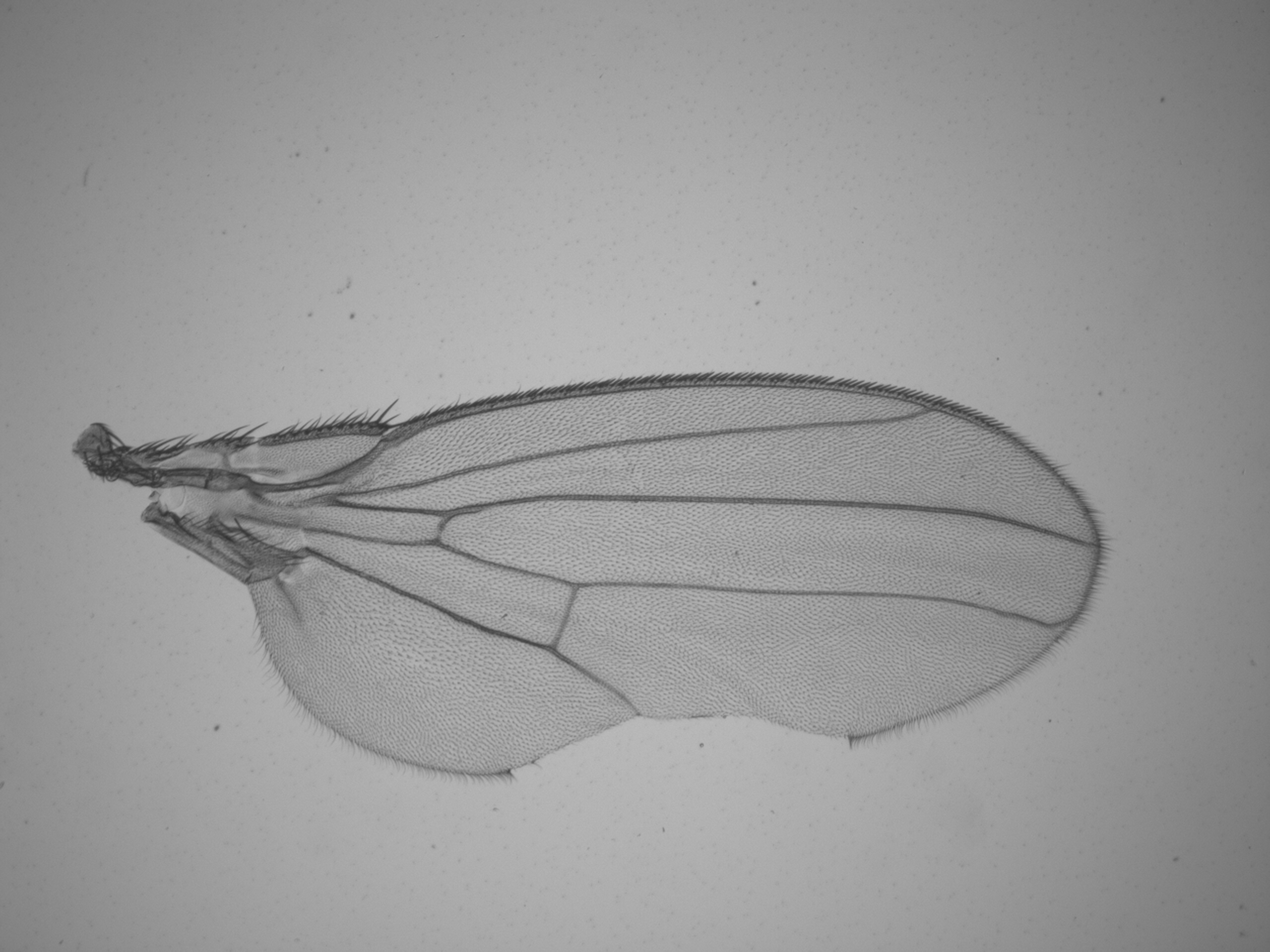

Supplement: Supplementary file 6 — Source data Fig. 4 [file 44319_2024_289_MOESM6_ESM.zip › Figure 4/F4E/F4E 230731 AP47 clones -1.tif]

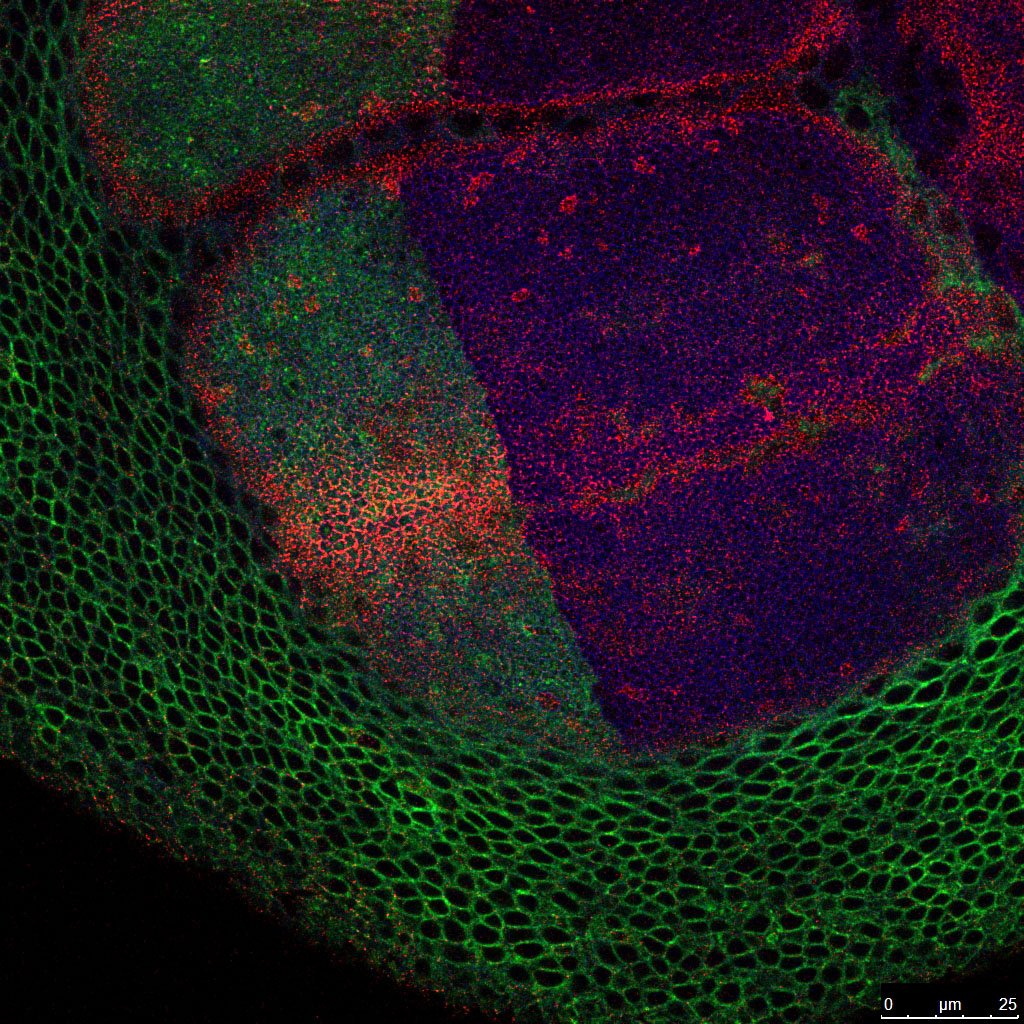

Supplement: Supplementary file 6 — Source data Fig. 4 [file 44319_2024_289_MOESM6_ESM.zip › Figure 4/F4F/F4F1 ts-Gal80 hh-G4-GFP AP-1gamma RNAi Exwg aPKC.tif]

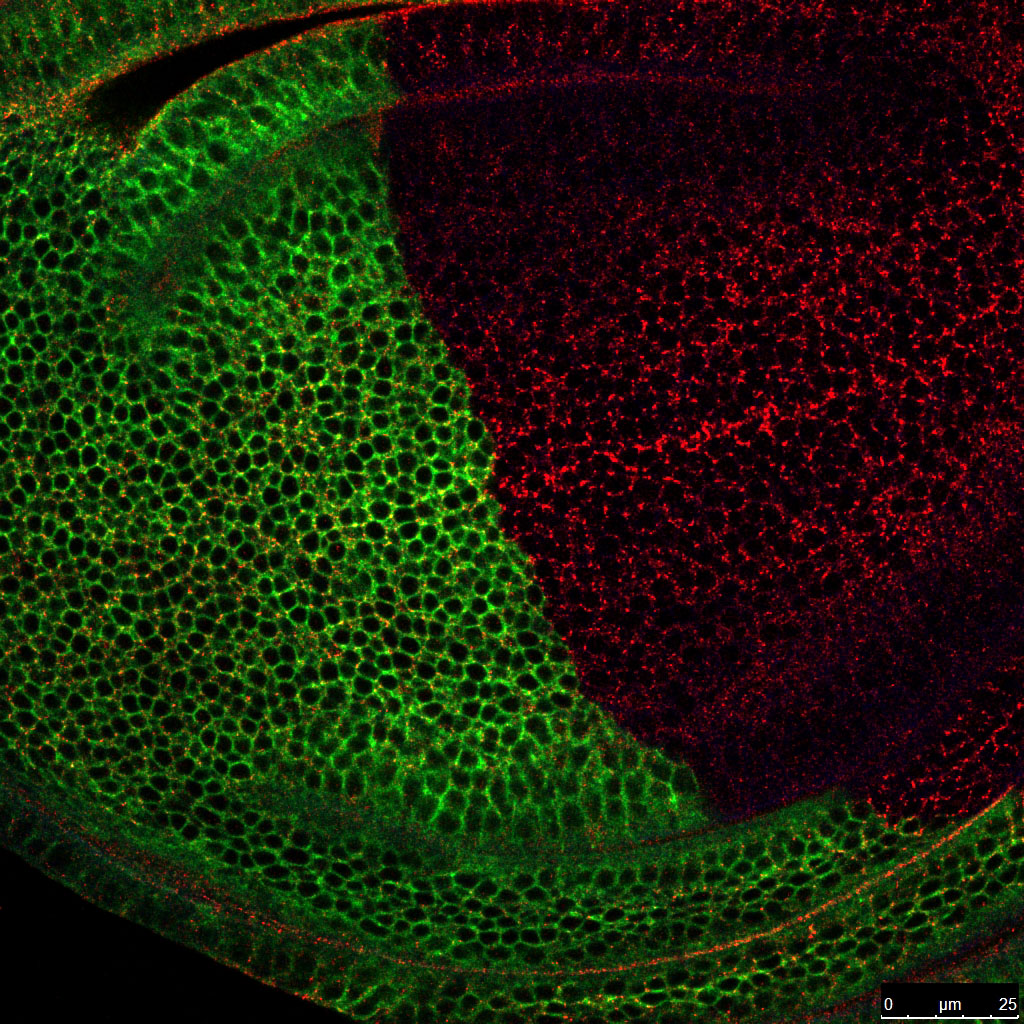

Supplement: Supplementary file 6 — Source data Fig. 4 [file 44319_2024_289_MOESM6_ESM.zip › Figure 4/F4F/F4F2 ts-Gal80 hh-G4-GFP AP-1gamma RNAi Exwg aPKC.tif]

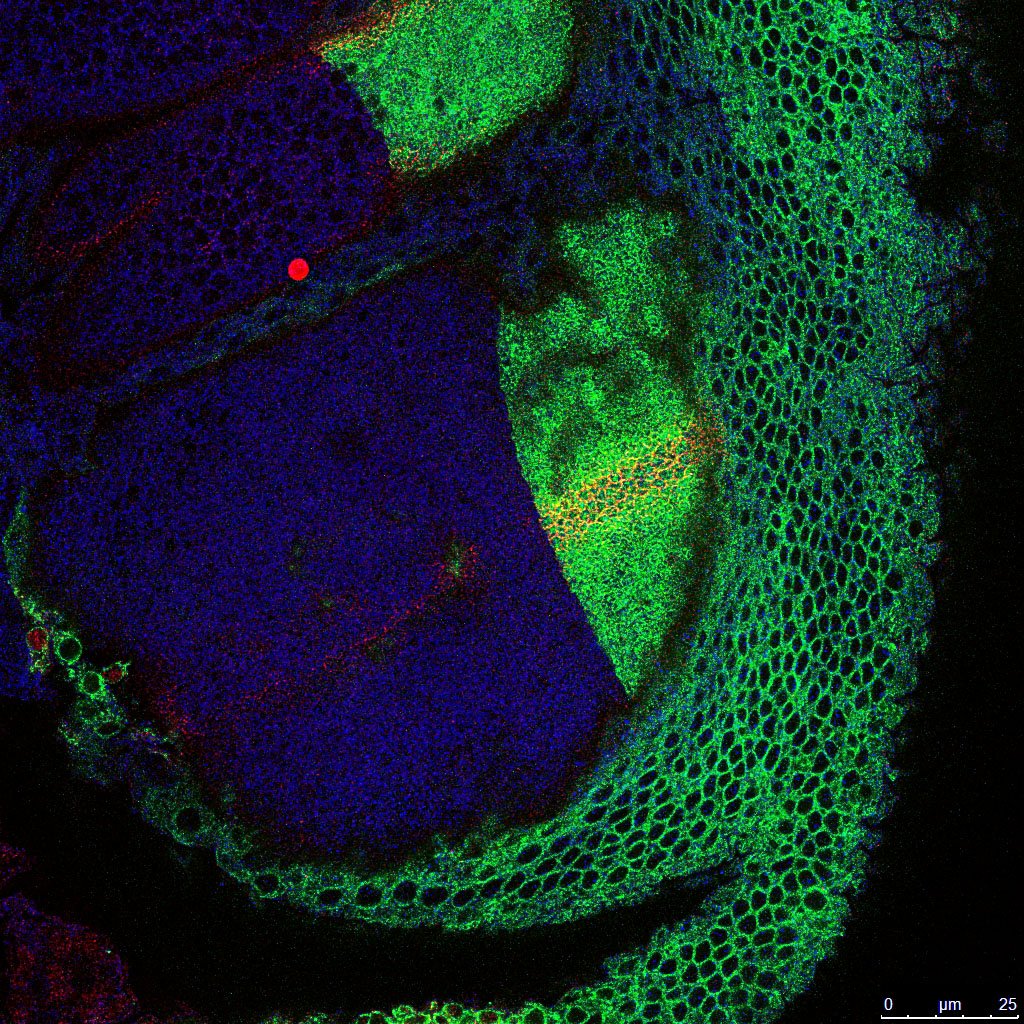

Supplement: Supplementary file 6 — Source data Fig. 4 [file 44319_2024_289_MOESM6_ESM.zip › Figure 4/F4G/F4G1 ts-Gal80 hh-G4-GFP AP-1gamma RNAi Th02340 Ehbp1RNAi Exwg Ehbp1.lif_Series003_Lng_global_SubVolume001.tif]

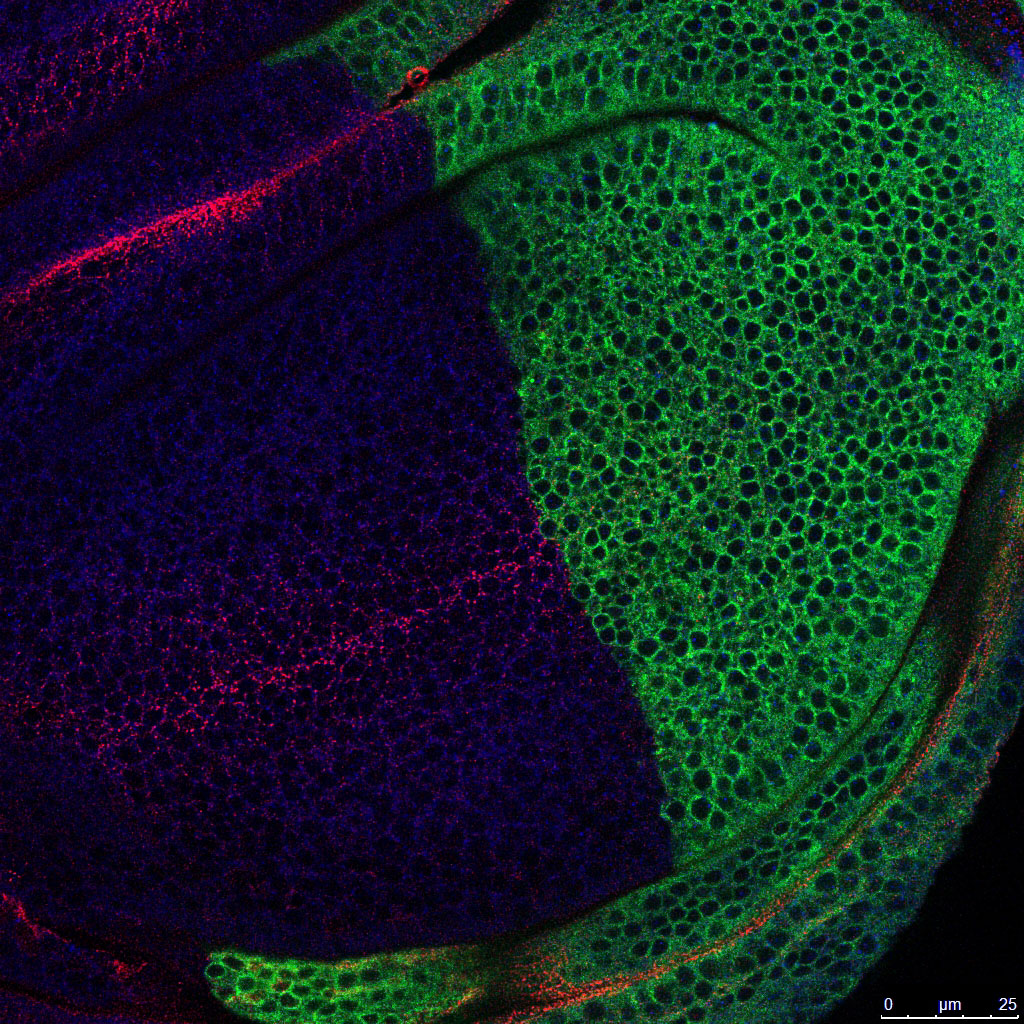

Supplement: Supplementary file 6 — Source data Fig. 4 [file 44319_2024_289_MOESM6_ESM.zip › Figure 4/F4G/F4G2 ts-Gal80 hh-G4-GFP AP-1gamma RNAi Th02340 Ehbp1RNAi Exwg Ehbp1.lif_Series003_Lng_global_SubVolume002.tif]

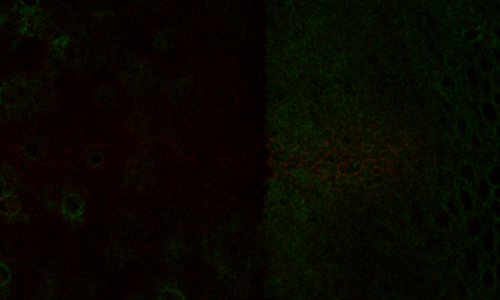

Supplement: Supplementary file 6 — Source data Fig. 4 [file 44319_2024_289_MOESM6_ESM.zip › Figure 4/F4H/F4H Images for statistical analysis - Apical ExWg/AP-1a├ RNAi, Apical ExWg/1 190912 ts-Gal80 hh-G4-GFP AP-1gamma RNAi 34h Exwg.tif]

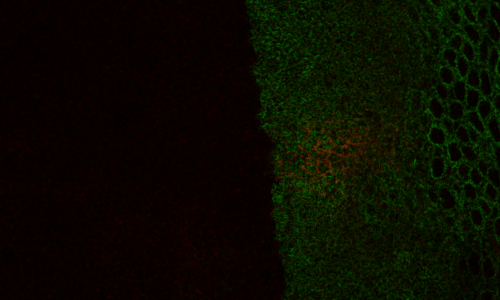

Supplement: Supplementary file 6 — Source data Fig. 4 [file 44319_2024_289_MOESM6_ESM.zip › Figure 4/F4H/F4H Images for statistical analysis - Apical ExWg/AP-1a├ RNAi, Apical ExWg/2 190912 ts-Gal80 hh-G4-GFP AP-1gamma RNAi 34h.tif]

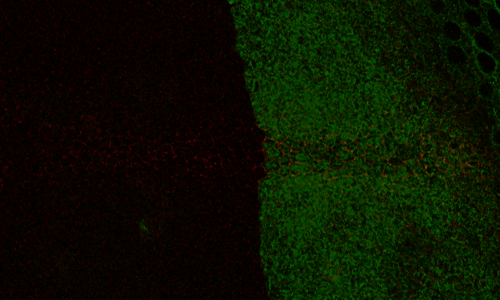

Supplement: Supplementary file 6 — Source data Fig. 4 [file 44319_2024_289_MOESM6_ESM.zip › Figure 4/F4H/F4H Images for statistical analysis - Apical ExWg/AP-1a├ RNAi, Apical ExWg/3 200516 ts-Gal80 hh-G4-GFP AP-1gamma RNAi exwg.tif]

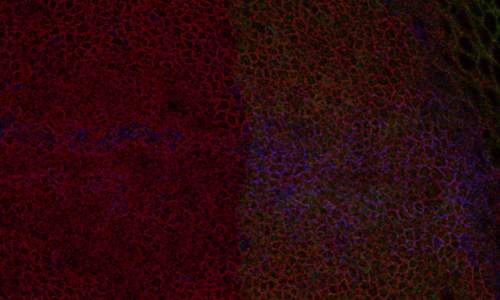

Supplement: Supplementary file 6 — Source data Fig. 4 [file 44319_2024_289_MOESM6_ESM.zip › Figure 4/F4H/F4H Images for statistical analysis - Apical ExWg/AP-1a├ RNAi, Apical ExWg/4 200820 ts-Gal80 hh-G4-GFP AP-1gamma RNAi Exwg aPKC.tif]

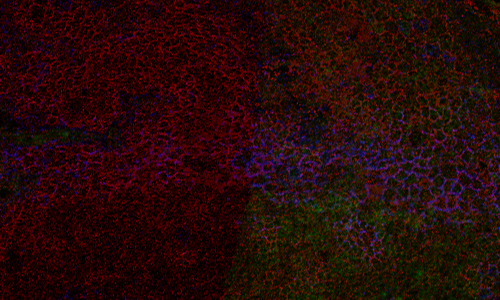

Supplement: Supplementary file 6 — Source data Fig. 4 [file 44319_2024_289_MOESM6_ESM.zip › Figure 4/F4H/F4H Images for statistical analysis - Apical ExWg/AP-1a├ RNAi, Apical ExWg/5 200820 ts-Gal80 hh-G4-GFP AP-1gamma RNAi Exwg aPKC.tif]

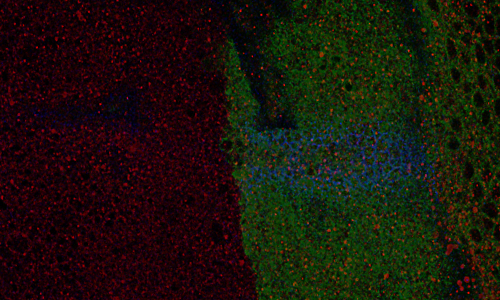

Supplement: Supplementary file 6 — Source data Fig. 4 [file 44319_2024_289_MOESM6_ESM.zip › Figure 4/F4H/F4H Images for statistical analysis - Apical ExWg/AP-1a├+Ehbp1 RNAi, Apical ExWg/200708 ts-Gal80 hh-G4-GFP AP-1gamma RNAi Th02340 Ehbp1 Exwg 2.tif]

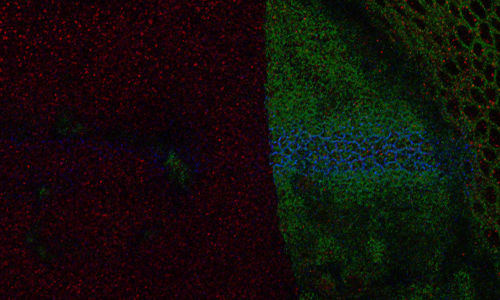

Supplement: Supplementary file 6 — Source data Fig. 4 [file 44319_2024_289_MOESM6_ESM.zip › Figure 4/F4H/F4H Images for statistical analysis - Apical ExWg/AP-1a├+Ehbp1 RNAi, Apical ExWg/200708 ts-Gal80 hh-G4-GFP AP-1gamma RNAi Th02340 Ehbp1 Exwg.tif]

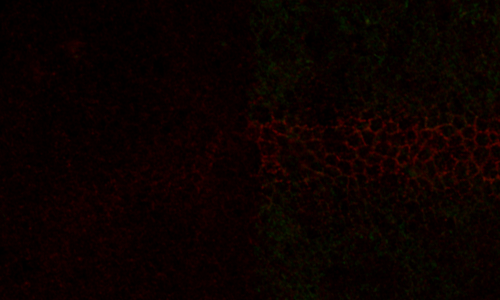

Supplement: Supplementary file 6 — Source data Fig. 4 [file 44319_2024_289_MOESM6_ESM.zip › Figure 4/F4H/F4H Images for statistical analysis - Apical ExWg/AP-1a├+Ehbp1 RNAi, Apical ExWg/240503 1 ts-Gal80 hh-G4-GFP AP-1gamma and Ehbp1 RNAi_Series003_8.tif]

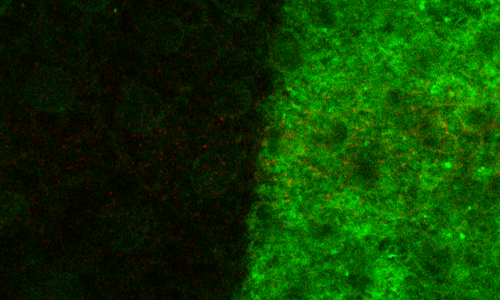

Supplement: Supplementary file 6 — Source data Fig. 4 [file 44319_2024_289_MOESM6_ESM.zip › Figure 4/F4H/F4H Images for statistical analysis - Apical ExWg/AP-1a├+Ehbp1 RNAi, Apical ExWg/240503 2 ts-Gal80 hh-G4-GFP AP-1gamma and Ehbp1 RNAi_Series002.tif]

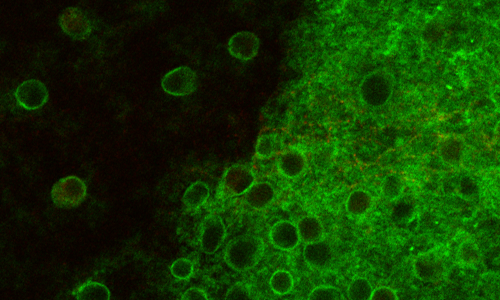

Supplement: Supplementary file 6 — Source data Fig. 4 [file 44319_2024_289_MOESM6_ESM.zip › Figure 4/F4H/F4H Images for statistical analysis - Apical ExWg/AP-1a├+Ehbp1 RNAi, Apical ExWg/240503 3 ts-Gal80 hh-G4-GFP AP-1gamma and Ehbp1 RNAi_Series004.tif]

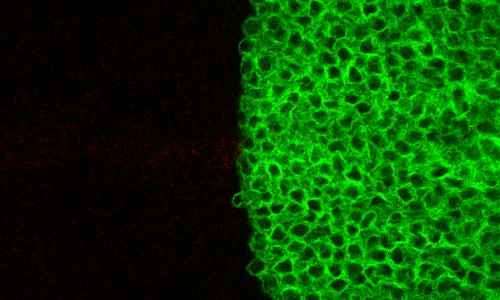

Supplement: Supplementary file 6 — Source data Fig. 4 [file 44319_2024_289_MOESM6_ESM.zip › Figure 4/F4H/F4H Images for statistical analysis - Apical ExWg/Ehbp1 RNAi, Apical ExWg/170111 hh-G4-GFP dEHBP1 ExWg 1-z .tif]

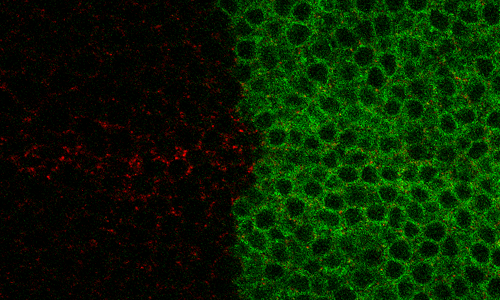

Supplement: Supplementary file 6 — Source data Fig. 4 [file 44319_2024_289_MOESM6_ESM.zip › Figure 4/F4H/F4H Images for statistical analysis - Apical ExWg/Ehbp1 RNAi, Apical ExWg/180227 ts-Gal80 hh-G4-GFP Th02340 ExWg.tif]

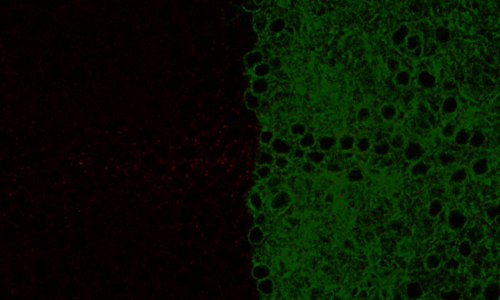

Supplement: Supplementary file 6 — Source data Fig. 4 [file 44319_2024_289_MOESM6_ESM.zip › Figure 4/F4H/F4H Images for statistical analysis - Apical ExWg/Ehbp1 RNAi, Apical ExWg/200708 ts-Gal80 hh-G4-GFP Th02340 exwg.tif]

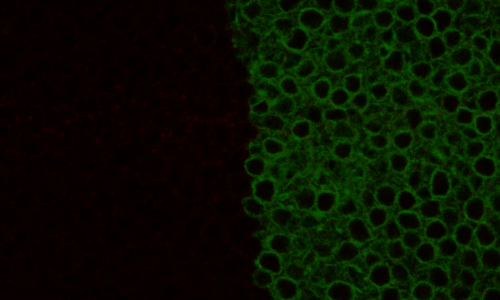

Supplement: Supplementary file 6 — Source data Fig. 4 [file 44319_2024_289_MOESM6_ESM.zip › Figure 4/F4H/F4H Images for statistical analysis - Apical ExWg/Ehbp1 RNAi, Apical ExWg/200713 ts-Gal80 hh-G4-GFP TH02340 Exwg 2.tif]

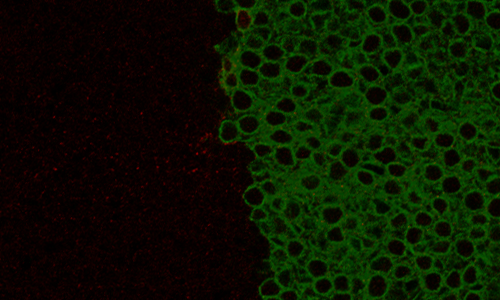

Supplement: Supplementary file 6 — Source data Fig. 4 [file 44319_2024_289_MOESM6_ESM.zip › Figure 4/F4H/F4H Images for statistical analysis - Apical ExWg/Ehbp1 RNAi, Apical ExWg/200713 ts-Gal80 hh-G4-GFP TH02340 Exwg.tif]

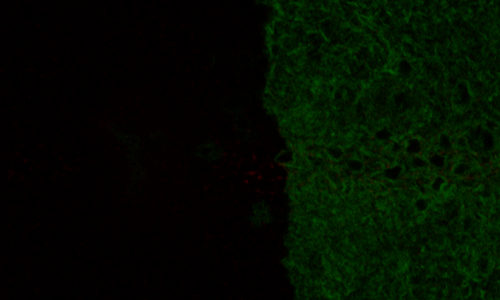

Supplement: Supplementary file 6 — Source data Fig. 4 [file 44319_2024_289_MOESM6_ESM.zip › Figure 4/F4H/F4H Images for statistical analysis - Apical ExWg/Ehbp1-wt-OE, Apical ExWg/Apical 1 hh-G4-GFP Ehbp1wt exwg_Series001_z012.tif]
