# Supplementary figures and images for: Ehbp1 orchestrates orderly sorting of Wnt/Wingless to the basolateral and apical cell membranes (part 2 of 3)
Source: EMBO Rep. 2024 Oct 14;25(11):5053–79. doi: 10.1038/s44319-024-00289-1 (PMC11549480; doi:10.1038/s44319-024-00289-1)

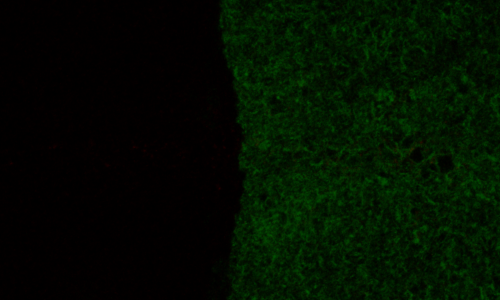

Supplement: Supplementary file 6 — Source data Fig. 4 [file 44319_2024_289_MOESM6_ESM.zip › Figure 4/F4H/F4H Images for statistical analysis - Apical ExWg/Ehbp1-wt-OE, Apical ExWg/Apical 2 hh-G4-GFP Ehbp1wt exwg_Series002_z018.tif]

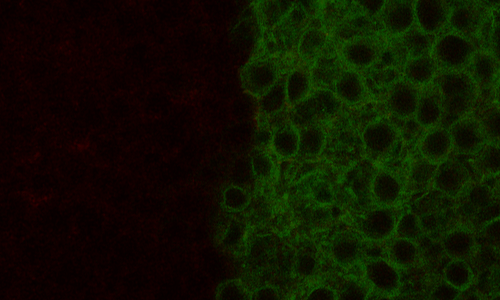

Supplement: Supplementary file 6 — Source data Fig. 4 [file 44319_2024_289_MOESM6_ESM.zip › Figure 4/F4H/F4H Images for statistical analysis - Apical ExWg/Ehbp1-wt-OE, Apical ExWg/Apical 3 20240503 hh-G4-GFP UAS-Ehbp1-wt ExWg_.tif]

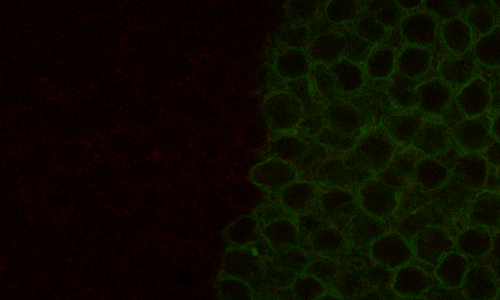

Supplement: Supplementary file 6 — Source data Fig. 4 [file 44319_2024_289_MOESM6_ESM.zip › Figure 4/F4H/F4H Images for statistical analysis - Apical ExWg/Ehbp1-wt-OE, Apical ExWg/Apical 4 20240503 hh-G4-GFP UAS-Ehbp1-wt ExWg.tif]

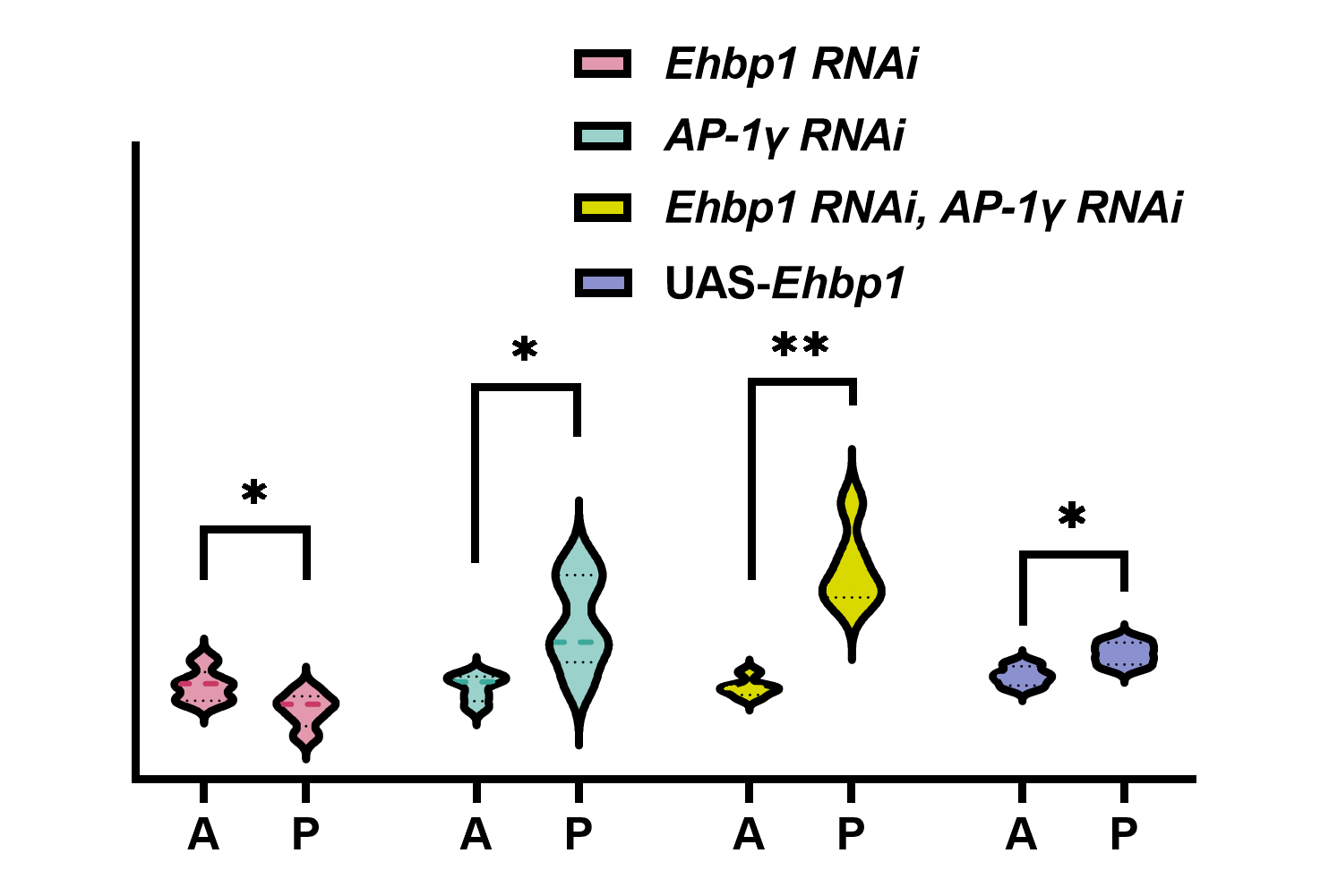

Supplement: Supplementary file 6 — Source data Fig. 4 [file 44319_2024_289_MOESM6_ESM.zip › Figure 4/F4H/Figure 4H Apical ExWg.tif]

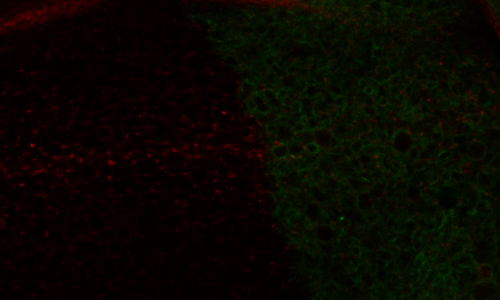

Supplement: Supplementary file 6 — Source data Fig. 4 [file 44319_2024_289_MOESM6_ESM.zip › Figure 4/F4I/F4I Images for statistical analysis - Basalateral ExWg/AP-1a├ RNAi, Basal ExWg/190912 ts-Gal80 hh-G4-GFP AP-1gamma RNAi 34h Exwg 1.tif]

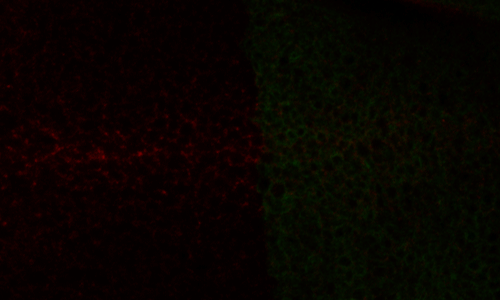

Supplement: Supplementary file 6 — Source data Fig. 4 [file 44319_2024_289_MOESM6_ESM.zip › Figure 4/F4I/F4I Images for statistical analysis - Basalateral ExWg/AP-1a├ RNAi, Basal ExWg/190912 ts-Gal80 hh-G4-GFP AP-1gamma RNAi 34h Exwg 2.tif]

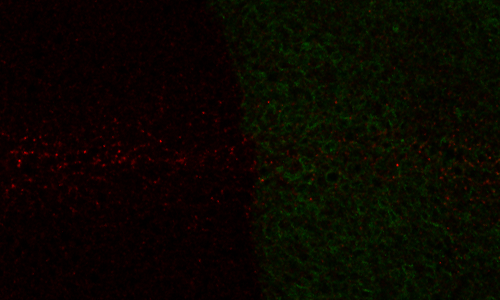

Supplement: Supplementary file 6 — Source data Fig. 4 [file 44319_2024_289_MOESM6_ESM.zip › Figure 4/F4I/F4I Images for statistical analysis - Basalateral ExWg/AP-1a├ RNAi, Basal ExWg/200516 ts-Gal80 hh-G4-GFP AP-1gamma RNAi exwg 3.tif]

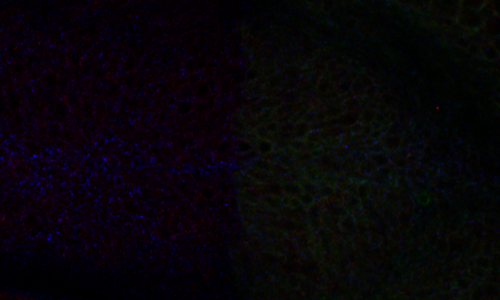

Supplement: Supplementary file 6 — Source data Fig. 4 [file 44319_2024_289_MOESM6_ESM.zip › Figure 4/F4I/F4I Images for statistical analysis - Basalateral ExWg/AP-1a├ RNAi, Basal ExWg/200820 ts-Gal80 hh-G4-GFP AP-1gamma RNAi Exwg aPKC_ 4.tif]

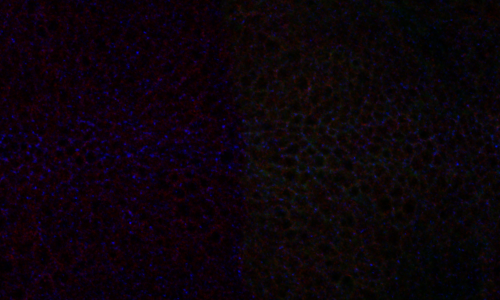

Supplement: Supplementary file 6 — Source data Fig. 4 [file 44319_2024_289_MOESM6_ESM.zip › Figure 4/F4I/F4I Images for statistical analysis - Basalateral ExWg/AP-1a├ RNAi, Basal ExWg/200820 ts-Gal80 hh-G4-GFP AP-1gamma RNAi Exwg aPKC_5.tif]

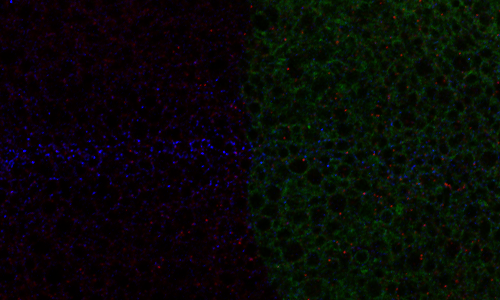

Supplement: Supplementary file 6 — Source data Fig. 4 [file 44319_2024_289_MOESM6_ESM.zip › Figure 4/F4I/F4I Images for statistical analysis - Basalateral ExWg/AP-1a├+Ehbp1 RNAi, Basal ExWg/200708 ts-Gal80 hh-G4-GFP double RNAi Exwg 1.tif]

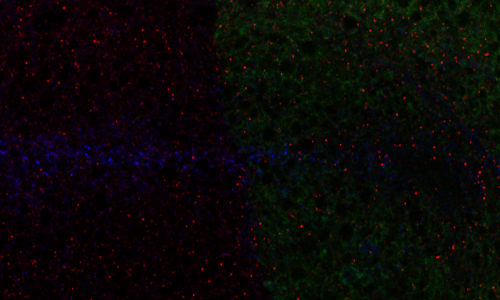

Supplement: Supplementary file 6 — Source data Fig. 4 [file 44319_2024_289_MOESM6_ESM.zip › Figure 4/F4I/F4I Images for statistical analysis - Basalateral ExWg/AP-1a├+Ehbp1 RNAi, Basal ExWg/200708 ts-Gal80 hh-G4-GFP double RNAi Exwg 2.tif]

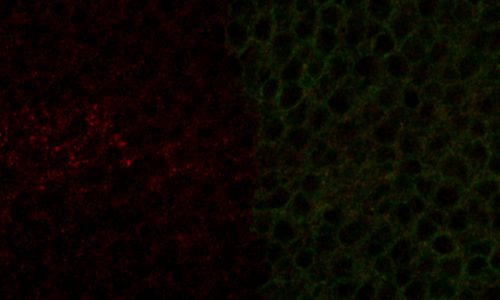

Supplement: Supplementary file 6 — Source data Fig. 4 [file 44319_2024_289_MOESM6_ESM.zip › Figure 4/F4I/F4I Images for statistical analysis - Basalateral ExWg/AP-1a├+Ehbp1 RNAi, Basal ExWg/240503 ts-Gal80 hh-G4-GFP double RNAi Exwg 3.tif]

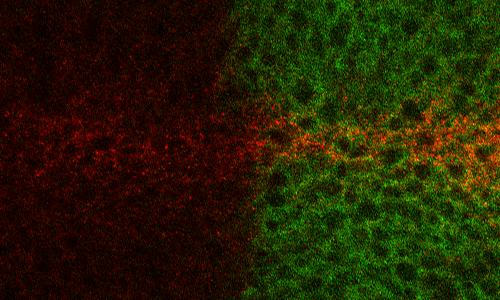

Supplement: Supplementary file 6 — Source data Fig. 4 [file 44319_2024_289_MOESM6_ESM.zip › Figure 4/F4I/F4I Images for statistical analysis - Basalateral ExWg/Ehbp1 RNAi, Basal ExWg/180227 ts-Gal80 hh-G4-GFP Th02340 ExWg_1.tif]

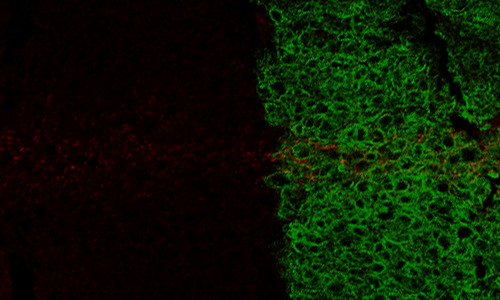

Supplement: Supplementary file 6 — Source data Fig. 4 [file 44319_2024_289_MOESM6_ESM.zip › Figure 4/F4I/F4I Images for statistical analysis - Basalateral ExWg/Ehbp1 RNAi, Basal ExWg/190912 ts-Gal80 hh-G4-GFP Th02340 34h Exwg_2.tif]

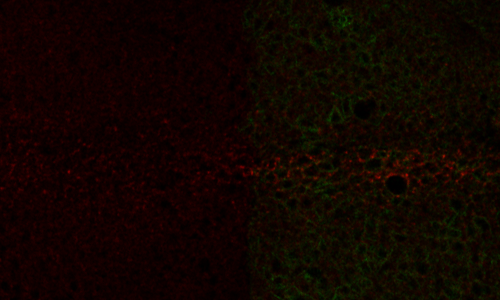

Supplement: Supplementary file 6 — Source data Fig. 4 [file 44319_2024_289_MOESM6_ESM.zip › Figure 4/F4I/F4I Images for statistical analysis - Basalateral ExWg/Ehbp1 RNAi, Basal ExWg/200202 ts-Gal80 hh-G4-GFP Th02340 Exwg_3.tif]

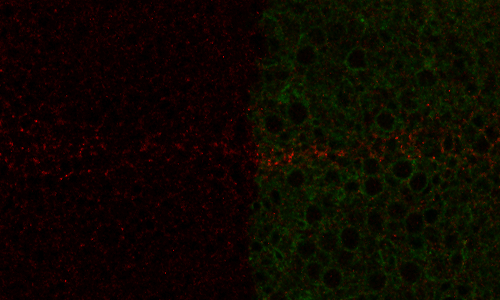

Supplement: Supplementary file 6 — Source data Fig. 4 [file 44319_2024_289_MOESM6_ESM.zip › Figure 4/F4I/F4I Images for statistical analysis - Basalateral ExWg/Ehbp1 RNAi, Basal ExWg/200708 ts-Gal80 hh-G4-GFP Th02340 exwg_4.tif]

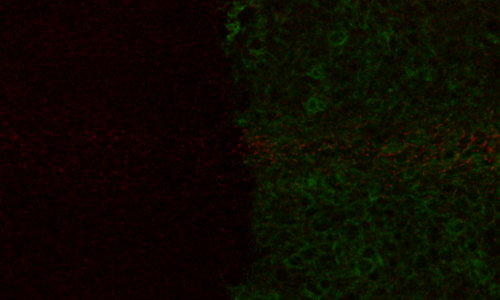

Supplement: Supplementary file 6 — Source data Fig. 4 [file 44319_2024_289_MOESM6_ESM.zip › Figure 4/F4I/F4I Images for statistical analysis - Basalateral ExWg/Ehbp1 RNAi, Basal ExWg/200713 ts-Gal80 hh-G4-GFP TH02340 Exwg_5.tif]

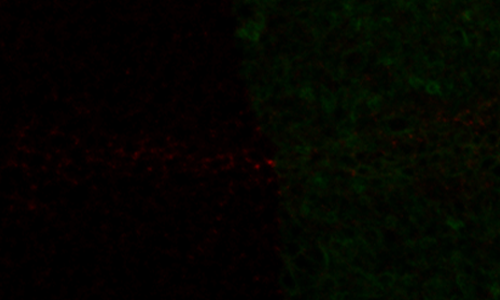

Supplement: Supplementary file 6 — Source data Fig. 4 [file 44319_2024_289_MOESM6_ESM.zip › Figure 4/F4I/F4I Images for statistical analysis - Basalateral ExWg/Ehbp1-wt-OE, Basal ExWg/Basal 1 20220507 hh-G4-GFP Ehbp1wt exwg_Series001_z139.tif]

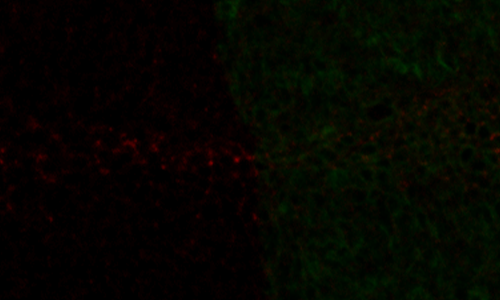

Supplement: Supplementary file 6 — Source data Fig. 4 [file 44319_2024_289_MOESM6_ESM.zip › Figure 4/F4I/F4I Images for statistical analysis - Basalateral ExWg/Ehbp1-wt-OE, Basal ExWg/Basal 2 20230806 hh-G4-GFP Ehbp1wt exwg_Series001_z0132.tif]

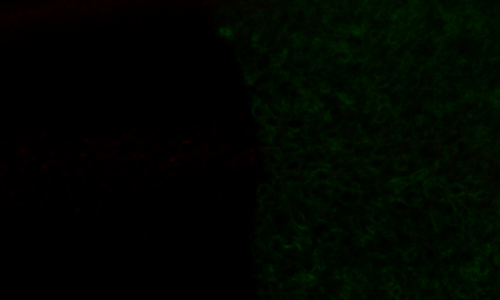

Supplement: Supplementary file 6 — Source data Fig. 4 [file 44319_2024_289_MOESM6_ESM.zip › Figure 4/F4I/F4I Images for statistical analysis - Basalateral ExWg/Ehbp1-wt-OE, Basal ExWg/Basal 3 20230806 hh-G4-GFP Ehbp1wt exwg_Series002_z155.tif]

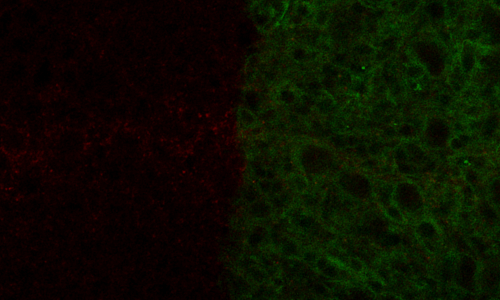

Supplement: Supplementary file 6 — Source data Fig. 4 [file 44319_2024_289_MOESM6_ESM.zip › Figure 4/F4I/F4I Images for statistical analysis - Basalateral ExWg/Ehbp1-wt-OE, Basal ExWg/Basal 4 20240503 ts-Gal80 hh-G4-GFP UAS-Ehbp1-wt ExWg_1.tif]

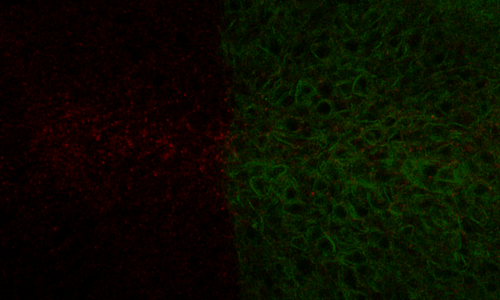

Supplement: Supplementary file 6 — Source data Fig. 4 [file 44319_2024_289_MOESM6_ESM.zip › Figure 4/F4I/F4I Images for statistical analysis - Basalateral ExWg/Ehbp1-wt-OE, Basal ExWg/Basal 5 20240503 ts-Gal80 hh-G4-GFP UAS-Ehbp1-wt ExWg_3.tif]

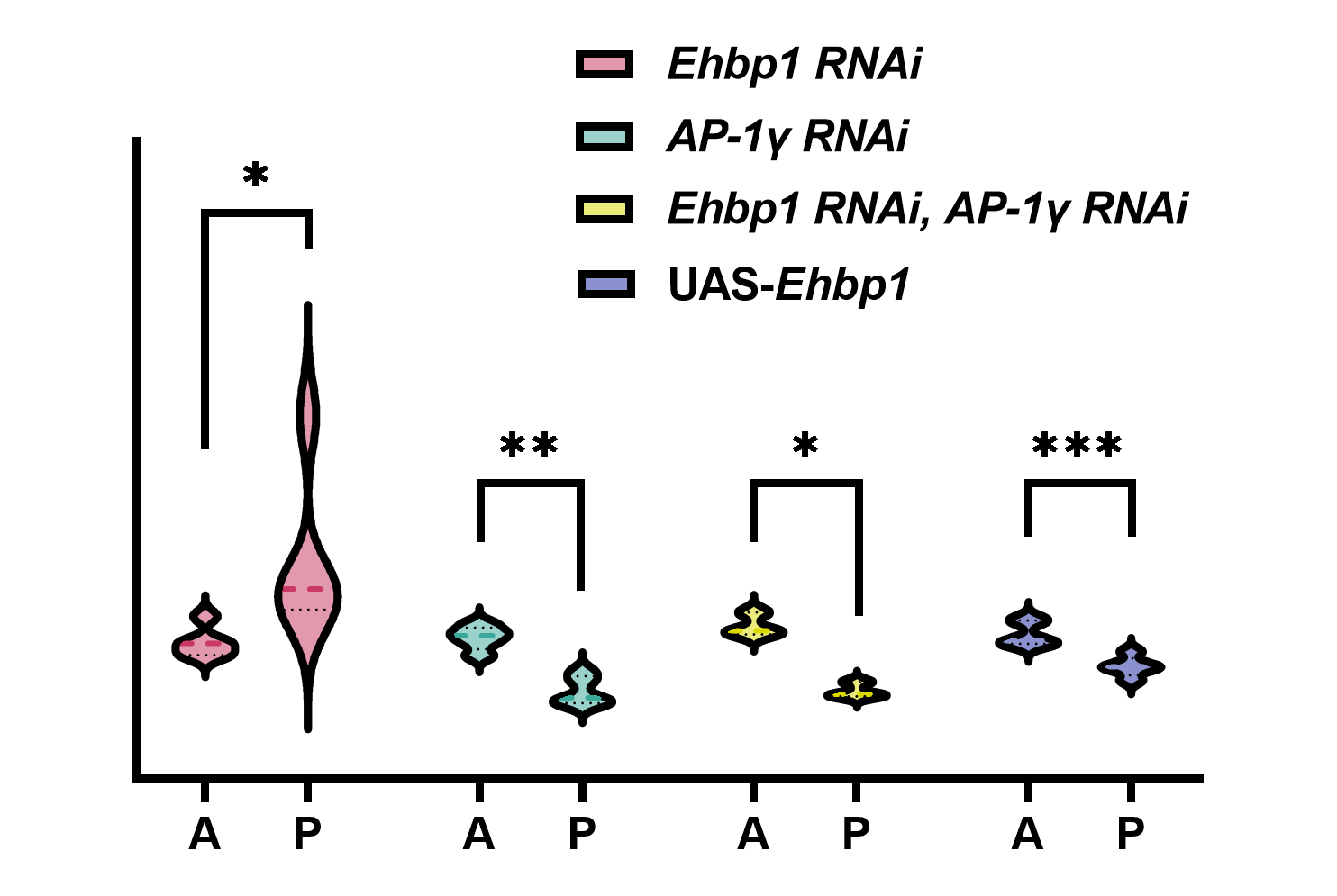

Supplement: Supplementary file 6 — Source data Fig. 4 [file 44319_2024_289_MOESM6_ESM.zip › Figure 4/F4I/Figure 4I Basalateral ExWg.tif]

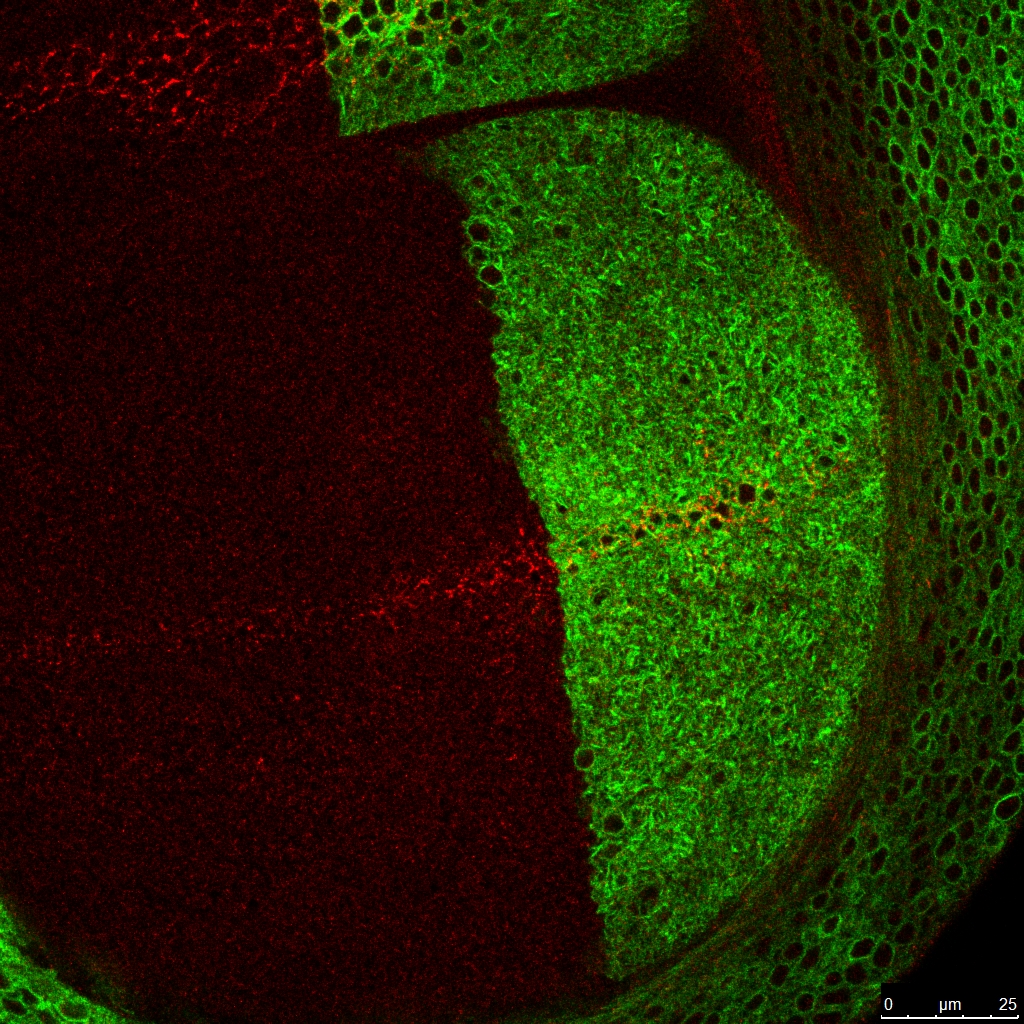

Supplement: Supplementary file 7 — Source data Fig. 5 [file 44319_2024_289_MOESM7_ESM.zip › Figure 5/F5A/F5A1 hh-G4-GFP Ehbp1wt exwg.lif_Series002_Lng_adaptive_SubVolume023.tif]

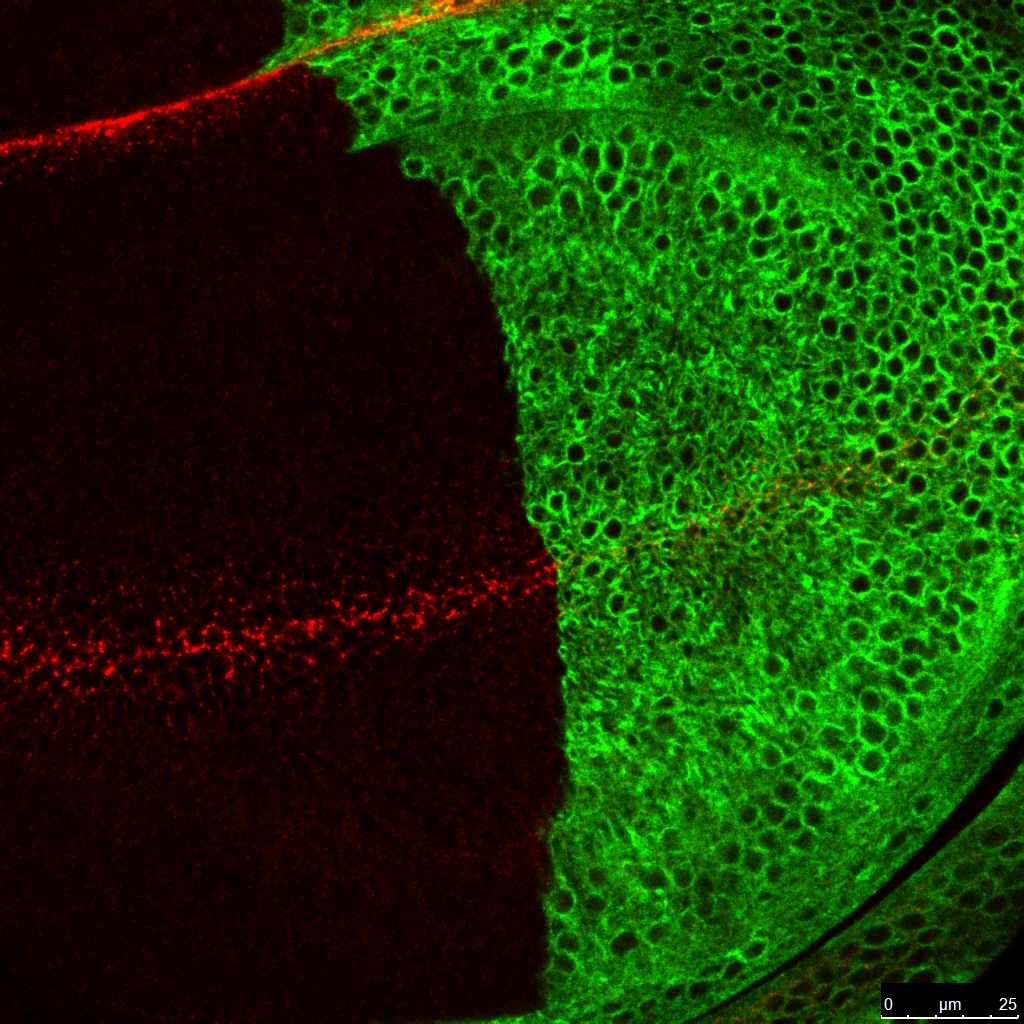

Supplement: Supplementary file 7 — Source data Fig. 5 [file 44319_2024_289_MOESM7_ESM.zip › Figure 5/F5A/F5A2 hh-G4-GFP Ehbp1wt exwg.lif_Series002_Lng_adaptive_SubVolume001.tif]

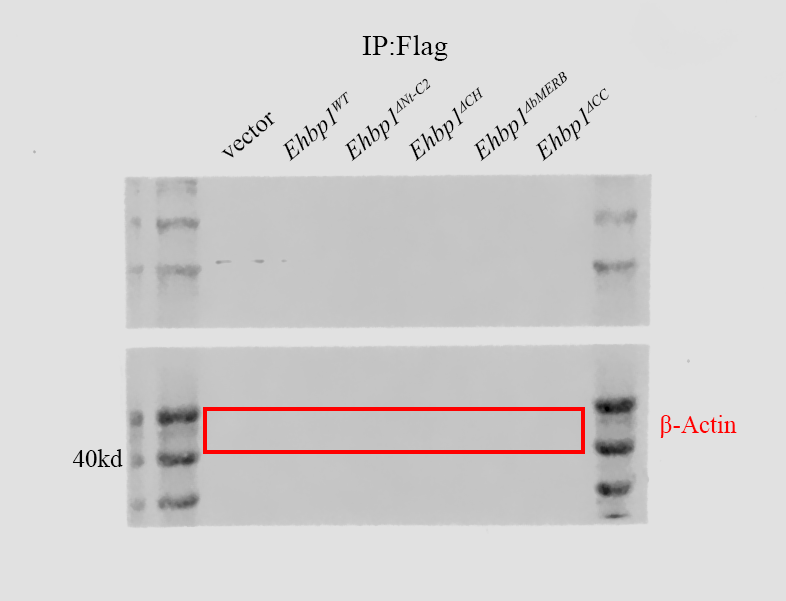

Supplement: Supplementary file 7 — Source data Fig. 5 [file 44319_2024_289_MOESM7_ESM.zip › Figure 5/F5D/F5D 20211103 Ehbp1-HA AP-1r-Flag hs-wls-HA IP-Flag anti-Actin.tif]

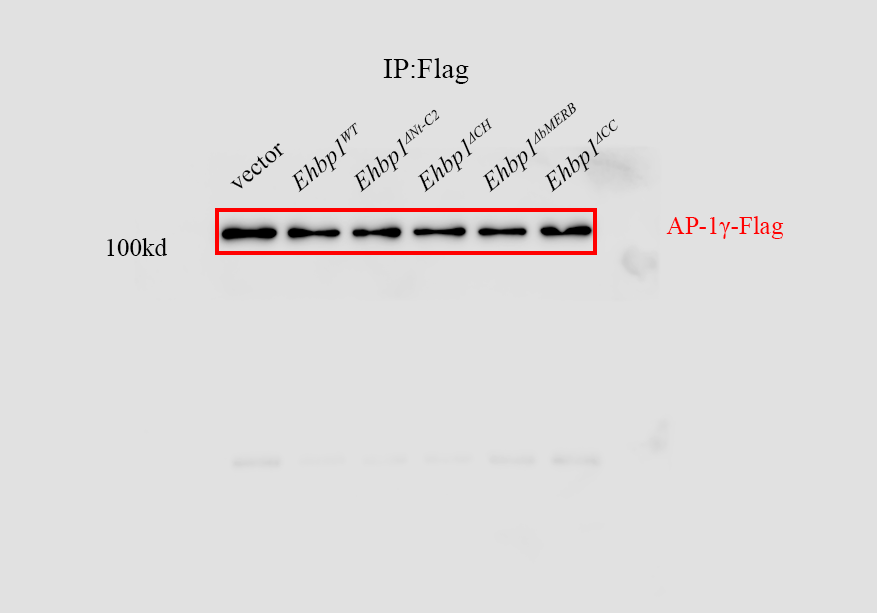

Supplement: Supplementary file 7 — Source data Fig. 5 [file 44319_2024_289_MOESM7_ESM.zip › Figure 5/F5D/F5D 20211103 Ehbp1-HA AP-1r-Flag hs-wls-HA IP-Flag anti-Flag.tif]

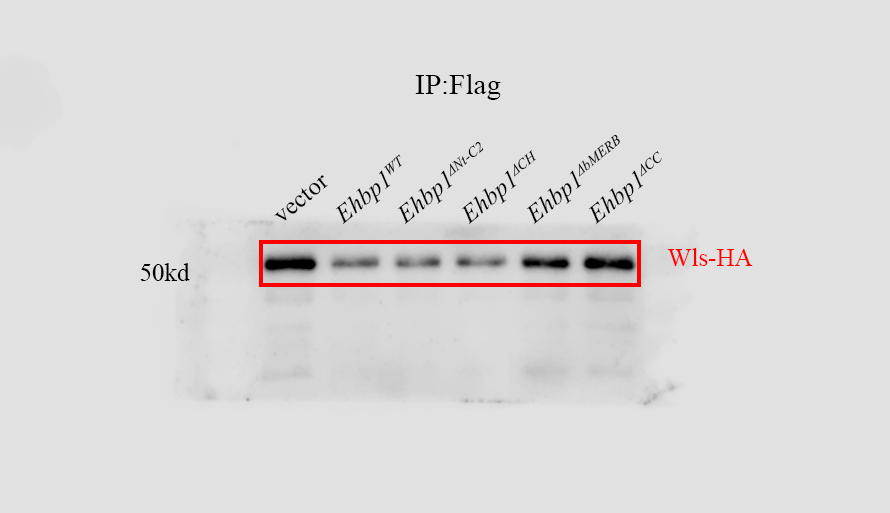

Supplement: Supplementary file 7 — Source data Fig. 5 [file 44319_2024_289_MOESM7_ESM.zip › Figure 5/F5D/F5D 20211103 Ehbp1-HA AP-1r-Flag hs-wls-HA IP-Flag anti-HA.tif]

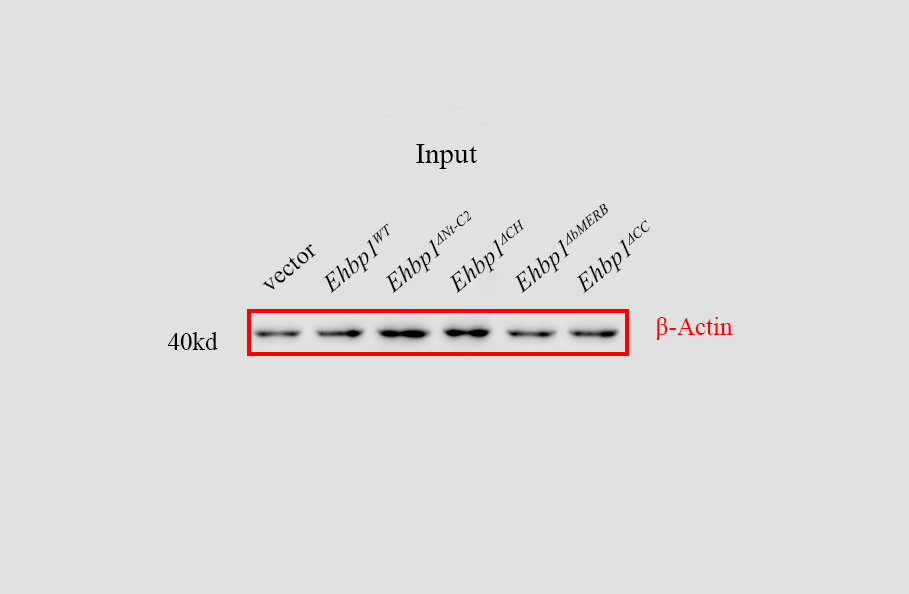

Supplement: Supplementary file 7 — Source data Fig. 5 [file 44319_2024_289_MOESM7_ESM.zip › Figure 5/F5D/F5D 20211103 Ehbp1-HA AP-1r-Flag hs-wls-HA Input anti-Actin.tif]

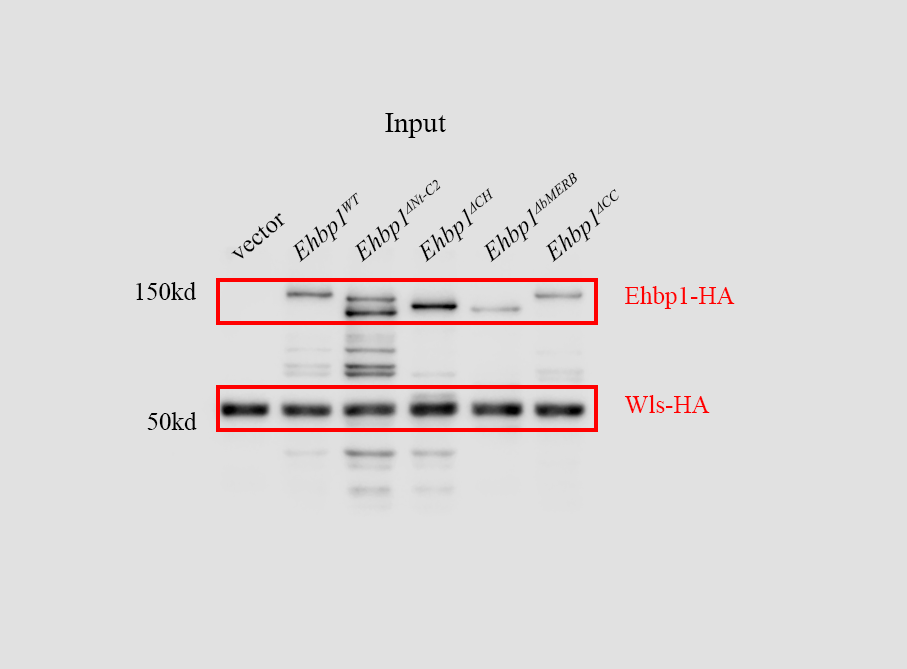

Supplement: Supplementary file 7 — Source data Fig. 5 [file 44319_2024_289_MOESM7_ESM.zip › Figure 5/F5D/F5D 20211103 Ehbp1-HA AP-1r-Flag hs-wls-HA Input anti-HA.tif]

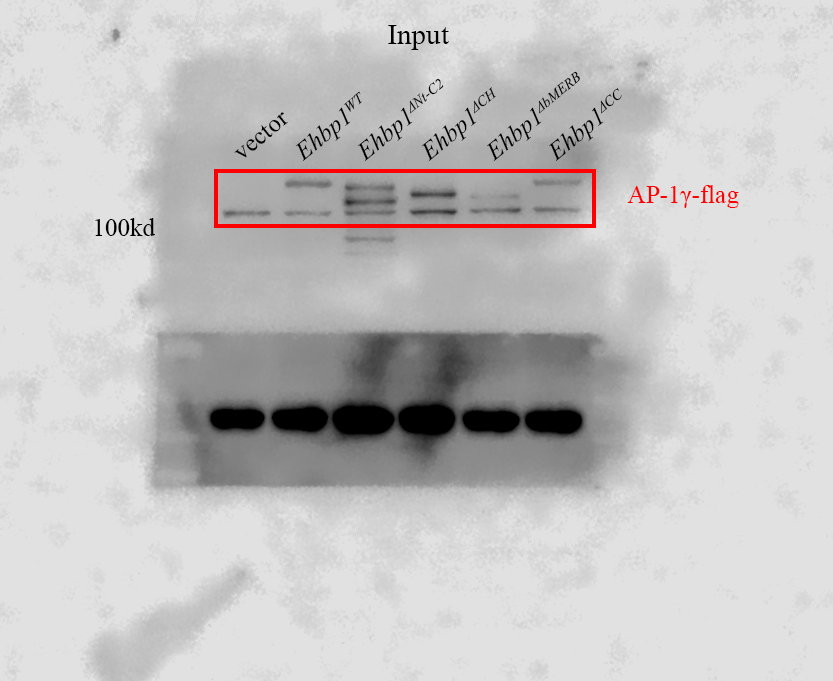

Supplement: Supplementary file 7 — Source data Fig. 5 [file 44319_2024_289_MOESM7_ESM.zip › Figure 5/F5D/F5D 20211103 Ehbp1-HA AP-1r-Flag hs-wls-HA Input anti-flag.tif]

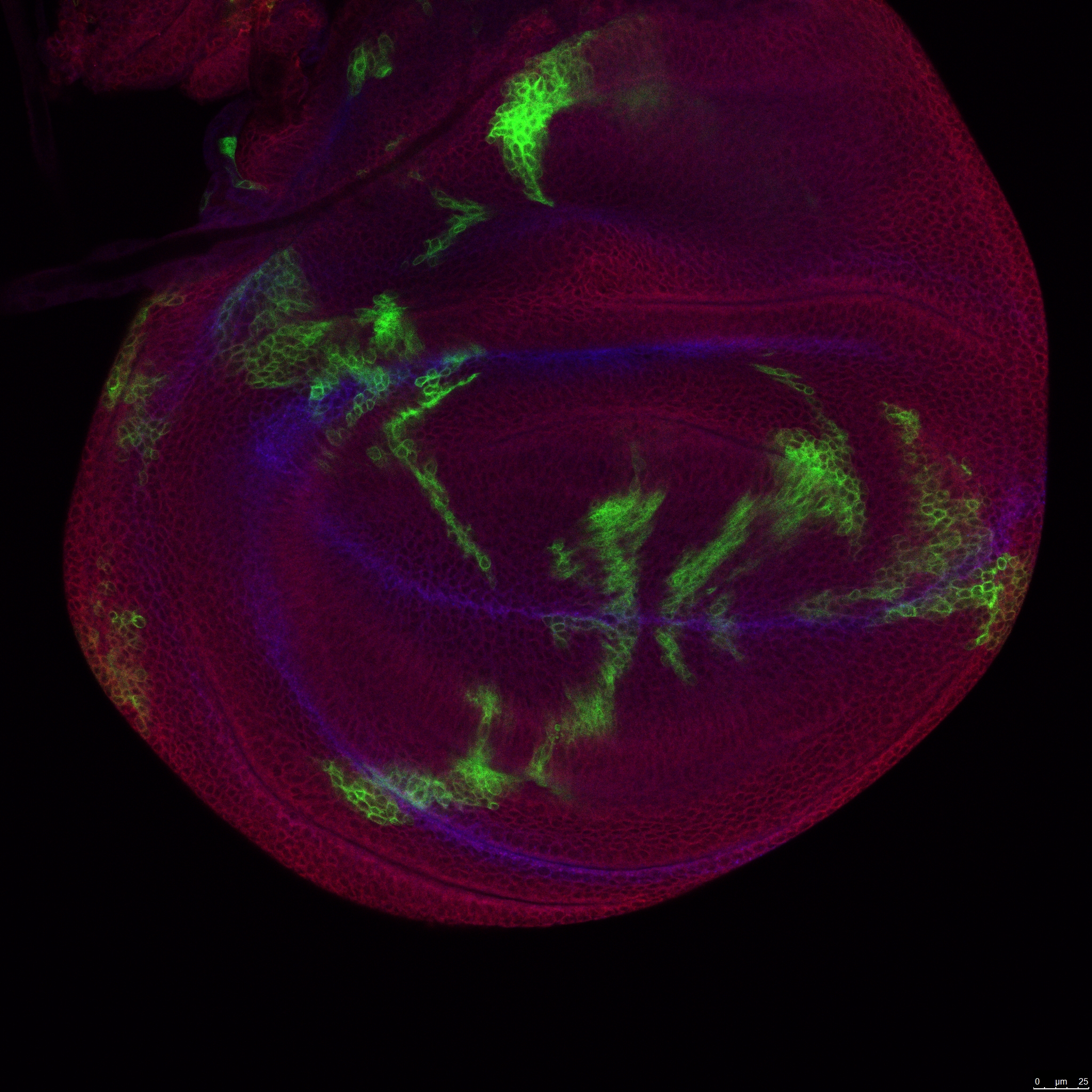

Supplement: Supplementary file 7 — Source data Fig. 5 [file 44319_2024_289_MOESM7_ESM.zip › Figure 5/F5E/F5E 20210615 Ehbp1 mutant Wg Wls.lif_20210615 MACRM 42D A28 WT Wg Wls -1 -z_SubVolume001.tif]

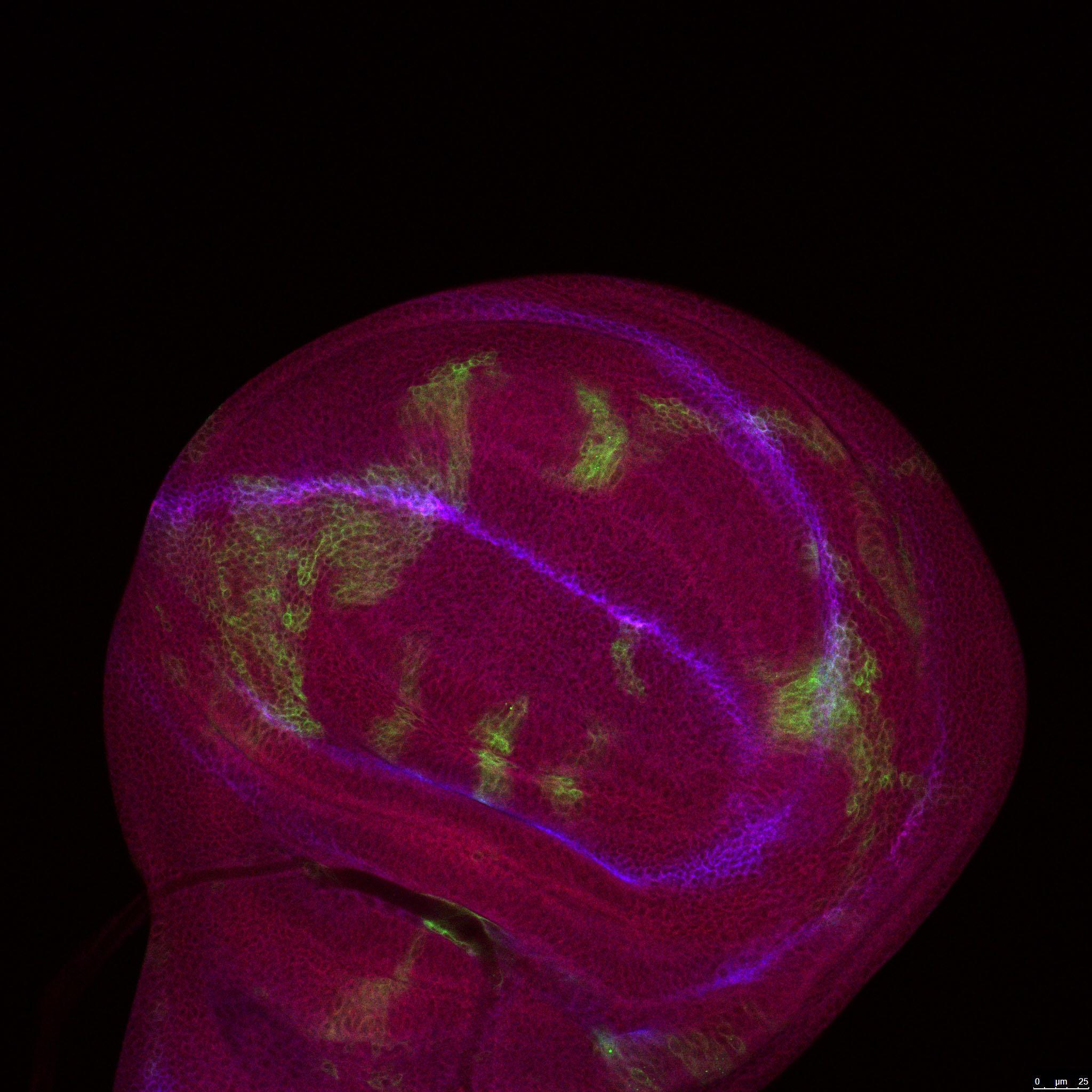

Supplement: Supplementary file 7 — Source data Fig. 5 [file 44319_2024_289_MOESM7_ESM.zip › Figure 5/F5F/F5F 20210615 Ehbp1 mutant Wg Wls.lif_20210615 MACRM 42D A28 dB Wg Wls -2_z0.tif]

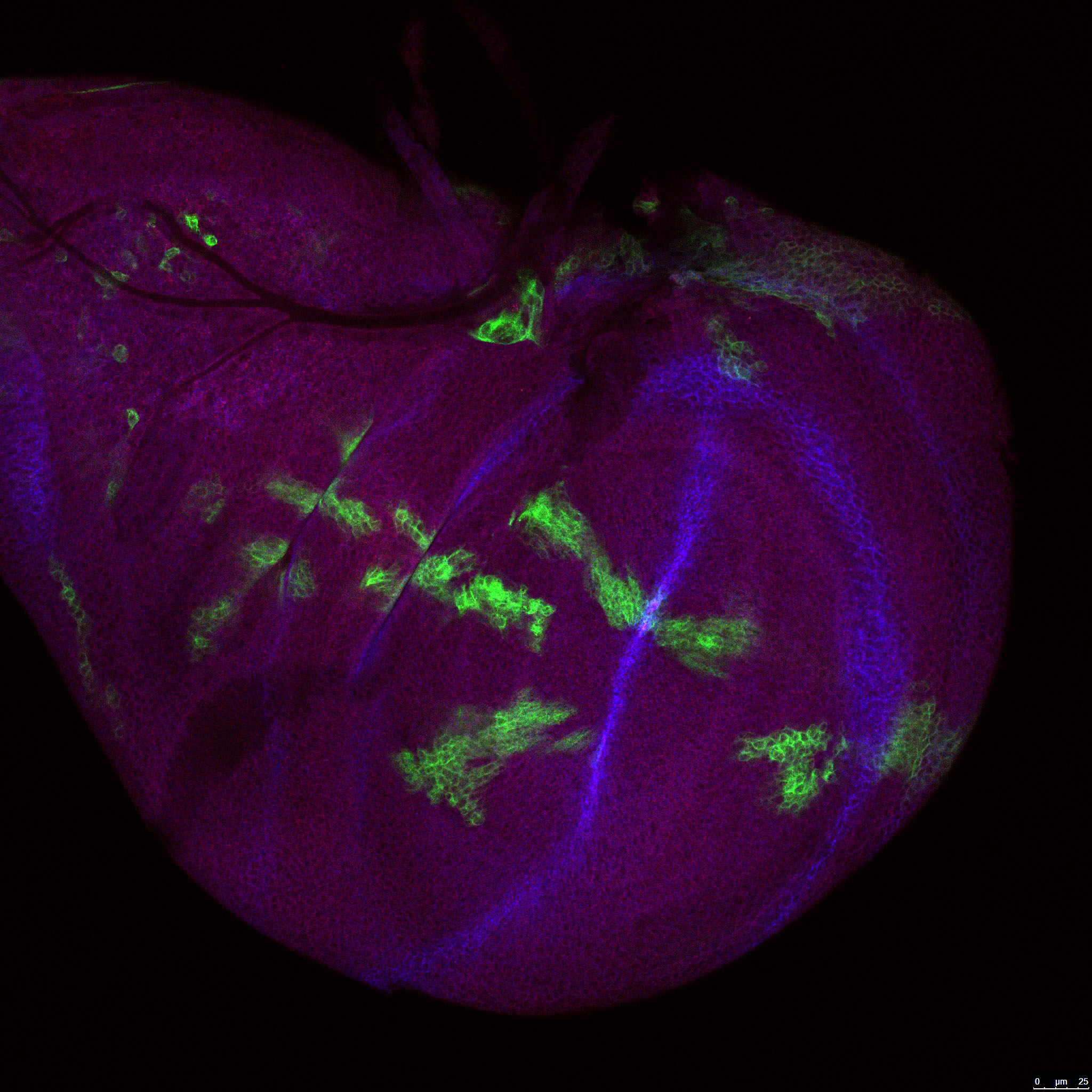

Supplement: Supplementary file 7 — Source data Fig. 5 [file 44319_2024_289_MOESM7_ESM.zip › Figure 5/F5G/F5G 20210615 Ehbp1 mutant Wg Wls.lif_20210615 MACRM 42D A28 dCC Wg Wls -2 -2_z0.tif]

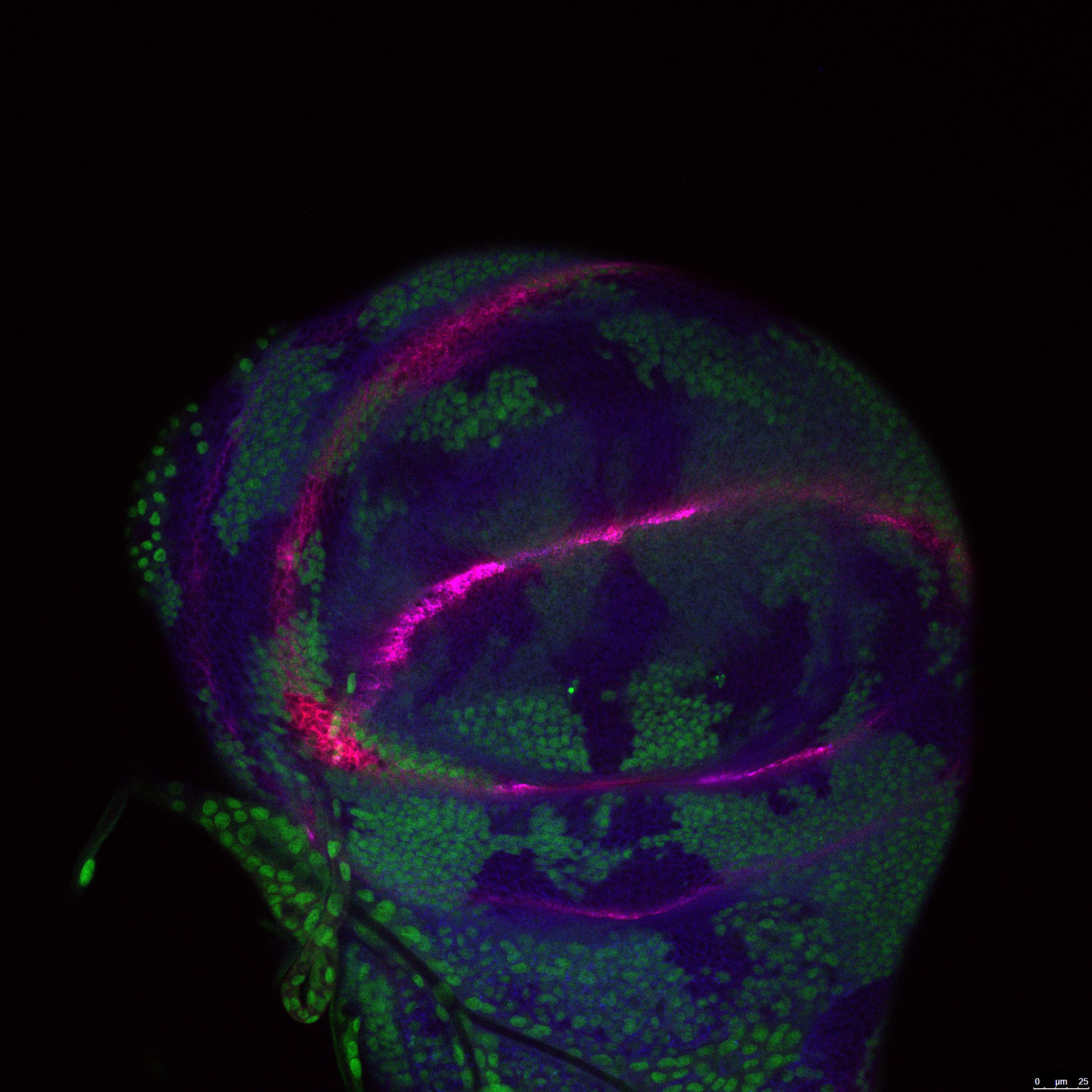

Supplement: Supplementary file 7 — Source data Fig. 5 [file 44319_2024_289_MOESM7_ESM.zip › Figure 5/F5H/F5H 20220923 Ehbp1 DeltaCC Wg Wls.lif_20220926 Ehbp1 DeltaCC Wls Wg -3 -z_z01.jpg]

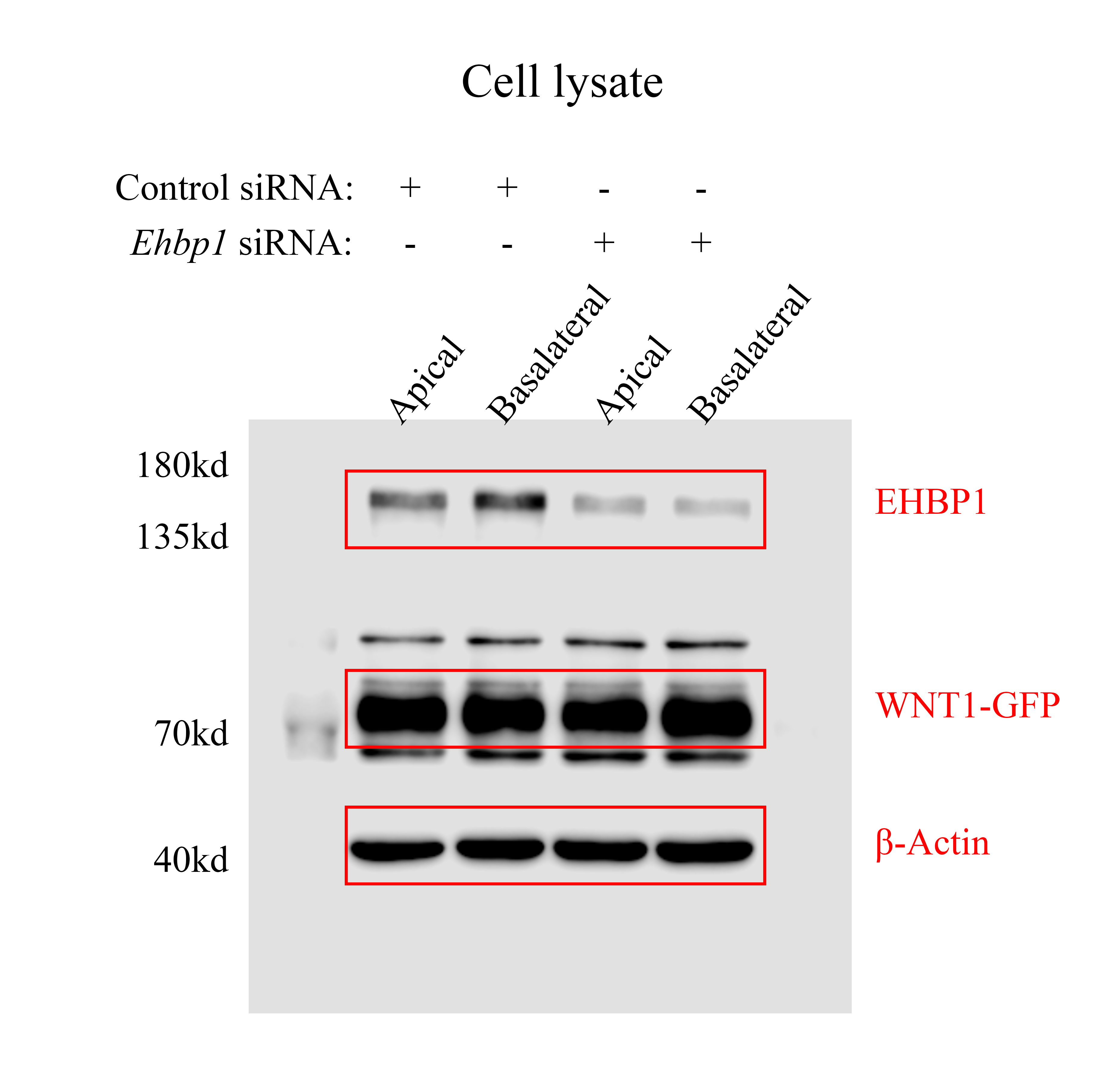

Supplement: Supplementary file 8 — Source data Fig. 6 [file 44319_2024_289_MOESM8_ESM.zip › Figure 6/F6A/F6A 20230926 MDCK transwell - input - Ehbp1 GFP actin - strong exposure.tif]

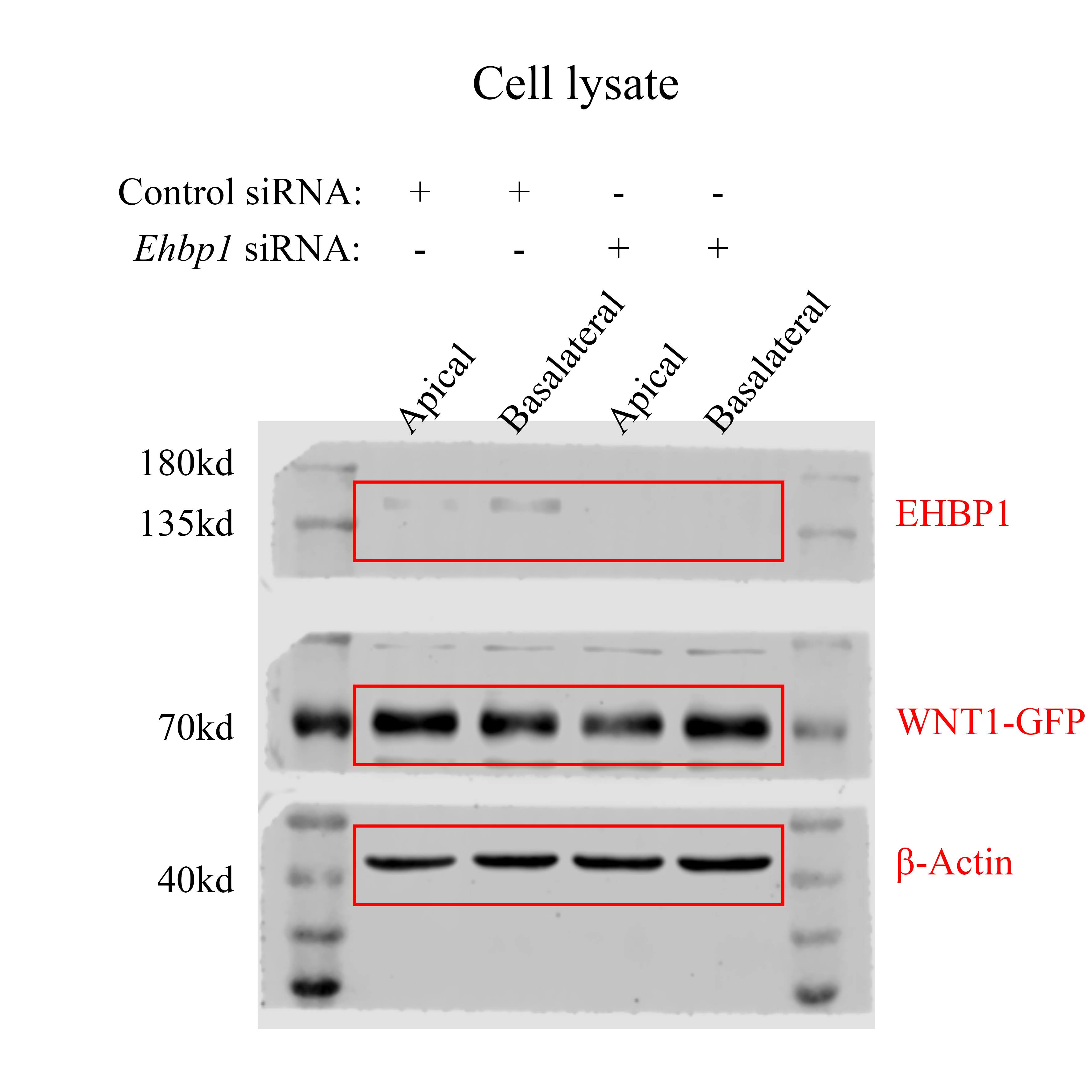

Supplement: Supplementary file 8 — Source data Fig. 6 [file 44319_2024_289_MOESM8_ESM.zip › Figure 6/F6A/F6A 20230926 MDCK transwell - input - Ehbp1 GFP actin - weak exposure.tif]

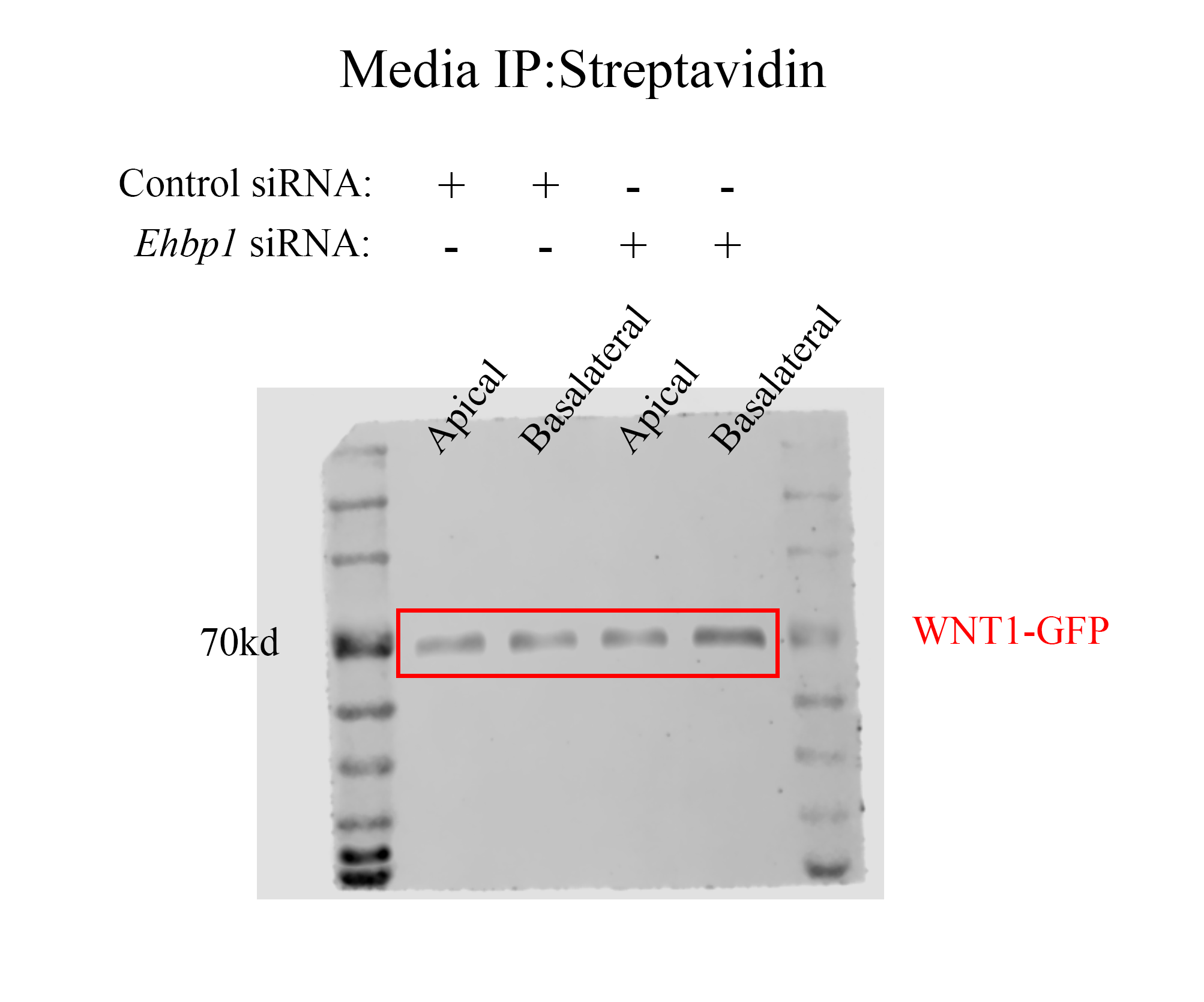

Supplement: Supplementary file 8 — Source data Fig. 6 [file 44319_2024_289_MOESM8_ESM.zip › Figure 6/F6A/F6A 20230926 MDCK transwell media - n biotin-IP - GFP - marker.tif]

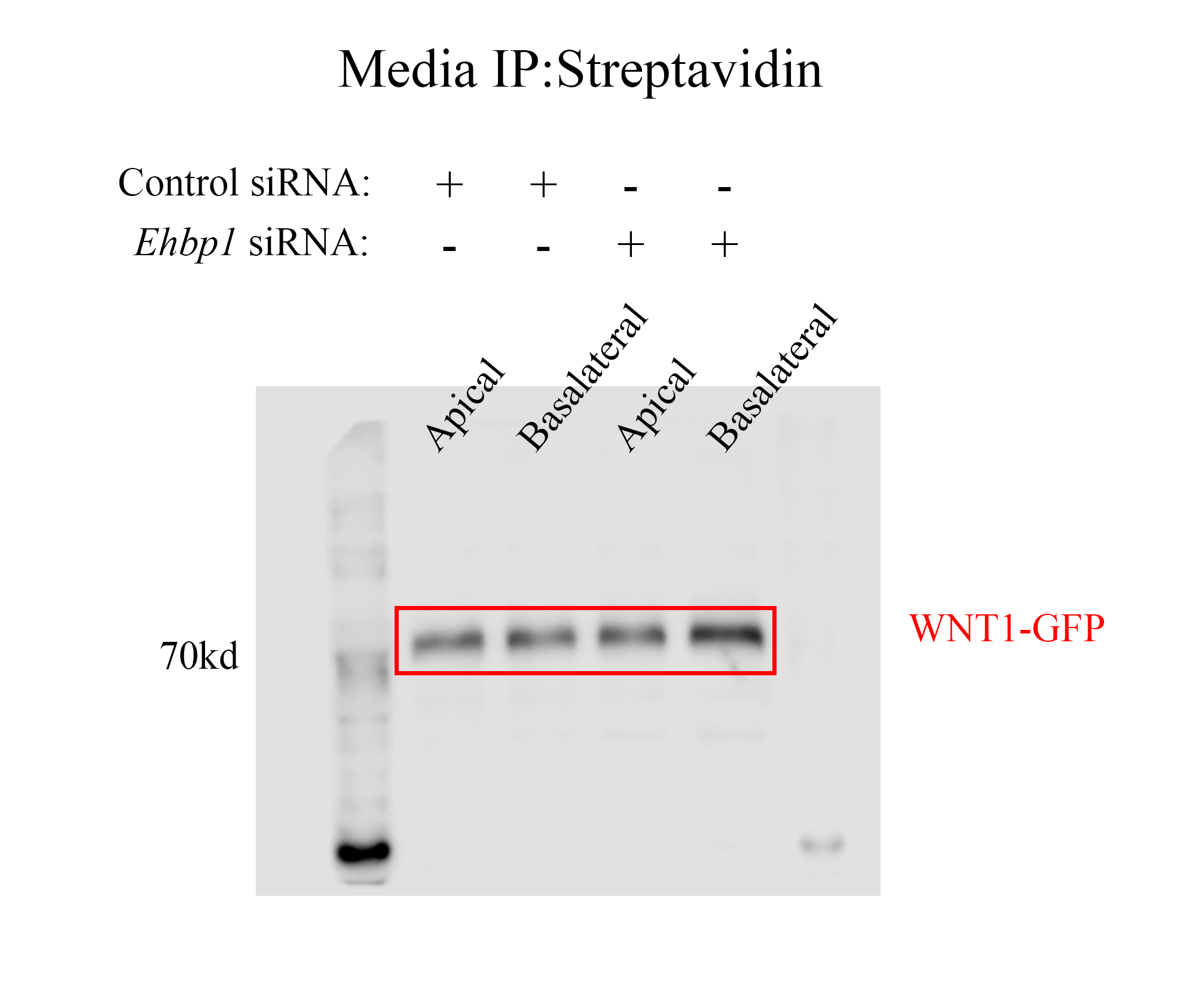

Supplement: Supplementary file 8 — Source data Fig. 6 [file 44319_2024_289_MOESM8_ESM.zip › Figure 6/F6A/F6A 20230926 MDCK transwell media n biotin-IP IB GFP2.tif]

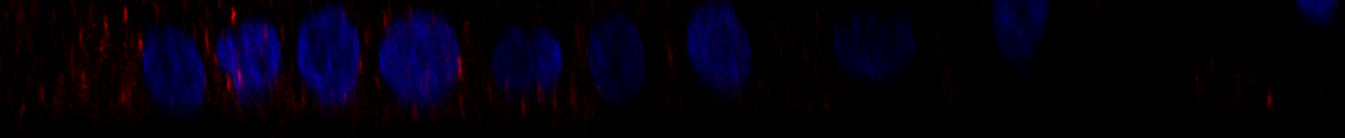

Supplement: Supplementary file 8 — Source data Fig. 6 [file 44319_2024_289_MOESM8_ESM.zip › Figure 6/F6B-F6M/F6B 20240715 MACD transwell WNT staining_20240715 MDCK NC RNAi WNT7A .tif]

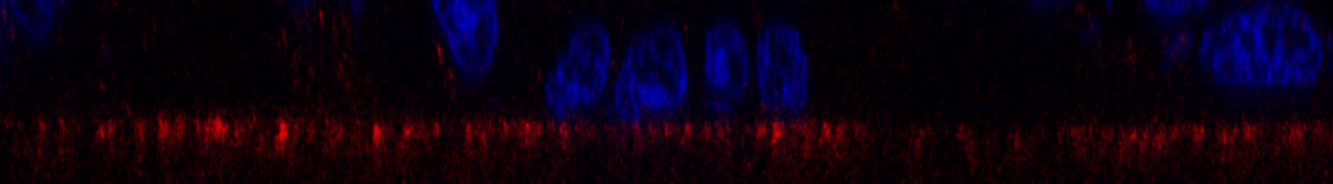

Supplement: Supplementary file 8 — Source data Fig. 6 [file 44319_2024_289_MOESM8_ESM.zip › Figure 6/F6B-F6M/F6C 20240811 MDCK transwell exWNT7A_20240811 MDCK transwell NC siRNA exWNT7A .tif]

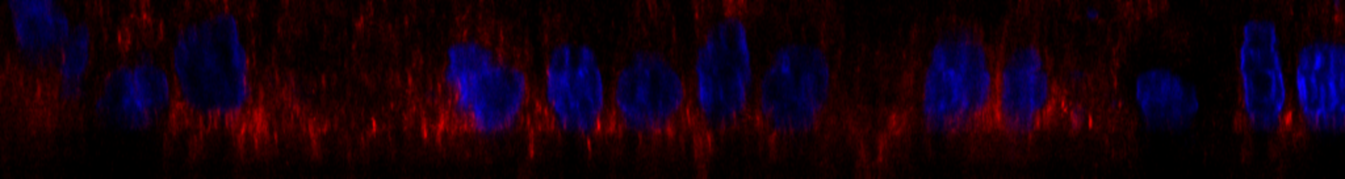

Supplement: Supplementary file 8 — Source data Fig. 6 [file 44319_2024_289_MOESM8_ESM.zip › Figure 6/F6B-F6M/F6D 20240626 MACD transwell WNT staining_20240626 MDCK Ehbp1 RNAi WNT7A .tif]

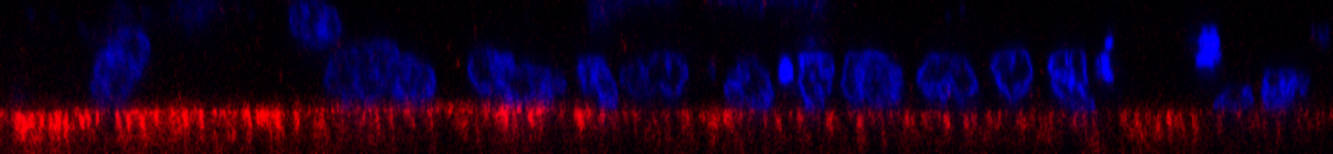

Supplement: Supplementary file 8 — Source data Fig. 6 [file 44319_2024_289_MOESM8_ESM.zip › Figure 6/F6B-F6M/F6E 20240801 MACD transwell exWNT7 staining_20240801 MDCK transwell EHBP1 siRNA exWNT7A .tif]

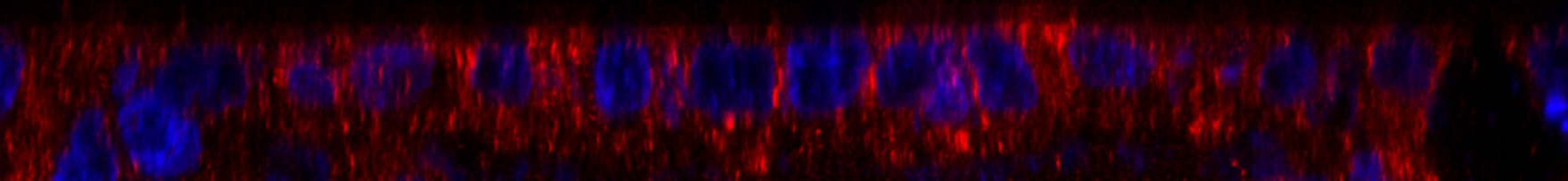

Supplement: Supplementary file 8 — Source data Fig. 6 [file 44319_2024_289_MOESM8_ESM.zip › Figure 6/F6B-F6M/F6F 20240626 MACD transwell WNT staining_20240626 MDCK Wls RNAi WNT7A .tif]

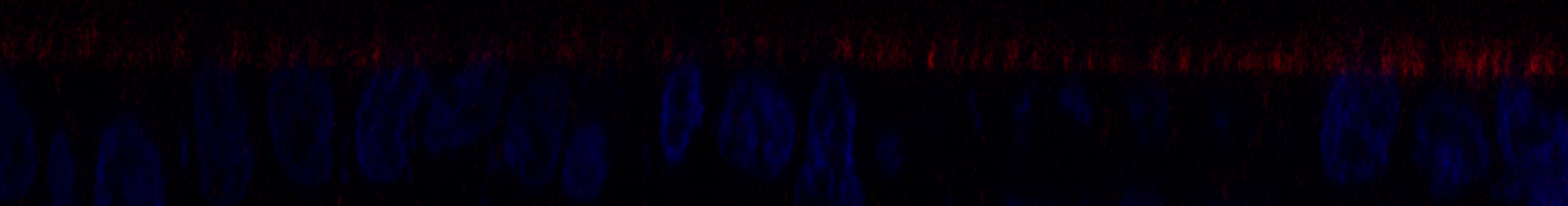

Supplement: Supplementary file 8 — Source data Fig. 6 [file 44319_2024_289_MOESM8_ESM.zip › Figure 6/F6B-F6M/F6G 20240811 MDCK transwell exWNT7A_20240811 MDCK transwell WLS siRNA exWNT7A .tif]

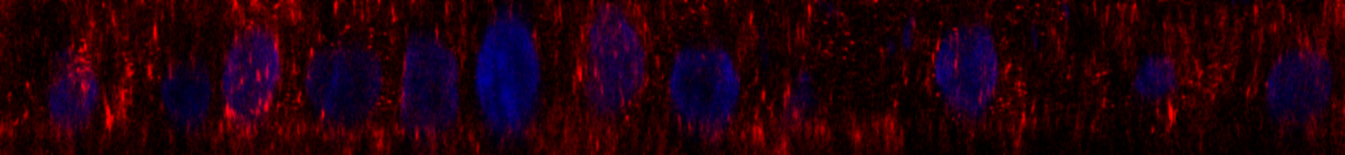

Supplement: Supplementary file 8 — Source data Fig. 6 [file 44319_2024_289_MOESM8_ESM.zip › Figure 6/F6B-F6M/F6H 20240715 MACD transwell WNT staining_20240715 MDCK Ehbp1 WLS RNAi WNT7A .tif]

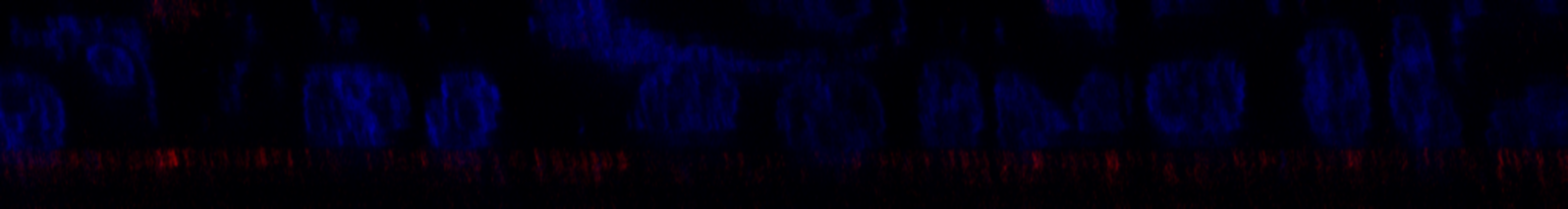

Supplement: Supplementary file 8 — Source data Fig. 6 [file 44319_2024_289_MOESM8_ESM.zip › Figure 6/F6B-F6M/F6I 20240811 MDCK transwell exWNT7A_20240811 MACD transwell EHBP1 WLS siRNA EXWNT7.tif]

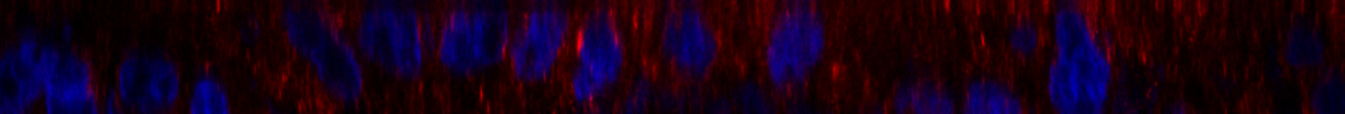

Supplement: Supplementary file 8 — Source data Fig. 6 [file 44319_2024_289_MOESM8_ESM.zip › Figure 6/F6B-F6M/F6J 20240626 MACD transwell WNT staining_20240626 MDCK AP1uA RNAi WNT7A .tif]

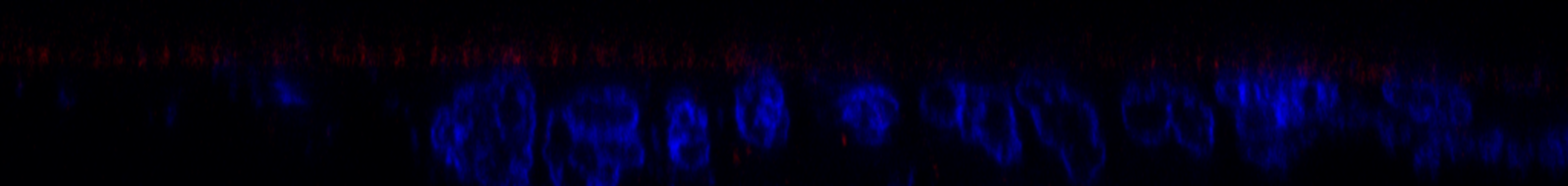

Supplement: Supplementary file 8 — Source data Fig. 6 [file 44319_2024_289_MOESM8_ESM.zip › Figure 6/F6B-F6M/F6K 20240811 MDCK transwell exWNT7A_20240811 MACD transwell AP1 siRNA EXWNT7 .tif]

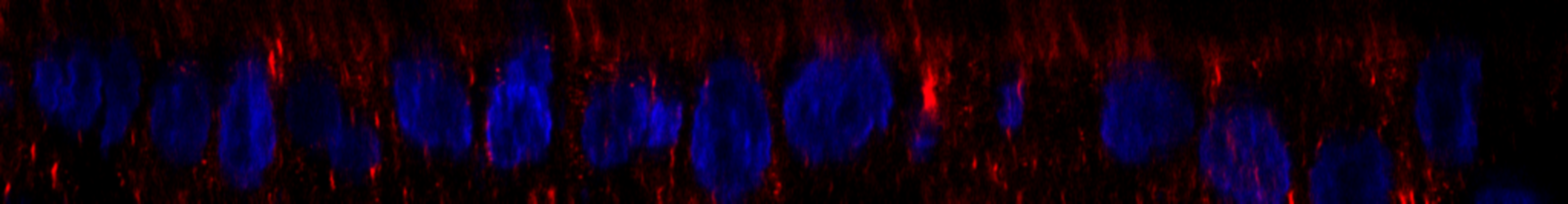

Supplement: Supplementary file 8 — Source data Fig. 6 [file 44319_2024_289_MOESM8_ESM.zip › Figure 6/F6B-F6M/F6L 20240715 MACD transwell WNT staining_20240715 MDCK Ehbp1 AP-1uA RNAi WNT7A .tif]

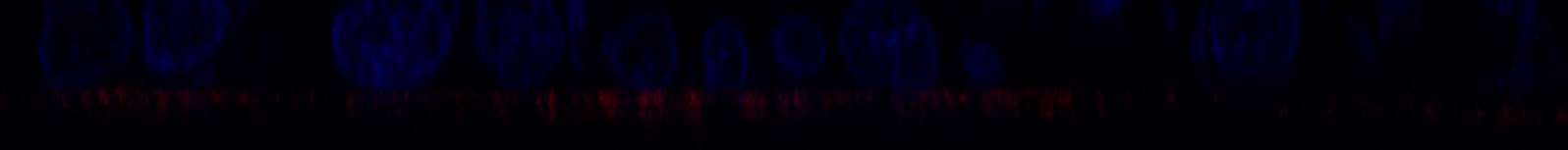

Supplement: Supplementary file 8 — Source data Fig. 6 [file 44319_2024_289_MOESM8_ESM.zip › Figure 6/F6B-F6M/F6M 20240811 MDCK transwell exWNT7A_20240811 MACD transwell EHBP1 AP1 siRNA EXWNT7 .tif]

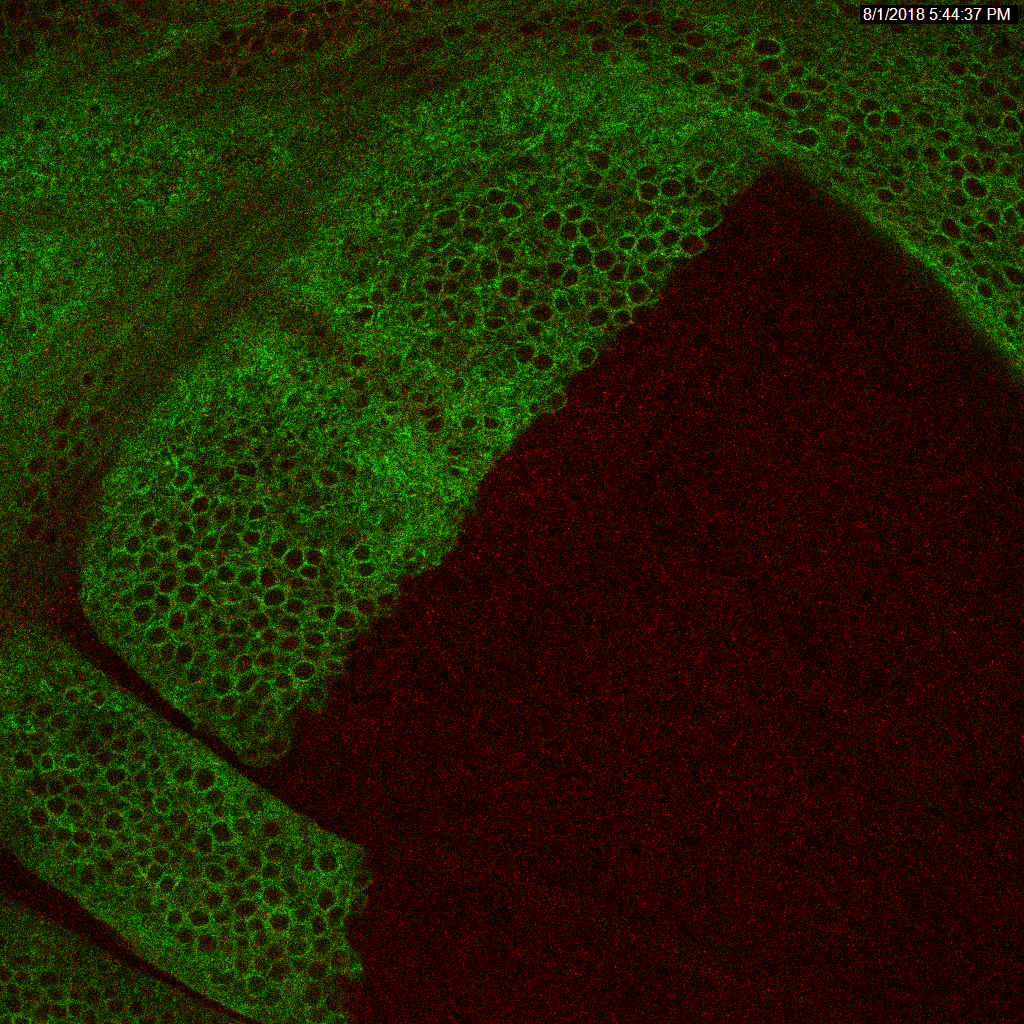

Supplement: Supplementary file 9 — EV Figures Source Data [file 44319_2024_289_MOESM9_ESM.zip › Figure EV1/EV1A/EV1A Apical ts-Gal80 hh-G4-GFP TH02340 ExWg 3D_Series003_z023.tif]

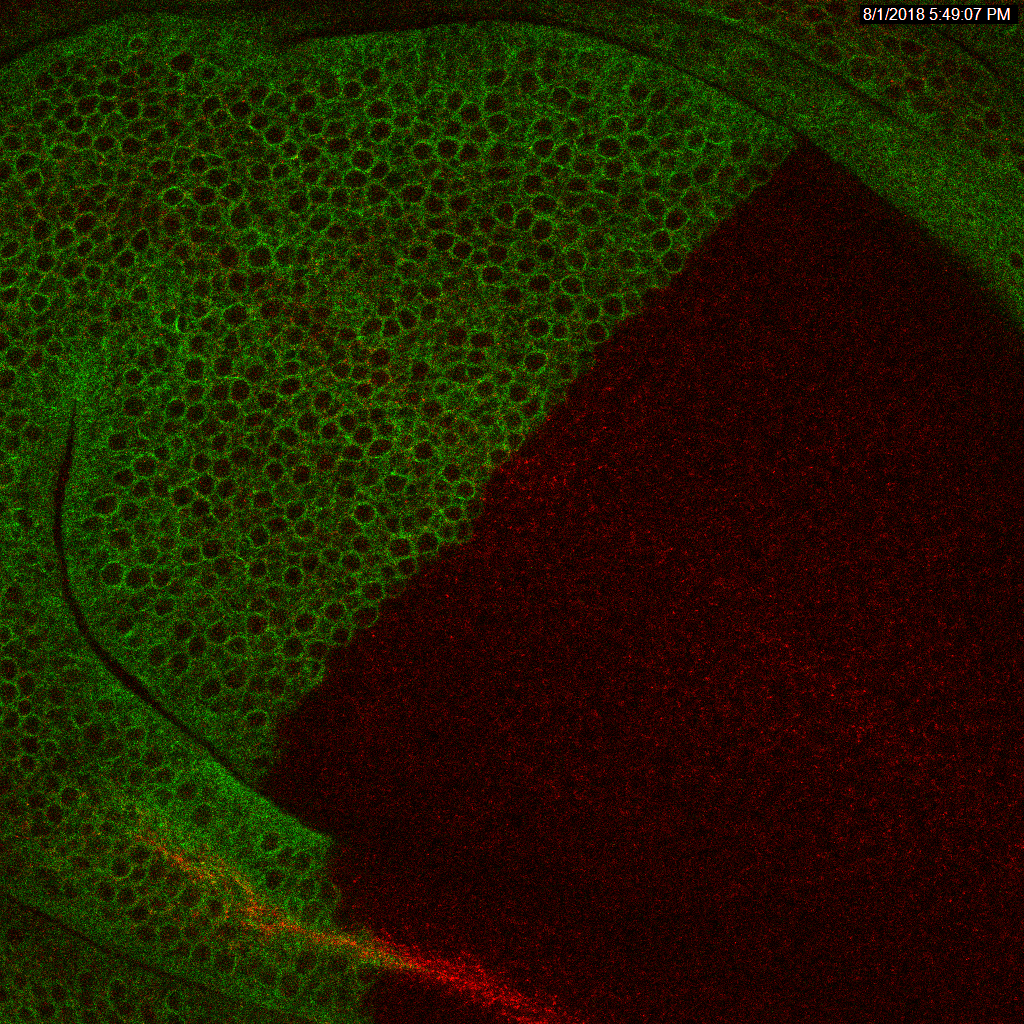

Supplement: Supplementary file 9 — EV Figures Source Data [file 44319_2024_289_MOESM9_ESM.zip › Figure EV1/EV1A/EV1A Basalateral ts-Gal80 hh-G4-GFP TH02340 ExWg 3D_Series003_z047.tif]

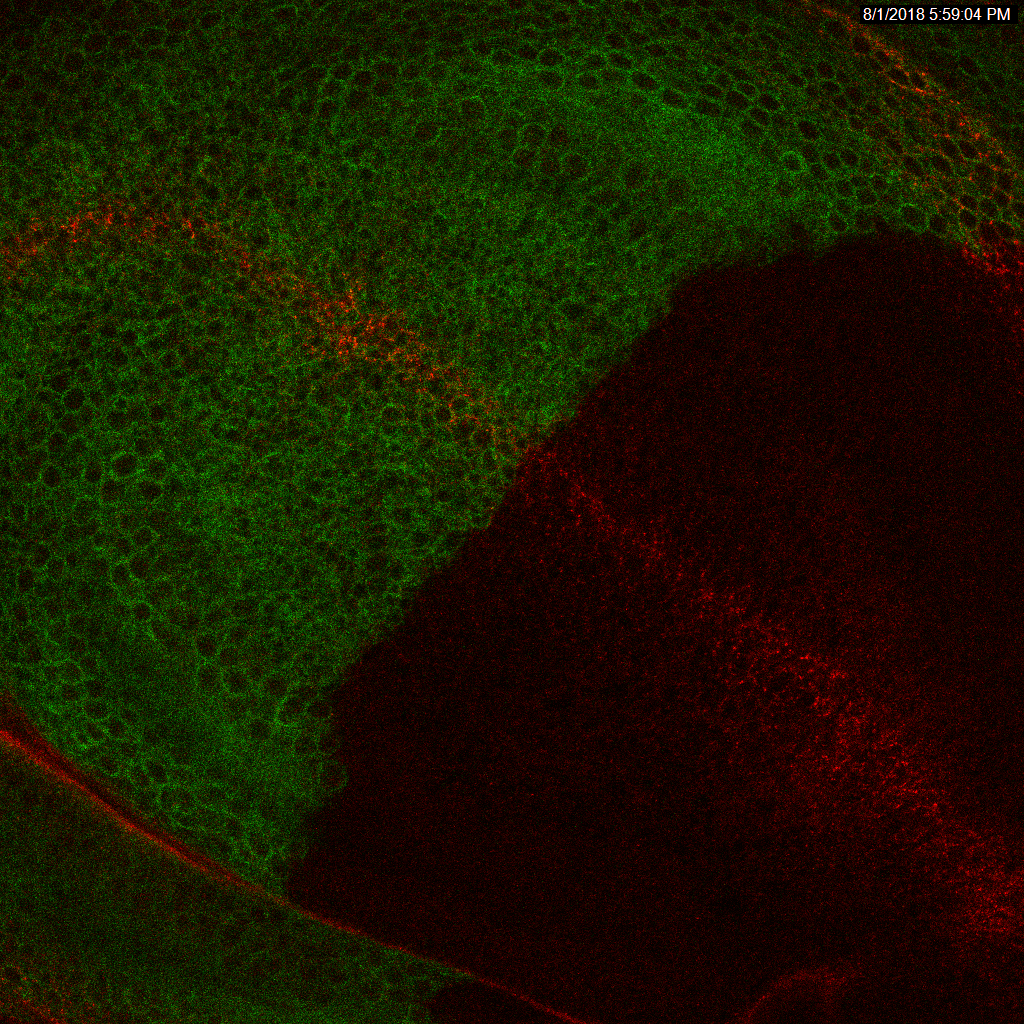

Supplement: Supplementary file 9 — EV Figures Source Data [file 44319_2024_289_MOESM9_ESM.zip › Figure EV1/EV1A/EV1A Subapical ts-Gal80 hh-G4-GFP TH02340 ExWg 3D_Series003_z100.tif]

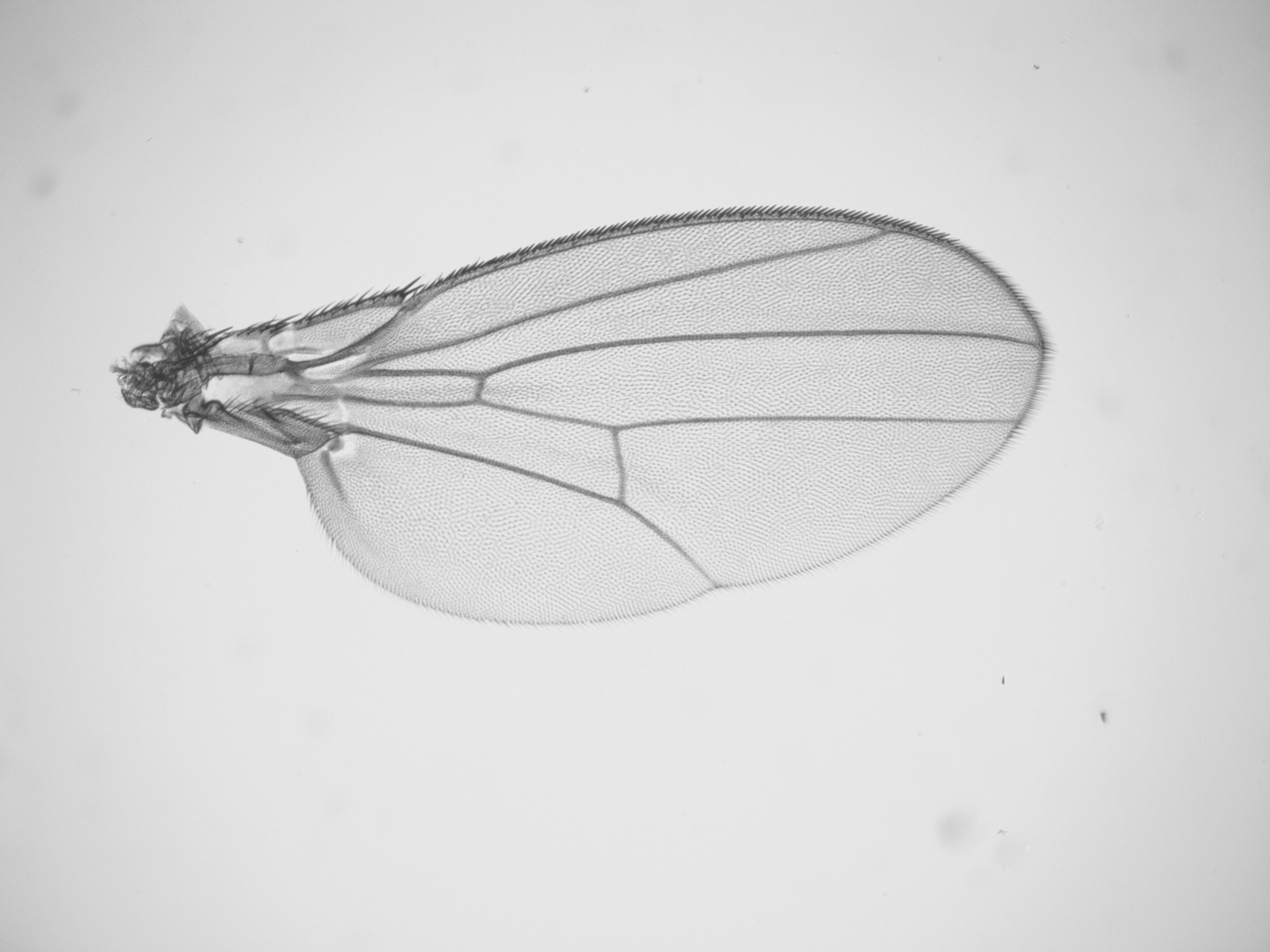

Supplement: Supplementary file 9 — EV Figures Source Data [file 44319_2024_289_MOESM9_ESM.zip › Figure EV1/EV1B/EV1B UAS-GFP Wg-G4 25C 1.tif]

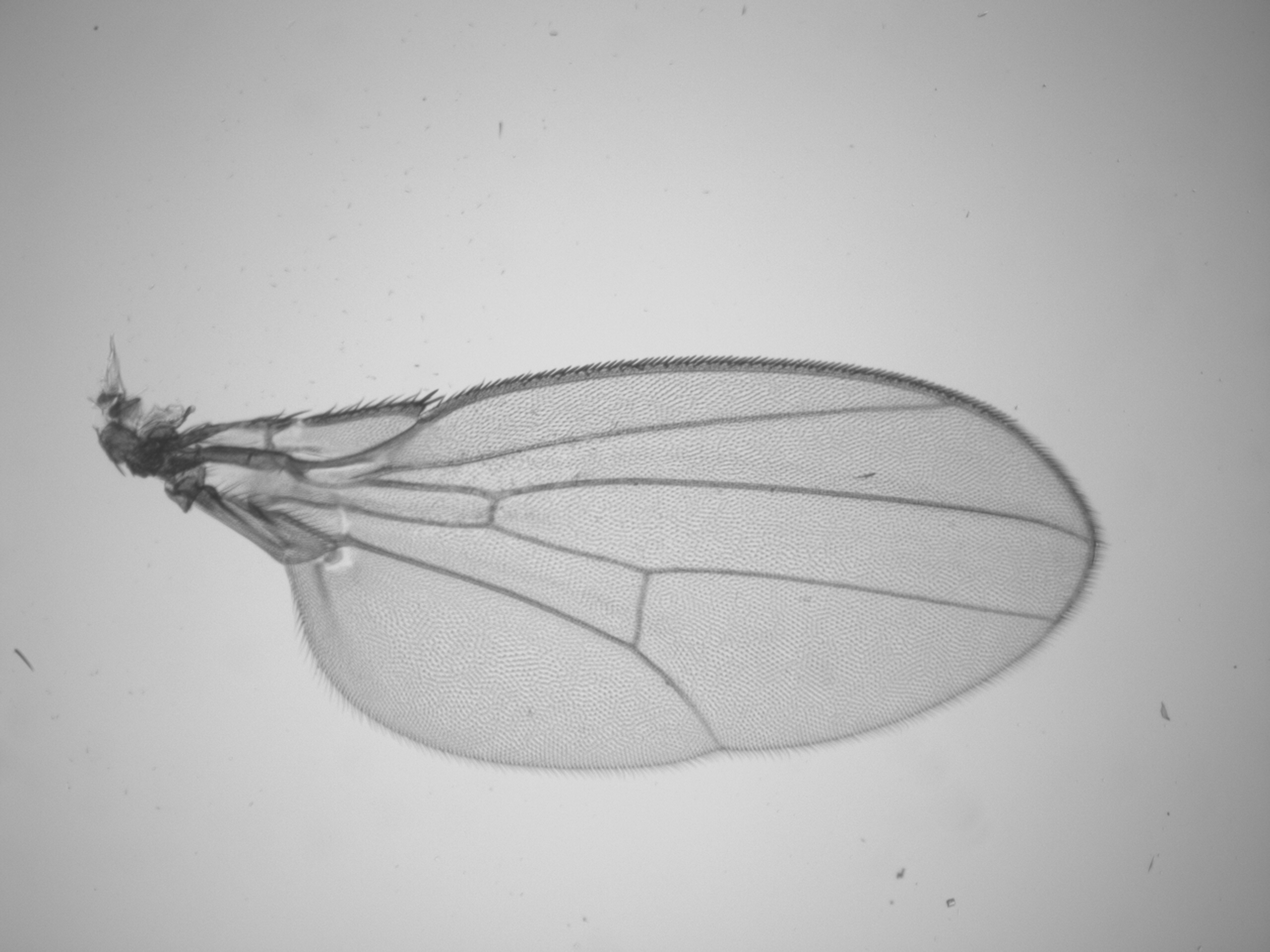

Supplement: Supplementary file 9 — EV Figures Source Data [file 44319_2024_289_MOESM9_ESM.zip › Figure EV1/EV1C/EV1C 231031 29C wg-G4 Gzl.LD -1 -1.tif]

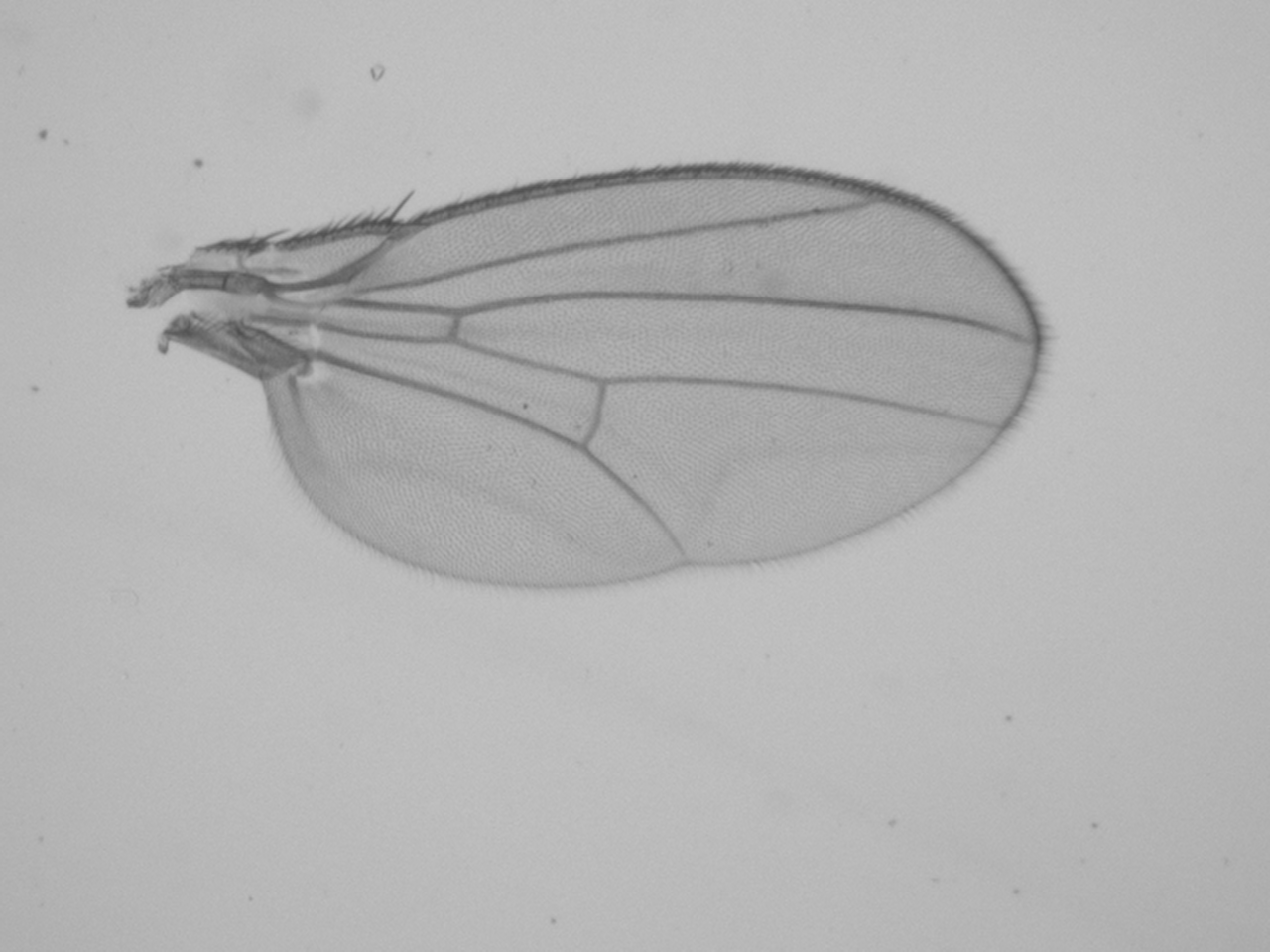

Supplement: Supplementary file 9 — EV Figures Source Data [file 44319_2024_289_MOESM9_ESM.zip › Figure EV1/EV1F/EV1F UAS-GFP Hh-G4 25C 1.tif]

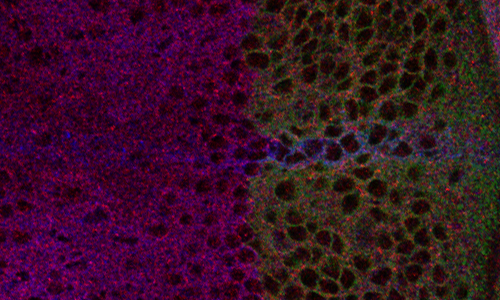

Supplement: Supplementary file 9 — EV Figures Source Data [file 44319_2024_289_MOESM9_ESM.zip › Figure EV2/EV2B/EV2B Images for statistical analysis - Ehbp1/190806 hh-G4-GFP BL41133 29C Ehbp1 wg_Series001_Lng_z024.tif]

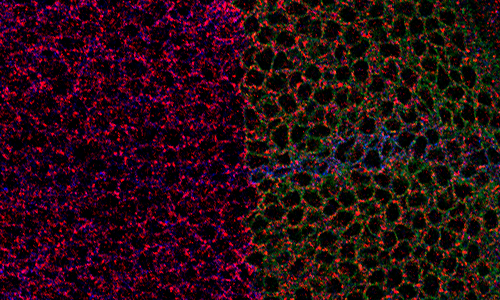

Supplement: Supplementary file 9 — EV Figures Source Data [file 44319_2024_289_MOESM9_ESM.zip › Figure EV2/EV2B/EV2B Images for statistical analysis - Ehbp1/190806 hh-G4-GFP BL41133 29C Ehbp1 wg_Series001_Lng_z049 -2.tif]

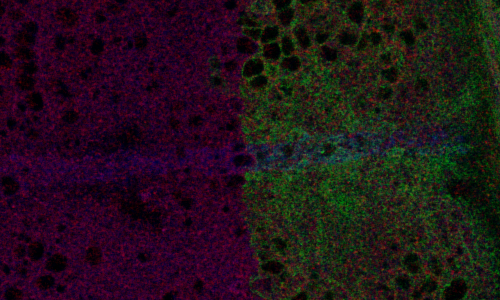

Supplement: Supplementary file 9 — EV Figures Source Data [file 44319_2024_289_MOESM9_ESM.zip › Figure EV2/EV2B/EV2B Images for statistical analysis - Ehbp1/190806 hh-G4-GFP BL41133 29C Ehbp1 wg_Series002_Lng_z019.tif]

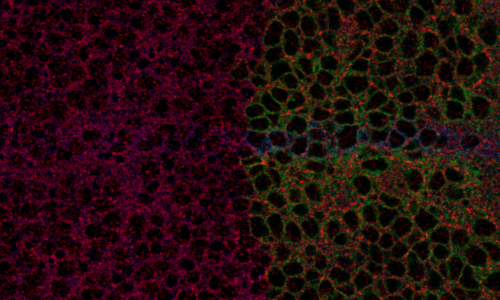

Supplement: Supplementary file 9 — EV Figures Source Data [file 44319_2024_289_MOESM9_ESM.zip › Figure EV2/EV2B/EV2B Images for statistical analysis - Ehbp1/190806 hh-G4-GFP BL41133 29C Ehbp1 wg_Series002_Lng_z037 -2.tif]

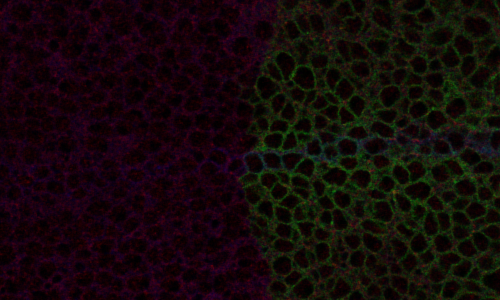

Supplement: Supplementary file 9 — EV Figures Source Data [file 44319_2024_289_MOESM9_ESM.zip › Figure EV2/EV2B/EV2B Images for statistical analysis - Ehbp1/190806 hh-G4-GFP BL41133 29C Ehbp1 wg_Series002_Lng_z058.tif]

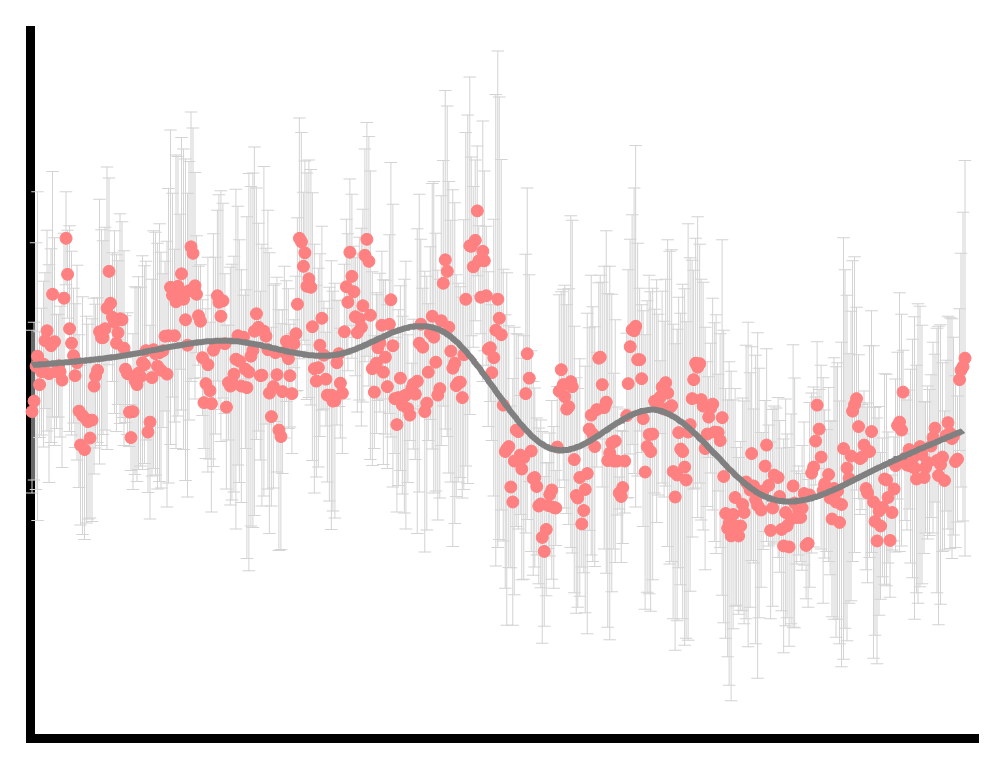

Supplement: Supplementary file 9 — EV Figures Source Data [file 44319_2024_289_MOESM9_ESM.zip › Figure EV2/EV2B/Figure EV2 B'' mir-bft oe Ehbp1.tif]

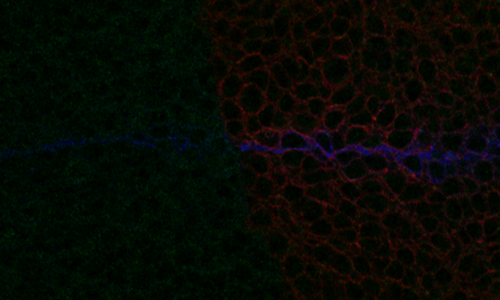

Supplement: Supplementary file 9 — EV Figures Source Data [file 44319_2024_289_MOESM9_ESM.zip › Figure EV2/EV2C/EV2C Images for statistical analysis - WT sensor/200501 tub-Ehbp1-GFP ts-gal80 hh-G4-RFP BL41133 gfp wg 34h_Series001_Lng_z26 2.tif]

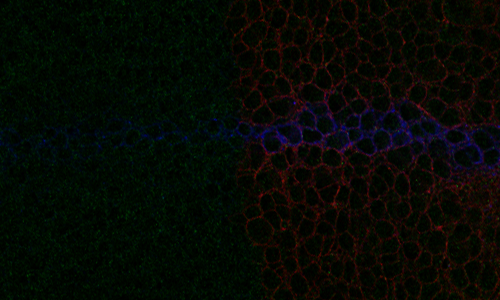

Supplement: Supplementary file 9 — EV Figures Source Data [file 44319_2024_289_MOESM9_ESM.zip › Figure EV2/EV2C/EV2C Images for statistical analysis - WT sensor/200501 tub-Ehbp1-GFP ts-gal80 hh-G4-RFP BL41133 gfp wg 34h_Series002_Lng_z15 2.tif]

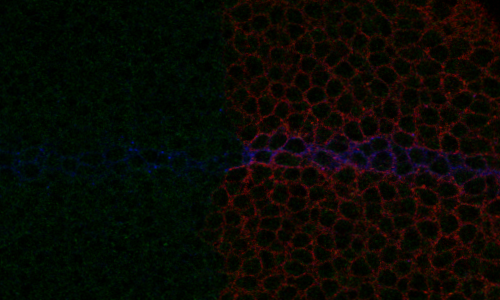

Supplement: Supplementary file 9 — EV Figures Source Data [file 44319_2024_289_MOESM9_ESM.zip › Figure EV2/EV2C/EV2C Images for statistical analysis - WT sensor/200501 tub-Ehbp1-GFP ts-gal80 hh-G4-RFP BL41133 gfp wg 34h_Series003_Lng_z15 2.tif]

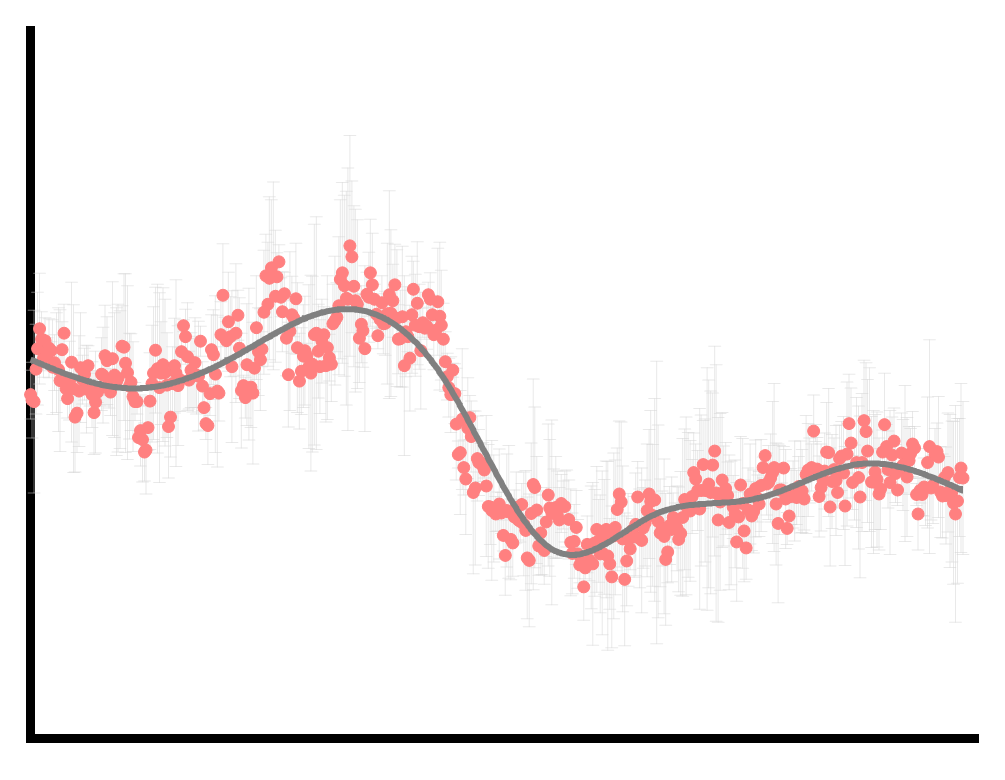

Supplement: Supplementary file 9 — EV Figures Source Data [file 44319_2024_289_MOESM9_ESM.zip › Figure EV2/EV2C/Figure EV2 C'' Ehbp1 wt sensor ExWg.tif]

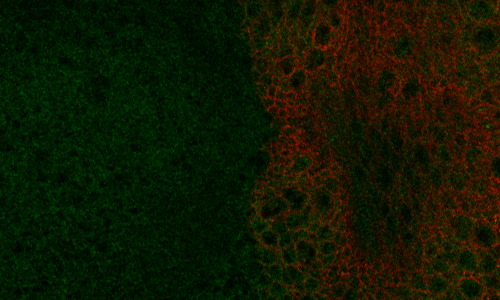

Supplement: Supplementary file 9 — EV Figures Source Data [file 44319_2024_289_MOESM9_ESM.zip › Figure EV2/EV2D/EV2D Images for statistical analysis - Mut sensor/200303 tub-GFP-Ehbp13UTR-mut ts-Gal80 hh-G4-RFP BL41133 GFP 48h_Series003_000_Lng_z09.tif]

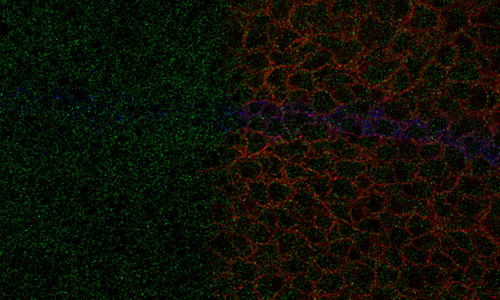

Supplement: Supplementary file 9 — EV Figures Source Data [file 44319_2024_289_MOESM9_ESM.zip › Figure EV2/EV2D/EV2D Images for statistical analysis - Mut sensor/200516 tub-GFP-Ehbp1-3'UTR-mut hh-G4-RFP BL41133 Wg GFP_Series002_Lng_global_z08 2.tif]

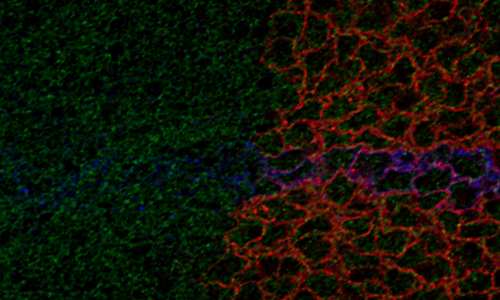

Supplement: Supplementary file 9 — EV Figures Source Data [file 44319_2024_289_MOESM9_ESM.zip › Figure EV2/EV2D/EV2D Images for statistical analysis - Mut sensor/200516 tub-GFP-Ehbp1-3'UTR-mut hh-G4-RFP BL41133 Wg GFP_Series003_Lng_global_z11 2.tif]

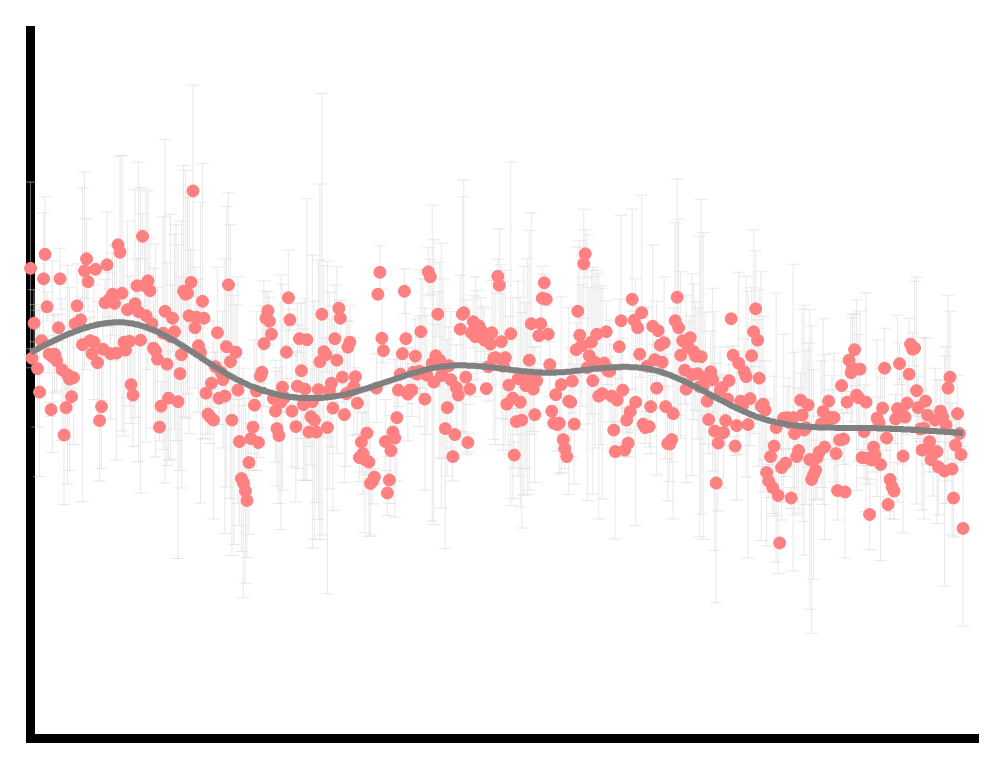

Supplement: Supplementary file 9 — EV Figures Source Data [file 44319_2024_289_MOESM9_ESM.zip › Figure EV2/EV2D/Figure EV2 D'' Ehbp1 mut sensor ExWg.tif]

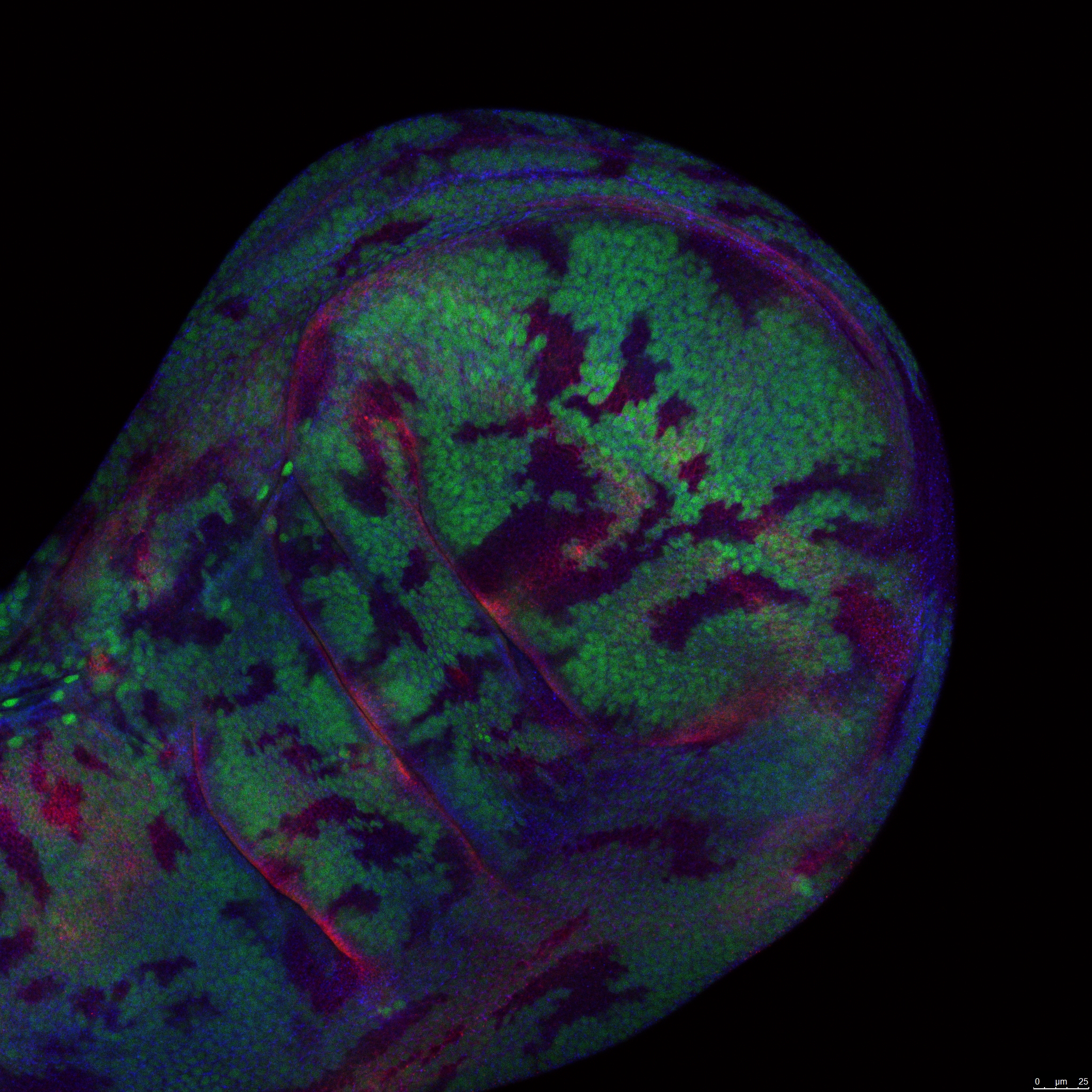

Supplement: Supplementary file 9 — EV Figures Source Data [file 44319_2024_289_MOESM9_ESM.zip › Figure EV3/EV3A/EV3A 20210511 Ehbp1 mutant Dl Ehbp1.lif_20210511 A28 Minute 42D Dl Ehbp1 -1 -z_z2.jpg]

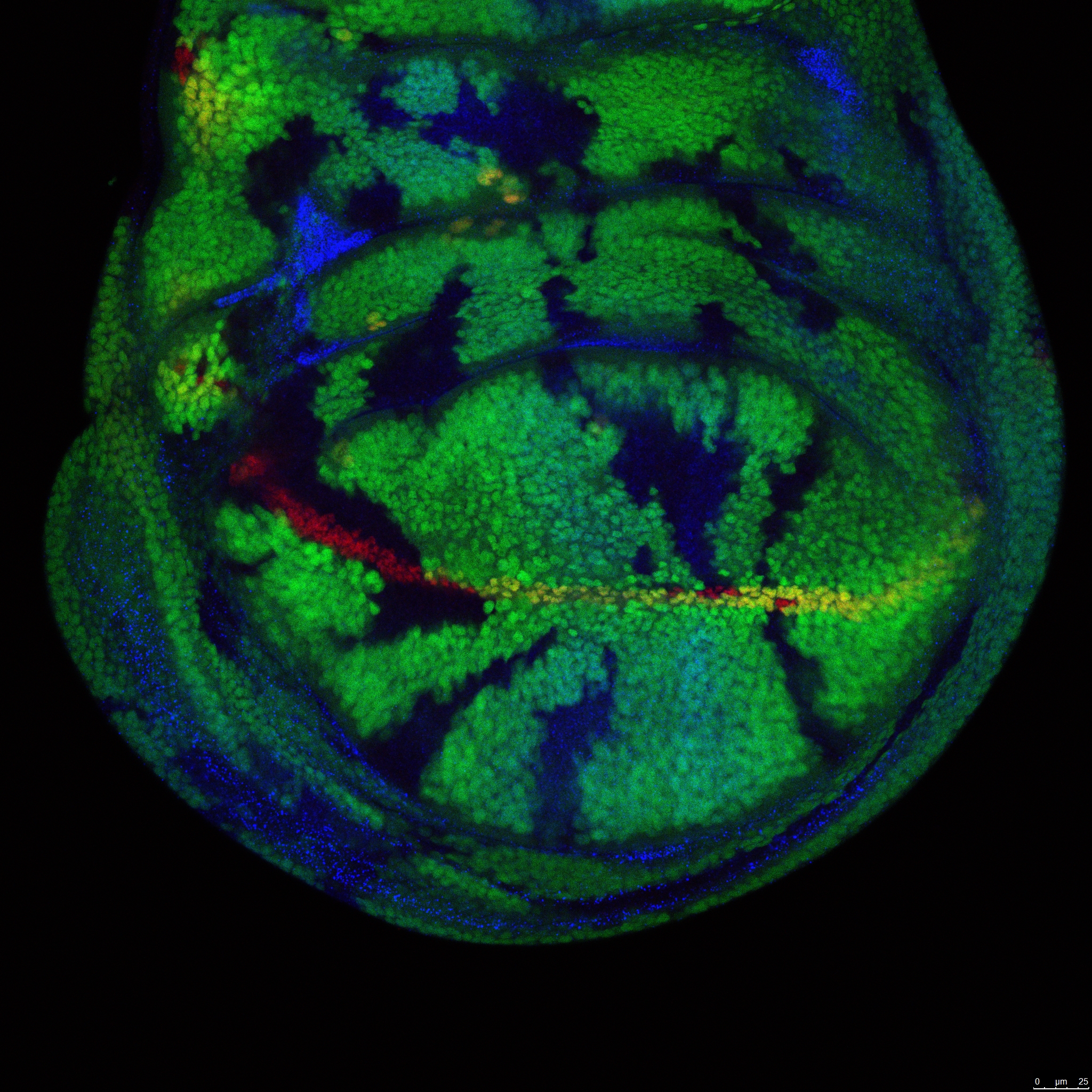

Supplement: Supplementary file 9 — EV Figures Source Data [file 44319_2024_289_MOESM9_ESM.zip › Figure EV3/EV3B/EV3B 20210517 Ehbp1 mutant Cut pSmad.lif_20210517 A28 Minute 42D Cut pSmad -1_z0.jpg]

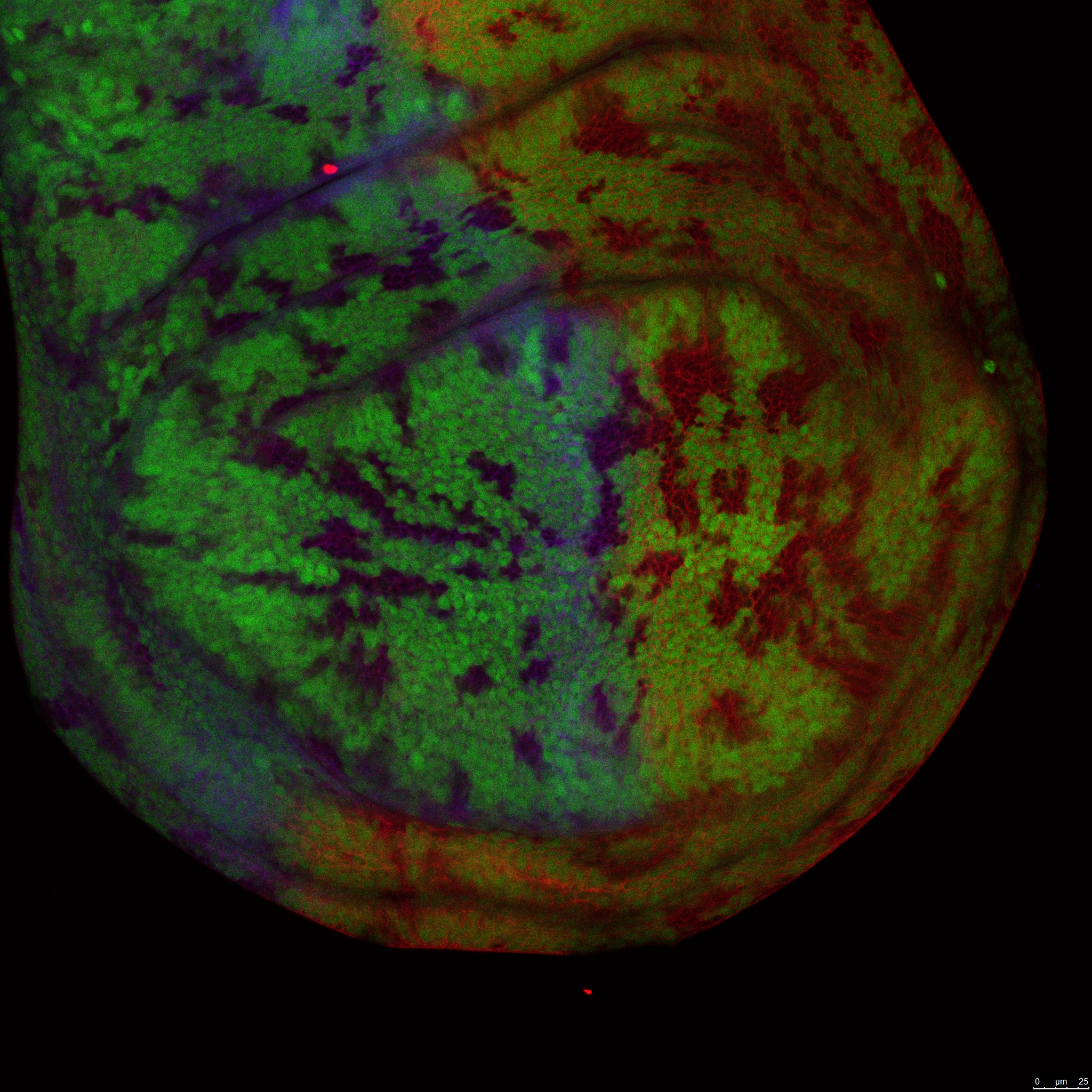

Supplement: Supplementary file 9 — EV Figures Source Data [file 44319_2024_289_MOESM9_ESM.zip › Figure EV3/EV3C/EV3C 20210511 Ehbp1 mutant Smo Ci.lif_20210511 A28 Minute 42D Smo Ci -3_z0.jpg]

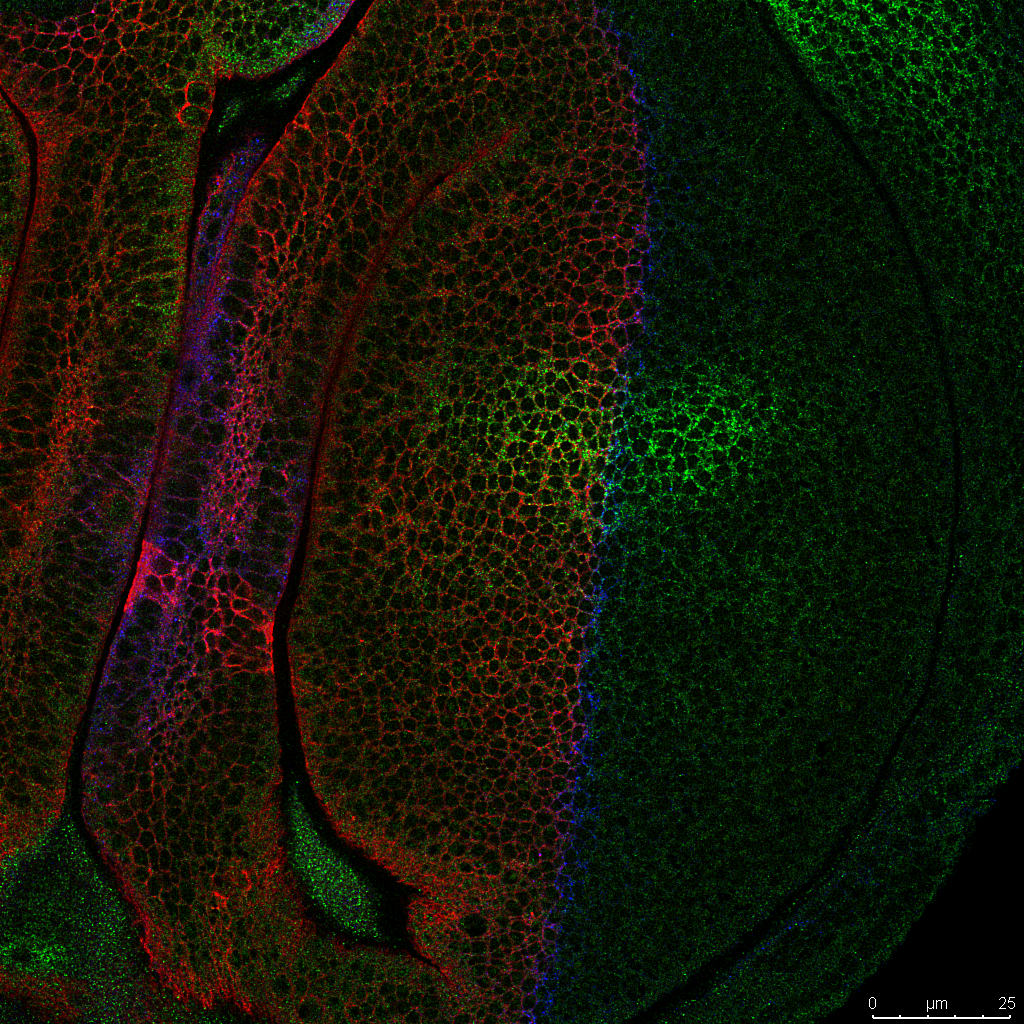

Supplement: Supplementary file 9 — EV Figures Source Data [file 44319_2024_289_MOESM9_ESM.zip › Figure EV3/EV3D/EV3D ap-G4-RFP TH02340 dpp-GFP wg_Series004_Lng_adaptive_z052.tif]

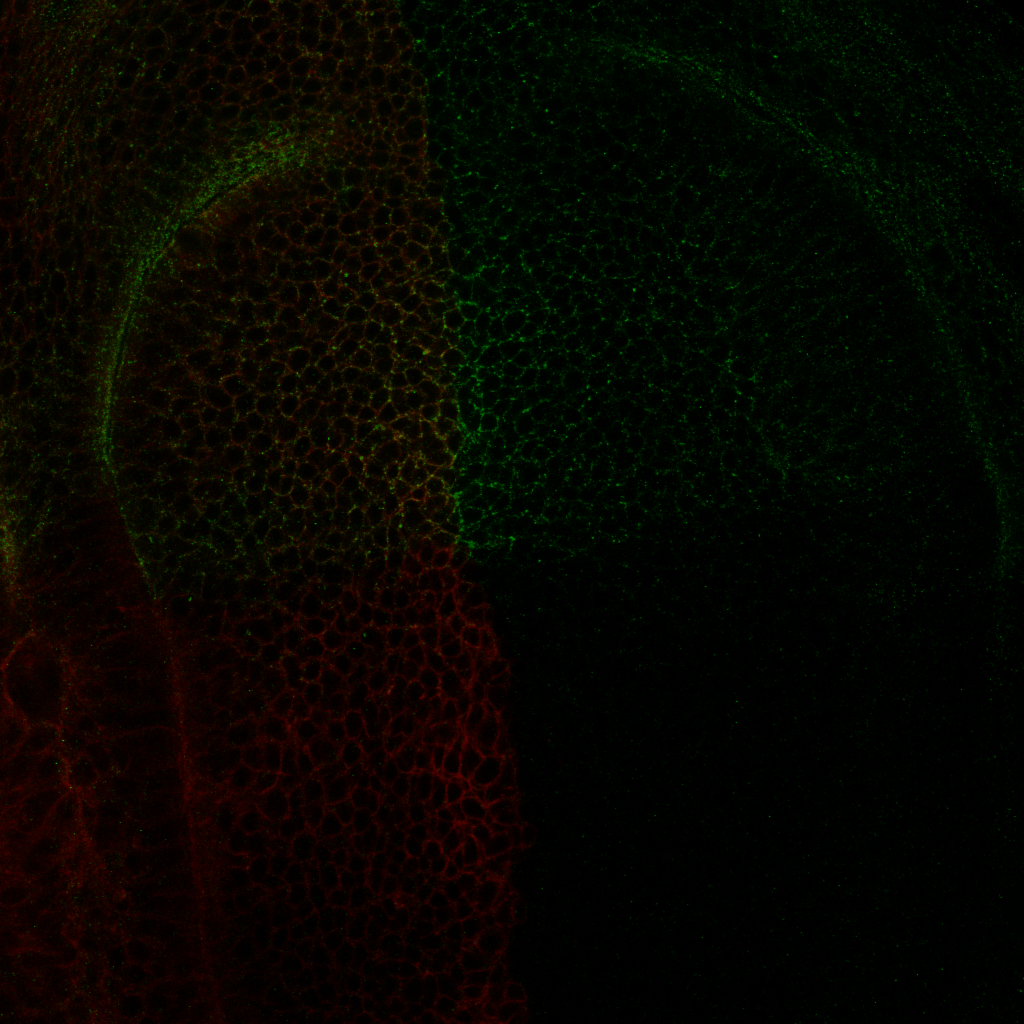

Supplement: Supplementary file 9 — EV Figures Source Data [file 44319_2024_289_MOESM9_ESM.zip › Figure EV3/EV3E/EV3E ap-G4-RFP TH02340 hh-GFP_Series002_Lng_adaptive_z20.tif]

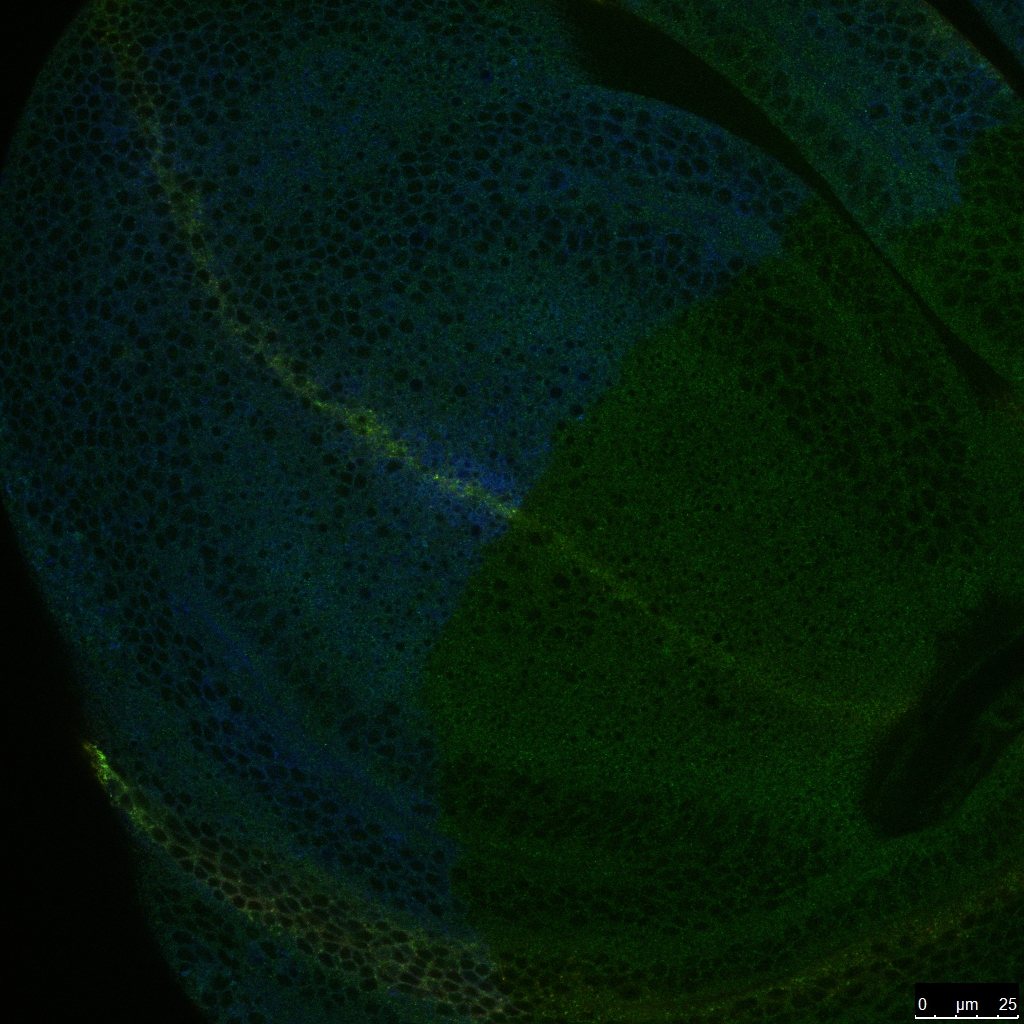

Supplement: Supplementary file 9 — EV Figures Source Data [file 44319_2024_289_MOESM9_ESM.zip › Figure EV4/EV4A/EV4A1 20230811 hh-Gal4 Exocyst RNA Wls Wg.lif_20230812 24C hh-Gal4 Th2636 -2 -B_z0.jpg]

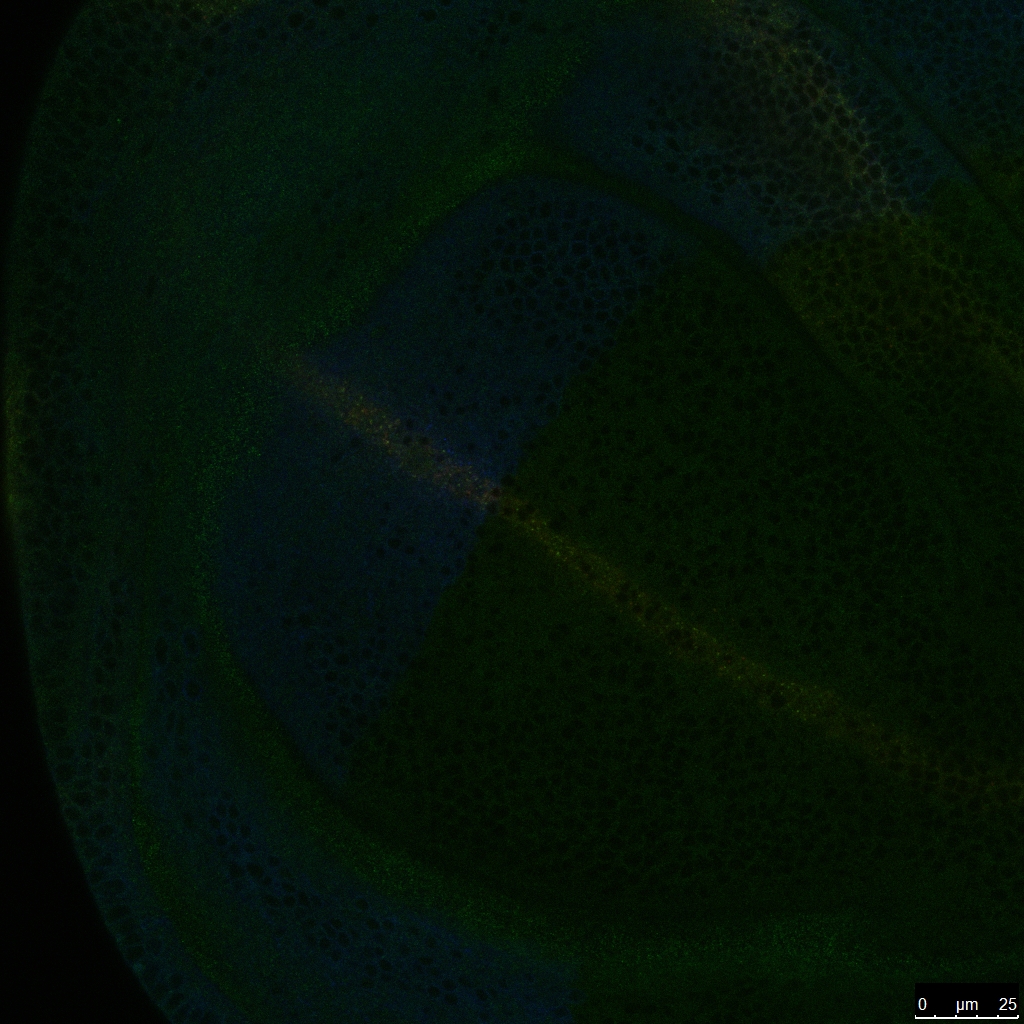

Supplement: Supplementary file 9 — EV Figures Source Data [file 44319_2024_289_MOESM9_ESM.zip › Figure EV4/EV4A/EV4A2 20230811 hh-Gal4 Exocyst RNA Wls Wg.lif_20230812 24C hh-Gal4 Th2636 -2 -A_z0.jpg]

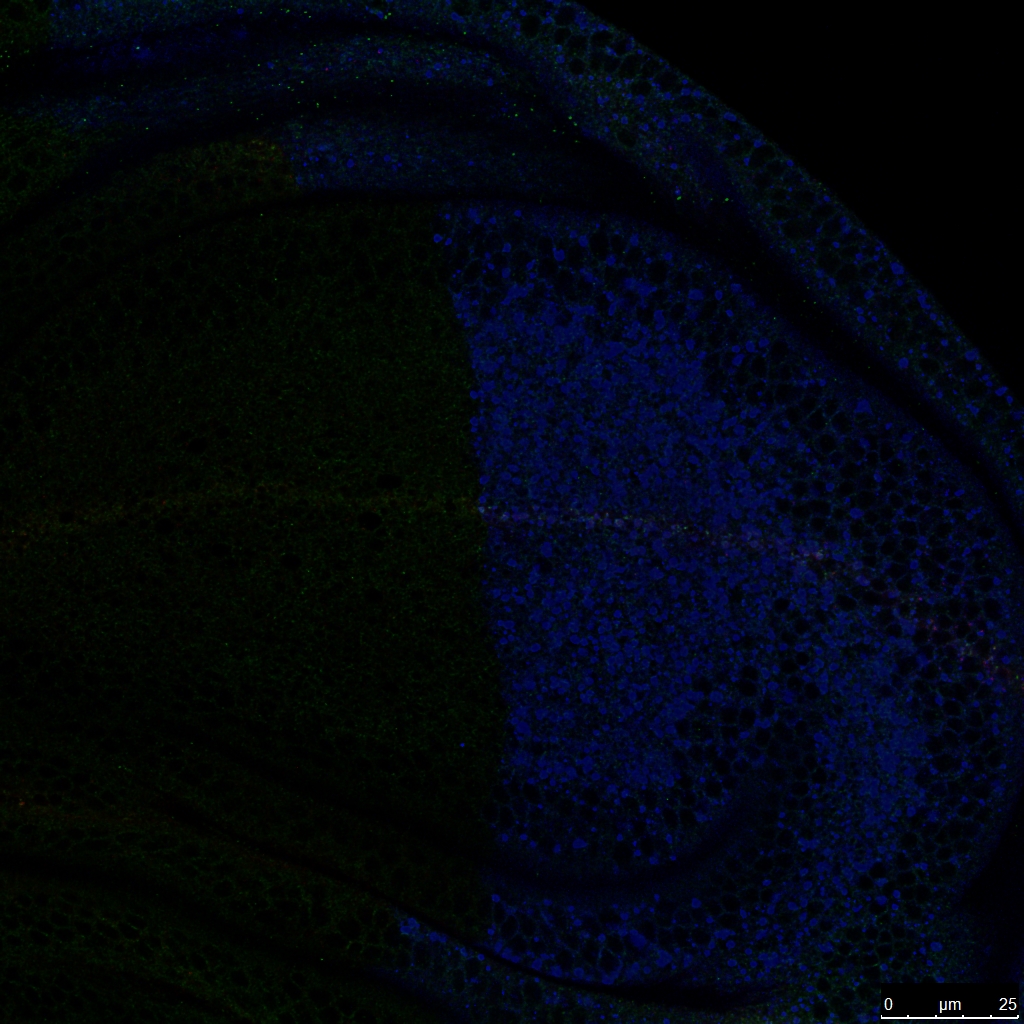

Supplement: Supplementary file 9 — EV Figures Source Data [file 44319_2024_289_MOESM9_ESM.zip › Figure EV4/EV4B/EV4B1 ts-Gal80 hh-G4-GFP UAS-God LD wg wls.lif_Series002_Lng_global_SubVolume001_z1.jpg]

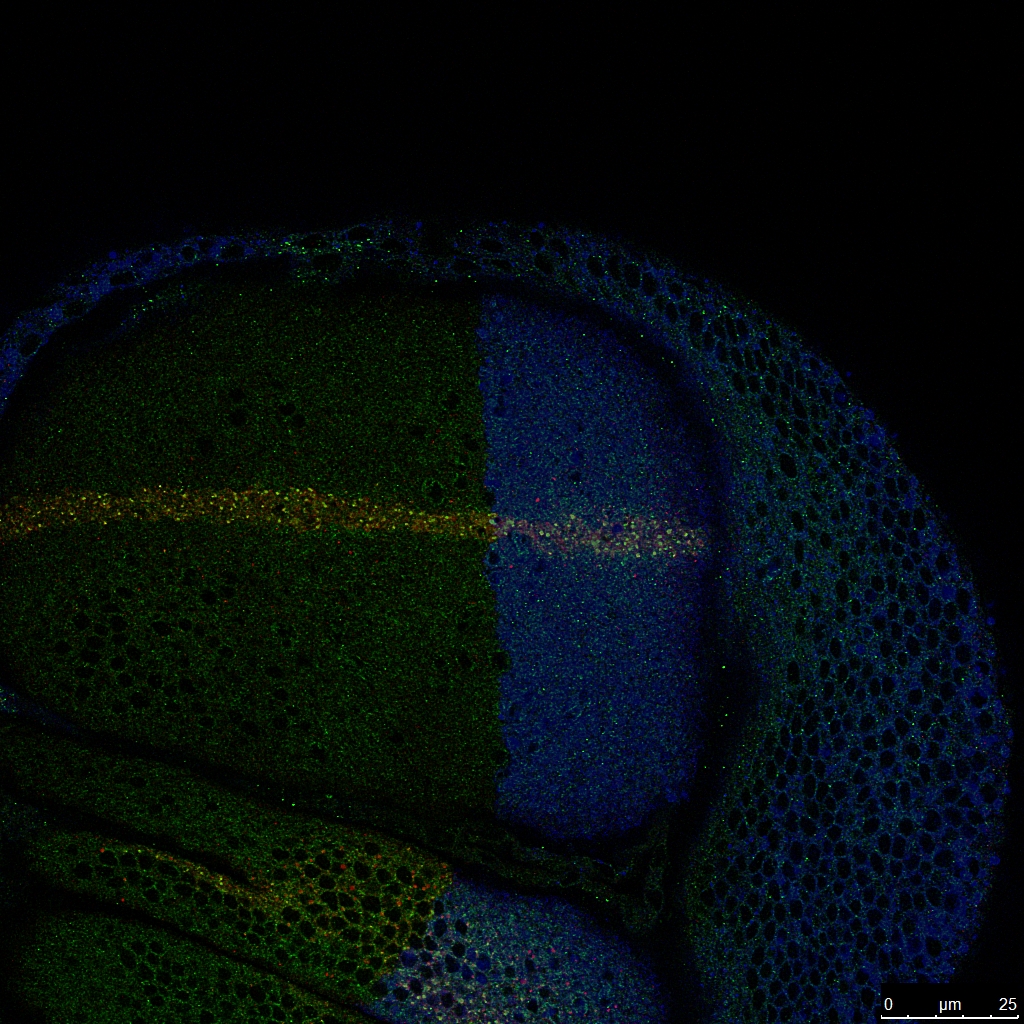

Supplement: Supplementary file 9 — EV Figures Source Data [file 44319_2024_289_MOESM9_ESM.zip › Figure EV4/EV4B/EV4B2 ts-Gal80 hh-G4-GFP UAS-God LD wg wls.lif_Series002_Lng_global_SubVolume001_z0.jpg]

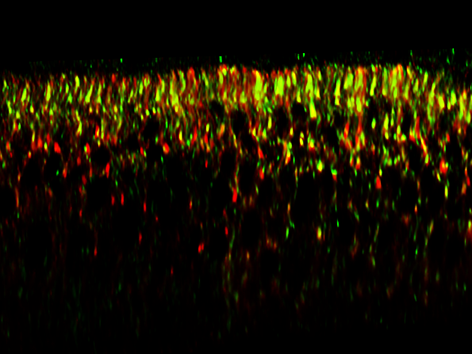

Supplement: Supplementary file 9 — EV Figures Source Data [file 44319_2024_289_MOESM9_ESM.zip › Figure EV4/EV4B/EV4B3 ts-Gal80 hh-G4-GFP UAS-God LD wg wls_Series002_Lng_global - 3D-Projection.tif]

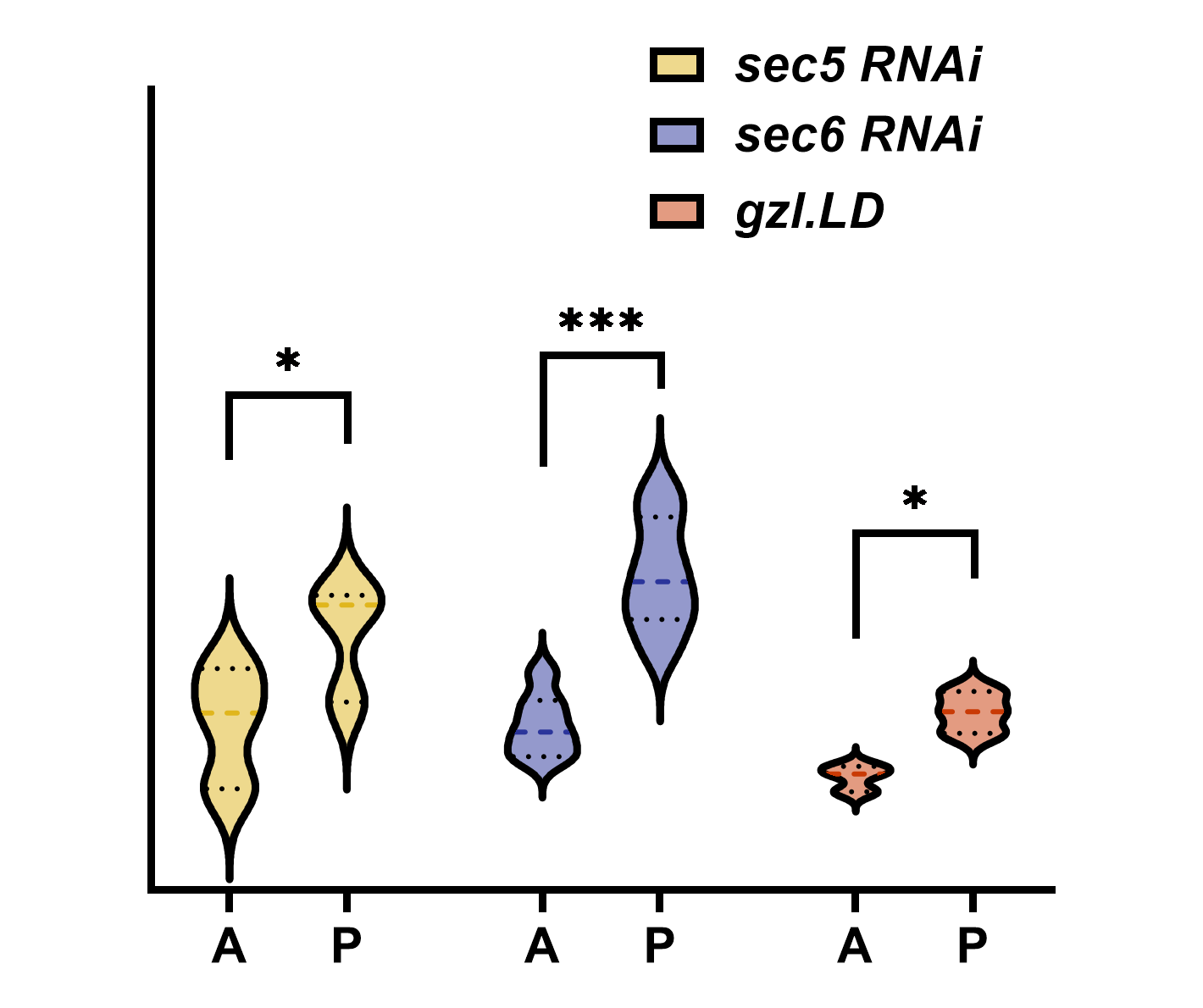

Supplement: Supplementary file 9 — EV Figures Source Data [file 44319_2024_289_MOESM9_ESM.zip › Figure EV4/EV4C-EV4F/Figure EV4C Basal Wg 2.tif]

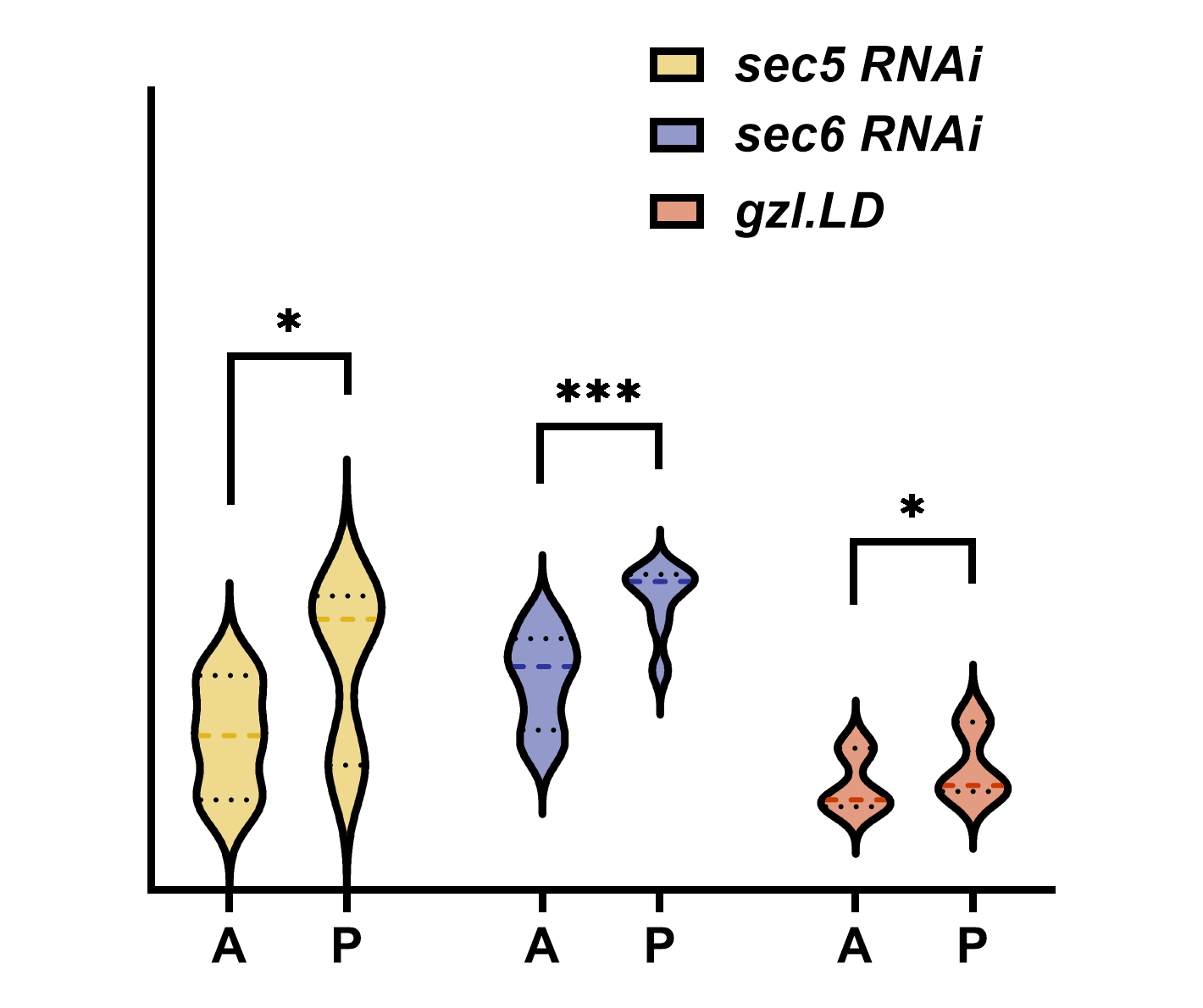

Supplement: Supplementary file 9 — EV Figures Source Data [file 44319_2024_289_MOESM9_ESM.zip › Figure EV4/EV4C-EV4F/Figure EV4D Basal Wls 2.tif]

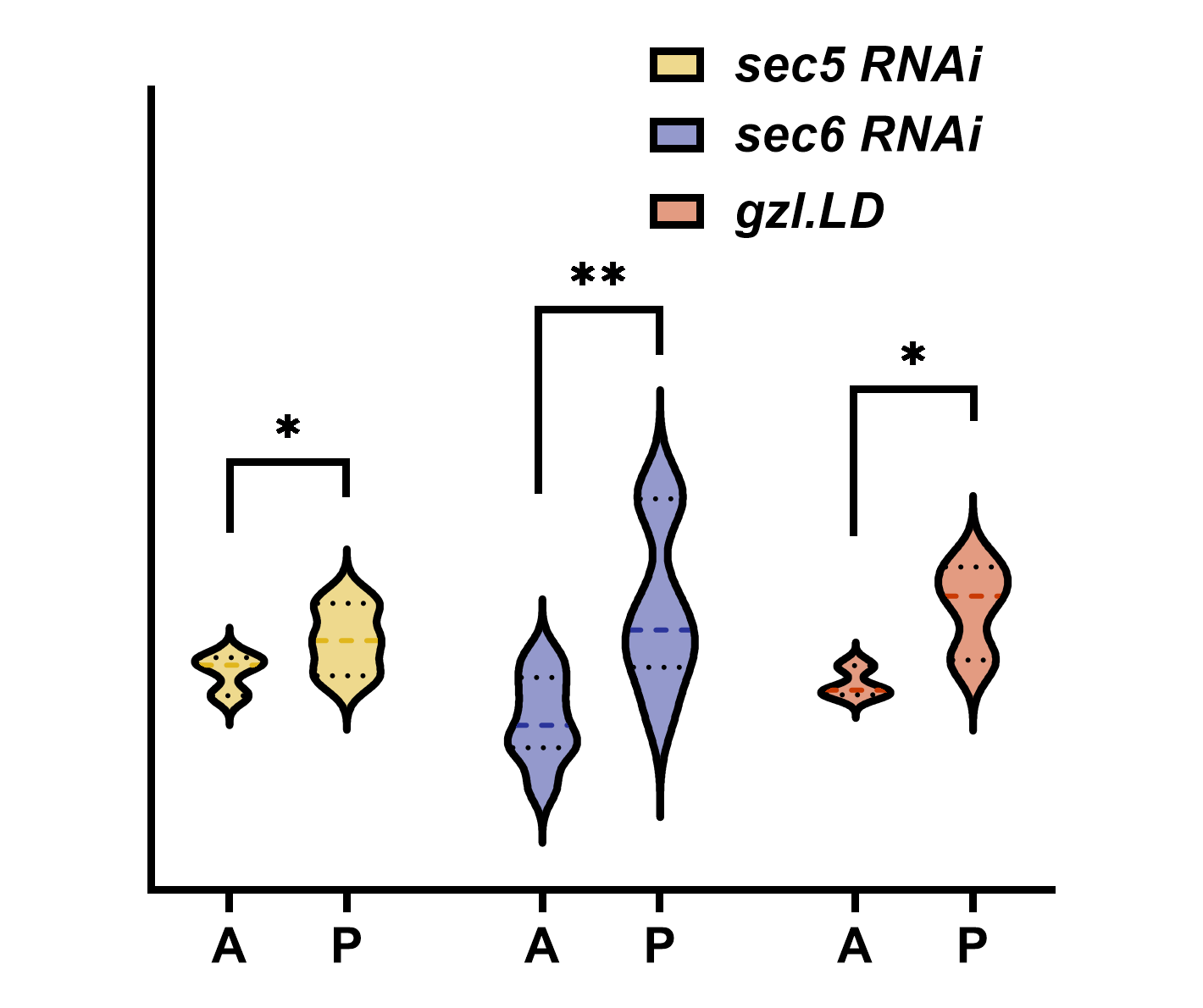

Supplement: Supplementary file 9 — EV Figures Source Data [file 44319_2024_289_MOESM9_ESM.zip › Figure EV4/EV4C-EV4F/Figure EV4E Apical Wg 2.tif]

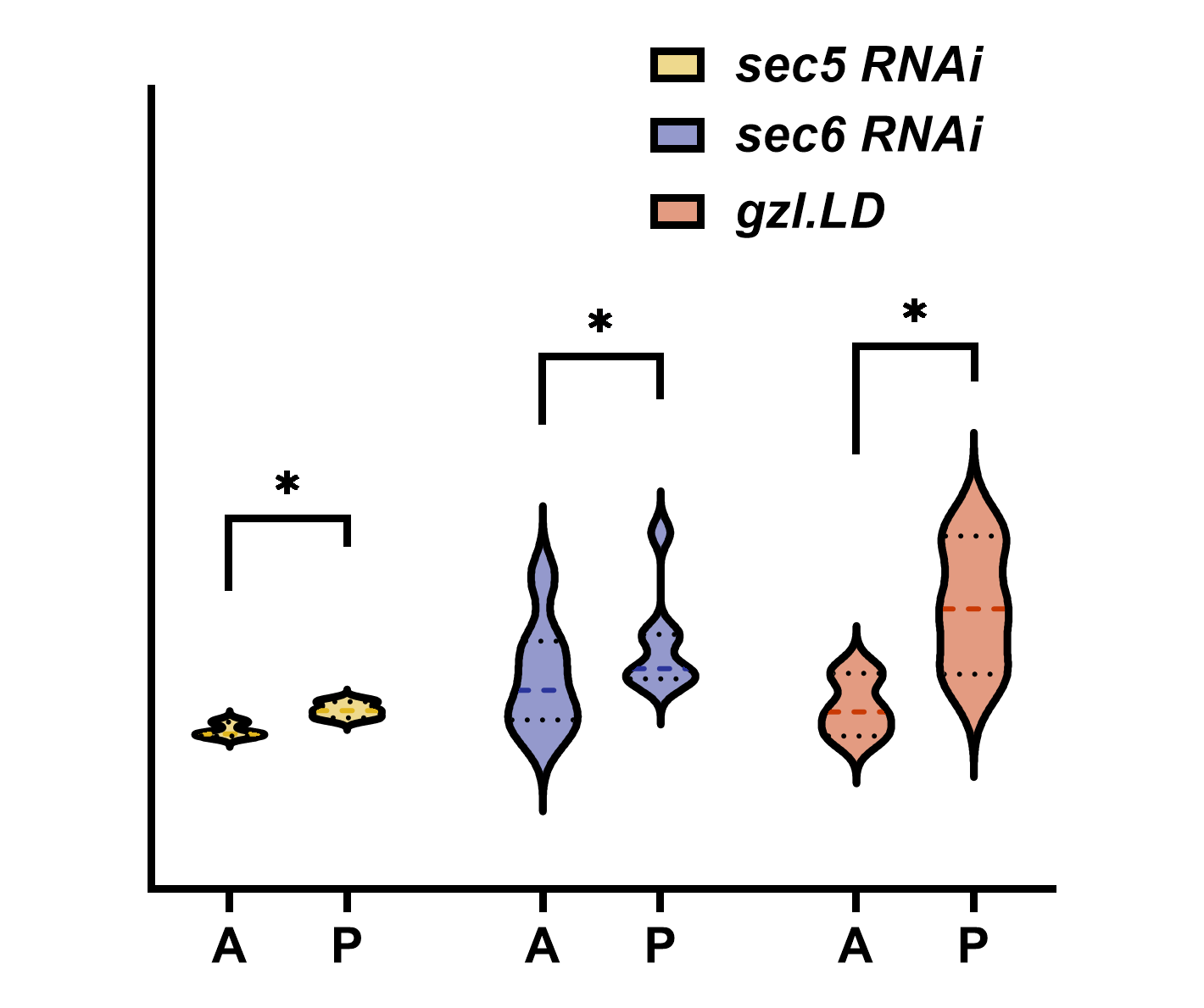

Supplement: Supplementary file 9 — EV Figures Source Data [file 44319_2024_289_MOESM9_ESM.zip › Figure EV4/EV4C-EV4F/Figure EV4F Apical Wls 2.tif]

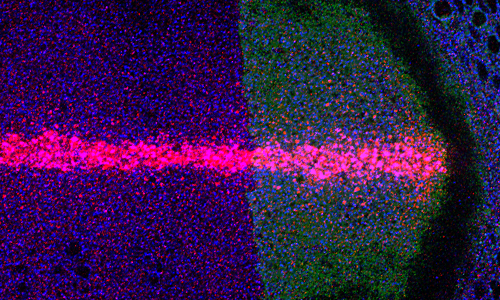

Supplement: Supplementary file 9 — EV Figures Source Data [file 44319_2024_289_MOESM9_ESM.zip › Figure EV4/EV4C-EV4F/Figures for statistical analysis in Figure EV4C to F/Gzd ld/200420 Apical 1 ts-Gal80 hh-G4-GFP UAS-God LD wg wls_Series001_Lng_global_z019.tif]

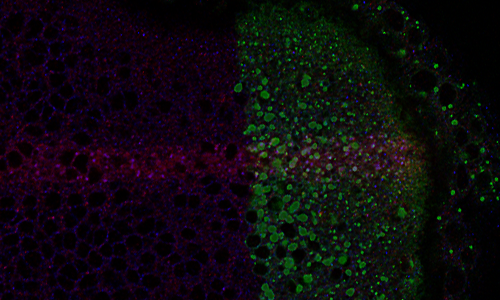

Supplement: Supplementary file 9 — EV Figures Source Data [file 44319_2024_289_MOESM9_ESM.zip › Figure EV4/EV4C-EV4F/Figures for statistical analysis in Figure EV4C to F/Gzd ld/200420 Apical 2 ts-Gal80 hh-G4-GFP UAS-God LD wg wls_Series002_Lng_global_000_z040.tif]

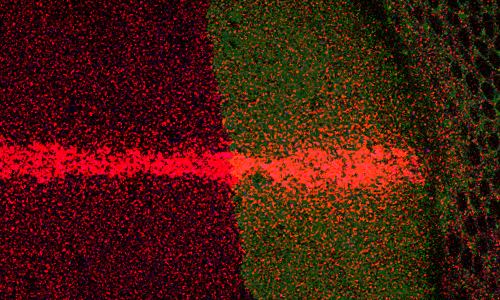

Supplement: Supplementary file 9 — EV Figures Source Data [file 44319_2024_289_MOESM9_ESM.zip › Figure EV4/EV4C-EV4F/Figures for statistical analysis in Figure EV4C to F/Gzd ld/200420 Apical 3 ts-Gal80 hh-G4-GFP UAS-God LD wg wls_Series002_Lng_global_z015.tif]

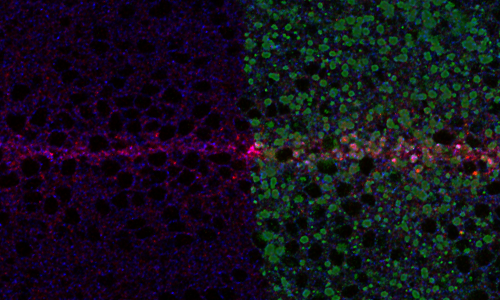

Supplement: Supplementary file 9 — EV Figures Source Data [file 44319_2024_289_MOESM9_ESM.zip › Figure EV4/EV4C-EV4F/Figures for statistical analysis in Figure EV4C to F/Gzd ld/200420 Basal 1 ts-Gal80 hh-G4-GFP UAS-God LD wg wls_Series001_Lng_global_z125.tif]

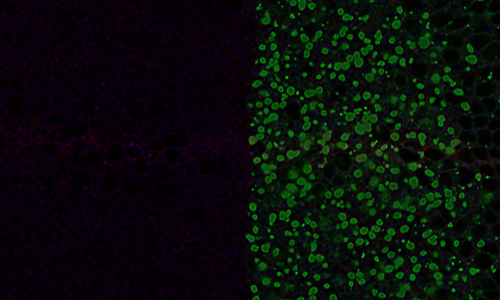

Supplement: Supplementary file 9 — EV Figures Source Data [file 44319_2024_289_MOESM9_ESM.zip › Figure EV4/EV4C-EV4F/Figures for statistical analysis in Figure EV4C to F/Gzd ld/200420 Basal 2 ts-Gal80 hh-G4-GFP UAS-God LD wg wls_Series002_Lng_global_000_z115.tif]

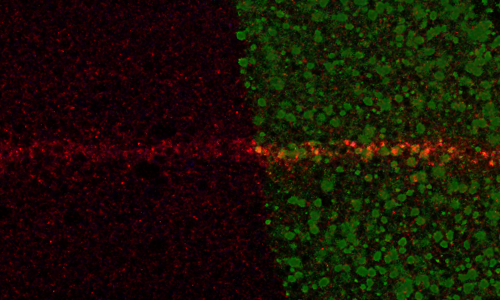

Supplement: Supplementary file 9 — EV Figures Source Data [file 44319_2024_289_MOESM9_ESM.zip › Figure EV4/EV4C-EV4F/Figures for statistical analysis in Figure EV4C to F/Gzd ld/200420 Basal 3 ts-Gal80 hh-G4-GFP UAS-God LD wg wls_Series002_Lng_global_z130.tif]

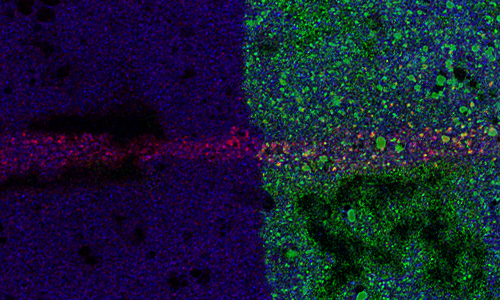

Supplement: Supplementary file 9 — EV Figures Source Data [file 44319_2024_289_MOESM9_ESM.zip › Figure EV4/EV4C-EV4F/Figures for statistical analysis in Figure EV4C to F/Gzd ld/20240503 Apical 4 ts-Gal80 hh-G4-GFP Gzl LD Wg Wls 2_Series002_Lng_adaptive_z015.tif]

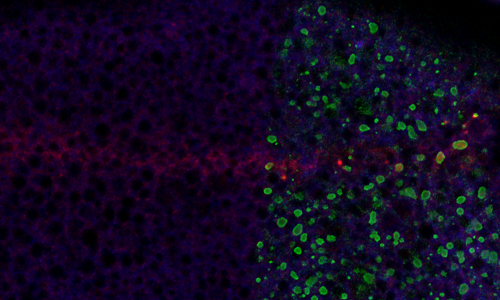

Supplement: Supplementary file 9 — EV Figures Source Data [file 44319_2024_289_MOESM9_ESM.zip › Figure EV4/EV4C-EV4F/Figures for statistical analysis in Figure EV4C to F/Gzd ld/20240503 Basal 4 ts-Gal80 hh-G4-GFP Gzl LD Wg Wls 2_Series002_Lng_adaptive_z106.tif]

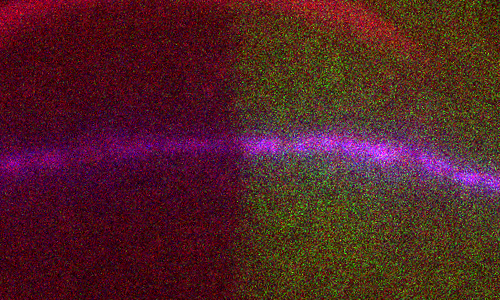

Supplement: Supplementary file 9 — EV Figures Source Data [file 44319_2024_289_MOESM9_ESM.zip › Figure EV4/EV4C-EV4F/Figures for statistical analysis in Figure EV4C to F/sec5 RNAi/20230811 hh-Gal4 Exocyst RNA Wls Wg_20230811 24C hh-Gal4 TH2688 -1 -z_z007.tif]

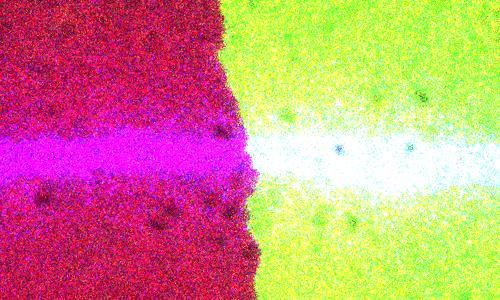

Supplement: Supplementary file 9 — EV Figures Source Data [file 44319_2024_289_MOESM9_ESM.zip › Figure EV4/EV4C-EV4F/Figures for statistical analysis in Figure EV4C to F/sec5 RNAi/20230811 hh-Gal4 Exocyst RNA Wls Wg_20230811 24C hh-Gal4 TH2688 -1 -z_z116.tif]

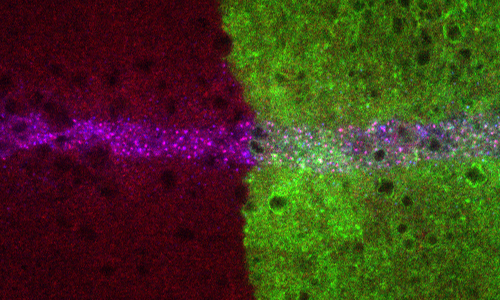

Supplement: Supplementary file 9 — EV Figures Source Data [file 44319_2024_289_MOESM9_ESM.zip › Figure EV4/EV4C-EV4F/Figures for statistical analysis in Figure EV4C to F/sec5 RNAi/20230811 hh-Gal4 Exocyst RNA Wls Wg_20230811 24C hh-Gal4 TH2688 -2 -A.tif]

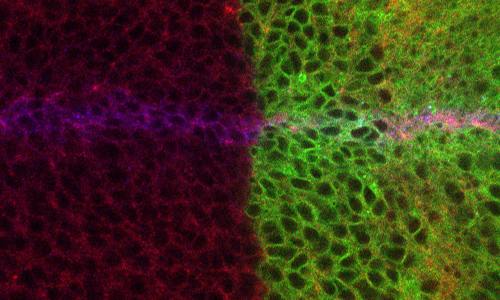

Supplement: Supplementary file 9 — EV Figures Source Data [file 44319_2024_289_MOESM9_ESM.zip › Figure EV4/EV4C-EV4F/Figures for statistical analysis in Figure EV4C to F/sec5 RNAi/20230811 hh-Gal4 Exocyst RNA Wls Wg_20230811 24C hh-Gal4 TH2688 -2 -B.tif]

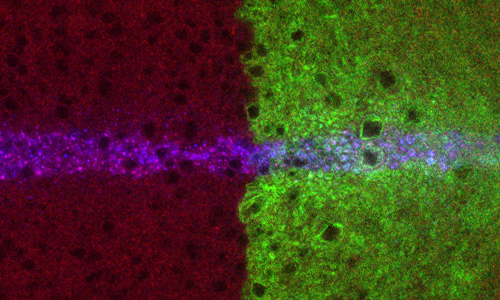

Supplement: Supplementary file 9 — EV Figures Source Data [file 44319_2024_289_MOESM9_ESM.zip › Figure EV4/EV4C-EV4F/Figures for statistical analysis in Figure EV4C to F/sec5 RNAi/20230811 hh-Gal4 Exocyst RNA Wls Wg_20230811 24C hh-Gal4 TH2688 -3 -A.tif]

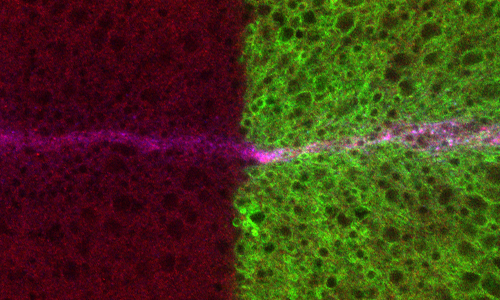

Supplement: Supplementary file 9 — EV Figures Source Data [file 44319_2024_289_MOESM9_ESM.zip › Figure EV4/EV4C-EV4F/Figures for statistical analysis in Figure EV4C to F/sec5 RNAi/20230811 hh-Gal4 Exocyst RNA Wls Wg_20230811 24C hh-Gal4 TH2688 -3 -B.tif]

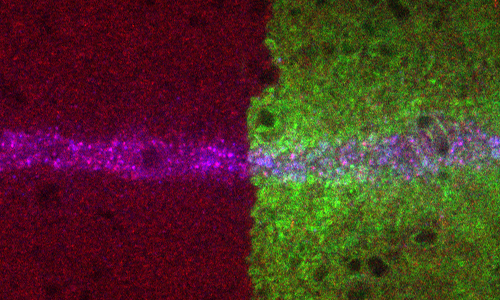

Supplement: Supplementary file 9 — EV Figures Source Data [file 44319_2024_289_MOESM9_ESM.zip › Figure EV4/EV4C-EV4F/Figures for statistical analysis in Figure EV4C to F/sec6 RNAi/20230811 hh-Gal4 Exocyst RNA Wls Wg_20230812 24C hh-Gal4 Th2636 -1 -A.tif]
