# Supplementary figures and images for: Ehbp1 orchestrates orderly sorting of Wnt/Wingless to the basolateral and apical cell membranes (part 3 of 3)
Source: EMBO Rep. 2024 Oct 14;25(11):5053–79. doi: 10.1038/s44319-024-00289-1 (PMC11549480; doi:10.1038/s44319-024-00289-1)

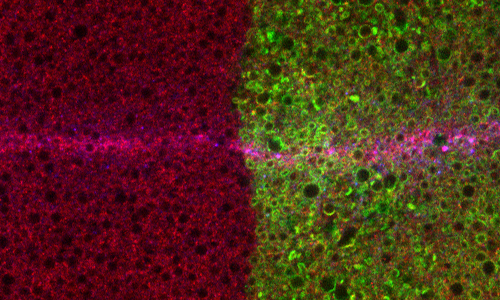

Supplement: Supplementary file 9 — EV Figures Source Data [file 44319_2024_289_MOESM9_ESM.zip › Figure EV4/EV4C-EV4F/Figures for statistical analysis in Figure EV4C to F/sec6 RNAi/20230811 hh-Gal4 Exocyst RNA Wls Wg_20230812 24C hh-Gal4 Th2636 -1 -B.tif]

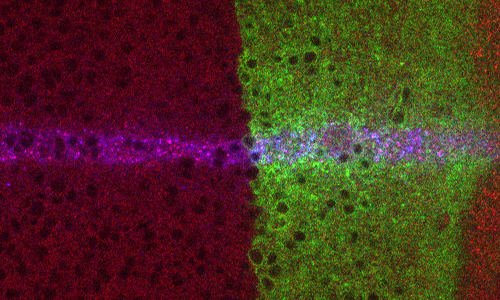

Supplement: Supplementary file 9 — EV Figures Source Data [file 44319_2024_289_MOESM9_ESM.zip › Figure EV4/EV4C-EV4F/Figures for statistical analysis in Figure EV4C to F/sec6 RNAi/20230811 hh-Gal4 Exocyst RNA Wls Wg_20230812 24C hh-Gal4 Th2636 -2 -A.tif]

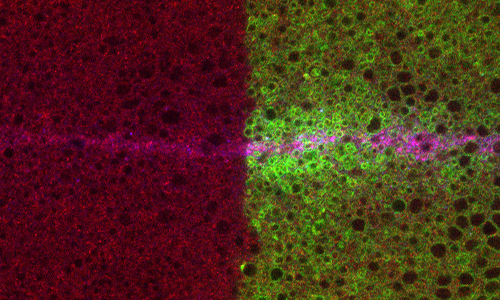

Supplement: Supplementary file 9 — EV Figures Source Data [file 44319_2024_289_MOESM9_ESM.zip › Figure EV4/EV4C-EV4F/Figures for statistical analysis in Figure EV4C to F/sec6 RNAi/20230811 hh-Gal4 Exocyst RNA Wls Wg_20230812 24C hh-Gal4 Th2636 -2 -B.tif]

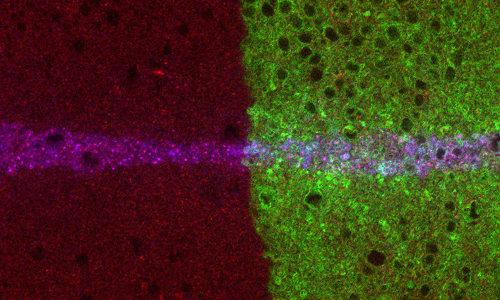

Supplement: Supplementary file 9 — EV Figures Source Data [file 44319_2024_289_MOESM9_ESM.zip › Figure EV4/EV4C-EV4F/Figures for statistical analysis in Figure EV4C to F/sec6 RNAi/20230811 hh-Gal4 Exocyst RNA Wls Wg_20230812 24C hh-Gal4 Th2636 -3 -A.tif]

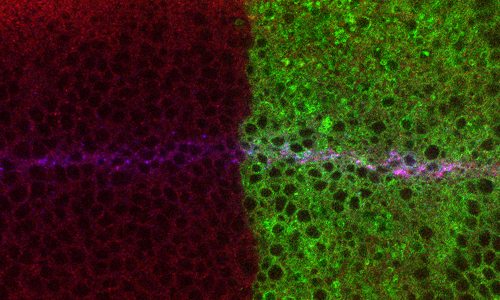

Supplement: Supplementary file 9 — EV Figures Source Data [file 44319_2024_289_MOESM9_ESM.zip › Figure EV4/EV4C-EV4F/Figures for statistical analysis in Figure EV4C to F/sec6 RNAi/20230811 hh-Gal4 Exocyst RNA Wls Wg_20230812 24C hh-Gal4 Th2636 -3 -B.tif]

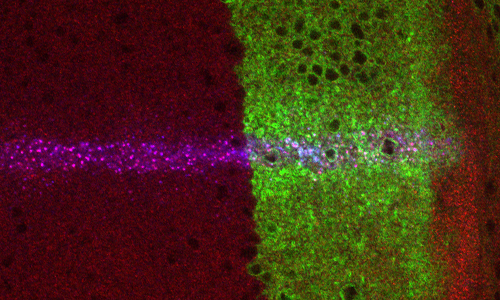

Supplement: Supplementary file 9 — EV Figures Source Data [file 44319_2024_289_MOESM9_ESM.zip › Figure EV4/EV4C-EV4F/Figures for statistical analysis in Figure EV4C to F/sec6 RNAi/20230811 hh-Gal4 Exocyst RNA Wls Wg_20230812 24C hh-Gal4 Th2636 -4 -A.tif]

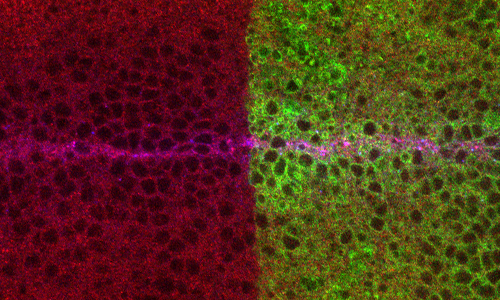

Supplement: Supplementary file 9 — EV Figures Source Data [file 44319_2024_289_MOESM9_ESM.zip › Figure EV4/EV4C-EV4F/Figures for statistical analysis in Figure EV4C to F/sec6 RNAi/20230811 hh-Gal4 Exocyst RNA Wls Wg_20230812 24C hh-Gal4 Th2636 -4 -B.tif]

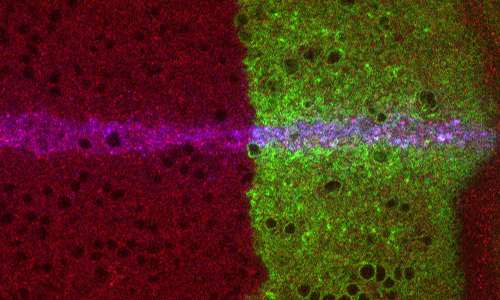

Supplement: Supplementary file 9 — EV Figures Source Data [file 44319_2024_289_MOESM9_ESM.zip › Figure EV4/EV4C-EV4F/Figures for statistical analysis in Figure EV4C to F/sec6 RNAi/20230811 hh-Gal4 Exocyst RNA Wls Wg_20230812 24C hh-Gal4 Th2636 -5 -A.tif]

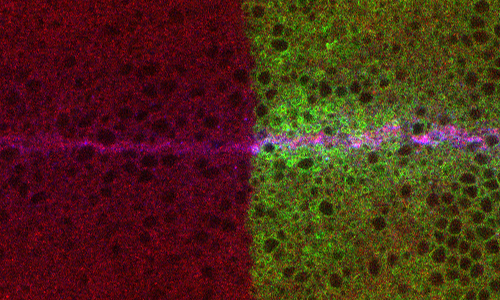

Supplement: Supplementary file 9 — EV Figures Source Data [file 44319_2024_289_MOESM9_ESM.zip › Figure EV4/EV4C-EV4F/Figures for statistical analysis in Figure EV4C to F/sec6 RNAi/20230811 hh-Gal4 Exocyst RNA Wls Wg_20230812 24C hh-Gal4 Th2636 -5 -B.tif]

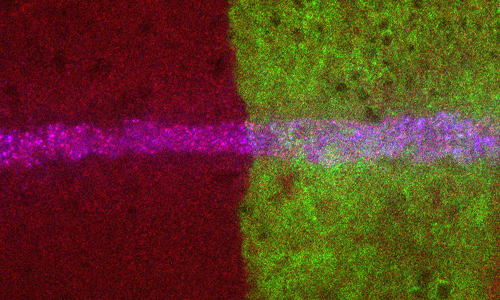

Supplement: Supplementary file 9 — EV Figures Source Data [file 44319_2024_289_MOESM9_ESM.zip › Figure EV4/EV4C-EV4F/Figures for statistical analysis in Figure EV4C to F/sec6 RNAi/20230811 hh-Gal4 Exocyst RNA Wls Wg_20230812 24C hh-Gal4 Th2636 -6 -A.tif]

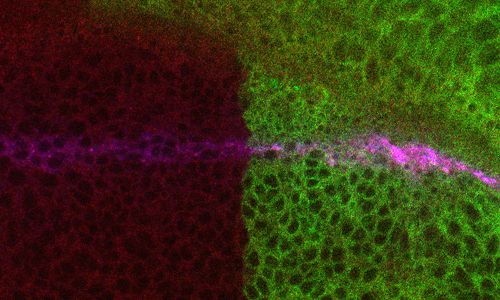

Supplement: Supplementary file 9 — EV Figures Source Data [file 44319_2024_289_MOESM9_ESM.zip › Figure EV4/EV4C-EV4F/Figures for statistical analysis in Figure EV4C to F/sec6 RNAi/20230811 hh-Gal4 Exocyst RNA Wls Wg_20230812 24C hh-Gal4 Th2636 -6 -B.tif]

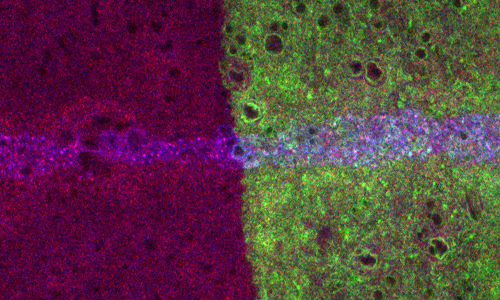

Supplement: Supplementary file 9 — EV Figures Source Data [file 44319_2024_289_MOESM9_ESM.zip › Figure EV4/EV4C-EV4F/Figures for statistical analysis in Figure EV4C to F/sec6 RNAi/20230811 hh-Gal4 Exocyst RNA Wls Wg_20230815 24C hh-Gal4 sec6 Th2636 -1 -A - ╕▒▒╛.tif]

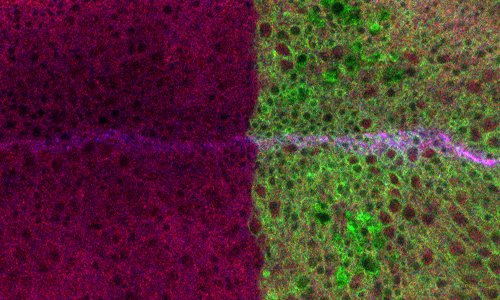

Supplement: Supplementary file 9 — EV Figures Source Data [file 44319_2024_289_MOESM9_ESM.zip › Figure EV4/EV4C-EV4F/Figures for statistical analysis in Figure EV4C to F/sec6 RNAi/20230811 hh-Gal4 Exocyst RNA Wls Wg_20230815 24C hh-Gal4 sec6 Th2636 -1 -B - ╕▒▒╛.tif]

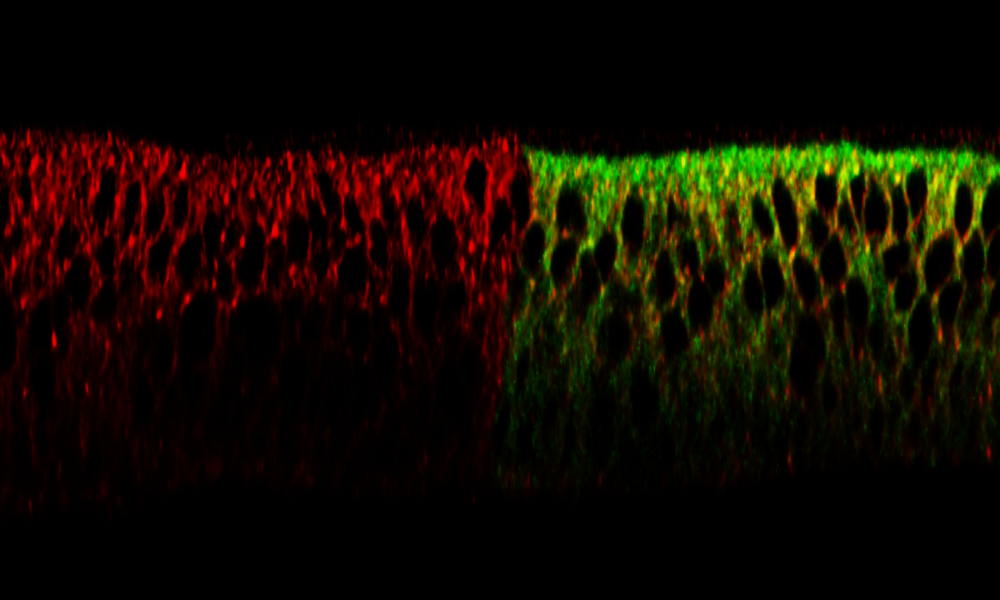

Supplement: Supplementary file 9 — EV Figures Source Data [file 44319_2024_289_MOESM9_ESM.zip › Figure EV5/EV5A/EV5A hh-Gal4 Rab8-CA Wg.tif]

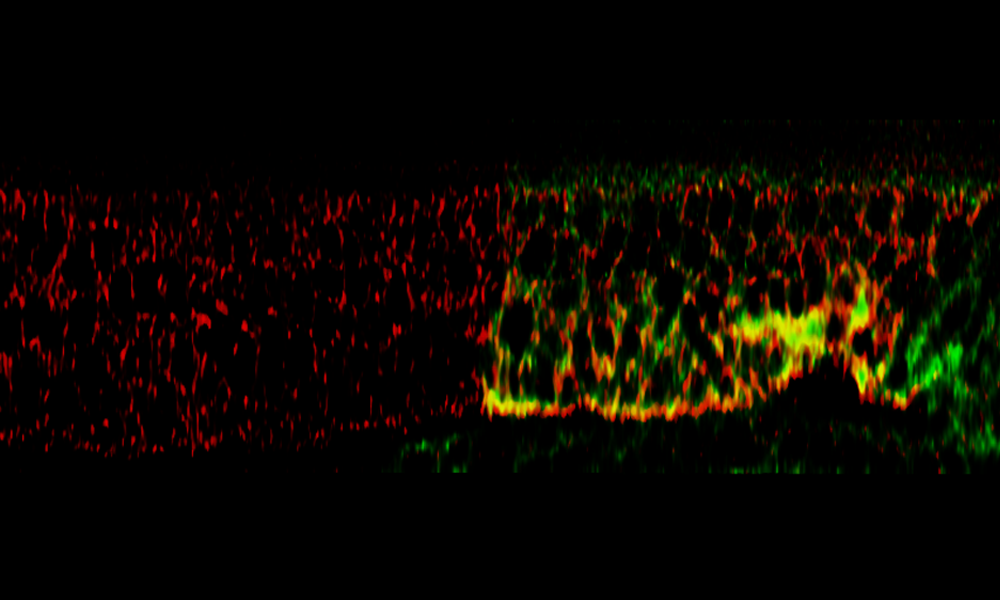

Supplement: Supplementary file 9 — EV Figures Source Data [file 44319_2024_289_MOESM9_ESM.zip › Figure EV5/EV5B/EV5B hh-Gal4 Rab8-CA Ehbp1RNAi Wg.tif]

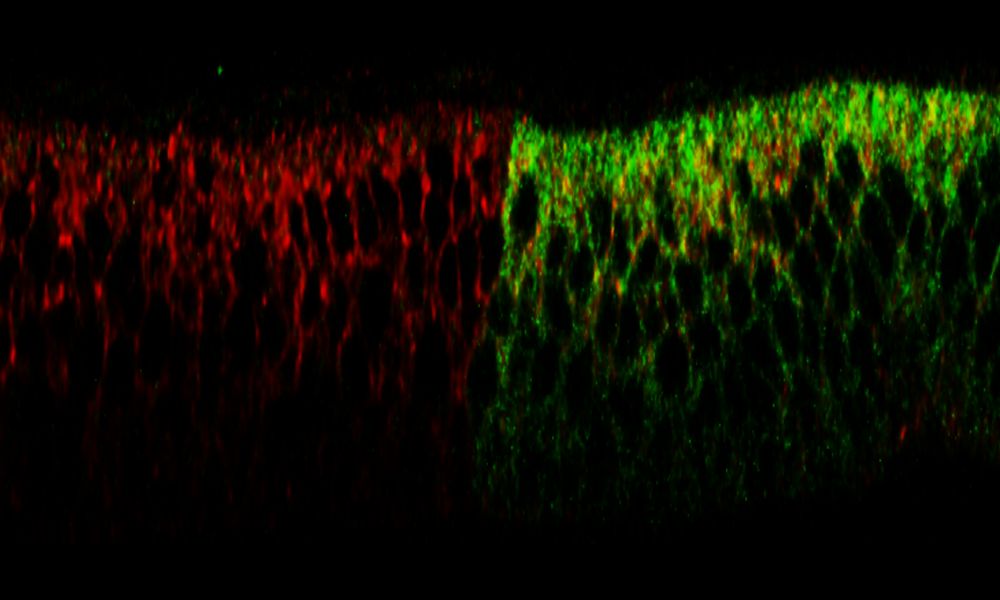

Supplement: Supplementary file 9 — EV Figures Source Data [file 44319_2024_289_MOESM9_ESM.zip › Figure EV5/EV5C/EV5C hh-Gal4 Rab8-DN Wg.tif]

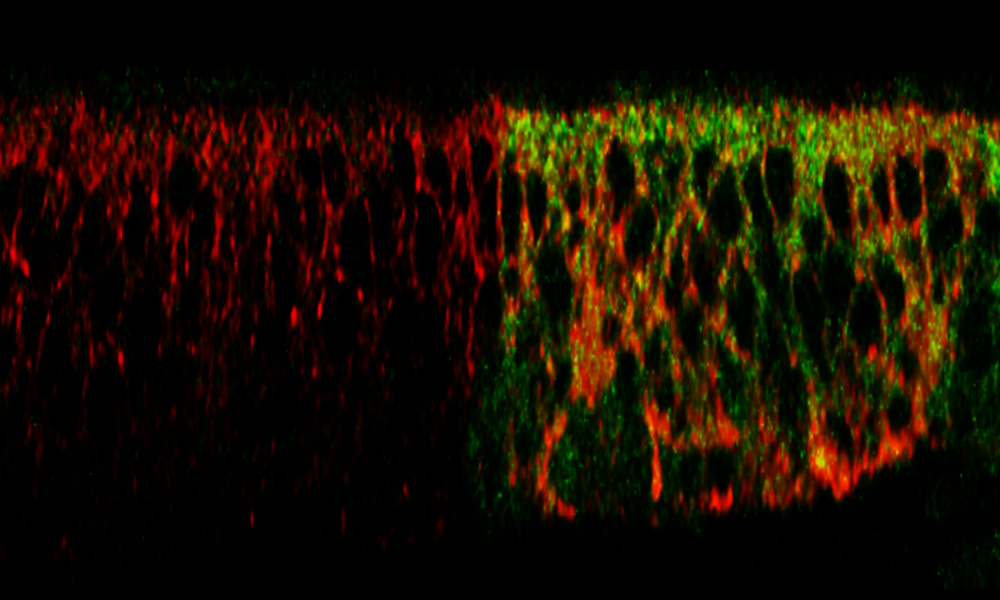

Supplement: Supplementary file 9 — EV Figures Source Data [file 44319_2024_289_MOESM9_ESM.zip › Figure EV5/EV5D/EV5D hh-Gal4 Rab8-DN Ehbp1RNAi Wg.tif]

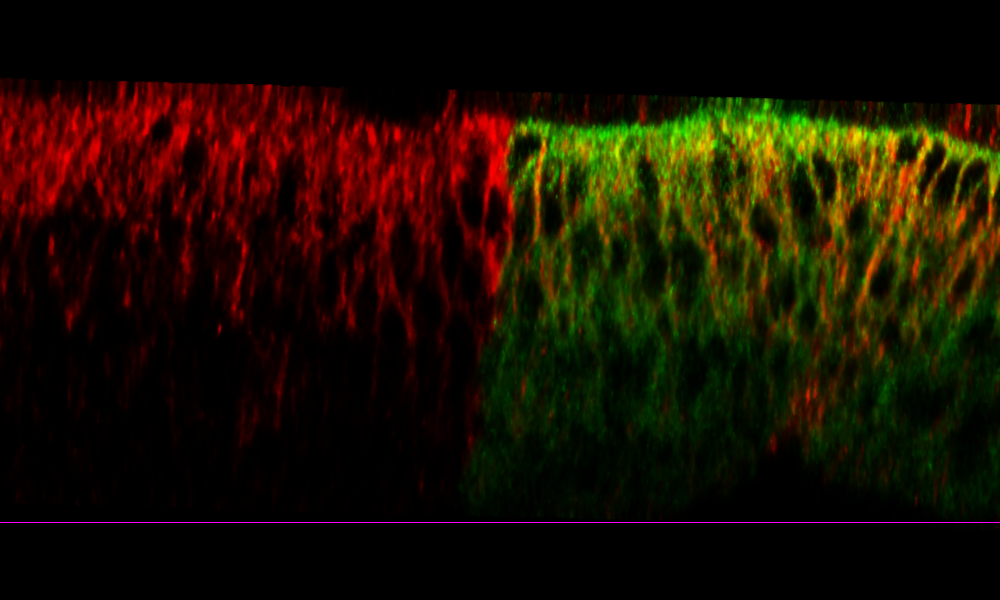

Supplement: Supplementary file 9 — EV Figures Source Data [file 44319_2024_289_MOESM9_ESM.zip › Figure EV5/EV5E/EV5E hh-Gal4 Rab10-CA Wg.tif]

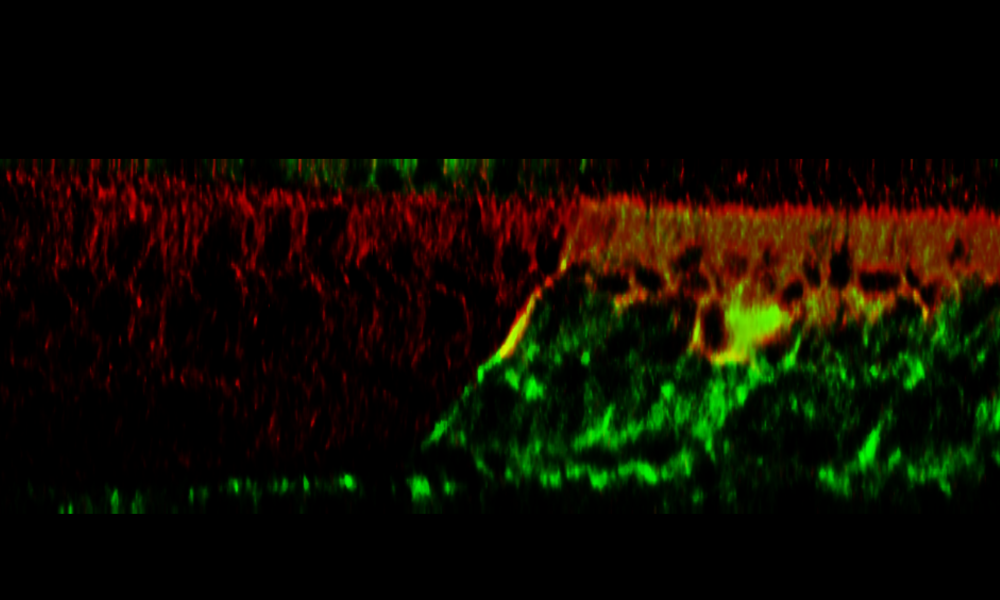

Supplement: Supplementary file 9 — EV Figures Source Data [file 44319_2024_289_MOESM9_ESM.zip › Figure EV5/EV5F/EV5F hh-Gal4 Rab10-CA Ehbp1RNAi Wg.tif]

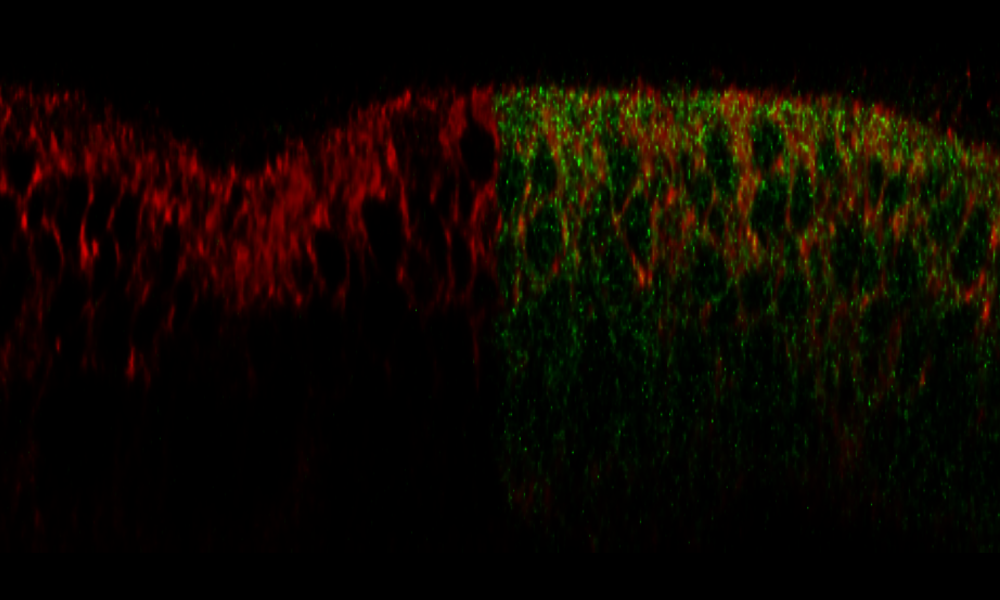

Supplement: Supplementary file 9 — EV Figures Source Data [file 44319_2024_289_MOESM9_ESM.zip › Figure EV5/EV5G/EV5G hh-Gal4 Rab10-DN Wg.tif]

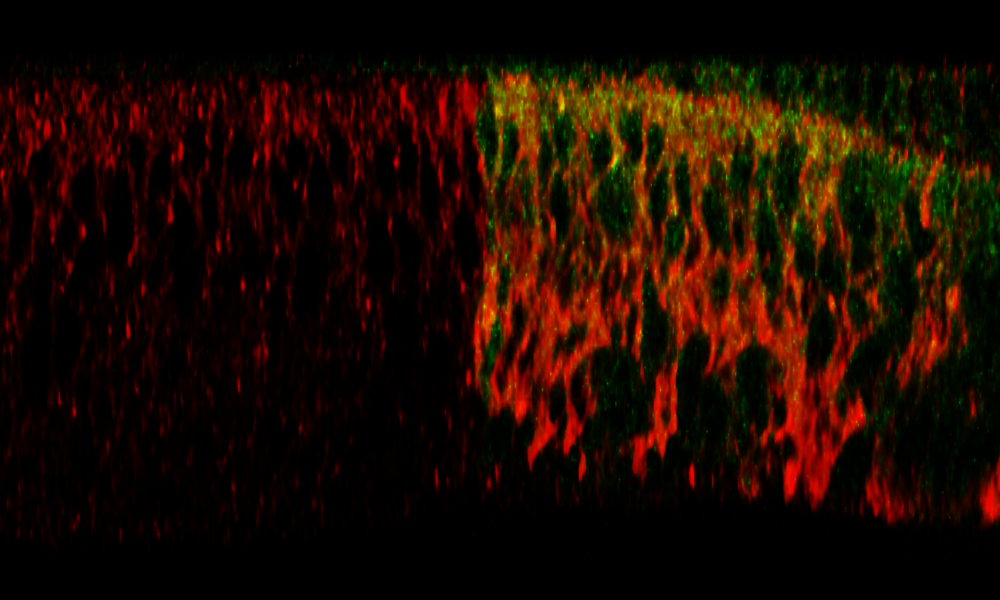

Supplement: Supplementary file 9 — EV Figures Source Data [file 44319_2024_289_MOESM9_ESM.zip › Figure EV5/EV5H/EV5H hh-Gal4 Rab10-DN Ehbp1RNAi Wg.tif]
