# Supplementary material for: Ehbp1 orchestrates orderly sorting of Wnt/Wingless to the basolateral and apical cell membranes
Source: EMBO Rep. 2024 Oct 14;25(11):5053–79. doi: 10.1038/s44319-024-00289-1 (PMC11549480; doi:10.1038/s44319-024-00289-1)
Supplement: Supplementary file 10 — Expanded View Figures [file 44319_2024_289_MOESM10_ESM.pdf]

Expanded View Figures

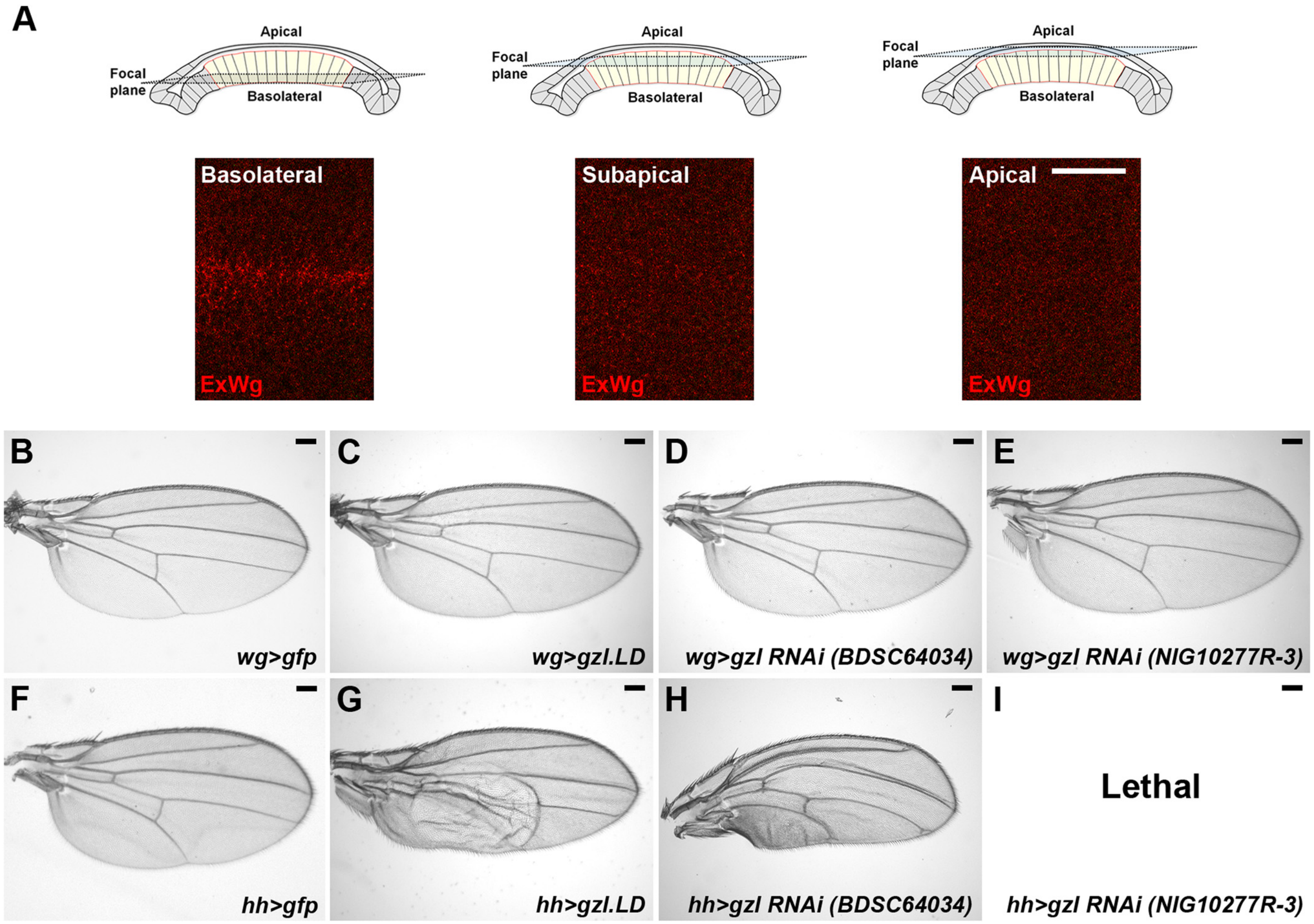

Figure EV1. Disruption of Gzl-mediated transcytosis has no impact on wing margin development.

(**A**) The basolateral (left), subapical (middle), and apical (right) membrane domains of immunofluorescence staining of extracellular Wg (ExWg) for the wild-type wing imaginal discs are shown. (**B**) Shown is a wild-type adult wing expressing the *wg-Gal4* driver. (**C–E**) When a dominant-negative *gzl.LD* (**C**) or RNAi against *gzl* (targeting distinct regions of the *gzl* locus, **D** and **E**) was expressed using the *wg-Gal4* driver, no noticeable defects were observed in wing margin development. (**F**) Shown is a wild-type adult wing expressing the *hh-Gal4* driver. (**G–I**) When a dominant-negative *gzl.LD* (**G**) or RNAi against *gzl* (targeting distinct regions of the *gzl* locus, **H** and **I**) was expressed using the *hh-Gal4* driver, the resulting phenotypes include lethality in the adult fly (**I**) or disruptions in the development of the posterior compartment of the adult wing blade (**G** and **H**). Despite these effects, the wing margin remained unaffected, showing no apparent defects. Scale bars: (**A**) 25 µm; (**B–I**) 100 µm. Source data are available online for this figure.

© The Author(s)

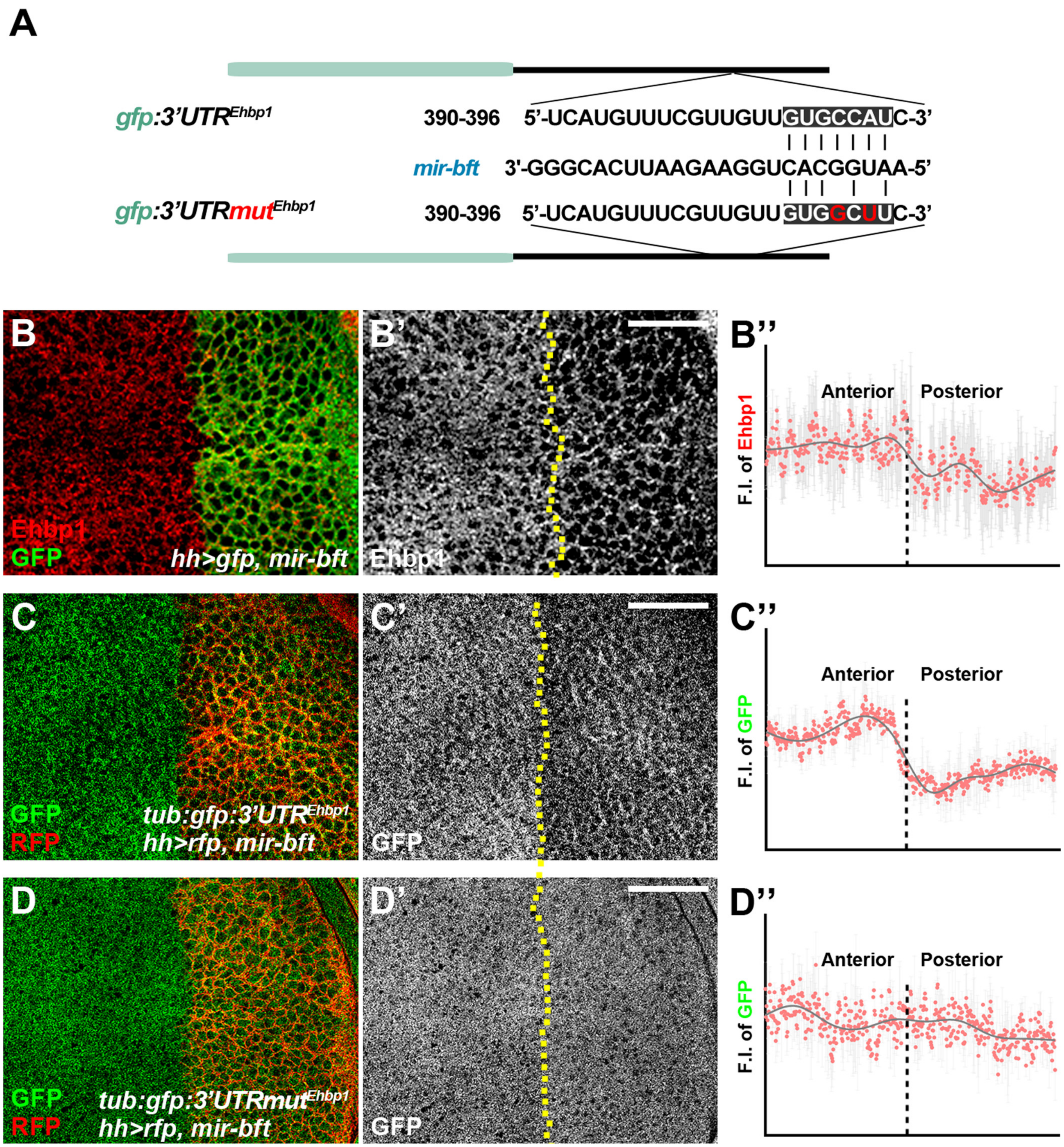

Figure EV2. ***Ehbp1* is a bona-fide target of *mir-bft*.**

(**A**) Shown is the strategy for constructing the *mir-bft* GFP sensor. The 3′UTR of *Ehbp1* was cloned with GFP coding sequence to create the *tub:gfp:3′UTR[Ehbp1]* sensor. The predicted target sequence for *mir-bft*, GUGCCAU, was mutated to GUGGCUU in the mutated *tub:gfp:3′UTRmut[Ehbp1]* sensor. (**B–B″**) Upon overexpression of *mir-bft* using the *hh-Gal4* driver, a significant reduction in Ehbp1 was observed in the posterior compartment of the wing disc (**B**, **B′**), and a plot profile of immunofluorescence staining in (**B′**) was generated (**B″**) (for each genotype, $n \geq 3$ biological replicates). Data are shown as mean ± SD. In these and all subsequent figures, wing discs are oriented with the anterior to the left and dorsal down, the anterior-posterior boundaries are indicated by dotted yellow lines. (**C–D″**) When *mir-bft* was overexpressed using the *hh-Gal4* driver, there was a reduction in the expression of *tub:gfp:3′UTR[Ehbp1]* sensor in the posterior compartment of the wing disc (**C–C′**). In contrast, the expression levels of *tub:gfp:3′UTRmut[Ehbp1]* sensor remained largely unchanged. Plot profiles of immunofluorescence staining in (**C′** and **D′**) were generated (**C″** and **D″**) (for each genotype, $n \geq 3$ biological replicates). Data are shown as mean ± SD. Scale bars, 25 μm. Source data are available online for this figure.

 © The Author(s)

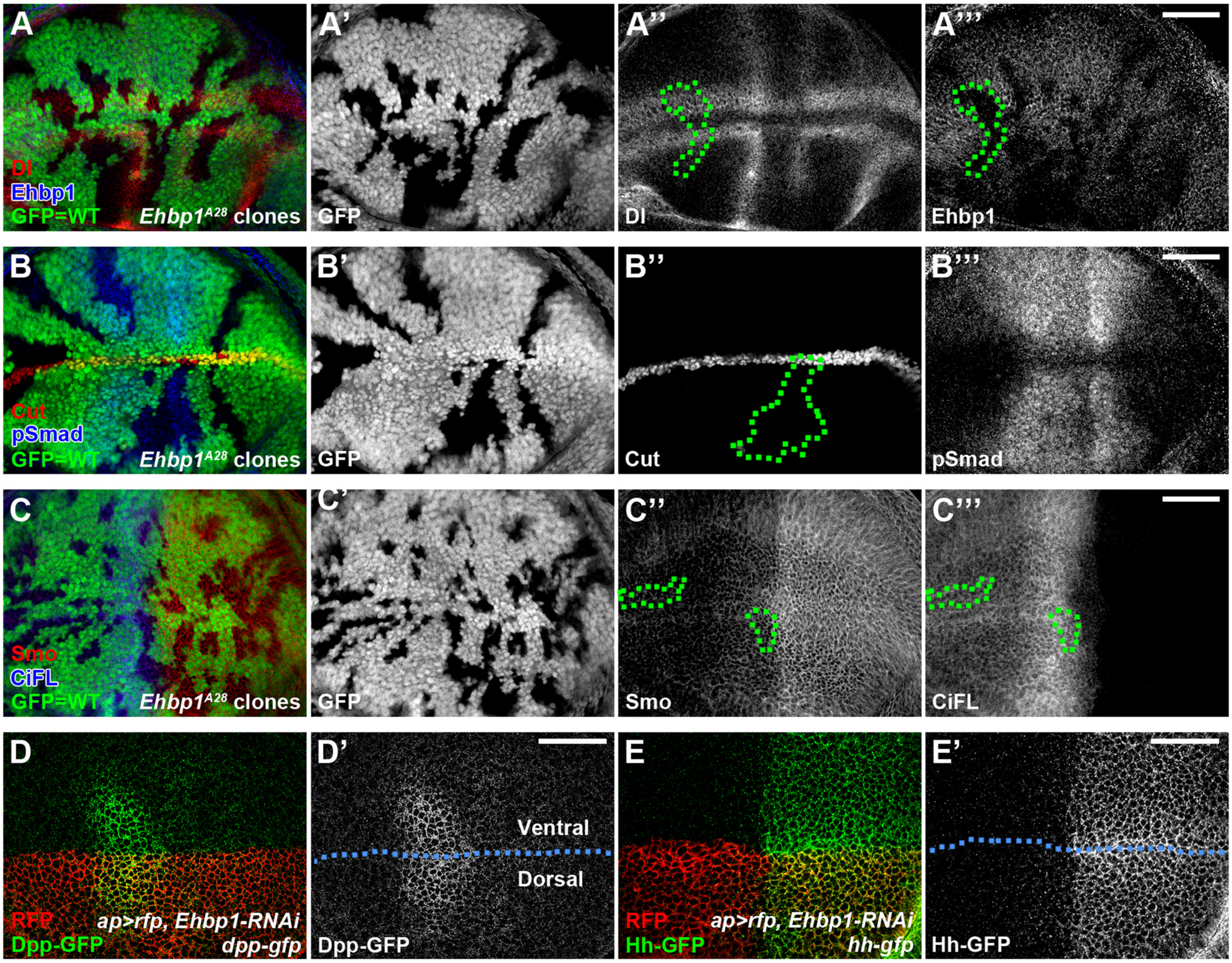

Figure EV3. Ehbp1 does not play a role in the Notch, Dpp, or Hh signaling pathways during wing development.

(**A–C'''**) In negatively marked *Ehbp1*^{A28} loss-of-function mutant clones (marked by the absence of GFP, outlined by dotted green lines), there were no obvious changes in the levels of the Notch signaling ligand Delta (Dl), the Notch signaling target Cut, the phosphorylation of the Dpp signaling activator Mothers against dpp (Mad/Smad), the Hh signaling activator Smoothened (Smo), or the activation of Hh signaling transcriptional factor Cubitus interruptus (CiFL). (**D–E'**) When RNAi against *Ehbp1* was expressed in the dorsal compartment of the wing discs using the *apterous* (*ap*)*-Gal4* driver, no alterations were observed in the distribution or levels of the morphogens Dpp or Hh, as indicated by GFP-trapped Dpp or Hh. The dotted blue lines indicate the D-V boundaries. Scale bars, 50 μm. Source data are available online for this figure.

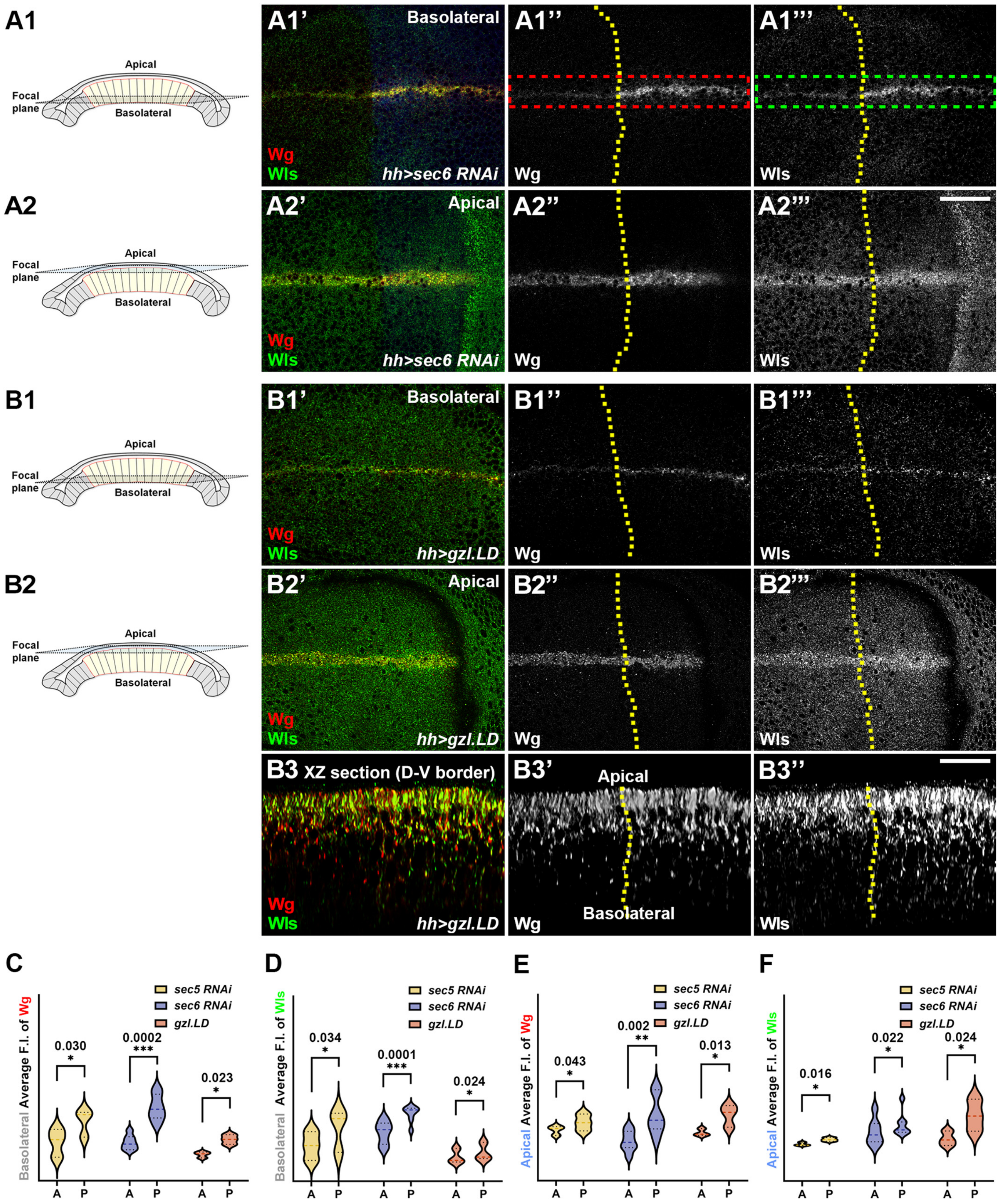
A1
Apical
Focal plane
Basolateral
A1'
Basolateral
Wg
Wls
hh>sec6 RNAi
A1''
Wg
A1'''
Wls
A2
Apical
Focal plane
Basolateral
A2'
Apical
Wg
Wls
hh>sec6 RNAi
A2''
Wg
A2'''
Wls
B1
Apical
Focal plane
Basolateral
B1'
Basolateral
Wg
Wls
hh>gzl.LD
B1''
Wg
B1'''
Wls
B2
Apical
Focal plane
Basolateral
B2'
Apical
Wg
Wls
hh>gzl.LD
B2''
Wg
B2'''
Wls
B3 XZ section (D-V border)
Wg
Wls
hh>gzl.LD
B3'
Apical
Wg
Basolateral
B3''
Wls
C
Basolateral Average F.I. of Wg
sec5 RNAi
sec6 RNAi
gzl.LD
0.030
*
0.0002
***
0.023
*
A P A P A P
D
Basolateral Average F.I. of Wls
sec5 RNAi
sec6 RNAi
gzl.LD
0.034
*
0.0001
***
0.024
*
A P A P A P
E
Apical Average F.I. of Wg
sec5 RNAi
sec6 RNAi
gzl.LD
0.043
*
0.002
**
0.013
*
A P A P A P
F
Apical Average F.I. of Wls
sec5 RNAi
sec6 RNAi
gzl.LD
0.016
*
0.022
*
0.024
*
A P A P A P

 © The Author(s)

◀ **Figure EV4. A transcytosis-independent pathway for basolateral Wg transport involves Wls activity.**

(**A1–B3″**) The basolateral (**A1–A1‴** and **B1–B1‴**) and apical (**A2–A2‴** and **B2–B2‴**) sections of immunofluorescence staining of Wg and Wls in the indicated genotypes are shown. When RNAi against *sec6* (**A1–A2‴**) or a dominant-negative *gzl.LD* (**B1–B2‴**) was expressed using the *hh-Gal4* driver, there was a significant accumulation of both Wls and Wg at the D-V boundary in the basolateral domains of the posterior compartments of the wing discs, while the apical accumulation was noticeably less intense. A 3D reconstruction of the D-V border cells (as viewed in an XZ section) from a wing disc expressing *gzl.LD* shows an enhanced transport of Wls to the basolateral domains (**B3–B3″**). Dotted yellow lines indicate the A-P boundaries. (**C–F**) Statistical analysis of Wg and Wls immunofluorescence intensity (F.I.) at the D-V boundary in both the basolateral and apical membrane domains was performed. The analyzed regions were delineated by rectangles, which were demarcated using red and green dashed lines. An example of this analysis is provided in (**A1–A1‴**). Immunofluorescence intensity data are presented as violin plots (for each genotype, $n \geq 3$ biological replicates). Two-tailed Student's t-tests were employed to analyze the differences between anterior and posterior F.I. *$p < 0.05$. **$p < 0.01$. ***$p < 0.001$. Scale bars, 25 µm. Source data are available online for this figure.

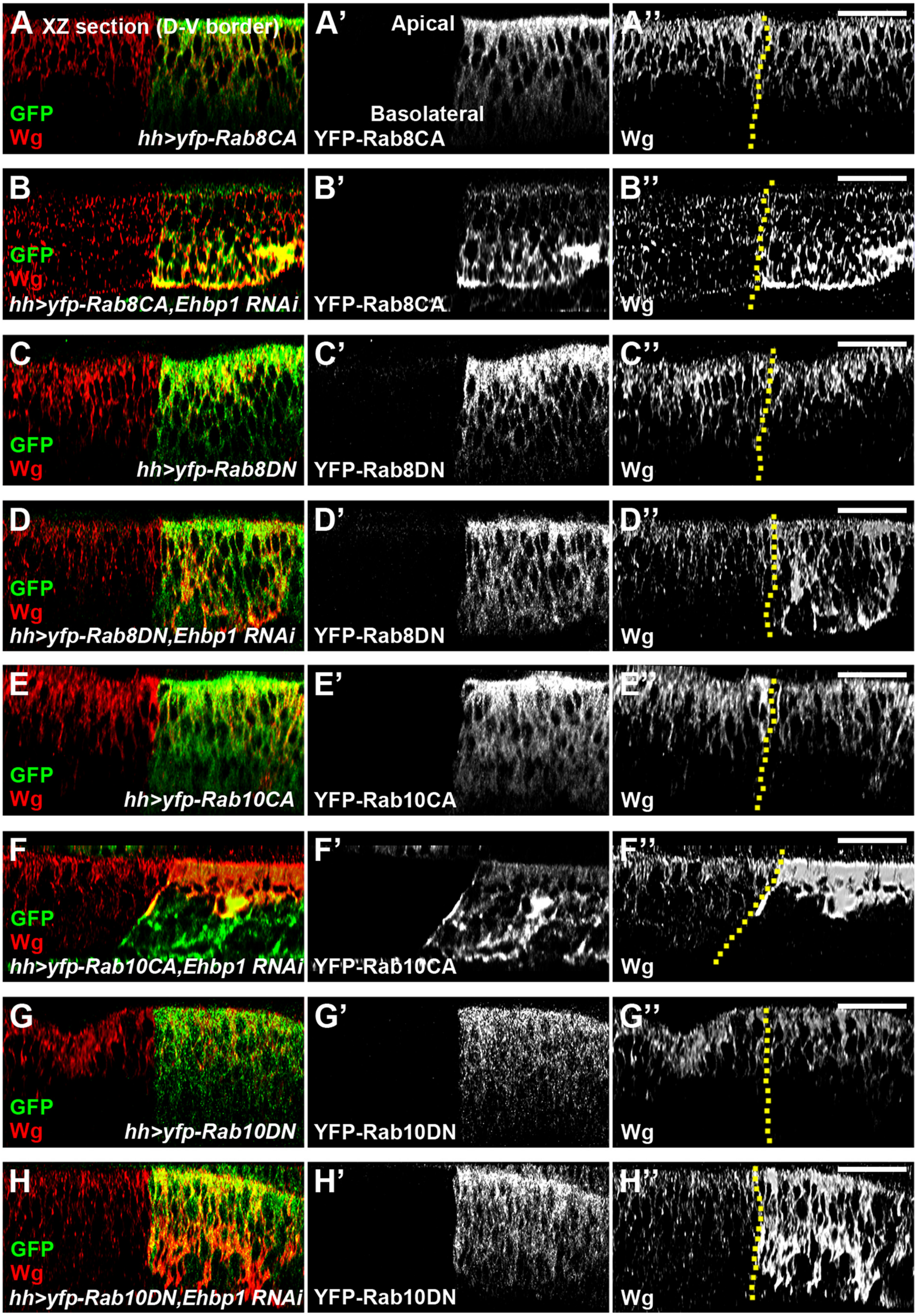
A XZ section (D-V border)
GFP
Wg
hh>yfp-Rab8CA
A'
Apical
Basolateral
YFP-Rab8CA
A''
Wg
B
GFP
Wg
hh>yfp-Rab8CA,Ehbp1 RNAi
B'
YFP-Rab8CA
B''
Wg
C
GFP
Wg
hh>yfp-Rab8DN
C'
YFP-Rab8DN
C''
Wg
D
GFP
Wg
hh>yfp-Rab8DN,Ehbp1 RNAi
D'
YFP-Rab8DN
D''
Wg
E
GFP
Wg
hh>yfp-Rab10CA
E'
YFP-Rab10CA
E''
Wg
F
GFP
Wg
hh>yfp-Rab10CA,Ehbp1 RNAi
F'
YFP-Rab10CA
F''
Wg
G
GFP
Wg
hh>yfp-Rab10DN
G'
YFP-Rab10DN
G''
Wg
H
GFP
Wg
hh>yfp-Rab10DN,Ehbp1 RNAi
H'
YFP-Rab10DN
H''
Wg

 © The Author(s)

Figure EV5. Neither Rab8 nor Rab10 is essential for the intracellular transport of Wg.

3D reconstructions of the D-V sections of the wing discs for the indicated genotypes are shown. Dotted yellow lines indicate the A-P boundaries. (**A–A″**) In cells where the constitutively active (CA) form of *Rab8* (*yfp-Rab8CA*) was overexpressed using the *hh-Gal4* driver, YFP-Rab8CA predominantly localized to the apical domain (**A**′). This did not appear to disrupt the polarized distribution of Wg (**A**″). (**B–B″**) When *Ehbp1* RNAi and *yfp-Rab8CA* were expressed using the *hh-Gal4* driver, YFP-Rab8CA was mainly found in the basolateral domain (**B**′), and Wg accumulated in the same domain. (**C–C″**) Overexpression of the dominant negative (DN) form of *Rab8* (*yfp-Rab8DN*) using the *hh-Gal4* driver resulted in YFP-Rab8DN being predominantly localized to the apical domain (**C**′), with no obvious defects in the polarized distribution of Wg (**C**″). (**D–D″**) Co-expression of *Ehbp1* RNAi and *yfp-Rab8DN* using the *hh-Gal4* driver showed no obvious defects in the distribution of YFP-Rab8DN (**D**′), although Wg accumulated in the basolateral domain (**D**″). (**E–E″**) The constitutively active (CA) form of *Rab10* (*yfp-Rab10CA*) overexpressed using the *hh-Gal4* driver was predominantly localized to the apical domain (**E**′), with no obvious impact on the polarized distribution of Wg (**E**″). (**F–F″**) When both *Ehbp1* RNAi and *yfp-Rab10CA* were expressed using the *hh-Gal4* driver, YFP-Rab10CA was mainly found in the basolateral domain (**F**′), while Wg accumulated in the apical domain (**F**″). (**G–G″**) When the dominant negative (DN) form of *Rab10* (*yfp-Rab8DN*) was overexpressed using the *hh-Gal4* driver, YFP-Rab10DN predominantly localized to the apical domain (**G**′), with no obvious defects observed in the polarized distribution of Wg (**G**″). (**H–H″**) Co-expression of *Ehbp1* RNAi and *yfp-Rab10DN* using the *hh-Gal4* driver showed no obvious defects in the distribution of YFP-Rab10DN (**H**′), although Wg accumulated in the basolateral domain (**H**″). Scale bars, 25 μm. Source data are available online for this figure.

© The Author(s)
